# Extracellular Vesicles Derived from M2 Microglia and Enriched in miR‑27b‑3p Attenuate Mitochondria‑Dependent Endothelial Apoptosis via the MKK4/JNK Pathway and Alleviate BBB Disruption After Intracerebral Hemorrhage

**DOI:** 10.1007/s12035-026-05718-x

**Published:** 2026-02-11

**Authors:** Junjie Gong, Jing Li, Di Wu, Anqi He, Kexin Li, Zhijuan Chen, Mingyu Zhao, Mengyao He, Yuchi Zhang, Jing Feng, Yuheng Liu, Zengguang Wang

**Affiliations:** 1 Department of Neurosurgery, Tianjin Medical University General Hospital, Tianjin, China; 2 Ministry of Education and Tianjin, Tianjin Neurological Institute, Key Laboratory of Post-Neuroinjury Neuro-Repair and Regeneration in Central Nervous System, Tianjin, China; 3 Department of Critical Care Medicine, Tianjin Medical University General Hospital, Tianjin, China; 4 Department of General Practice, Tianjin Medical University General Hospital, Tianjin, China; 5 Tianjin Medical University General Hospital, Tianjin, China

**Keywords:** Microglia, EVs, MicroRNA, Apoptosis, Endothelial cell, Blood–brain barrier, ICH

## Abstract

Intracerebral hemorrhage (ICH) is a life-threatening cerebrovascular disorder frequently accompanied by blood–brain barrier (BBB) disruption. Endothelial apoptosis is a key contributor to BBB damage, leading to loss of barrier function, exacerbation of brain edema and inflammation, and a cascade of adverse outcomes. Previous studies have shown that extracellular vesicles (EVs) released from M2-polarized microglia are enriched in neuroprotective miRNAs; however, their effects on endothelial cells after ICH and the underlying mechanisms remain unclear. This study aimed to determine the protective effects of M2‑EVs and their enriched miRNAs on vascular endothelial cells after ICH and to elucidate the relevant target genes and signaling pathways. Microglia were polarized to the M2 phenotype by IL‑4 stimulation, and EVs were isolated from M0‑ and M2‑polarized microglia. EVs were characterized by Western blotting, nanoparticle tracking analysis (NTA), and transmission electron microscopy (TEM). The effects of M2‑EVs on endothelial cells were evaluated in both *in vitro* and *in vivo* ICH models. *In vitro*, transendothelial electrical resistance, TUNEL staining, CCK‑8 assays, Western blotting, immunofluorescence, and TEM were used to assess endothelial apoptosis and tight junction integrity. *In vivo*, BBB disruption and brain edema after ICH were assessed by EB extravasation, MRI, and brain water content, and endothelial apoptosis and tight junction damage were further examined by Western blotting, immunofluorescence, and TEM. Neurological recovery was evaluated using neurological severity scores, rotarod and corner tests, and gait analysis. Candidate miRNAs were screened by sequencing, and their therapeutic effects, target genes, and downstream signaling pathways were validated. In both *in vitro* and *in vivo* ICH models, M2‑EVs reduced endothelial apoptosis, preserved tight junctions, and attenuated BBB disruption, thereby alleviating brain edema, limiting hematoma expansion and ameliorating neurological deficits in mice. miRNA sequencing identified miR‑27b‑3p, enriched in M2‑EVs, as a key mediator. miR‑27b‑3p directly targeted MKK4 and reduced phosphorylation of MKK4 and JNK in endothelial cells. This, in turn, increased the Bcl‑2/Bax ratio, helped maintain mitochondrial homeostasis, decreased mitochondrial release of cytochrome c (Cyt c), and lowered the expression of the downstream apoptotic effector Caspase‑3. As a result, endothelial apoptosis was suppressed and tight junction integrity was maintained, which mitigated BBB‑related neurological dysfunction after ICH. This study demonstrates that M2‑EVs, particularly those enriched in miR‑27b‑3p, protect the BBB after ICH by targeting the MKK4/JNK signaling pathway, increasing the Bcl‑2/Bax ratio and stabilizing mitochondrial function. These changes reduce Cyt c release and Caspase‑3 expression, thereby inhibiting endothelial apoptosis, preserving BBB integrity and improving neurological outcomes in mice at 3 days post‑ICH. Together, these findings suggest that miR‑27b‑3p carried by M2‑EVs represents a promising neurovascular protective strategy with considerable translational potential for the treatment of hemorrhagic stroke.

**Graphical Abstract:**

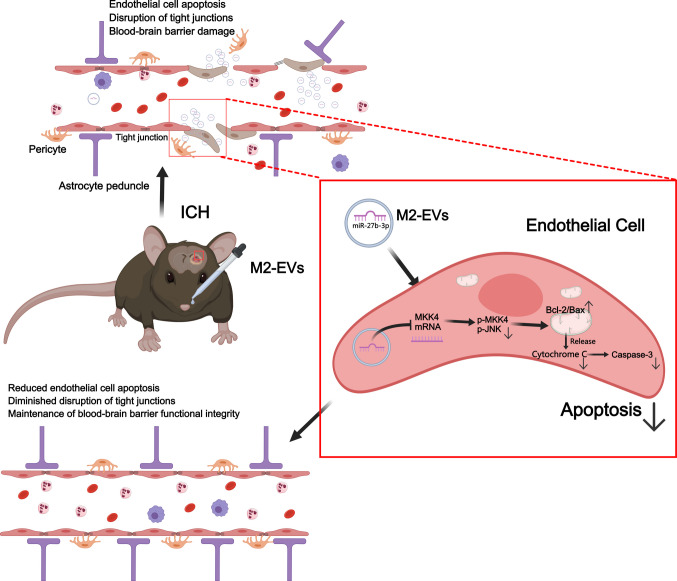

**Supplementary Information:**

The online version contains supplementary material available at 10.1007/s12035-026-05718-x.

## Introduction

Intracerebral hemorrhage (ICH) is a severe acute cerebrovascular disorder caused by the rupture of intracranial vessels and subsequent blood extravasation into the brain parenchyma. Although ICH accounts for only 10–15% of all stroke cases, its mortality and disability rates are markedly higher than those of ischemic stroke [1–3]. Brain injury after ICH can be divided into primary and secondary components [4, 5]. Primary injury is mainly determined by the initial hematoma volume and its expansion, whereas secondary injury involves inflammatory responses, oxidative stress, and cell apoptosis, all of which have a major impact on prognosis [6–8]. The core structural unit of the blood–brain barrier (BBB) is the brain microvascular endothelial cell, which maintains BBB integrity and cerebral homeostasis through tight and adherens junctions [9–11]. Following ICH, secondary injury leads to increased endothelial apoptosis and downregulation of tight junction proteins, resulting in BBB disruption and increased vascular permeability. This, in turn, aggravates brain edema and inflammation and impairs vascular and tissue repair [4, 12–17]. Therefore, preserving endothelial cell function and BBB integrity after ICH is a key strategy for limiting secondary damage to the central nervous system (CNS) and promoting neurological recovery.

Apoptosis is a crucial biological process that occurs following various brain injuries and typically proceeds through intrinsic (mitochondrial) and extrinsic (death receptor–mediated) pathways [18–20]. It serves to remove damaged cells and thereby prevents inflammation caused by cell lysis. However, excessive or dysregulated apoptosis can lead to marked tissue dysfunction and neurological impairment. Mitochondria play a central role in this process. As the primary energy producers, they are highly susceptible to diverse damaging stimuli. Under such stress, pro‑apoptotic proteins such as Bax and Bak insert into the mitochondrial outer membrane and alter its permeability, leading to the release of cytochrome c (Cyt c) into the cytosol. Cyt c then promotes the formation of the apoptosome, which recruits and activates Caspase‑9, thereby activating the downstream executioner Caspase‑3 and ultimately driving cell apoptosis. In contrast, anti‑apoptotic proteins such as Bcl‑2 and Bcl‑xL preserve mitochondrial membrane integrity by inhibiting Bax and Bak. This inhibition helps maintain mitochondrial function and enhances cellular resistance to apoptosis. Thus, by modulating the balance between pro‑ and anti‑apoptotic proteins, it is possible to influence cell fate and affect recovery processes following brain injury [11, 19–27].

Microglia are the principal immune cells of the CNS and play a pivotal role in the pathophysiology of ICH. After brain injury, they become activated, migrate, proliferate, and secrete a range of cytokines that can exert both deleterious and reparative effects on brain tissue [28–31]. Extracellular vesicles (EVs) are nano‑sized vesicles generated by inward budding of the plasma membrane and act as important carriers of paracrine signals [32–34]. Recent studies have shown that EVs released from M2 microglia contain multiple anti‑inflammatory factors and microRNAs (miRNAs), which together exert diverse protective effects on CNS cells after various types of brain injury [35]. Among the cargo of these EVs, miRNAs are particularly important. miRNAs are small non‑coding RNAs of approximately 21–25 nucleotides, widely present in animals, plants, and some viruses. By binding to the 3′ untranslated region (3′ UTR) of target mRNAs, they regulate gene expression at the post‑transcriptional level, inhibiting translation or promoting mRNA degradation. Through this mechanism, miRNAs play a crucial role in the regulation of cellular functions [36, 37].

Previous studies have shown that EVs released from M2 microglia are enriched in various miRNAs that target specific genes and reduce neuronal apoptosis after different types of brain injury [38–41]. However, the effects of M2 microglia‑derived EVs on endothelial cells have not been extensively investigated. Available evidence suggests that these EVs can enhance endothelial autophagy *in vitro* and thereby improve BBB integrity [42], whereas EVs derived from microglia exposed to lipopolysaccharide (LPS) or oxygen–glucose deprivation (OGD) have been reported to promote endothelial apoptosis [43, 44]. The temporal profile of apoptosis after ICH is not yet fully defined. Unlike traumatic brain injury or ischemic stroke, BBB disruption in ICH is driven mainly by hematoma expansion and toxic blood components, which further aggravate hematoma enlargement and amplify inflammatory responses. Thus, reducing endothelial apoptosis and BBB disruption after ICH may be critical for limiting hematoma size, attenuating secondary injury, and promoting neurological recovery [4, 31]. Most existing studies have focused on regulating neuronal apoptosis after brain injury, whereas the protective effects of M2‑EVs on endothelial cells after ICH, particularly in the context of apoptosis regulation, remain poorly understood.

Based on this background, the present study aimed to investigate, in both *in vitro* and *in vivo* models of ICH, the direct effects of M2‑EVs on cerebrovascular endothelial apoptosis and BBB integrity, and to elucidate the underlying molecular mechanisms, with particular emphasis on the role of miRNAs carried within M2‑EVs. 

## Materials and Methods

### Cultivation and Pretreatment of Microglial Cells

The murine BV2 microglial cell line (Meisen, China) was cultured in Dulbecco’s Modified Eagle Medium (DMEM; Gibco, USA) supplemented with 10% fetal bovine serum (Cell‑box, China) and 1% penicillin–streptomycin (Solarbio, China). Cells were maintained at 37 °C in a humidified incubator with 5% CO₂ and 95% air. BV2 cells were seeded at a density of 1 × 10^5^ cells/mL and then stimulated with 20 ng/mL interleukin‑4 (IL‑4; Procell, China) for 48 h [38, 45, 46]. After stimulation, the microglial cells were characterized by immunofluorescence staining, qPCR, and Western blot analysis.

### Cultivation of Primary Mouse Brain Microvascular Endothelial Cells

Primary mouse brain microvascular endothelial cells (Meisen, China) were cultured in Endothelial Cell Medium (ECM; ScienCell, USA) supplemented with 10% fetal bovine serum, 1% penicillin–streptomycin, and 0.1% endothelial cell growth supplement (ECGS; ScienCell, USA). Cells were maintained at 37 °C in a humidified incubator with 5% CO₂ and 95% air. When cultures reached 80%–90% confluence, the cells were harvested and used for subsequent experiments.

### Isolation, Characterization and Labeling of EVs

After stimulation of BV2 microglia with IL‑4 for 48 h to induce M2 polarization, the cells were washed twice with PBS and then cultured in serum‑free medium for an additional 48 h. The conditioned medium was collected and sequentially centrifuged at 300 g for 10 min, 2000 g for 10 min, and 10,000 g for 30 min to remove cells and debris, followed by ultracentrifugation at 100,000 g for 70 min to pellet EVs. To remove contaminating proteins, the EV pellet was washed once with PBS and ultracentrifuged again at 100,000 g for 70 min. Purified EVs were resuspended in PBS and stored at − 80 °C until use. EV protein concentration was determined using a BCA protein assay kit (Thermo Fisher Scientific, USA). EV size and concentration were assessed by nanoparticle tracking analysis (NTA) and transmission electron microscopy (TEM). Western blotting was used to detect EV marker proteins CD9, CD63, and Hsp70. For fluorescent labeling, EVs were incubated with the PKH26 red fluorescent cell linker kit (Solarbio, China). Briefly, EVs were mixed with 2 μL PKH26 dye in 500 μL Diluent C and incubated at room temperature in the dark for 4 min. Excess dye was quenched by adding 200 μL fetal bovine serum, and labeled EVs were washed with PBS by ultracentrifugation at 100,000 g for 70 min. The final EV pellet was resuspended in PBS for subsequent *in vitro* and *in vivo* experiments.

### Preparation of Hemin Solution and Determination of Intervention Conditions

A 1 mM hemin chloride stock solution was prepared by dissolving hemin chloride (MedChem Express, China) in a mixture of 0.2% (v/v) DMSO (Solarbio, China), 2% (v/v) ammonia (Solarbio, China), and 97.8% (v/v) DMEM. A previous study reported that endothelial cells treated with 0, 200, 400, or 600 µM hemin for 8 h showed survival rates of approximately 100%, 70%, 44%, and 20%, respectively [47]. In light of these data, we conservatively selected 100 µM hemin as the fixed concentration for subsequent experiments. To determine the optimal exposure time, cell viability was measured by CCK‑8 assay after 6, 12, 24, 36, 48, and 72 h of hemin treatment. The time point at which cell viability decreased to approximately 50% was chosen as the intervention duration.

### Cell Viability Assay

Cell viability was assessed using the Cell Counting Kit‑8 (CCK‑8; Solarbio, China). Briefly, primary mouse brain microvascular endothelial cells were treated with hemin in 96‑well plates, after which the culture medium was replaced with serum‑free medium. Then, 10 µL of CCK‑8 solution was added to each well and the plates were incubated for 2 h. Absorbance was measured at 450 nm using a microplate reader. Cell viability was calculated according to the following formula: Cell viability (%) = [OD(hemin) − OD(cell‑free)]/[OD (no hemin) − OD (cell‑free)] × 100%.

### Measurement of Transendothelial Electrical Resistance

To assess endothelial barrier function *in vitro*, transendothelial electrical resistance (TEER) was measured in primary mouse brain microvascular endothelial cell monolayers. TEER was recorded using an EVOM3 volt/ohm meter (World Precision Instruments, Sarasota, FL, USA). For each group, the TEER value of a blank insert was subtracted from the measured value of the corresponding cell‑covered insert. All TEER values are expressed in Ω cm^2^.

### TUNEL Assay

After immunofluorescent labeling of primary mouse brain microvascular endothelial cells, apoptotic cells were detected using a TUNEL apoptosis detection kit (green fluorescence; Absin, China). According to the manufacturer’s instructions, the TUNEL reaction mixture was prepared and 50 μL was added to each sample to ensure complete coverage. Samples were incubated at 37 °C in the dark for 30 min and then thoroughly washed with PBS. Finally, the slides were mounted and examined under a fluorescence microscope.

### Animals

All animals used in this study were purchased from Beijing Vital River Laboratory Animal Technology Co., Ltd. (License No. SCXK (Jing) 2021‑0006). To avoid potential confounding effects of sex, only male C57BL/6 mice aged 8–10 weeks and weighing 22–25 g were included. Mice were housed under controlled conditions at 18–22 °C with 50%–60% humidity, on a standard 12‑h light/dark cycle, with free access to food and water. All animal procedures were approved by the Animal Care and Use Committee of Tianjin Medical University General Hospital (Approval No. IRB2025‑DWFL‑104) and were conducted in accordance with the ARRIVE guidelines. Mice were randomly assigned to experimental groups and received the corresponding treatments.

### ICH Modeling

ICH was induced using a collagenase model as previously described [48]. Mice were anesthetized with isoflurane (5% for induction, 2% for maintenance; 792632 Sigma‑Aldrich). After shaving and disinfection, the head was fixed in a stereotaxic frame. A burr hole was drilled 2.0 mm lateral to the right of bregma and 0.5 mm anterior to bregma. A microsyringe (33‑gauge, 10 mm; Hamilton) preloaded with collagenase VII (0.1 U/μL; C2399, Sigma‑Aldrich) was mounted on a microinjection pump (RWD Life Science, Shenzhen, China), and the needle was advanced vertically to a depth of 3.5 mm. Collagenase was injected at 0.2 μL/min to a total volume of 0.5 μL. After injection, the needle was left in place for 5 min before being slowly withdrawn. The skin was disinfected again, sutured, and the mice were allowed to recover on a heated pad. All mice, except those in the sham group, underwent the procedure described above. Sham mice received a 0.5 μL intracerebral injection of PBS instead of collagenase VII using the same coordinates and procedure. Contralateral limb deficits (hemiparesis) were monitored postoperatively, and neurological function was assessed at 24 h using the modified neurological severity score (mNSS) to confirm successful ICH modeling.

### EVs Administration and Brain Labeling

Following collagenase‑induced ICH, EVs derived from M0 and M2 microglia were administered intranasally at 1 h, 1 day, and 2 days after injury. A suspension containing 10^8^ PKH26‑labeled EVs in 10 μL PBS was slowly applied to the right nostril to allow natural inhalation. Mice that did not receive EVs were given 10 μL PBS as controls. On day 1 after hemorrhage, mice were anesthetized and sacrificed, and brains were harvested for frozen sectioning. Brain vascular endothelial cells were then labeled with CD31 to assess the uptake of EVs by endothelial cells after ICH.

### Assessment of Neurological Deficits

To quantify neurological function in ICH mice receiving different treatments, a battery of standardized behavioral tests was performed. All assessments were carried out by two investigators blinded to group allocation. Neurological function was evaluated on days 1 and 3 after successful ICH induction. The mNSS was used to assess motor, sensory, reflex, and balance functions on both days 1 and 3 post‑ICH. The total score ranges from 0 to 18, with higher scores indicating more severe neurological deficits.

### Rotarod Test

Motor coordination was evaluated using an accelerating rotarod. Mice were placed on the rotating rod, which accelerated from 4 to 40 revolutions per minute over 300 s, and the latency to fall was recorded. Before surgery, mice were trained for 3 consecutive days, with three trials per day. On days 1 and 3 after surgery, three test trials were performed each day, and the mean latency was used for statistical analysis. A shorter latency to fall indicated poorer motor coordination and balance.

### Corner Test

In the corner test, mice were placed between two glass plates forming a 30° angle, which forced them to turn either left or right, and the turning direction was recorded. Each mouse underwent 10 trials with an interval of at least 30 s between trials. A higher frequency of turning toward the ipsilateral (injured) side was interpreted as indicating greater sensorimotor impairment.

### Gait Analysis

On day 3 after ICH, gait was assessed using the CatWalk XT system. Mice were allowed to walk freely along a glass walkway (21 cm in length and 3.6 cm in width), and runs were recorded with the CatWalk XT software. Only runs in which mice traversed the track within 4 s were accepted for analysis. To evaluate motor dysfunction, we analyzed the maximum paw pressure, maximum contact area, and single‑stance phase of the affected limbs during contact with the walkway.

### Magnetic Resonance Imaging

MRI scans were performed on mice at 1 and 3 days post‑ICH. Mice were anesthetized with isoflurane and subjected to coronal T2‑weighted imaging using a 9.4 T MRI scanner (Bruker BioSpec 94/30) to visualize the hematoma core and the surrounding perihematomal edema. Hematoma volume was calculated using the following formula: hematoma volume = (maximum cross‑sectional area of the coronal hematoma focus) × (number of hematoma slices) × 0.5.

### Evaluation of Brain Water Content

Brain water content was measured as previously described [49]. Briefly, mice were deeply anesthetized and sacrificed by cervical dislocation 72 h after ICH. Brains were rapidly removed and divided into three regions: the ipsilateral hemisphere containing the hemorrhagic lesion, the contralateral hemisphere, and the cerebellum. The wet weight of each sample was immediately measured using an analytical microbalance (Mettler Toledo, Columbus, OH, USA). Samples were then dried at 100 °C for 24 h to obtain the dry weight. Brain water content was calculated using the following formula: Brain water content (%) = (wet weight − dry weight)/wet weight × 100%.

### Assessment of BBB Integrity

Disruption of the BBB was evaluated using the Evans Blue (EB) permeability assay, as previously described [50]. Briefly, on day 3 after ICH induction, EB (2% solution, 2 mL/kg; Solarbio, China) was administered via tail vein injection. Two hours later, mice were perfused with saline, after which the ipsilateral brain tissue was collected, weighed, and homogenized in PBS. The homogenates were incubated with formamide at 60 °C for 72 h to extract EB. After centrifugation, the absorbance of the supernatant was measured at 600 nm using a spectrophotometer (Thermo Fisher Technology, Waltham, MA, USA) to determine EB concentration. The amount of EB in brain tissue (g/g wet brain) was calculated using the formula: EB content (g/g wet brain) = EB concentration (g/mL) × formamide volume (mL)/wet weight (g).

### Western Blot Analysis

For cell lysates, primary mouse brain microvascular endothelial cells were treated with hemin for 24 h, washed three times with PBS to remove residual hemin, and lysed on ice for 20 min in RIPA buffer. The lysates were scraped, centrifuged at 13,000 rpm for 20 min, and the supernatants were collected. For tissue samples, mice were deeply anesthetized on day 3 after ICH and euthanized by cardiac perfusion with ice‑cold PBS. Peri‑hematomal brain tissue was rapidly dissected, snap‑frozen in liquid nitrogen, weighed, homogenized in RIPA buffer (volume proportional to tissue weight), and centrifuged at 13,000 rpm for 20 min. Protein concentrations in all supernatants were determined using a BCA Protein Assay Kit (Thermo Fisher Scientific, USA).

For Western blotting, 50 µg protein per lane was loaded for both cell and tissue samples. Proteins were separated by SDS‑PAGE and transferred to PVDF membranes (Millipore Sigma, DE). Membranes were blocked, incubated with primary antibodies (see below) overnight at 4 °C, washed with TBST and then incubated with HRP‑conjugated secondary antibodies at room temperature for 1 h. Bands were visualized using a chemiluminescence detection kit (Thermo Fisher Scientific, USA). Band intensity was quantified with ImageJ software and normalized to β‑Tubulin, β‑Actin, or GAPDH.

Primary antibodies: CD206 (1:1000, Cell Signaling Technology, #24595), Arg‑1 (1:500, Santa Cruz, sc‑81154), CD9 (1:1000, Cell Signaling Technology, #98327), CD63 (1:1000, Biorbyt, orb11317), Hsp70 (1:1000, Selleck, F1607), Claudin‑5 (1:1000, Invitrogen, 35‑2500), Occludin (1:1000, Cell Signaling Technology, #91131), VE‑cadherin (1:1000, Abcam, ab318152), Caspase‑3 (1:1000, Cell Signaling Technology, #14220), Bax (1:1000, Cell Signaling Technology, #2772), Bcl‑2 (1:1000, Cell Signaling Technology, #3498), MKK4 (1:1000, Selleck, F1499), p‑MKK4 (1:1000, Cell Signaling Technology, #9156), JNK (1:1000, Cell Signaling Technology, #9252), p‑JNK (1:1000, Cell Signaling Technology, #4668), β‑Tubulin (1:1000, Abclonal, A12289), β‑Actin (1:10,000, Abclonal, AC026), GAPDH (1:10,000, Abclonal, A19056).

Secondary antibodies: HRP‑conjugated goat anti‑rabbit IgG (H + L) and HRP‑conjugated goat anti‑mouse IgG (H + L) (Invitrogen, USA).

### Immunofluorescence Staining and Quantitative Analysis

Immunofluorescence was performed on paraffin‑embedded brain sections and on primary mouse brain microvascular endothelial cells. Brain sections were deparaffinized, subjected to antigen retrieval, washed in TBST, blocked with 10% donkey serum at 37 °C, and incubated overnight at 4 °C with primary antibodies diluted in TBST. After washing, sections were incubated with fluorescent secondary antibodies, counterstained with DAPI, mounted with antifade medium, and imaged using a fluorescence microscope (Leica, USA). For cell staining, treated coverslips in 12‑well plates were washed with PBS, fixed with 4% formaldehyde, permeabilized with 0.2% Triton X‑100, blocked with 5% bovine serum albumin, and incubated overnight at 4 °C with primary antibodies. The following day, cells were washed, incubated with fluorescent secondary antibodies at room temperature, stained with DAPI, mounted, and imaged with a laser confocal microscope (Olympus, Japan). Primary antibodies used for immunofluorescence were: Iba‑1 (1:1000, Abcam, ab289874), CD206 (1:1000, Cell Signaling Technology, #24595), CD31 (1:200, R&D, AF3628), Claudin‑5 (1:200, Invitrogen, 352588), Occludin (1:200, Cell Signaling Technology, #91131), and Caspase‑3 (1:100, Proteintech, 19677‑1‑AP). Nuclei were stained with DAPI dihydrochloride (Thermo Fisher Scientific, USA).

For quantitative analysis, brains were collected on day 3 after ICH and processed into paraffin‑embedded sections (*n* = 6 per group). For each mouse, three non‑consecutive coronal sections at the striatal level were selected, and each selected section was required to contain the hematoma. These sections were stained for Caspase‑3, Claudin‑5, or Occludin, and three non‑overlapping peri‑hematomal fields were imaged per section for subsequent statistical analysis.

Endothelial apoptosis was quantified by colocalization of CD31 and Caspase‑3. Two blinded investigators independently counted cells, and the mean value was used. Endothelial apoptosis rate (%) = (number of CD31/Caspase‑3 double‑positive cells ÷ total number of CD31‑positive cells) × 100%.

Tight junction protein expression was quantified in ImageJ by manually outlining CD31-positive microvessels and calculating the mean fluorescence intensity of the target protein within these regions.

### Transmission Electron Microscopy

For TEM, cultured cells and peri‑hematomal brain tissue were processed using standard protocols. Cells were gently scraped from 10‑cm dishes, pelleted at 1,000 rpm, washed in 0.1 M phosphate buffer (PB, pH 7.4), and embedded in 1% agarose; the solidified cell blocks were cut into ~ 1 mm^3^ pieces. For brain samples, mice were deeply anesthetized, perfused with PBS, and brains were quickly removed. Hematoma and surrounding tissue were dissected, cut into ~ 1 mm^3^ blocks, and transferred into electron microscopy fixative at 4 °C. Cell and tissue blocks were rinsed in 0.1 M PB and post‑fixed in 1% osmium tetroxide in 0.1 M PB for 2 h at room temperature in the dark, then washed again in PB. Samples were dehydrated through a graded ethanol series (30%–100%), followed by two changes of 100% acetone, and infiltrated sequentially with acetone/812 resin mixtures and finally pure 812 resin. Resin‑embedded specimens were cured and polymerized, and 70‑nm ultrathin sections were cut with an ultramicrotome and collected on 200‑mesh copper grids. Sections were stained with 2% uranyl acetate in ethanol and lead citrate, rinsed with ultrapure water, air‑dried, and examined under a transmission electron microscope.

### miRNA Sequencing and Target Gene Prediction

miRNA sequencing of M0‑EVs (baseline culture) and IL‑4-stimulated M2‑EVs was performed by Lianchuan BioTech (China) according to the company’s standard protocols for library preparation and high‑throughput sequencing. Differentially expressed miRNAs between M0‑EVs and M2‑EVs were identified based on fold‑change in expression, and bioinformatic prediction of target genes was subsequently carried out for these miRNAs.

### Real‑Time Quantitative PCR

Total RNA was extracted from cells and EVs using TRIzol reagent (Invitrogen, USA) according to the manufacturer’s instructions. RNA quantity and purity were assessed with a NanoDrop ND‑1000 spectrophotometer (Thermo Fisher Scientific, USA). Single‑stranded cDNA was synthesized using a universal cDNA synthesis kit (TransGen Biotech, China). qPCR was performed on a QuantStudio 3 Fast Real‑Time PCR system (Applied Biosystems, USA) using SYBR Green Master Mix (TransGen Biotech, China). U6 was used as an internal control for EV miRNA, and GAPDH was used as the reference gene for tissue and cell samples. Relative expression levels were normalized to the control group.

### Lentiviral Transduction

BV2 microglial cells that had been polarized to the M2 phenotype were transduced for 72 h with either an hU6‑MCS‑CBh‑gcGFP‑IRES‑puromycin‑mmu‑miR‑27b‑3p inhibitor lentivirus (miR-27b-3p k/d) or the corresponding inhibitor negative control lentivirus (miR-27b-3p cn; Genechem, Shanghai, China), according to the manufacturer’s instructions. After transduction, cells were selected with puromycin (Thermo Fisher Scientific, USA), and GFP expression was examined by fluorescence microscopy to verify transduction efficiency.

### Dual‑Luciferase Reporter Assay

miRNAs generally regulate target genes by binding to the 3′ untranslated region (3′ UTR) of their mRNAs. To verify whether miR-27b-3p directly targets Grb2 and Mkk4, the wild‑type 3′ UTR fragments of Grb2 and Mkk4 were cloned into luciferase reporter vectors and transfected into primary mouse brain microvascular endothelial cells. Site‑directed mutagenesis of the predicted miR-27b-3p binding sites within these 3′ UTRs was performed to further confirm the specificity of the interaction. Luciferase activity was measured using the Dual‑Luciferase® Reporter Assay System (Promega, Fitchburg, WI). The ratio of Renilla luciferase (OBIO, China) to firefly luciferase activity was calculated to determine whether miR‑27b‑3p specifically inhibits these target genes.

### Statistical Analysis

Statistical analyses were performed using GraphPad Prism 10. Quantitative data are presented as mean ± standard deviation (mean ± SD). Differences between two groups were analyzed with an unpaired t‑test, whereas multiple group comparisons were evaluated by one‑way ANOVA followed by Tukey’s or Dunnett’s post hoc tests as appropriate; in some cases, Dunnett’s multiple comparisons test was specifically applied, as indicated in the figure legends. A *p *value < 0.05 was considered statistically significant. All cell experiments were performed in at least three independent replicates, and animal experiments included 6 mice per group. Exact *p* values and sample sizes (*n*) are reported in the figures and corresponding figure legends.

## Results

### Characterization of M2‑Polarized Microglia and Their Derived EVs

**Fig. 1:**
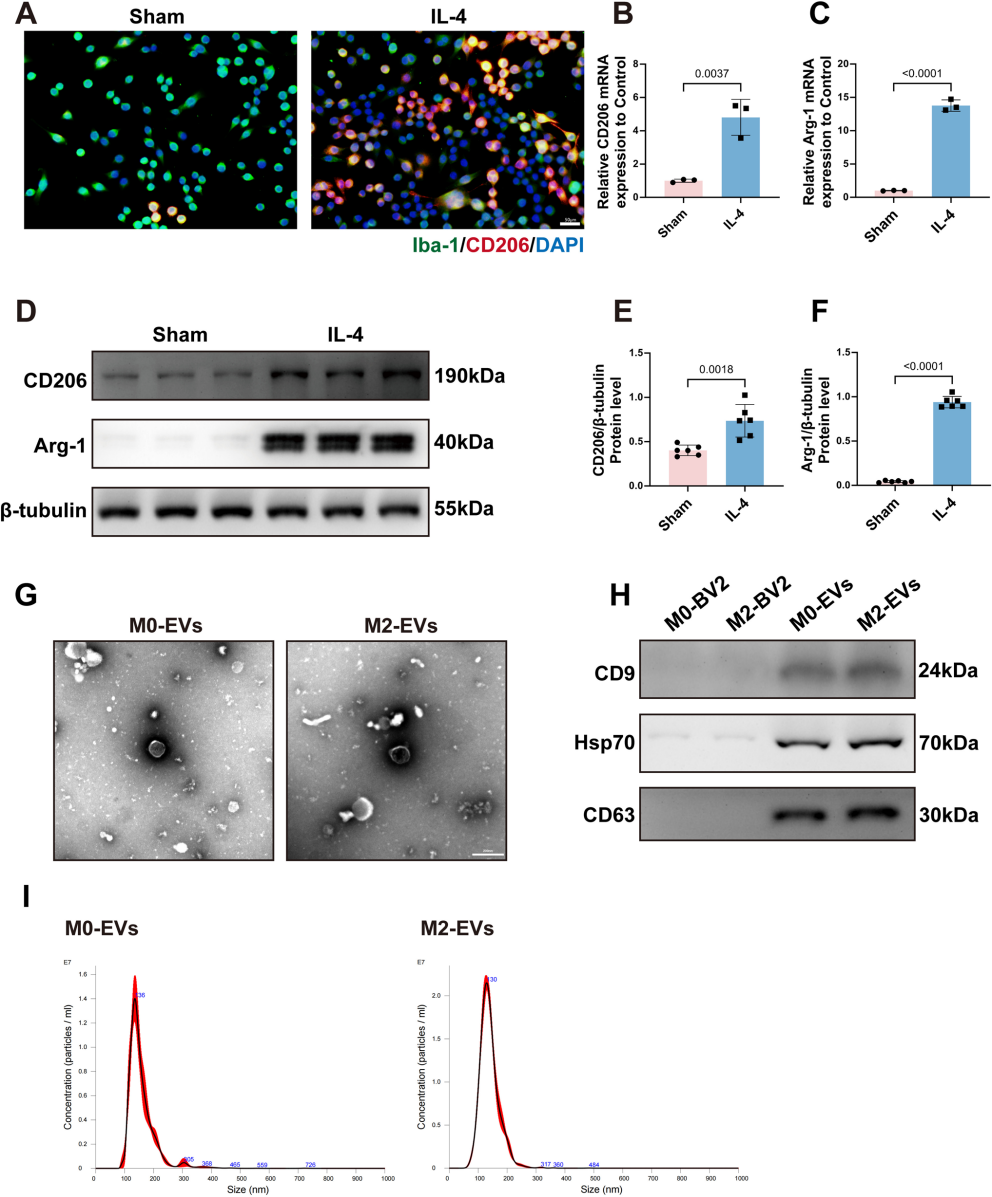
IL‑4-induced microglial polarization toward the M2 phenotype and characterization of microglia‑derived EVs.** A** Representative immunofluorescence images showing a significant increase in CD206‑positive microglia in the IL‑4-treated group compared with the sham group. Scale bar, 50 µm. **B**,** C** qPCR analysis of mRNA expression levels of the M2 markers CD206 and Arg‑1 (CD206: *t*(4) = 6.095, *p* = 0.0037; Arg‑1: *t*(4) = 26.04, *p* < 0.0001; *n* = 3). **D** Representative Western blot images showing increased protein expression of CD206 and Arg‑1 in microglia following IL‑4 stimulation. **E**, **F** Quantification of CD206 and Arg‑1 protein levels (CD206: *t*(10) = 4.224, *p* = 0.0018; Arg‑1: *t*(10) = 33.47, *p* < 0.0001; *n* = 6). **G** TEM images of M0‑EVs and M2‑EVs displaying typical vesicular morphology. Scale bar, 200 nm. **H** Western blot analysis of the EV marker proteins CD9, CD63, and HSP70 in EVs derived from M0‑ and M2‑polarized microglia and in the corresponding cell lysates. **I** NTA of M0‑EVs and M2‑EVs showing particle concentration profiles. All quantitative data are presented as mean ± SD, and statistical comparisons were performed using unpaired two‑tailed *t*‑tests

BV2 cells were treated with 20 ng/mL IL‑4 for 48 h to induce polarization toward an M2 phenotype, as described previously. Immunofluorescence staining revealed a marked increase in the proportion of CD206‑positive cells following IL‑4 treatment (Fig. 1A). Consistently, qPCR analysis showed significant upregulation of mRNA expression for the M2 markers CD206 and Arg‑1 (Fig. 1B, C). At the protein level, Western blot analysis yielded similar findings, with increased expression of both CD206 and Arg‑1 (Fig. 1D–F). Together, these results indicate that the M2 polarization model was successfully established. EVs were subsequently isolated from the conditioned medium of untreated (M0) and IL‑4‑induced M2 microglia and designated as M0‑EVs and M2‑EVs, respectively, with M0‑EVs serving as the experimental control. TEM revealed no apparent differences in morphology or size distribution between the two EV groups (Fig. 1G). Western blot analysis demonstrated that the isolated vesicles were enriched in the canonical EV marker proteins CD9, CD63, and HSP70, whereas these proteins were not comparably enriched in the corresponding cell lysate controls (Fig. 1H). Nanoparticle tracking analysis further showed that the particle concentrations of M0‑EVs and M2‑EVs were 1.45 × 10^8^ particles/mL and 2.24 × 10^8^ particles/mL, respectively (Fig. 1I).

### M2‑EVs Alleviate Hemin‑Induced Endothelial Apoptosis and Improve Tight Junction Integrity

**Fig. 2:**
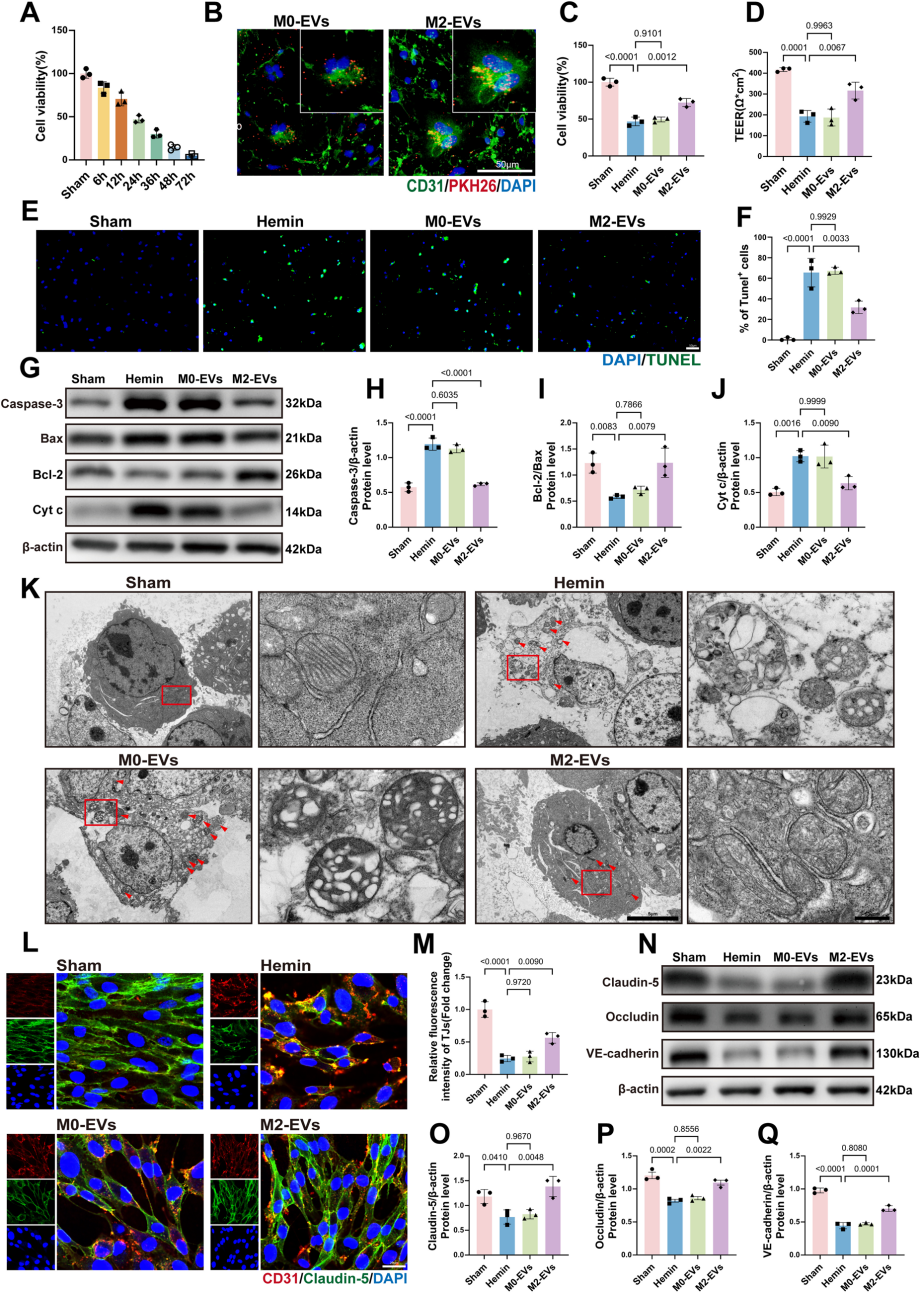
M2‑EVs protect against hemin‑induced endothelial injury by attenuating apoptosis and preserving junctional integrity.** A** Endothelial cell viability following treatment with 100 µM hemin for different durations, assessed using the CCK‑8 assay. **B** Fluorescence microscopy images showing uptake of PKH26‑labeled (red) M0‑EVs and M2‑EVs by endothelial cells. **C**, **D** Quantitative analysis of cell viability (*F*(3, 8) = 71.12,* p* < 0.0001; **C**) and TEER (*F*(3, 8) = 34.86, *p* < 0.0001; **D**; *n* = 3), indicating that treatment with M2‑EVs increases cell viability and improves TEER compared with the hemin‑injured group. **E**,** F** Evaluation of apoptosis by TUNEL staining (scale bar, 50 µm) and its quantification (*F*(3, 8) = 48.75, *p* < 0.0001; *n* = 3), showing that M2‑EVs reduce the proportion of TUNEL‑positive cells. **G** Representative Western blot bands of apoptosis‑related proteins Caspase‑3, Bax, Cyt c, and Bcl‑2. **H**–**J** Quantitative analysis of apoptosis‑related protein expression (Caspase‑3: *F*(3, 8) = 82.42,* p* < 0.0001; Bcl‑2/Bax: *F*(3, 8) = 11.41, *p* = 0.0029; Cyt c: *F*(3, 8) = 18.46, *p* = 0.0006; *n* = 3), demonstrating that treatment with M2‑EVs lowers Caspase‑3 and Cyt c levels while increasing the Bcl‑2/Bax ratio compared with the hemin group. **K** TEM images showing mitochondrial morphological changes associated with the apoptotic pathway, including swelling and loss of cristae (red arrows indicate abnormal mitochondria). Scale bar, 5.0 µm (inset scale bar, 500 nm). **L**,** M** Immunofluorescence co‑staining of CD31 (red) and Claudin‑5 (green) to evaluate tight junctions (scale bar, 20 µm), with quantification (*F*(3, 8) = 48.21, *p* < 0.0001) indicating that M2‑EVs alleviate Claudin‑5 disruption. **N** Representative Western blot images of the tight junction proteins Claudin‑5 and Occludin and the adherens junction protein VE‑cadherin. **O**–**Q** Quantitative analysis of junctional protein expression (Claudin‑5: *F*(3, 8) = 11.27, *p* = 0.003; Occludin: *F*(3, 8) = 28.85, *p* = 0.0001; VE‑cadherin: *F*(3, 8) = 128.2, *p* < 0.0001; *n* = 3), showing that M2‑EVs upregulate Claudin‑5, Occludin, and VE‑cadherin compared with the hemin group. Data are presented as mean ± SD. One‑way ANOVA with Tukey’s post hoc test was used for multiple‑group comparisons

To establish a hemin injury model, endothelial cells were exposed to 100 µM hemin. CCK‑8 analysis showed that cell viability decreased to about 50% after 24 h (Fig. 2A), and this condition was used in subsequent experiments. When hemin‑injured endothelial cells were co‑incubated with PKH26‑labeled M0‑EVs or M2‑EVs, fluorescence microscopy confirmed efficient internalization of both types of EVs (Fig. 2B). However, only M2‑EVs significantly attenuated hemin‑induced endothelial injury: treatment with M2‑EVs increased cell viability in the CCK‑8 assay (Fig. 2C) and increased TEER (Fig. 2D). Consistent with these findings, TUNEL staining revealed a marked reduction in apoptotic cells after treatment with M2‑EVs (Fig. 2E, F). Western blot analysis further revealed that M2‑EVs reduced the expression of the pro‑apoptotic proteins Caspase‑3, Bax, and Cyt c, while increasing the anti‑apoptotic protein Bcl‑2, thereby elevating the Bcl‑2/Bax ratio (Fig. 2G–J). In parallel, TEM showed mitochondrial swelling and cristae disruption in the hemin group and in groups treated with M0‑EVs—morphological features characteristic of apoptosis—whereas mitochondrial ultrastructure was largely preserved in the M2‑EVs‑treated group (Fig. 2K). M2‑EVs also protected endothelial barrier integrity. Quantitative immunofluorescence demonstrated that M2‑EVs mitigated hemin‑induced disruption of the tight junction protein Claudin‑5 (Fig. 2L, M). Western blotting further confirmed increased expression of the tight junction proteins Claudin‑5 and Occludin, as well as the adherens junction protein VE‑cadherin, in M2‑EVs‑treated cells (Fig. 2N–Q). Taken together, these data indicate that, in this *in vitro* model of ICH, M2‑EVs alleviate hemin‑induced endothelial apoptosis and help preserve BBB tight junction integrity, likely by modulating the Bcl‑2/Bax balance, maintaining mitochondrial homeostasis, and limiting Caspase‑3‑dependent apoptotic signaling.

### M2‑EVs Attenuate Cerebral Edema and Improve Neurological Function at 3 Days Post‑ICH in Mice

**Fig. 3:**
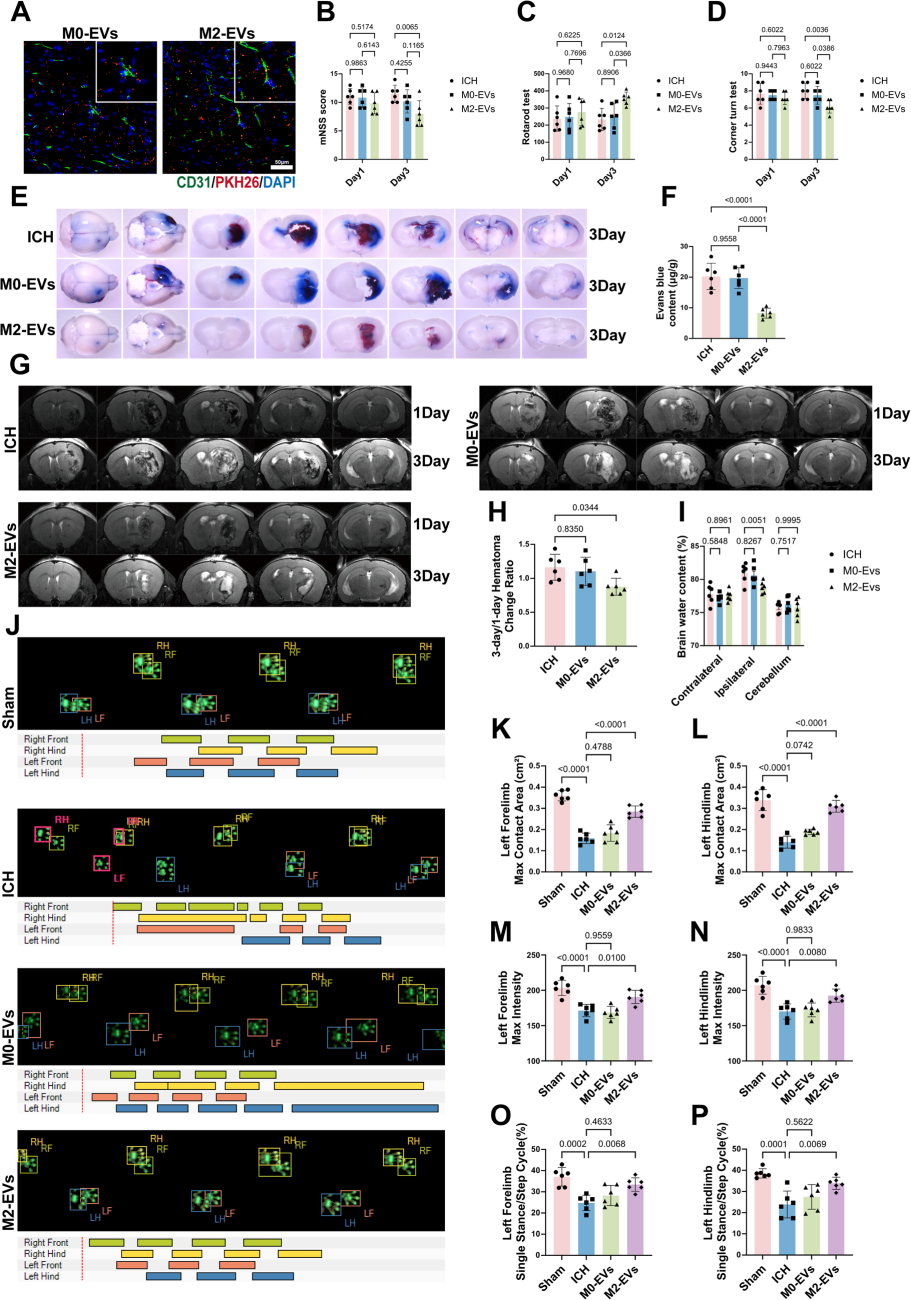
M2‑EVs alleviate cerebral edema and improve neurological function after ICH in mice. **A **Uptake of PKH26‑labeled EVs (red) by cerebrovascular endothelial cells (CD31, green) in the mouse brain after intranasal administration. **B**–**D** Neurological and behavioral assessments at day 3 post‑ICH using the mNSS (*F*(2, 30) = 5.108, *p* = 0.0123; *n* = 6), rotarod test (*F*(2, 30) = 4.562, *p* = 0.0186; *n* = 6), and corner test (*F*(2, 30) = 5.417, *p* = 0.0098; *n* = 6). Treatment with M2‑EVs significantly improved neurological function compared with the ICH group. **E **Representative brain slices from the EB extravasation assay. **F **Quantification of EB content in brain tissue (*F*(2, 15) = 25.38, *p* < 0.0001; *n* = 6), showing that M2‑EVs markedly reduced peri‑hematomal EB leakage on day 3. **G **Representative MRI scans on days 1 and 3 after ICH. **H **Quantitative analysis of the change rate in hematoma volume from day 1 to day 3 (*F*(2, 15) = 4.351, *p* = 0.0323; *n* = 6), indicating that M2‑EVs significantly limited hematoma expansion by day 3. **I **Quantitative analysis of brain water content in the ipsilateral hemisphere on day 3 (*q* = 3.182, *p* = 0.0051; *n* = 6), demonstrating that treatment with M2‑EVs significantly reduced ipsilateral brain water content. **J **Schematic diagram of the gait analysis setup. LF, left forelimb; LH, left hindlimb; RF, right forelimb; RH, right hindlimb. **K**–**P** Quantitative gait analysis of the contralateral (left) limbs, including maximum contact area (LF: *F*(3, 20) = 60.09, *p* < 0.0001; LH: *F*(3, 20) = 51.95, *p* < 0.0001), maximum paw pressure (LF: *F*(3, 20) = 18.42, *p* < 0.0001; LH: *F*(3, 20) = 16.37, *p* < 0.0001), and the ratio of single‑limb stance time to gait cycle duration (LF: *F*(3, 20) = 10.98, *p* = 0.0002; LH: *F*(3, 20) = 11.83, *p* = 0.0001; *n* = 6). Treatment with M2‑EVs significantly improved all of these parameters in both the left forelimb and left hindlimb. Data are presented as mean ± SD. Multiple‑group comparisons were analyzed by one‑way ANOVA followed by Tukey’s post hoc test, and brain water content was analyzed using Dunnett’s multiple comparisons test

To evaluate the effect of M2‑EVs on the BBB after ICH, mice received intranasal PBS, M0‑EVs, or M2‑EVs immediately after collagenase‑induced hemorrhage. Fluorescence imaging on day 1 confirmed the uptake of M2‑EVs by cerebrovascular endothelial cells (Fig. 3A). Because cerebral edema typically peaks between days 1 and 3 post‑ICH, neurological and motor assessments were performed during this interval. No significant differences in neurological deficit scores or motor performance were detected among groups on day 1. By day 3, however, mice treated with M2‑EVs showed markedly improved neurological scores, prolonged fall latency in the rotarod test, and a more balanced turning preference in the corner test, indicating better motor coordination (Fig. 3B–D). These findings suggest that the therapeutic effect of M2‑EVs was most evident on day 3, which was therefore selected as the main time point for subsequent experiments. To assess BBB integrity, EB extravasation was measured on day 3. Treatment with M2‑EVs significantly reduced EB leakage compared with the ICH and M0‑EVs‑treated groups (Fig. 3E, F). Serial MRI demonstrated pronounced perilesional edema, heterogeneous hematoma density, and apparent hematoma expansion in the ICH and M0‑EVs‑treated groups by day 3, whereas the M2‑EVs‑treated group exhibited more homogeneous hematoma density and a significantly smaller hematoma volume (Fig. 3G, H). Consistently, brain water content measurements confirmed that M2‑EVs alleviated cerebral edema (Fig. 3I). Gait analysis provided additional objective evidence of improved motor function. Relative to the ICH and M0‑EVs‑treated groups, mice treated with M2‑EVs showed significant improvements in several parameters of the limbs contralateral to the hemorrhage (left limbs), including maximum contact area, maximum paw pressure, and the ratio of single‑limb stance time to step cycle (Fig. 3J–P).

### M2‑EVs Alleviate Tight Junction Disruption After ICH by Inhibiting Endothelial Apoptosis

**Fig. 4:**
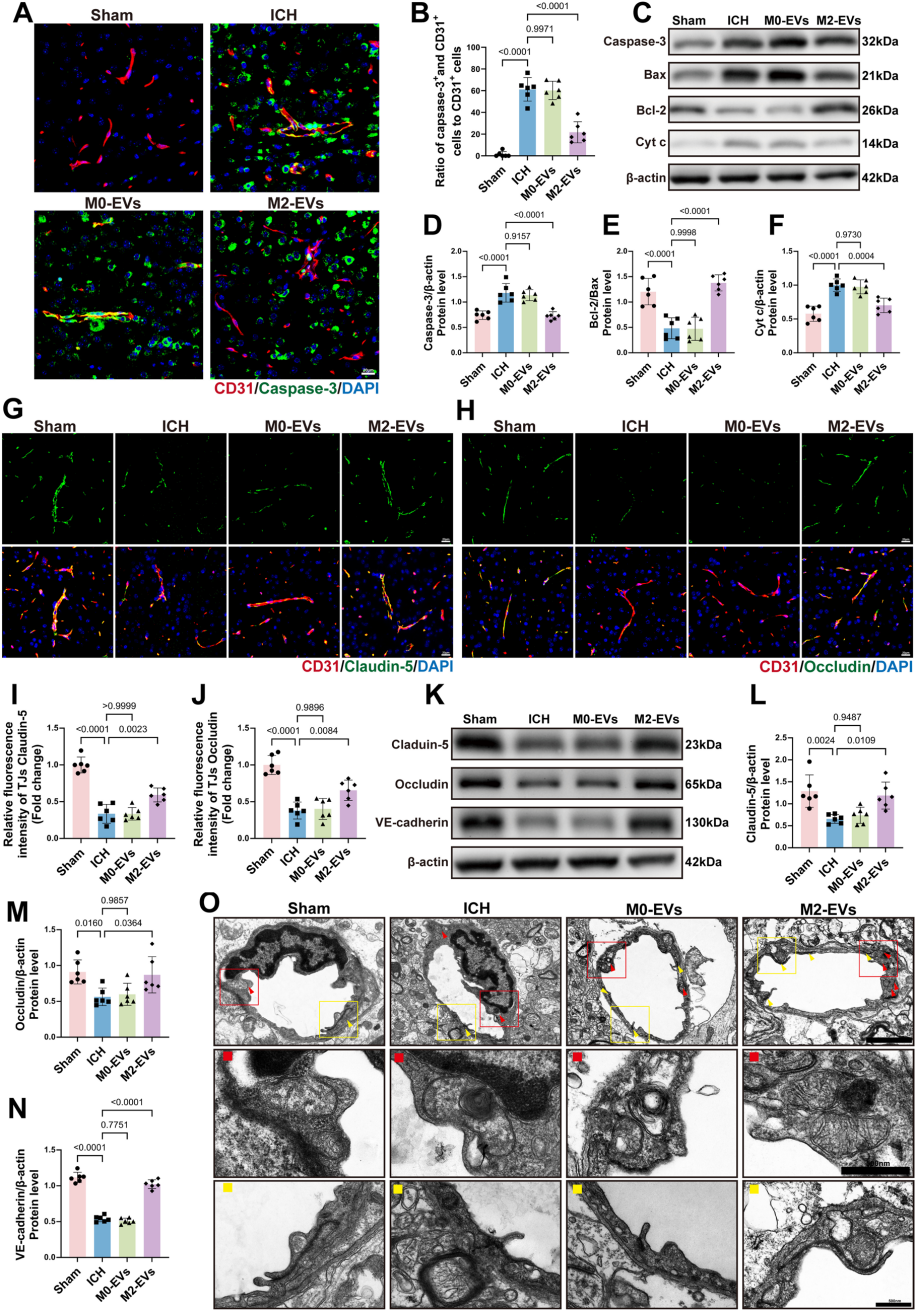
M2‑EVs attenuate BBB injury after ICH in mice by reducing endothelial apoptosis and tight junction disruption.** A **Immunofluorescence staining of endothelial apoptosis, with CD31 (red) and Caspase‑3 (green) co‑labeling. Scale bar: 20 µm. **B **Quantitative analysis of endothelial apoptosis by immunofluorescence (*F*(3, 20) = 72.15, *p* < 0.0001; *n* = 6), showing that M2‑EVs markedly reduce Caspase‑3 expression in cerebrovascular endothelial cells compared with the ICH group. **C **Representative Western blot images of the apoptosis‑related proteins Caspase‑3, Bax, Cyt c, and Bcl‑2. **D**–**F** Quantitative analysis of apoptosis‑related protein expression (Caspase‑3: *F*(3, 20) = 24.61, *p* < 0.0001; Cyt c: *F*(3, 20) = 24.15, *p* < 0.0001; Bcl‑2/Bax: *F*(3, 20) = 28.88, *p* < 0.0001; *n* = 6), indicating that, relative to the ICH group, treatment with M2‑EVs decreases Caspase‑3 and Cyt c levels and increases the Bcl‑2/Bax ratio. **G**,** H** Immunofluorescence staining of endothelial tight junctions, with CD31 (red) co‑labeled with Claudin‑5 (green) or Occludin (green). Scale bars: 20 µm. **I**,** J** Quantitative analysis of tight junction integrity (Claudin‑5: *F*(3, 20) = 52.93, *p* < 0.0001; Occludin: *F*(3, 20) = 28.78, *p* < 0.0001; *n* = 6), indicating that M2‑EVs markedly attenuate Claudin‑5 and Occludin disruption compared with the ICH group. **K** Representative Western blot images of the tight junction proteins Claudin‑5 and Occludin and the adherens junction protein VE‑cadherin. **L**–**N** Quantitative analysis of junctional protein expression (Claudin‑5: *F*(3, 20) = 8.771, *p* = 0.0007; Occludin: *F*(3, 20) = 5.976, *p* = 0.0044; VE‑cadherin: *F*(3, 20) = 198.8, *p* < 0.0001; *n* = 6), showing that treatment with M2‑EVs significantly upregulates Claudin‑5, Occludin, and VE‑cadherin compared with the ICH group. **O** TEM images of endothelial mitochondrial and tight junction ultrastructure in peri‑hematomal regions at 3 days post‑ICH. Overall scale bar: 2.0 µm. The red box (enlarged in the red panel) highlights mitochondrial alterations, and the yellow box (enlarged in the yellow panel) highlights tight junction structures. Detail scale bars: 500 nm. Data are presented as mean ± SD. Multiple‑group comparisons were analyzed by one‑way ANOVA followed by Tukey’s post hoc test

At 3 days after ICH, when M2‑EVs had already been shown to reduce brain edema, vascular leakage and neurological deficits, their effects on endothelial apoptosis and tight junction integrity were further examined. Immunofluorescence analysis revealed that endothelial apoptosis in the peri‑hematomal region was markedly reduced in the M2‑EVs‑treated group compared with the ICH and M0‑EVs‑treated groups (Fig. 4A, B). Consistently, Western blot analysis showed that treatment with M2‑EVs decreased the expression of the apoptosis‑related proteins Caspase‑3 and Cyt c and increased the Bcl‑2/Bax ratio, in agreement with the *in vitro* findings (Fig. 4C–F). We next assessed the status of vascular endothelial tight junctions. Immunofluorescence staining demonstrated that treatment with M2‑EVs significantly upregulated the tight junction proteins Claudin‑5 and Occludin in microvessels (Fig. 4G–J). Western blotting further confirmed elevated levels of Claudin‑5, Occludin, and the adherens junction protein VE‑cadherin in brain tissue from M2‑EVs‑treated mice (Fig. 4K–N). Together, these data indicate that, *in vivo*, M2‑EVs suppress endothelial apoptosis and thereby help preserve tight junction integrity. To directly evaluate endothelial ultrastructure, TEM was performed on peri‑hematomal brain tissue. In the sham group, endothelial cells exhibited intact morphology, with well‑preserved mitochondria showing clear cristae (red arrows) and tightly apposed tight junctions (yellow arrows). By contrast, the ICH and M0‑EVs‑treated groups displayed pronounced mitochondrial damage, including swelling, cristae loss and myelin figure formation, accompanied by disorganized tight junctions. These pathological alterations were markedly attenuated in the M2‑EVs‑treated group (Fig. 4O). Taken together with the Western blot evidence of increased Bcl‑2 and decreased Bax, Cyt c, and Caspase‑3, these findings suggest that M2‑EVs mitigate BBB disruption by inhibiting endothelial apoptosis, likely through a mitochondrial pathway.

### Identification of miR‑27b‑3p as a Key Apoptosis‑Related miRNA Enriched in M2‑EVs

**Fig. 5:**
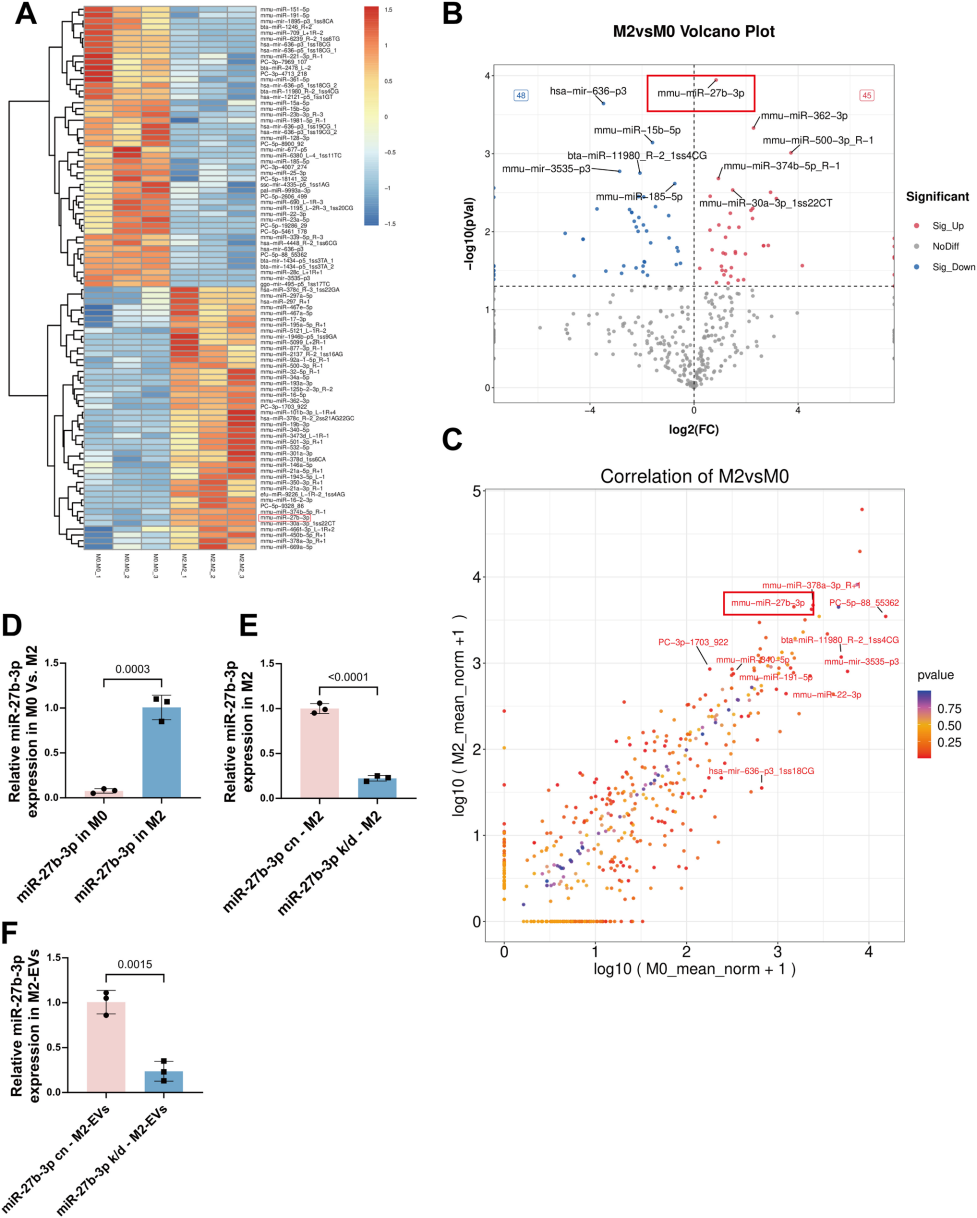
Identification of miR‑27b‑3p enrichment in M2‑EVs and its reduction by lentiviral knockdown in M2 microglia and their EVs. **A **Heatmap of differentially expressed miRNAs between M0‑EVs and M2‑EVs. **B **Volcano plot showing the global distribution of differentially expressed miRNAs, highlighting miR‑27b‑3p as one of the most strongly altered miRNAs. **C **Scatter plot of miRNA expression levels and relative abundance, with miR‑27b‑3p ranking among the most enriched miRNAs. **D **qPCR analysis of miR‑27b‑3p expression in M0 and M2 microglia (*t*(4) = 11.60, *p* = 0.0003; *n* = 3), showing significantly higher miR‑27b‑3p levels in M2‑polarized microglia compared with M0 microglia. **E **qPCR analysis of miR‑27b‑3p expression in M2 microglia after lentiviral knockdown (*t*(4) = 21.18, *p* < 0.0001; *n* = 3), demonstrating a significant reduction in miR‑27b‑3p levels. **F **qPCR analysis of miR‑27b‑3p in M2‑EVs derived from the same cells (*t*(4) = 7.809, *p* = 0.0015; *n* = 3), showing that miR‑27b‑3p content is markedly reduced in EVs from the knockdown group. All quantitative data are presented as mean ± SD. An unpaired *t*‑test was used for statistical analysis

Previous studies have shown that miRNAs enriched in EVs play pivotal roles in intercellular communication and gene regulation, exerting important modulatory effects on multiple cell types after brain injury [35]. Therefore, we compared the miRNA expression profiles of M0‑EVs and M2‑EVs. Using a threshold of *p* < 0.05, 93 differentially expressed miRNAs were identified, including 45 upregulated and 48 downregulated species (Fig. 5A). Among these, miR‑27b‑3p emerged as one of the most prominently altered miRNAs in the volcano plot (Fig. 5B), and its differential abundance between the two groups was further supported by scatter plot analysis (Fig. 5C). Given the reported role of miR‑27b‑3p in regulating apoptosis [51], we hypothesized that it might represent a key functional component of M2‑EVs. To explore this possibility, we first measured miR‑27b‑3p levels in M0 and M2 microglia by qPCR and confirmed a significant upregulation in M2‑polarized cells (Fig. 5D). We then knocked down miR‑27b‑3p in M2 microglia using a lentiviral vector, with a scrambled sequence as the control. qPCR analysis demonstrated a marked reduction of miR‑27b‑3p expression in the transduced M2 microglia and in the EVs subsequently released from these cells, confirming efficient knockdown (Fig. 5E, F).

### miR‑27b‑3p Enriched in M2‑EVs Alleviates Endothelial Apoptosis and Tight Junction Disruption *In Vitro*

**Fig. 6:**
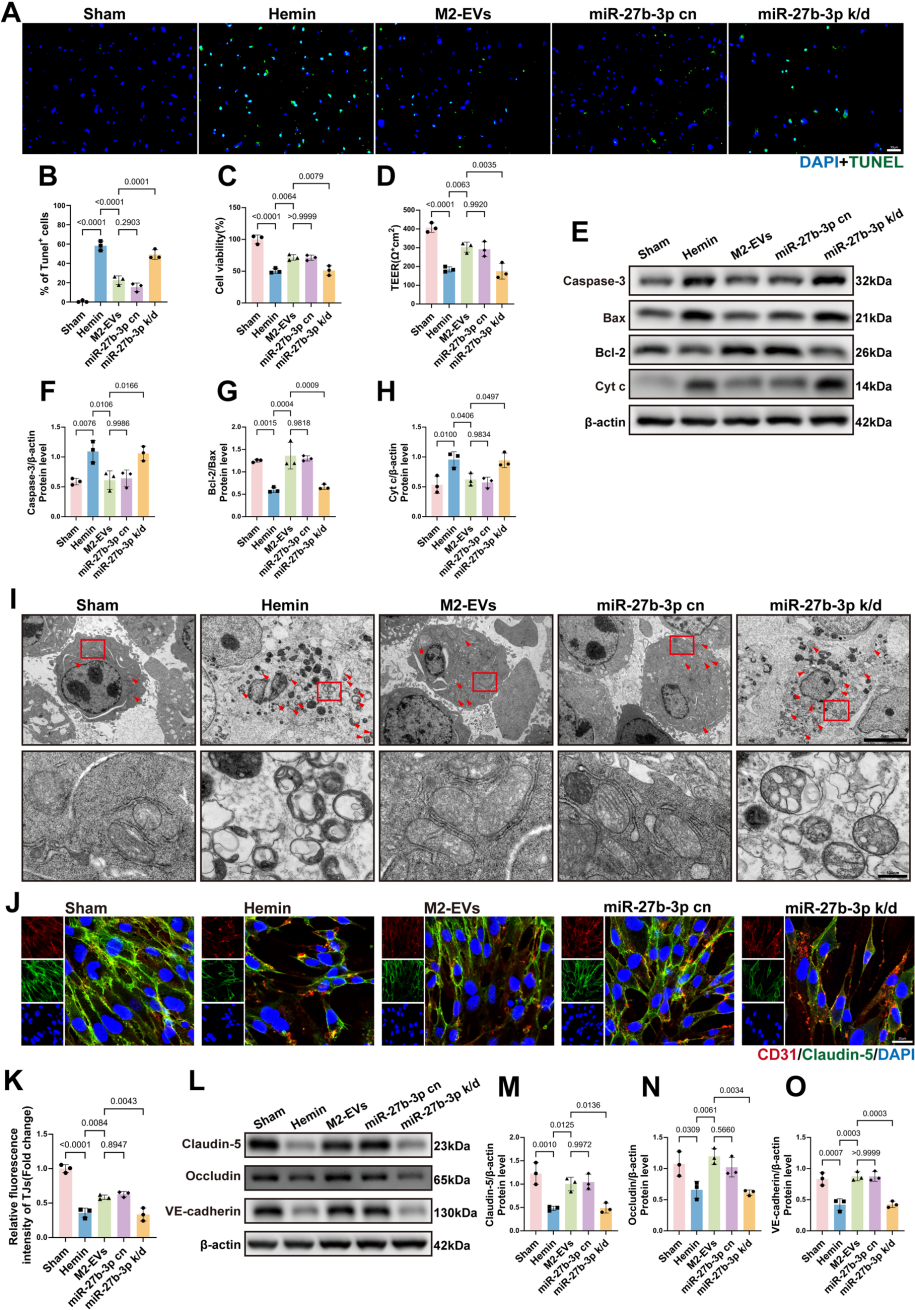
miR‑27b‑3p enriched in M2‑EVs attenuates endothelial apoptosis and preserves tight junction integrity *in vitro*. **A **Detection of apoptosis by TUNEL immunofluorescence staining (green). **B **Quantification of TUNEL‑positive cells (*F*(4, 10) = 96.26, *p* < 0.0001; *n* = 3), indicating that miR‑27b‑3p knockdown reverses the anti‑apoptotic effect of M2‑EVs and markedly increases apoptosis. **C **Quantitative analysis of cell viability by CCK‑8 assay (*F*(4, 10) = 39.73, *p* < 0.0001; *n* = 3), showing that miR‑27b‑3p knockdown abolishes the protective effect of M2‑EVs on endothelial survival. **D **Quantitative analysis of TEER (*F*(4, 10) = 28.48,* p* < 0.0001; *n* = 3), demonstrating a marked decrease in TEER after miR‑27b‑3p knockdown. **E **Representative Western blot images of the apoptosis‑related proteins Caspase‑3, Bax, Cyt c, and Bcl‑2 in endothelial cells. **F**–**H** Quantitative analysis of apoptosis‑related protein expression (Caspase‑3: *F*(4, 10) = 10.33, *p* = 0.0014; Cyt c: *F*(4, 10) = 9.051, *p* = 0.0023; Bcl‑2/Bax: *F*(4, 10) = 20.89, *p* < 0.0001; *n* = 3). Compared with M2‑EVs treatment, miR‑27b‑3p knockdown increases Caspase‑3 and Cyt c levels and decreases the Bcl‑2/Bax ratio. **I **TEM images showing ultrastructural changes associated with mitochondrial‑mediated endothelial apoptosis. Scale bar: 5.0 µm (inset scale bar: 500 nm). **J **Immunofluorescence staining of endothelial tight junctions, with CD31 (red) and Claudin‑5 (green). **K **Quantitative analysis of Claudin‑5 fluorescence intensity (*F*(4, 10) = 55.62, *p* < 0.0001; *n* = 3), showing that Claudin‑5 disruption is aggravated and signal intensity is significantly reduced when miR‑27b‑3p is knocked down in M2‑EVs. **L **Representative Western blot images of the tight junction proteins Claudin‑5 and Occludin and the adherens junction protein VE‑cadherin. **M**–**O ** Quantitative analysis of junctional protein expression (Claudin‑5: *F*(4, 10) = 15.17, *p* = 0.0003; Occludin: *F*(4, 10) = 10.27,* p* = 0.0014; VE‑cadherin: *F*(4, 10) = 26.61, *p* < 0.0001; *n* = 3), showing that the knockdown of miR‑27b‑3p in M2‑EVs reduces Claudin‑5, Occludin, and VE‑cadherin expression. Data are presented as mean ± SD. Multiple‑group comparisons were performed using one‑way ANOVA followed by Tukey’s post hoc test

In this *in vitro* model, M2‑EVs and the miR‑27b‑3p cn group exerted comparable protective effects on hemin‑injured endothelial cells. Compared with the hemin‑injured group, both treatments significantly reduced the proportion of TUNEL‑positive cells (Fig. 6A, B) and markedly improved cell viability and TEER (Fig. 6C, D). Western blot analysis further showed that M2‑EVs and the miR‑27b‑3p cn group decreased the expression of the pro‑apoptotic proteins Caspase‑3 and Cyt c while increasing the Bcl‑2/Bax ratio, indicating attenuation of apoptotic signaling (Fig. 6E–H). TEM revealed that endothelial cells and their mitochondria in these two treatment groups maintained relatively intact morphology, comparable to that observed in the normal control group (Fig. 6I). Moreover, immunofluorescence staining demonstrated that M2‑EVs and the miR‑27b‑3p cn group mitigated disruption of the tight junction protein Claudin‑5 (Fig. 6J, K). Western blot analysis further confirmed that, relative to the hemin group, both treatments increased the expression of the tight junction proteins Claudin‑5 and Occludin, as well as the adherens junction protein VE‑cadherin (Fig. 6L–O). By contrast, knockdown of miR‑27b‑3p abrogated these protective effects and reversed the improvements in anti‑apoptotic signaling and junctional integrity.

### miR‑27b‑3p Enriched in M2‑EVs Alleviates Cerebral Edema, Hematoma Expansion, and Neurological Deficits at 3 Days After ICH in Mice

**Fig. 7:**
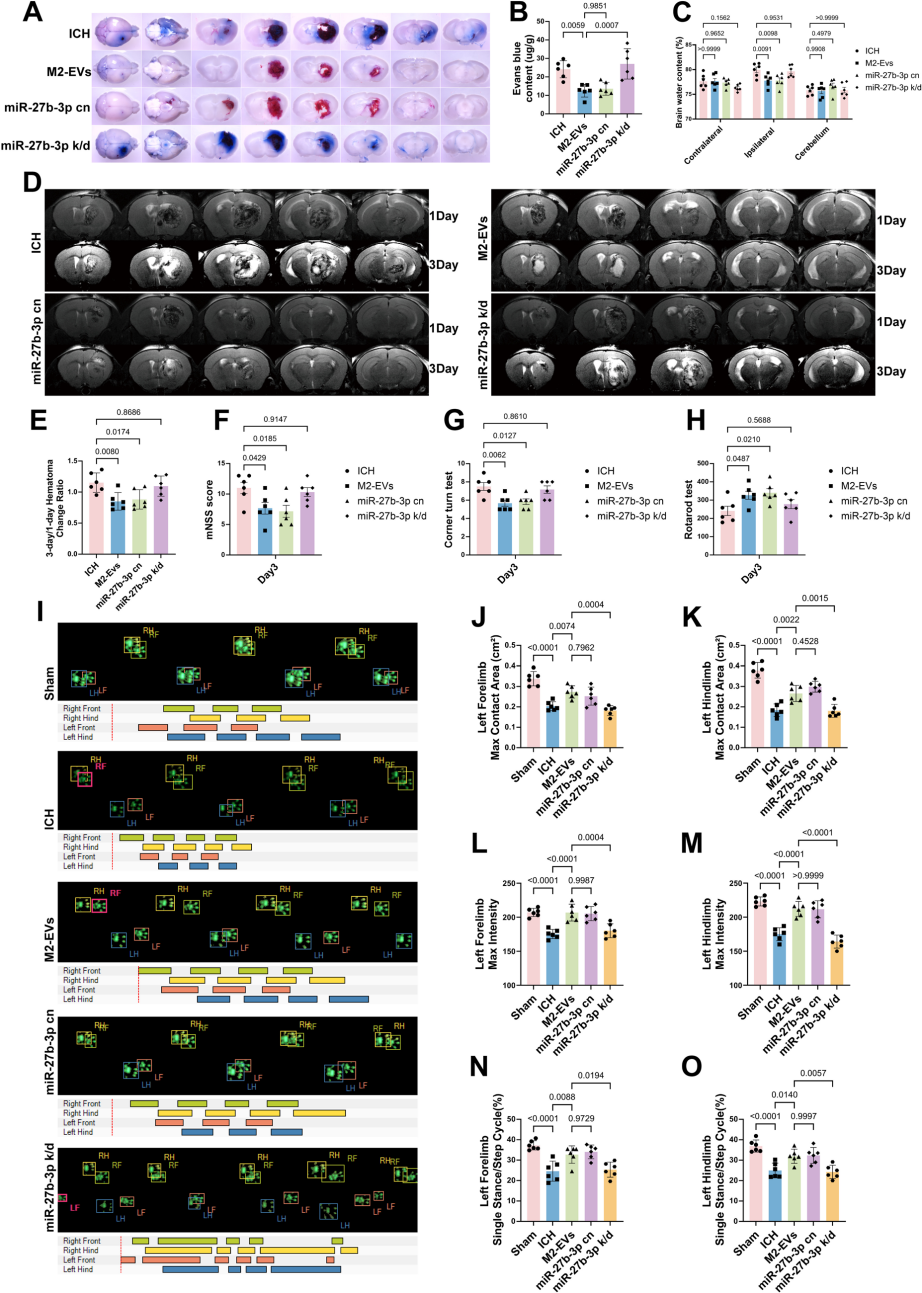
miR‑27b‑3p enriched in M2‑EVs ameliorates cerebral edema, limits hematoma expansion, and improves neurological function at 3 days after ICH in mice. **A **Representative macroscopic images of brain slices from the EB extravasation assay. **B **Quantitative analysis of EB content in brain tissue (*F*(3, 20) = 3.849, *p* = 0.0252; *n* = 6), showing that knockdown of miR‑27b‑3p in M2‑EVs significantly increases vascular leakage compared with the M2‑EVs‑treated group. **C **Quantitative analysis of brain water content in the ipsilateral hemisphere on day 3 post‑ICH (*q* = 2.161, *p* = 0.0098; *n* = 6). Brain edema is significantly aggravated in the miR‑27b‑3p knockdown group, reaching a level comparable to that of the ICH model group. **D **Representative MRI scans on days 1 and 3 post‑ICH. **E **Quantification of the hematoma volume change rate from day 1 to day 3 (*F*(3, 20) = 5.774, *p* = 0.0052; *n* = 6), indicating that miR‑27b‑3p knockdown significantly reverses the suppressive effect of M2‑EVs on hematoma expansion. **F**–**H ** Neurological and behavioral assessments on day 3 post‑ICH, including the mNSS (*F*(3, 20) = 4.476, *p* = 0.0147), rotarod test (*F*(3, 20) = 3.690, *p* = 0.0290), and corner test (*F*(3, 20) = 6.246, *p* = 0.0036; *n* = 6). **I **Schematic of the gait analysis setup. LF, left forelimb; LH, left hindlimb; RF, right forelimb; RH, right hindlimb. **J**–**O** Quantitative gait analysis of the contralateral (left) limbs: maximum contact area (LF: *F*(4, 25) = 21.97, *p* < 0.0001; LH: *F*(4, 25) = 37.06, *p* < 0.0001; **J**, **K**), maximum paw pressure (LF: *F*(4, 25) = 16.73, *p* < 0.0001; LH: *F*(4, 25) = 39.83, *p* < 0.0001; **L**, **M**), and the ratio of single‑limb stance time to step cycle duration (LF: *F*(4, 25) = 12.86, *p* < 0.0001; LH: *F*(4, 25) = 14.59, *p* < 0.0001; **N**, **O**; *n* = 6). The improvements in all gait parameters mediated by M2‑EVs are significantly reversed by miR‑27b‑3p knockdown. Data are presented as mean ± SD. Multiple‑group comparisons were analyzed by one‑way ANOVA followed by Tukey’s post hoc test, and brain water content was analyzed using Dunnett’s multiple comparisons test

To further verify the protective effects of miR‑27b‑3p on the BBB and neurological function in an *in vivo* model, we performed a series of neurological and behavioral assessments. Knockdown of miR‑27b‑3p in M2‑EVs abolished the therapeutic benefits of M2‑EVs, and the resulting phenotype closely resembled that of the ICH model group. Both the ICH group and the miR‑27b‑3p knockdown group showed marked EB extravasation (Fig. 7A, B), significantly increased brain water content (Fig. 7C), and varying degrees of hematoma expansion (Fig. 7D, E). Functional evaluation on day 3 post‑ICH revealed higher neurological deficit scores, shorter fall latency in the rotarod test, and poorer turning performance in the corner test in these two groups (Fig. 7F–H). Consistently, gait analysis demonstrated significant reductions in several parameters of the limbs contralateral to the hemorrhagic hemisphere (left forelimb and hindlimb)—including maximum contact area, maximum paw pressure, and the ratio of single‑limb stance time to gait cycle duration—compared with the sham group (Fig. 7I–O). In contrast, treatment with M2‑EVs significantly improved all of the above parameters, and the miR‑27b‑3p cn group exhibited therapeutic effects comparable to those observed in the M2‑EVs‑treated group.

### miR‑27b‑3p Enriched in M2‑EVs Preserves Mitochondrial Homeostasis, Attenuates Mitochondria‑Mediated Endothelial Apoptosis, and Mitigates Tight Junction Disruption After ICH in Mice

**Fig. 8:**
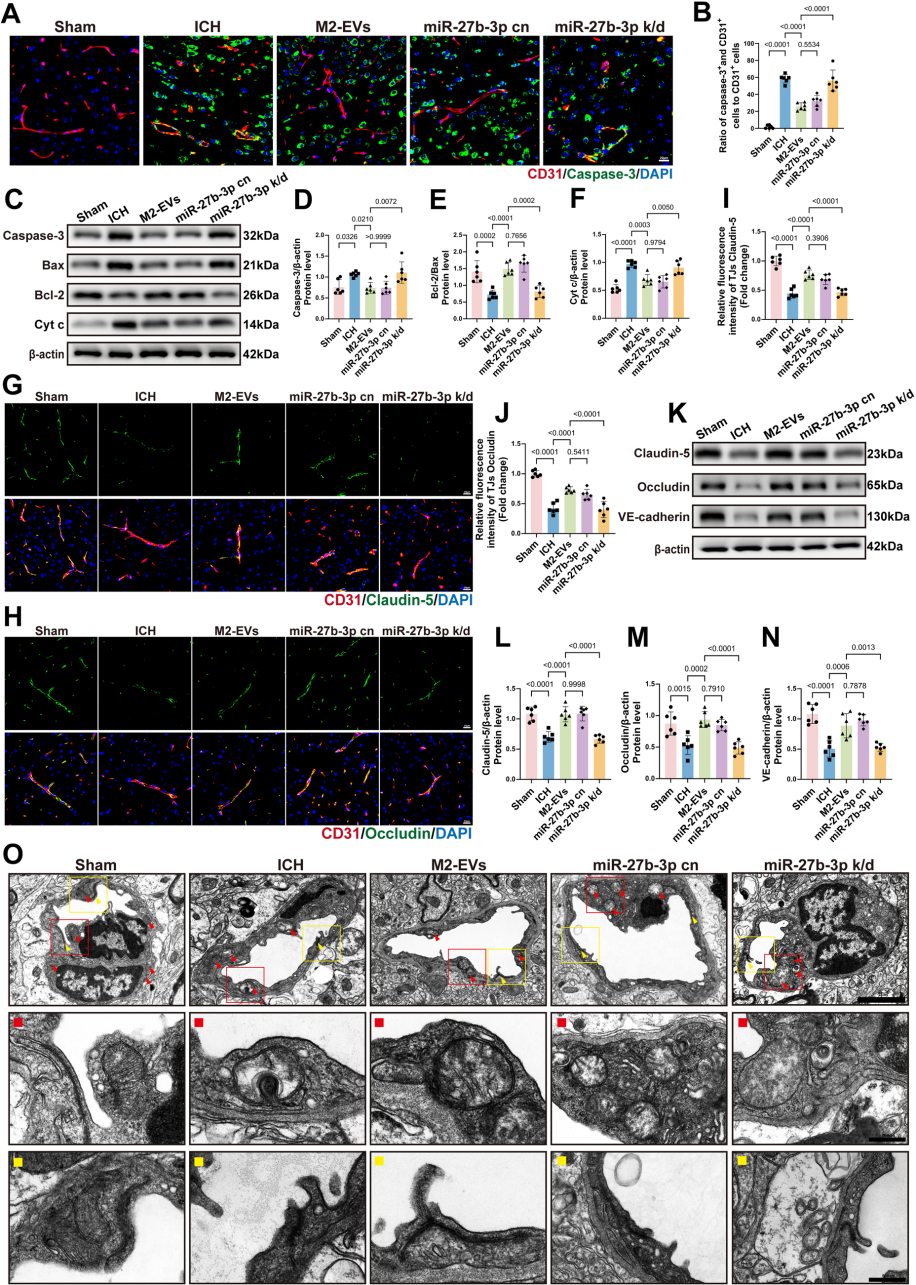
miR‑27b‑3p in M2‑EVs protects against endothelial apoptosis and tight junction disruption after ICH in mice. **A **Immunofluorescence staining of endothelial apoptosis in brain tissue, with CD31 (red) and Caspase‑3 (green) co‑labeling. Scale bar: 20 μm. **B **Quantitative analysis of endothelial apoptosis by immunofluorescence (*F*(4, 25) = 66.64, *p* < 0.0001; *n* = 6), showing significantly higher apoptosis in the miR‑27b‑3p knockdown group than in the M2‑EVs group. **C **Representative Western blot images of the apoptosis‑related proteins Caspase‑3, Bcl‑2, Bax, and Cyt c. **D**–**F ** Quantitative analysis of apoptosis‑related protein expression by Western blot (Caspase‑3: *F*(4, 25) = 7.335,* p* = 0.0005; Cyt c: *F*(4, 25) = 21.35, *p* < 0.0001; Bcl‑2/Bax: *F*(4, 25) = 20.17, *p* < 0.0001; *n* = 6). Compared with the M2‑EVs group, the miR‑27b‑3p knockdown group shows higher Caspase‑3 and Cyt c levels and a lower Bcl‑2/Bax ratio. **G**,** H** Immunofluorescence staining of endothelial tight junctions in brain tissue, with CD31 (red) co‑labeled with Claudin‑5 (green) or Occludin (green). Scale bars: 20 μm. **I**,** J** Quantitative analysis of tight junction integrity by immunofluorescence (Claudin‑5: *F*(4, 25) = 51.27, *p* < 0.0001; Occludin: *F*(4, 25) = 45.67, *p* < 0.0001; *n* = 6), indicating that Claudin‑5 and Occludin fluorescence intensity is significantly reduced in the miR‑27b‑3p knockdown group. **K **Representative Western blot images of the tight junction proteins Claudin‑5 and Occludin and the adherens junction protein VE‑cadherin. **L**–**N** Quantitative analysis of junctional protein expression by Western blot (Claudin‑5: *F*(4, 25) = 25.51, *p* < 0.0001; Occludin: *F*(4, 25) = 14.64, *p* < 0.0001; VE‑cadherin: *F*(4, 25) = 21.63, *p* < 0.0001; *n* = 6). Relative to the M2‑EVs group, the miR‑27b‑3p knockdown group exhibits lower expression of Claudin‑5, Occludin, and VE‑cadherin. **O **TEM images of mitochondrial and tight junction ultrastructure in endothelial cells around the hematoma at 3 days post‑ICH. Overall scale bar: 2.0 μm. Red arrows indicate mitochondria and yellow arrows indicate tight junctions; the red box (enlarged in the red panel) highlights mitochondrial morphology, and the yellow box (enlarged in the yellow panel) highlights tight junction structures. Detail scale bars: 500 nm. Data are presented as mean ± SD. Multiple‑group comparisons were performed using one‑way ANOVA followed by Tukey’s post hoc test

To elucidate how miR‑27b‑3p enriched in M2‑EVs protects the vascular endothelium after ICH, we performed mechanistic analyses. Immunofluorescence staining showed that, compared with the ICH group, both the M2‑EVs‑treated group and the miR‑27b‑3p cn group exhibited markedly reduced Caspase‑3 expression in endothelial cells surrounding the hematoma (Fig. 8A, B). Consistently, Western blot analysis demonstrated that these two treatments decreased Caspase‑3 and Cyt c levels while increasing the Bcl‑2/Bax ratio, indicating inhibition of the mitochondrial apoptotic pathway (Fig. 8C–F). We next evaluated endothelial junctional integrity. Immunofluorescence revealed that treatment with either miR‑27b‑3p cn or M2‑EVs attenuated disruption of the tight junction proteins Claudin‑5 and Occludin (Fig. 8G–J). Western blot analysis further confirmed that, in these groups, expression of Claudin‑5, Occludin, and the adherens junction protein VE‑cadherin was upregulated, whereas this effect was lost in the miR‑27b‑3p knockdown groups (Fig. 8K–N). TEM provided additional structural support for these findings (Fig. 8O). Endothelial cells in the ICH and miR‑27b‑3p knockdown groups displayed marked mitochondrial injury and junctional disorganization, including mitochondrial swelling, loss or blurring of cristae, myelin figure formation (red arrows), and disrupted tight junctions (yellow arrows). By contrast, mitochondrial morphology and junctional structures were largely preserved in the miR‑27b‑3p cn and M2‑EVs‑treated groups, where these pathological changes were substantially alleviated.

### miR‑27b‑3p Attenuates Endothelial Apoptosis in the ICH Model by Targeting the MKK4/JNK Pathway

**Fig. 9:**
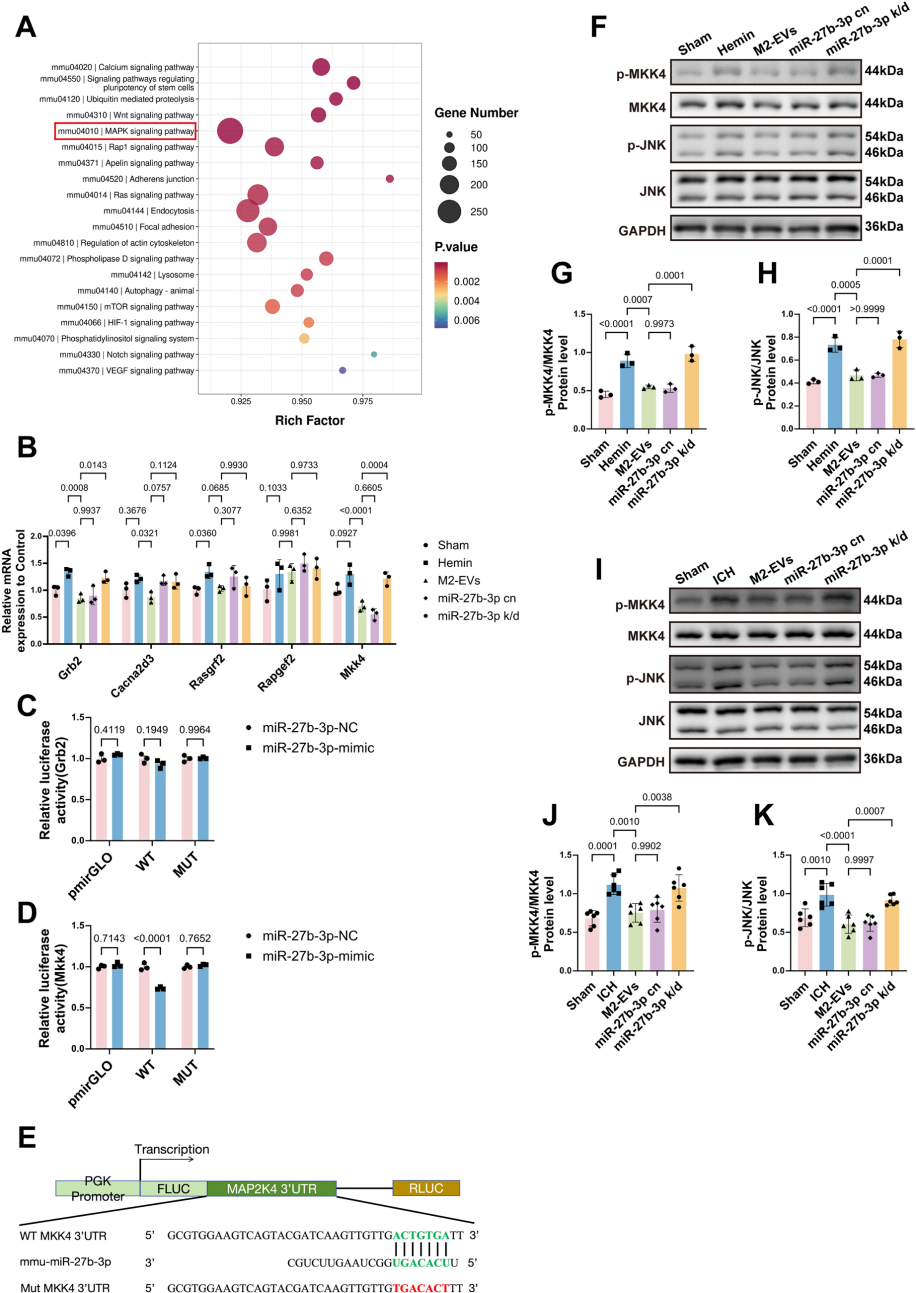
miR‑27b‑3p targets MKK4 and reduces the phosphorylation of MKK4 and its downstream effector JNK. **A **Bubble plot of KEGG pathway enrichment analysis for predicted target genes of miR‑27b‑3p. **B **qPCR analysis of the top five predicted miR‑27b‑3p target genes within the MAPK signaling pathway (*F*(16, 50) = 4.615, *p* < 0.0001; *n* = 3). Compared with the hemin group, treatment with M2‑EVs or miR‑27b‑3p significantly decreased Grb2 and Mkk4 expression, whereas this effect was reversed after miR‑27b‑3p knockdown. **C**–**E ** Dual‑luciferase reporter assays assessing the interaction of miR‑27b‑3p with the Grb2 and Mkk4 3′‑UTRs (Grb2: *F*(2, 12) = 3.096, *p* = 0.0824; Mkk4: *F*(2, 12) = 53.50,* p* < 0.0001; *n* = 3). The miR‑27b‑3p mimic significantly suppressed luciferase activity driven by the Mkk4 3′‑UTR but had no significant effect on the Grb2 3′‑UTR reporter. **F**,** I** Representative Western blot images of MKK4, p‑MKK4, JNK, and p‑JNK *in vitro* (**F**) and *in vivo* (**I**).** G**,** H**,** J**,** K** Quantitative analysis of pathway protein expression *in vitro* (p‑MKK4: *F*(4, 10) = 37.70, *p* < 0.0001; p‑JNK: *F*(4, 10) = 35.87,* p* < 0.0001; G, H; *n* = 3) and *in vivo* (p‑MKK4: *F*(4, 25) = 12.37, *p* < 0.0001; p‑JNK: *F*(4, 25) = 14.54, *p* < 0.0001; J, K; *n* = 6). Relative to the model group, treatment with M2‑EVs reduced p‑MKK4 and p‑JNK levels, whereas knockdown of miR‑27b‑3p in M2‑EVs reversed these effects in both i*n vitro* and *in vivo*. Data are presented as mean ± SD. For multi‑group comparisons, one‑way ANOVA with Tukey’s post hoc test was applied; dual‑luciferase reporter data were analyzed by two‑way ANOVA with Sidak’s multiple comparisons test

To identify the key target through which miR‑27b‑3p regulates apoptosis in cerebrovascular endothelial cells, we first predicted potential target genes using the miRanda and TargetScan databases. Bioinformatic analysis indicated that many of these candidates were enriched in the MAPK signaling pathway (Fig. 9A), which plays a central role in cell growth, differentiation, and apoptosis. We therefore focused on MAPK‑related targets of miR‑27b‑3p. The top five genes ranked by TargetScan score were Grb2, Cacna2d3, Rasgrf2, Rapgef2, and Mkk4. Subsequent qPCR analysis showed altered expression of Grb2 and Mkk4 in both the M2‑EVs‑treated group and the miR‑27b‑3p cn group (Fig. 9B). To determine the direct target, we performed dual‑luciferase reporter assays. miR‑27b‑3p mimic significantly suppressed luciferase activity in mouse primary brain microvascular endothelial cells transfected with the Mkk4 3′‑UTR reporter but had no significant effect in cells transfected with the Grb2 3′‑UTR reporter (Fig. 9C–E). We then examined the expression of phosphorylated MKK4 and JNK by Western blot in both *in vitro* (Fig. 9F–H) and *in vivo* samples (Fig. 9I–K). Both treatment with M2‑EVs and administration of miR‑27b‑3p cn significantly reduced p‑MKK4 and p‑JNK levels, whereas this effect was reversed in the miR‑27b‑3p k/d group. Taken together, these findings suggest that miR‑27b‑3p directly targets MKK4, inhibits its phosphorylation and thereby limits downstream JNK activation. This modulation of the MKK4/JNK pathway is likely to increase the Bcl‑2/Bax ratio, stabilize mitochondrial homeostasis, reduce Cyt c release and subsequent Caspase‑3 activation, and ultimately suppress intrinsic endothelial apoptosis. Consequently, disruption of tight and adherens junctions is attenuated, BBB integrity is better preserved, and cerebral edema, hematoma expansion, and neurological deficits are ameliorated at 3 days post‑ICH.

## Discussion

In this study, we first showed in an *in vitro* ICH model that M2‑EVs markedly inhibited endothelial apoptosis and alleviated disruption of tight junction structures. Consistent with these findings, *in vivo* experiments in ICH mice demonstrated that M2‑EVs reduced cerebrovascular endothelial cell apoptosis and tight junction damage, thereby preserving BBB integrity, attenuating cerebral edema and improving neurological deficits. miRNA sequencing combined with functional validation further revealed that miR‑27b‑3p, which is enriched in M2‑EVs, targets and suppresses MKK4, thereby modulating the downstream MKK4/JNK signaling pathway. Regulation of this pathway inhibited the mitochondrial apoptotic cascade in endothelial cells, as evidenced by increased Bcl‑2 expression, decreased Bax, Cyt c and Caspase‑3 expression, and improved mitochondrial and tight junction ultrastructure on TEM. In contrast, knockdown of miR‑27b‑3p in M2‑EVs abolished these protective effects. Taken together, these results suggest that miR‑27b‑3p carried by M2‑EVs mitigates mitochondria‑dependent endothelial apoptosis via the MKK4/JNK pathway, thereby maintaining BBB function and ultimately reducing post‑ICH cerebral edema, hematoma expansion and neurological impairment.

As immune cells that are highly sensitive to microenvironmental changes within the CNS, microglia play a dual regulatory role in brain injury and repair [29–31]. Growing evidence indicates that EVs secreted by microglia in different activation states exert distinct regulatory effects on various brain cell types, with their cargo miRNAs being particularly important for mediating intercellular communication and gene expression [37, 52]. With respect to endothelial cells, different miRNAs have been shown to exert opposing effects through specific signaling pathways. For example, Xie et al. reported that miR‑424‑5p derived from microglia subjected to OGD promotes endothelial apoptosis and aggravates BBB disruption by inhibiting the FGF2/STAT3 pathway [43]. Jiang et al. reported that EVs derived from M1‑polarized microglia increased endothelial apoptosis, reduced TEER and the expression of BBB‑associated proteins, and consequently enhanced BBB permeability, whereas vesicles from resting microglia had no such effect [44]. In contrast, Zhao et al. demonstrated that miR‑124‑3p, enriched in EVs from IL‑4-induced M2 microglia, enhances endothelial autophagy and suppresses apoptosis via activation of the mTOR pathway [42]. Furthermore, Pan et al. showed that miR‑23a‑5p in M2‑EVs alleviates post‑ischemic BBB injury by downregulating TNF‑α‑mediated MMP3 and NF‑κB signaling while upregulating the tight junction proteins ZO‑1 and Claudin‑5 [46]. Collectively, these studies highlight endothelial apoptosis as a key mechanism underlying BBB dysfunction, closely associated with poor neurological outcomes. Consequently, directly or indirectly preserving BBB integrity has emerged as a critical therapeutic strategy for promoting neurological recovery [12, 13, 15, 16, 53].

Notably, previous studies have shown that downregulation of miR‑27b‑3p in endothelial cells under stress conditions promotes intrinsic apoptosis [51]. Under physiological conditions, this miRNA targets FOXO1 and inhibits the Akt/FOXO1 signaling pathway, thereby attenuating mitochondrial oxidative stress, inflammatory responses, and Caspase‑3‑dependent apoptosis. In addition, Li et al. reported that miR‑27b‑3p alleviates endothelial apoptosis by suppressing Apaf‑1 [54]. In line with these findings, our comparative miRNA sequencing of M0‑EVs and M2‑EVs revealed that miR‑27b‑3p is significantly enriched in M2‑EVs. Its upregulated expression strongly correlates with reduced endothelial apoptosis, amelioration of tight junction damage, and improved neurological recovery after ICH. Taken together, these data highlight the substantial therapeutic potential of miR‑27b‑3p in regulating cell apoptosis and related cellular processes. Based on the pronounced changes we observed in apoptosis‑related proteins, including Bcl‑2, Bax, Cyt c, and Caspase‑3, we focused on the MAPK signaling pathway rather than the calcium, Wnt, or Apelin pathways, despite some of these pathways showing higher statistical significance in enrichment analysis. This choice was primarily driven by the central role of the MAPK family in cellular stress responses and regulation of the mitochondrial apoptotic pathway. Within this pathway, JNK acts as a key effector that can attenuate the anti‑apoptotic function of Bcl‑2 through phosphorylation and positively regulate the expression of the pro‑apoptotic protein Bax. Bcl‑2 is an anti‑apoptotic protein located on the mitochondrial membrane, and an imbalance in the Bcl‑2/Bax ratio increases mitochondrial membrane permeability, thereby promoting Cyt c release and ultimately activating the apoptotic cascade [19–21, 55–58].

Through *in vitro* qPCR analysis, we found that treatment with M2‑EVs significantly downregulated Mkk4 and Grb2 expression, whereas this effect was lost after miR‑27b‑3p knockdown. Dual‑luciferase reporter assays further demonstrated that the miR‑27b‑3p mimic specifically targets Mkk4, with no significant regulatory effect on Grb2, thereby identifying Mkk4 as a key target gene of miR‑27b‑3p. Previous studies have reported that MKK4 promotes apoptosis by activating downstream JNK [59, 60], and work by Yuan et al. confirmed that inhibition of the ASK1/MKK4/JNK pathway reduces apoptosis following cerebral ischemia–reperfusion [61]. In our study, miR‑27b‑3p enriched in M2‑EVs suppressed Mkk4, leading to reduced MKK4 phosphorylation and diminished activation of downstream p‑JNK. Consequently, the Bcl‑2/Bax ratio increased, Cyt c release decreased, and expression of the executioner Caspase‑3 was reduced. This mechanism effectively attenuated endothelial apoptosis and tight junction disruption in both *in vitro* and *in vivo* ICH models, helping to preserve BBB integrity and ultimately reducing post‑ICH cerebral edema and hematoma expansion, while improving neurological outcomes.

Building on previous studies showing that miR‑27b‑3p supports endothelial cell survival by interfering with FOXO1 signaling, our results further demonstrate that miR‑27b‑3p exerts protective effects by inhibiting the pro‑apoptotic MKK4/JNK axis, highlighting its multi‑target and bidirectional regulatory properties. Furthermore, unlike the Apaf-1-focused mechanism, our study suggests that miR-27b-3p acts at a more upstream level by stabilizing the mitochondrial outer membrane and inhibiting Cyt c release, thereby blocking apoptosis initiation. This mechanism may provide more timely and broad-ranging cytoprotective effects. Additionally, given the close association of the MKK4/JNK pathway with inflammatory and stress signaling, miR-27b-3p may play a pivotal role in integrating stress responses and regulating cell survival, also pointing toward future directions for exploring the role of this pathway in inflammation modulation.

Although this study has preliminarily elucidated the molecular mechanisms by which M2‑EVs and their cargo miR‑27b‑3p protect endothelial cells in both *in vitro* and *in vivo* models of ICH, several limitations should be acknowledged. First, apoptosis is regulated by multiple, interconnected signaling pathways, whereas we focused solely on the MKK4/JNK pathway; other potential mechanisms remain to be explored. Second, although dual‑luciferase reporter assays confirmed that Mkk4 is a direct target of miR‑27b‑3p, we have not yet performed rescue experiments in which Mkk4 is overexpressed in endothelial cells. Third, the BBB is a complex structure composed of endothelial cells, the basement membrane, pericytes, and astrocytic end‑feet [9, 62]. In this study, we primarily emphasized preservation of barrier function through the inhibition of endothelial apoptosis and did not systematically evaluate the effects of M2‑EVs on other cellular components of the BBB, nor did we further examine their impact on neurons and other glial cells that are closely associated with BBB function. Finally, our *in vivo* experiments mainly assessed short‑term outcomes within 3 days after ICH and did not investigate the long‑term effects of M2‑EVs on hematoma clearance, tissue repair, or sustained neurological recovery.

In light of the main findings and limitations of this study, our future work will proceed along several directions. First, it will be important to perform rescue experiments, such as overexpressing MKK4 in both *in vitro* and *in vivo* models, to more rigorously validate the causal relationship within the miR‑27b‑3p/MKK4/JNK signaling axis. Second, we plan to further investigate how M2‑EVs and miR‑27b‑3p regulate other cellular components of the BBB after ICH, including pericytes, astrocytes, and additional endothelial subpopulations, as well as their broader effects on neurons and glial cells. Third, given the central role of the MAPK pathway in controlling cell growth, proliferation, differentiation, apoptosis, and stress responses, future studies will aim to identify additional MAPK‑related and cross‑talk targets of miR‑27b‑3p and to systematically map its regulatory network across different brain cell types in the post‑ICH microenvironment. This will be critical for fully elucidating the neuroprotective mechanisms of miR‑27b‑3p. Finally, it will be essential to evaluate the long‑term impact of M2‑EVs and miR‑27b‑3p on functional recovery after ICH, including their effects on hematoma resolution, tissue remodeling, and sustained neurological outcomes.

In addition, extracellular vesicles encompass heterogeneous subtypes, including exosomes, microvesicles, and apoptotic bodies [33, 34, 52, 63]. In future studies, it will be important to further delineate the specific roles of different extracellular vesicle subtypes in the CNS. To date, most work has focused on exosomes, whereas the functions of apoptotic bodies have only recently begun to attract attention. Accumulating evidence indicates that apoptotic bodies can exert significant effects in immune regulation, vascular protection, tissue regeneration, and the reduction of cell death. In addition, when used as drug carriers, apoptotic bodies may enhance drug stability, cellular uptake, and targeting efficiency. Together, these findings suggest that systematically exploring the actions of apoptotic bodies within the CNS will be valuable for broadening and deepening EV‑based therapeutic strategies [64–66].

At the level of clinical translation, the development of engineered M2‑EVs is particularly important. For example, modifying their surface proteins may enhance targeting specificity toward cerebrovascular endothelial cells, and designing biomimetic nanocarriers loaded with miR‑27b‑3p may improve their stability and delivery efficiency *in vivo*. In addition, it will be necessary to systematically evaluate the optimal therapeutic time window and long‑term safety of M2‑EVs, as well as their sustained effects on neuroregeneration and functional recovery. It should also be noted that microglia are highly sensitive to environmental changes, and the biological activity of microglia‑derived EVs is readily influenced by culture conditions and experimental interventions. Therefore, further optimization of stimulation parameters, EV preservation methods, and administration strategies is required. Collectively, these efforts will help drive therapeutic strategies based on microglia‑derived EVs and their active components from basic research toward clinical application, providing a novel and precisely regulated treatment option for patients with ICH.

## Conclusion

This study demonstrates that miR‑27b‑3p, enriched in M2‑EVs, targets and suppresses MKK4, thereby modulating the downstream MKK4/JNK signaling pathway. This regulation increases the Bcl‑2/Bax ratio, helps maintain mitochondrial homeostasis, and reduces the release of Cyt c into the cytosol, which in turn lowers the expression of the downstream effector Caspase‑3. In both *in vitro* and *in vivo* ICH models, this mechanism markedly attenuates endothelial apoptosis, preserves endothelial tight junction integrity, and stabilizes the BBB. As a result, cerebral edema and hematoma expansion are reduced at 3 days after ICH in mice, contributing to improved neurological recovery.

## Supplementary Information

Below is the link to the electronic supplementary material.

ESM 1 (PDF 69.0 MB)

## Data Availability

No datasets were generated or analyzed during the current study.

## Abbreviations

BBB: Blood–brain barrier
CNS: Central nervous system
EB: Evans blue
ICH: Intracerebral hemorrhage
NTA: Nanoparticle tracking analysis
OGD: Oxygen–glucose deprivation
TBI: Traumatic brain injury
TEER: Transendothelial electrical resistance
TEM: Transmission electron microscopy
MKK4: Mitogen-activated protein kinase kinase 4
p-MKK4: Phosphorylated mitogen-activated protein kinase kinase 4
JNK: C-Jun N-terminal kinase
p-JNK: Phosphorylated c-Jun N-terminal kinase
Grb2: Growth factor receptor-bound protein 2
Cacna2d3: Calcium voltage-gated channel auxiliary subunit alpha2delta 3
Rasgrf2: Ras protein-specific guanine nucleotide-releasing factor 2
Rapgef2: Rap guanine nucleotide exchange factor 2
Cyt c: Cytochrome c
Bax: Bcl-2-associated X protein
Bcl-2: B-cell lymphoma 2
Caspase-3: Cysteine-aspartic protease-3
MAPK: Mitogen-activated protein kinase
