# Supplementary material for: Extracellular Vesicles Derived from M2 Microglia and Enriched in miR‑27b‑3p Attenuate Mitochondria‑Dependent Endothelial Apoptosis via the MKK4/JNK Pathway and Alleviate BBB Disruption After Intracerebral Hemorrhage
Source: Mol Neurobiol. 2026 Feb 11;63(1):431. doi: 10.1007/s12035-026-05718-x (PMC12894209; doi:10.1007/s12035-026-05718-x)

Dear Editor and Reviewers,

Thank you for your review and support of our manuscript. This document contains the original Western blot (WB) data referenced in the article.

We have labeled the protein type, group designation, marker catalog number, and corresponding location within the manuscript at the top of each image. Protein targets are annotated on the left side of the figures. The WB bands utilized in the manuscript are marked with red boxes, and the internal controls are highlighted with green boxes. Protein molecular weights are indicated to the right of the marker. Please do not hesitate to contact us with any questions.

All our target proteins and internal controls were developed using the same PVDF membrane. The protocol involved developing the target proteins first, followed by membrane regeneration, and subsequent incubation with internal control antibodies. For certain groups, the PVDF membrane was incubated with multiple target proteins prior to the final incubation with the internal control. Consequently, different target proteins within specific groups may share the same internal control. To prevent unnecessary confusion during the review of the original WB data, we have explicitly noted the groups and target proteins that utilize the same internal control below.

Sincerely,

Zengguang Wang

***

Groups and target proteins sharing the same internal control:

Cell; Group: Sham; Hemin; M0-Evs; M2-Evs (Caspase-3 & Bax)

Mouse; Group: Sham; ICH; M0-Evs; M2-Evs (Caspase-3 & Bax)

Mouse; Group：Sham; ICH; M0-Evs; M2-Evs (Claudin 5 & Occludin)

Mouse; Group: Sham; ICH; M2-Evs; miR-27b-3p cn; miR-27b-3p k/d (Caspase-3 & Bax)

Uncropped Gels and Blots image(s)

BV-2; CD206; Repeat*6
Group:Control, IL-4
Figure. 1C

Marker: ThermoFisher 26616

CD206

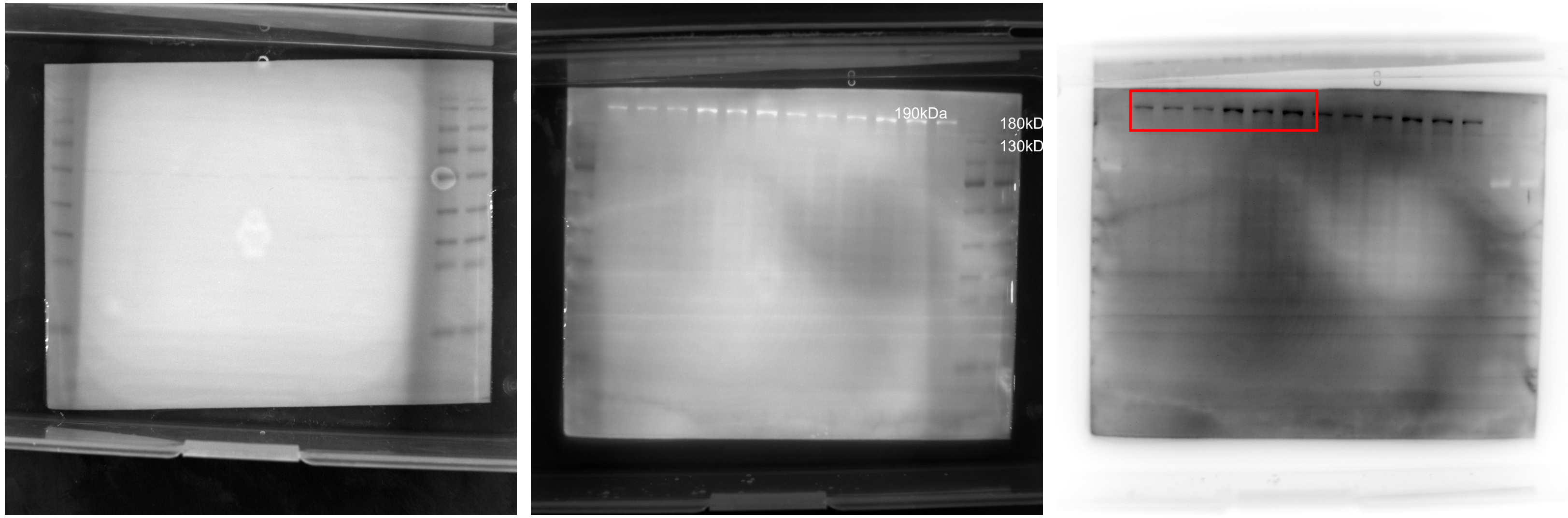

β-Tubulin

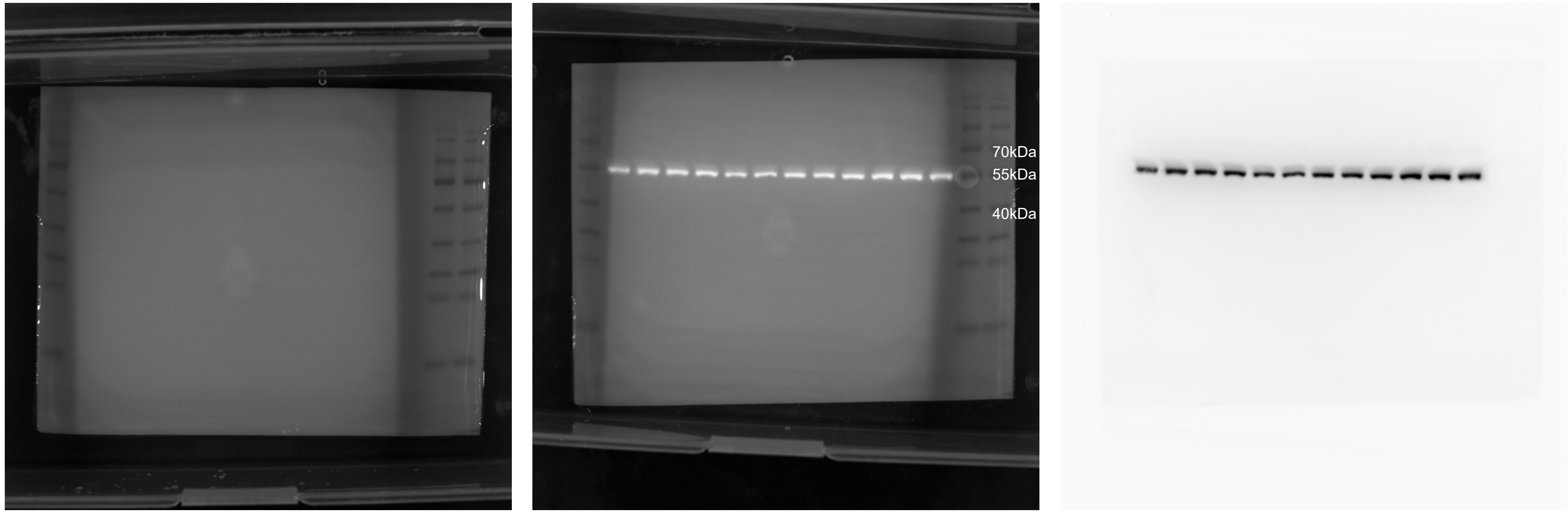

BV-2; Arg-1; Repeat*6
Group:Control, IL-4
Figure. 1C

Marker: ThermoFisher 26616

Arg-1

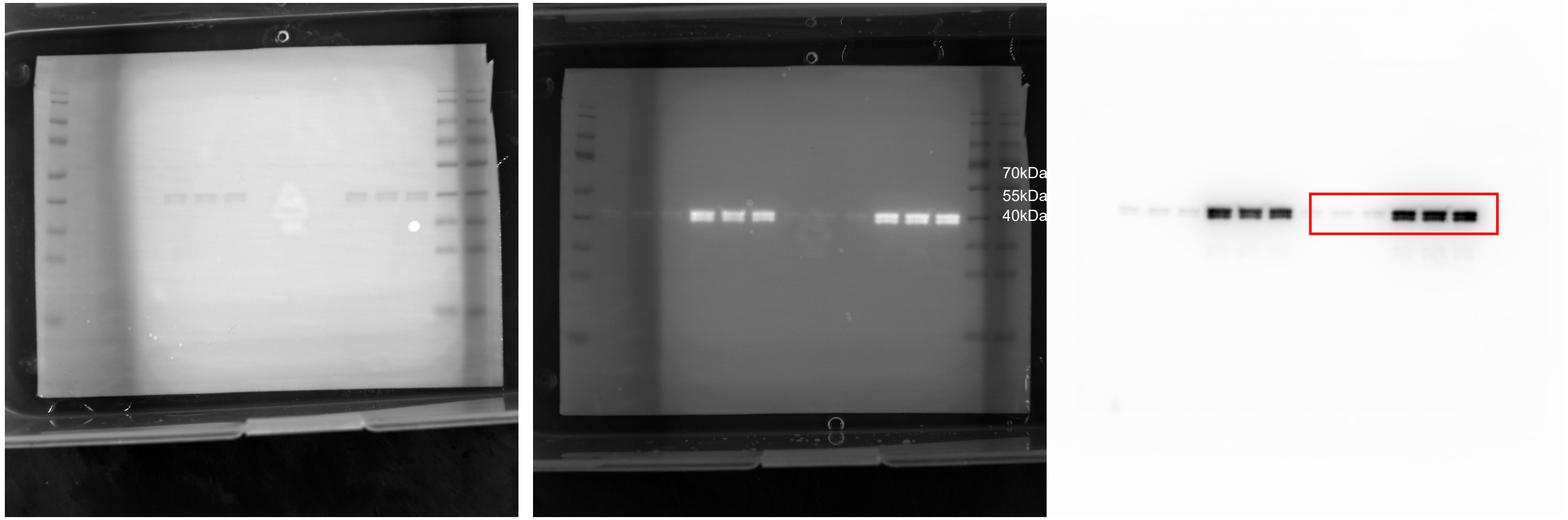

β-Tubulin

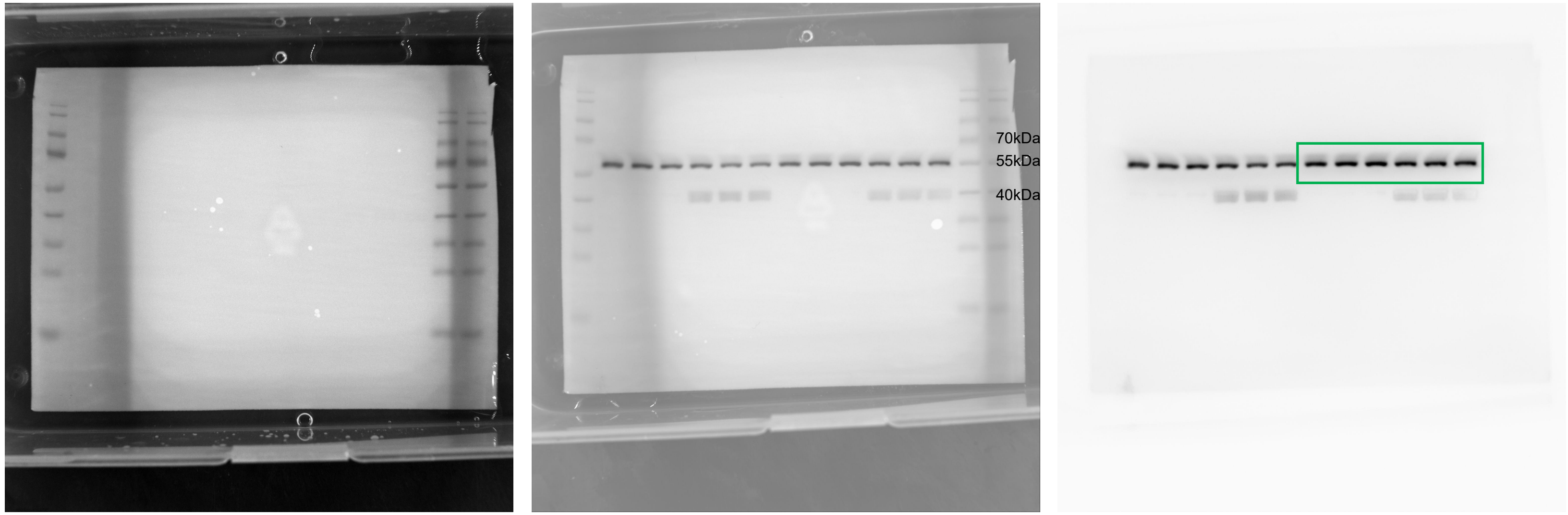

BV-2&Evs; CD206；Repeat*3

Marker: ThermoFisher 26616

Group:M0-Bv2；M2-Bv2；M0-Evs；M2-Evs

Figure. 1G

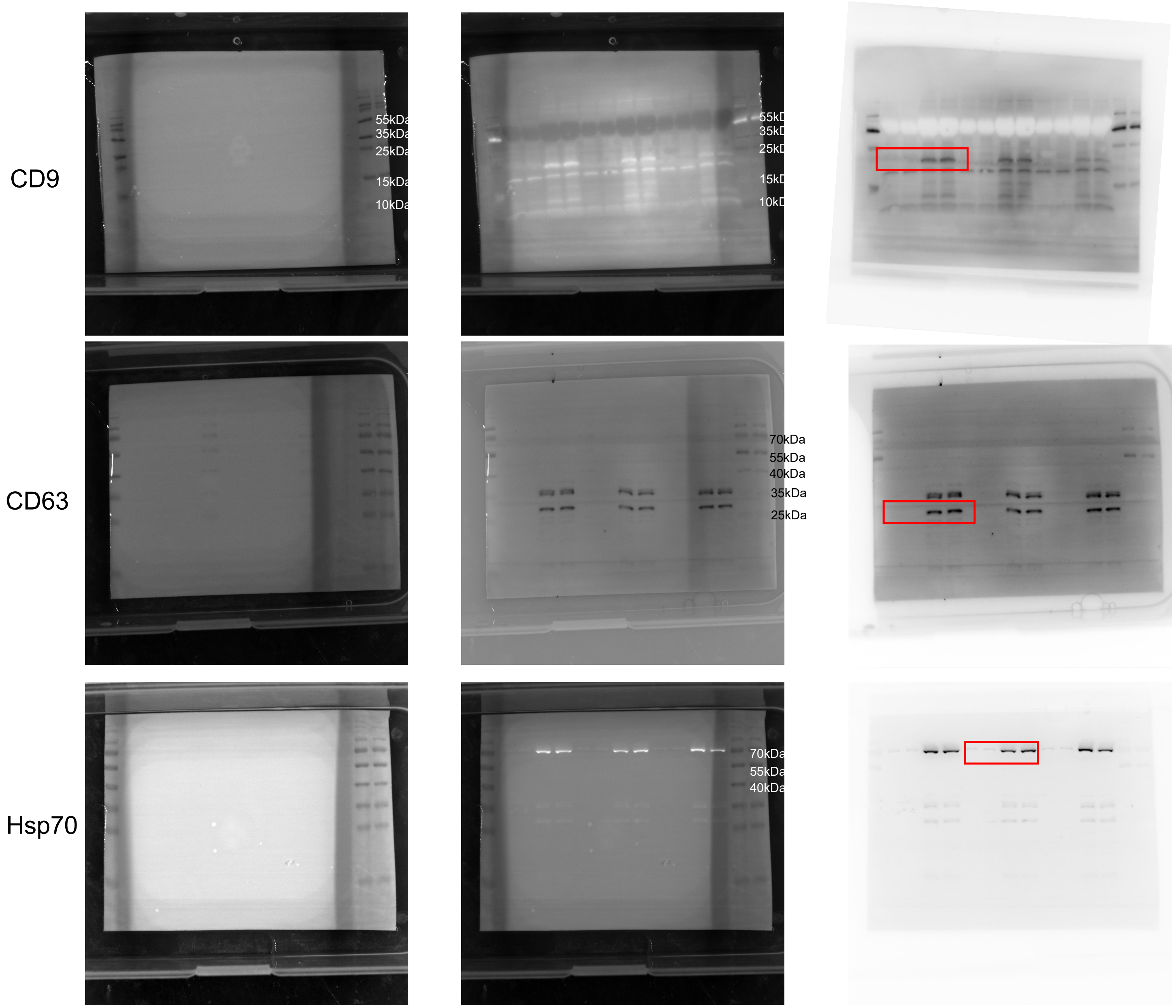

Cell; Caspase-3；Repeat*3

Group:Sham, Hemin, M0-Evs, M2-Evs

Figure. 2G

Marker: ThermoFisher 26616

Caspase-3

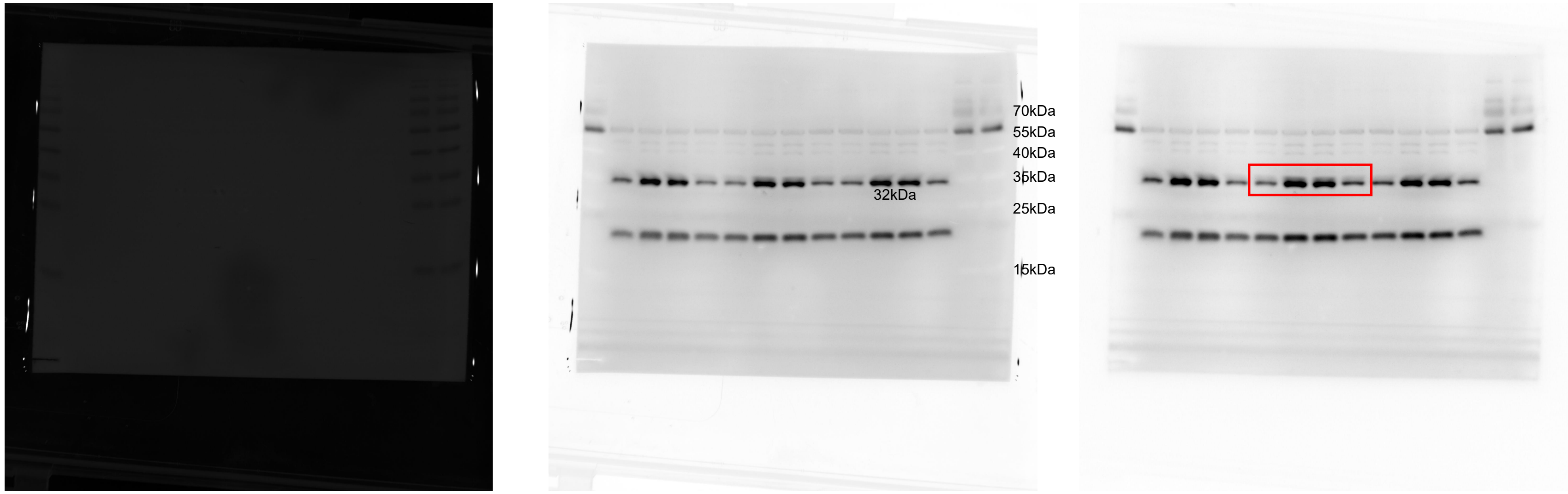

β-Actin

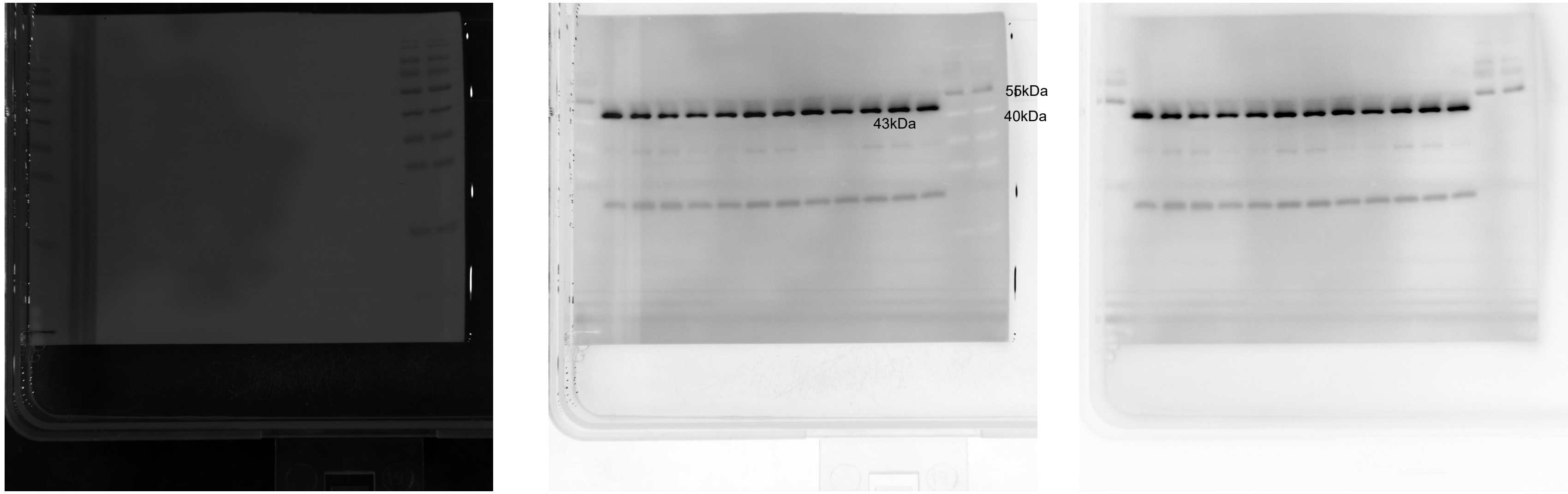

Cell; Bax; Repeat*3

Group:Sham, Hemin, M0-Evs, M2-Evs

Figure. 2G

Marker: ThermoFisher 26616

Bax

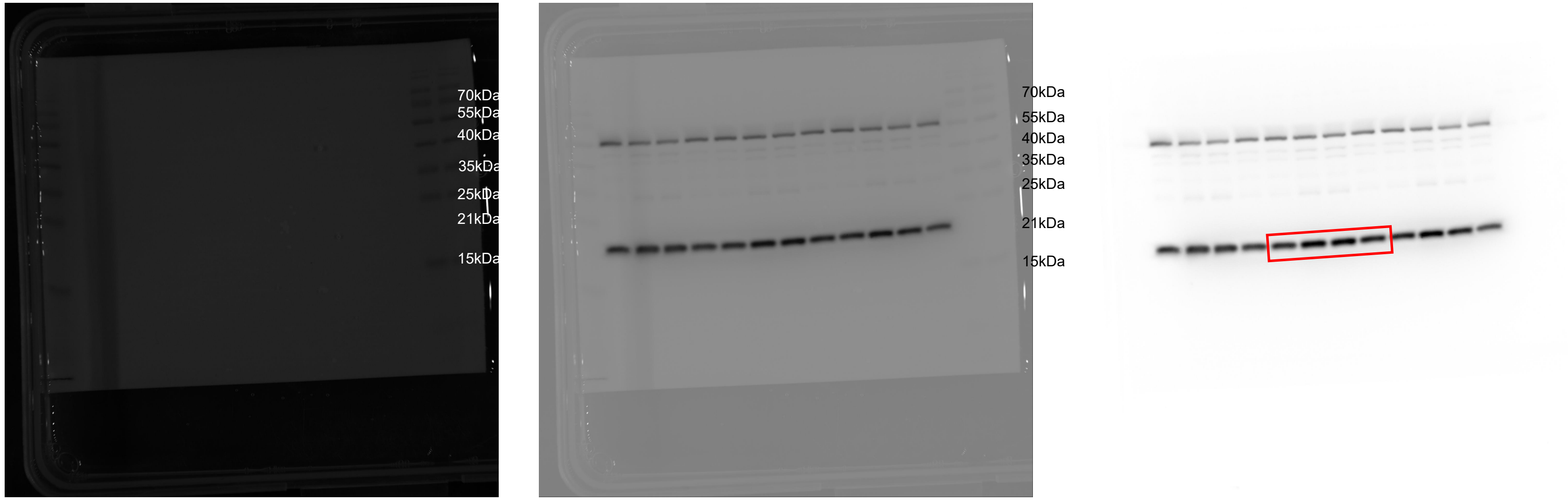

β-Actin

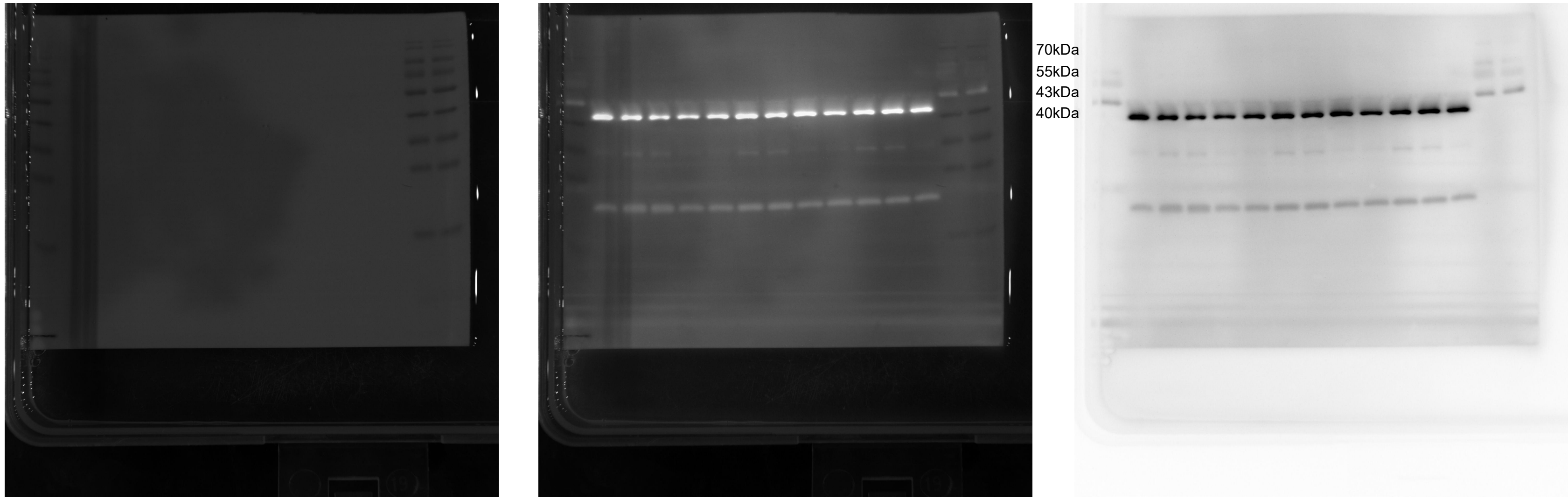

Cell; Bcl-2； Repeat*3

Group:Sham, Hemin, M0-Evs, M2-Evs

Figure. 2G

Marker: ThermoFisher 26616

Bcl-2

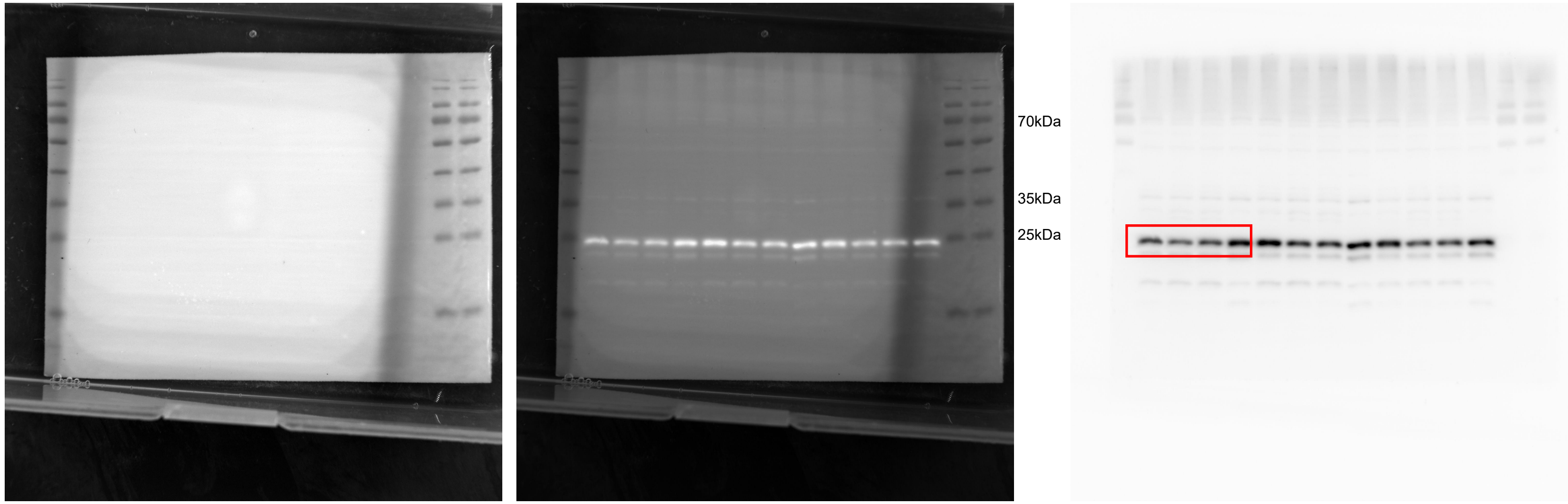

β-Actin

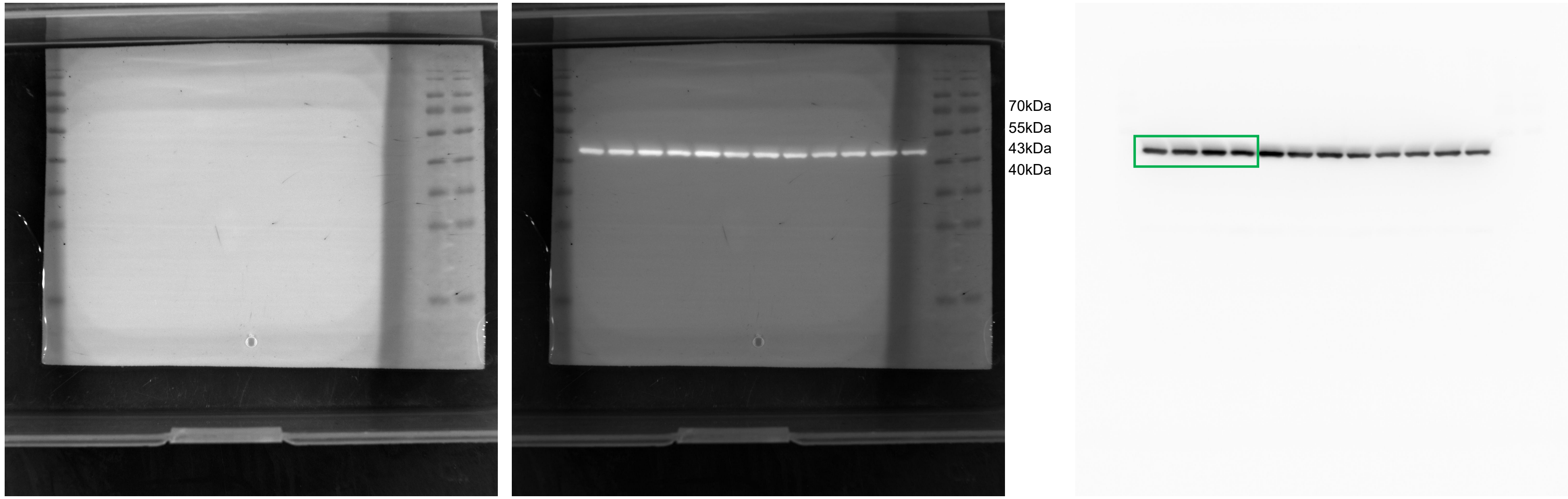

Cell; Cyt-C；Repeat*3

Group:Sham, Hemin, M0-Evs, M2-Evs

Figure. 2G

Marker: ThermoFisher 26616

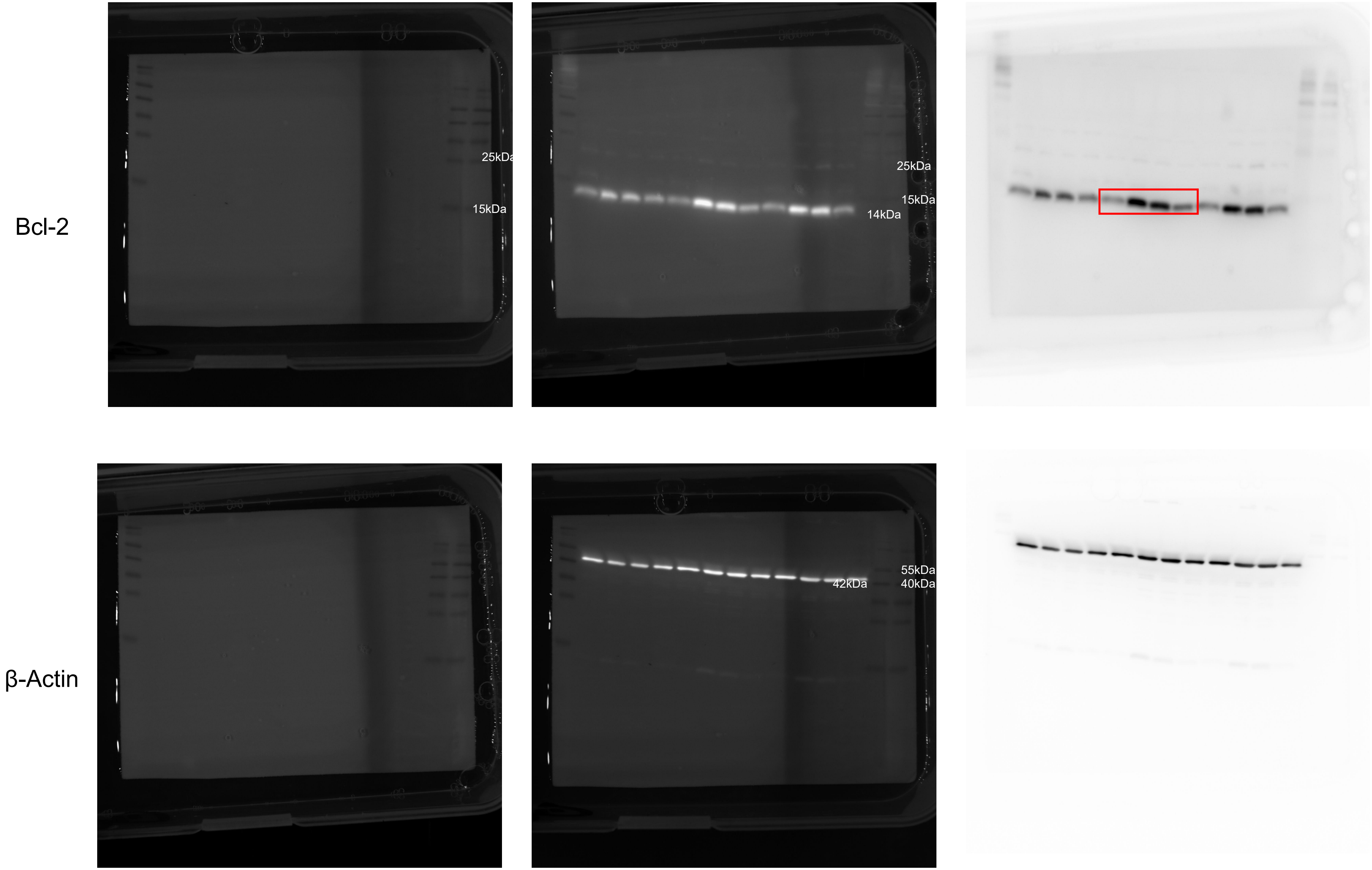

Cell; Claudin 5; Repeat*3

Marker: ThermoFisher 26616

Group:Sham, Hemin, M0-Evs, M2-Evs

Figure. 2N

Claudin 5

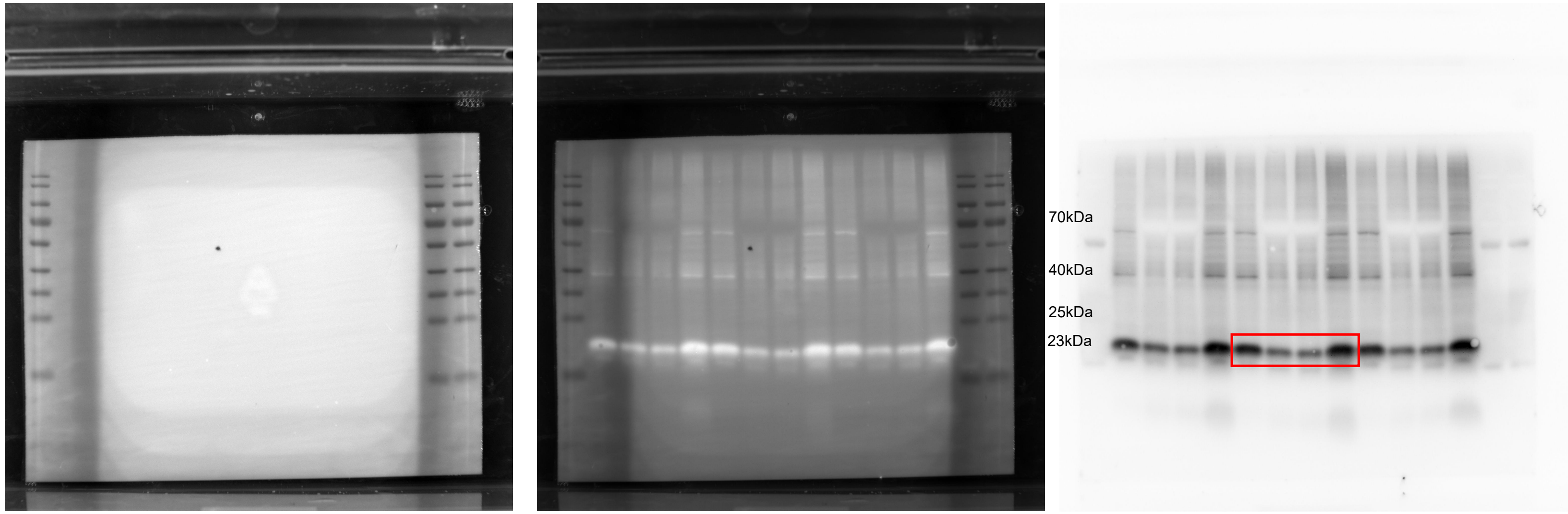

β-Actin

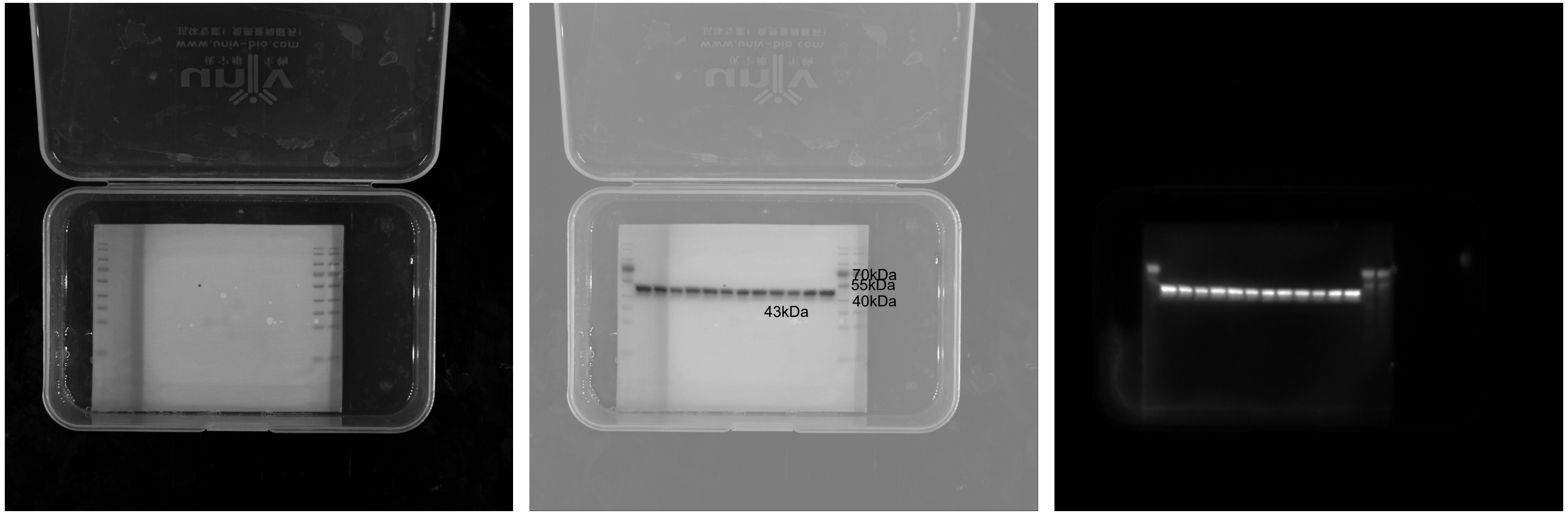

Cell; Occludin; Repeat*3
Group:Sham, Hemin, M0-Evs, M2-Evs
Figure. 2N

Marker: ThermoFisher 26616

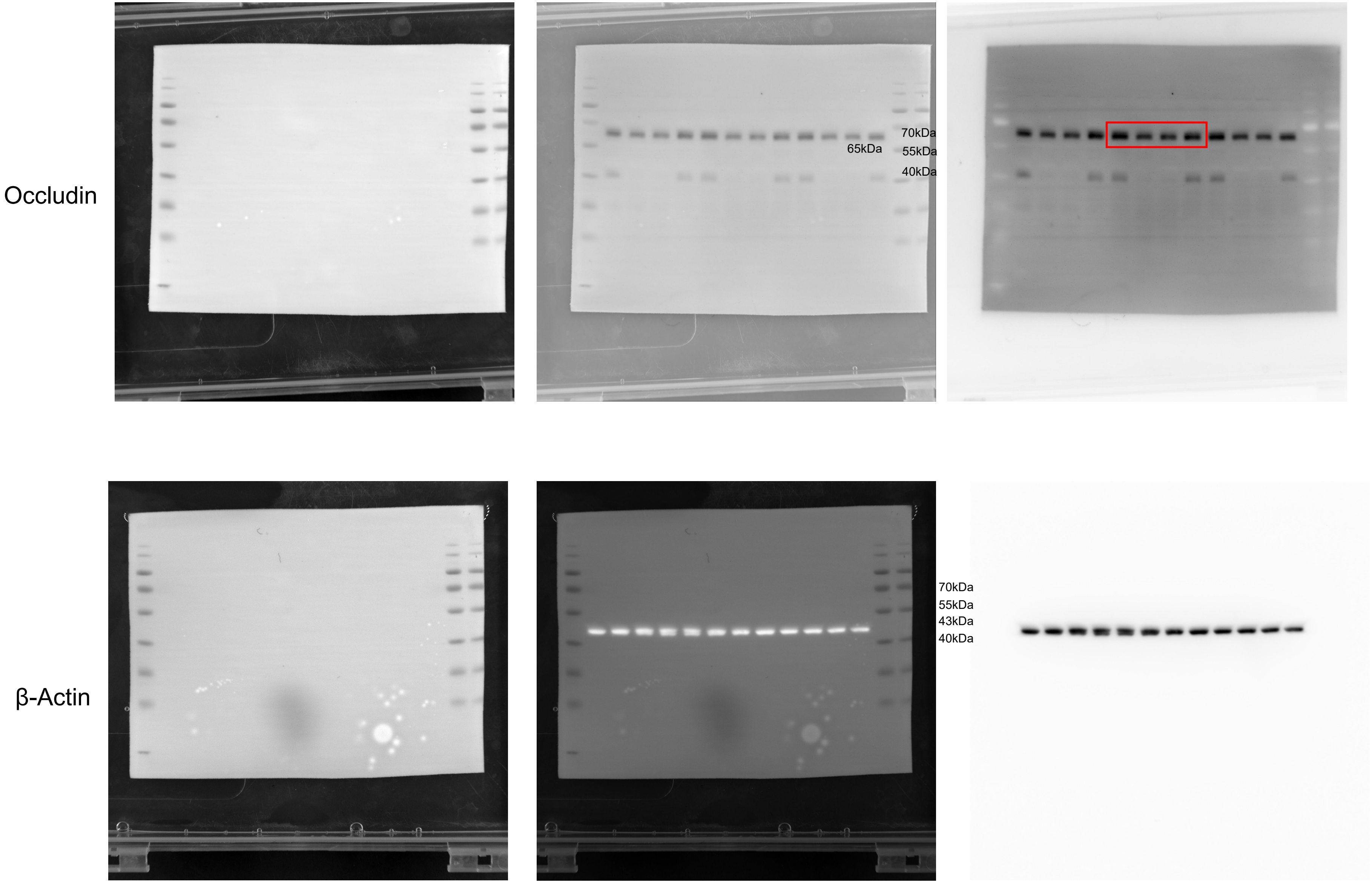

Cell; VE-Cadherin；Repeat*3
Group:Sham, Hemin, M0-Evs, M2-Evs
Figure. 2N

Marker: ThermoFisher 26616

VE-Cadhrein

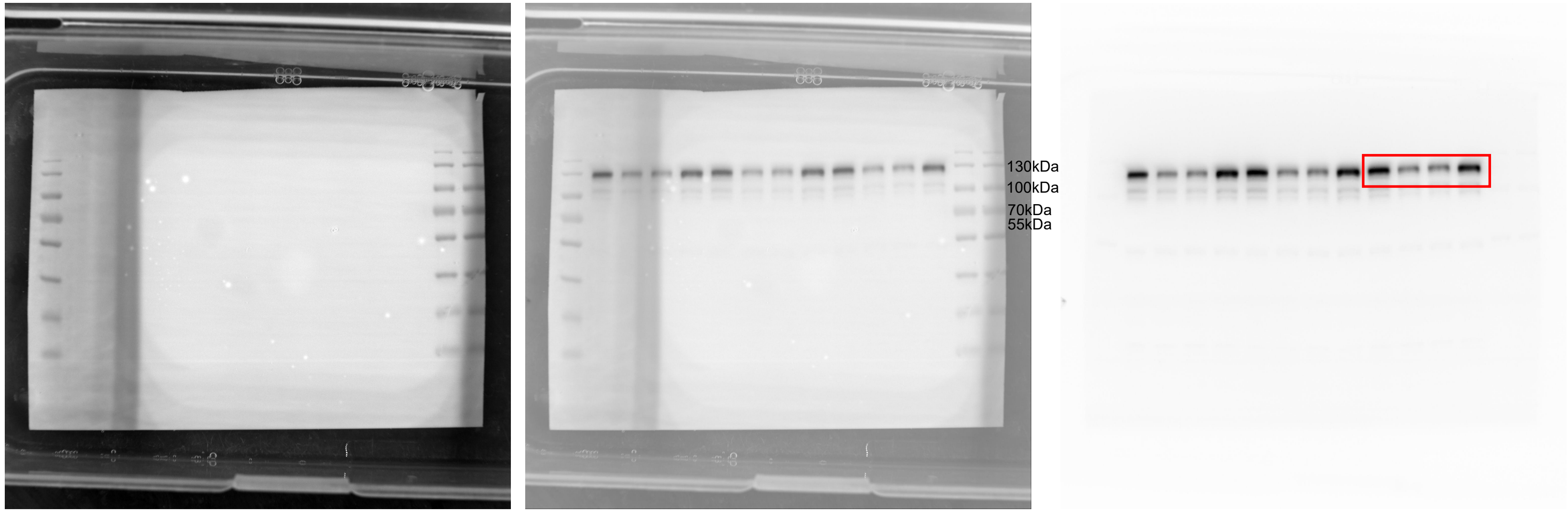

β-Actin

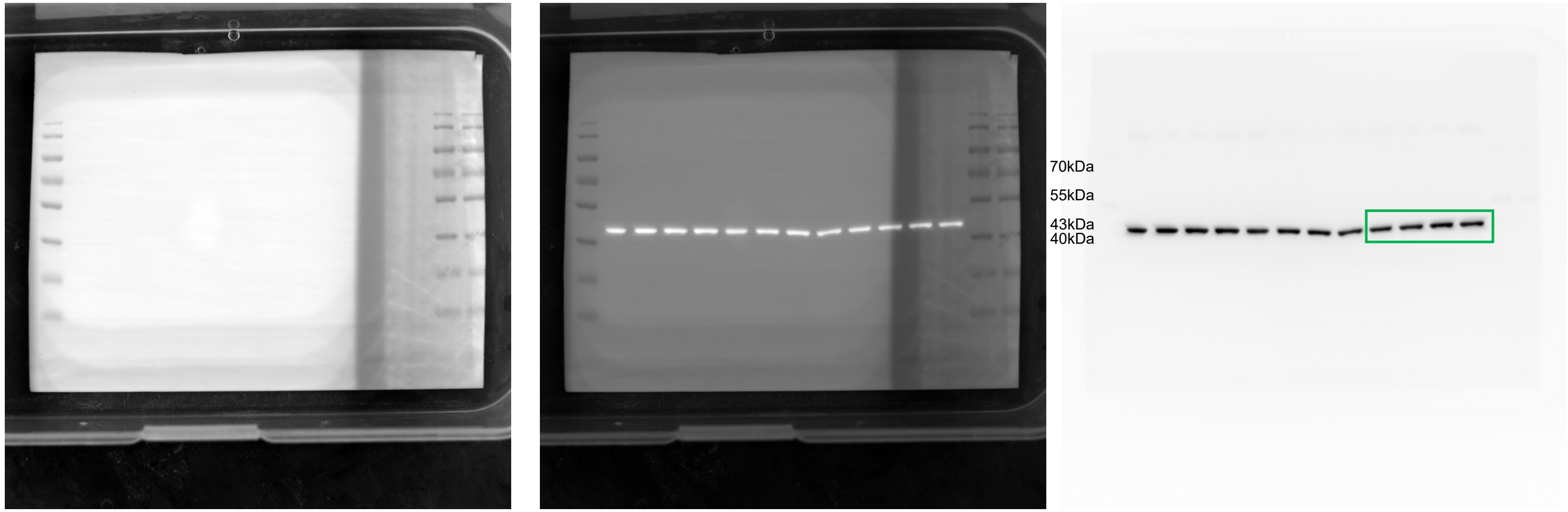

Mouse; Caspase-3； Repeat*6
Group:Sham, ICH, M0-Evs, M2-Evs
Figure. 4C

Marker: ThermoFisher 26616

Caspase-3

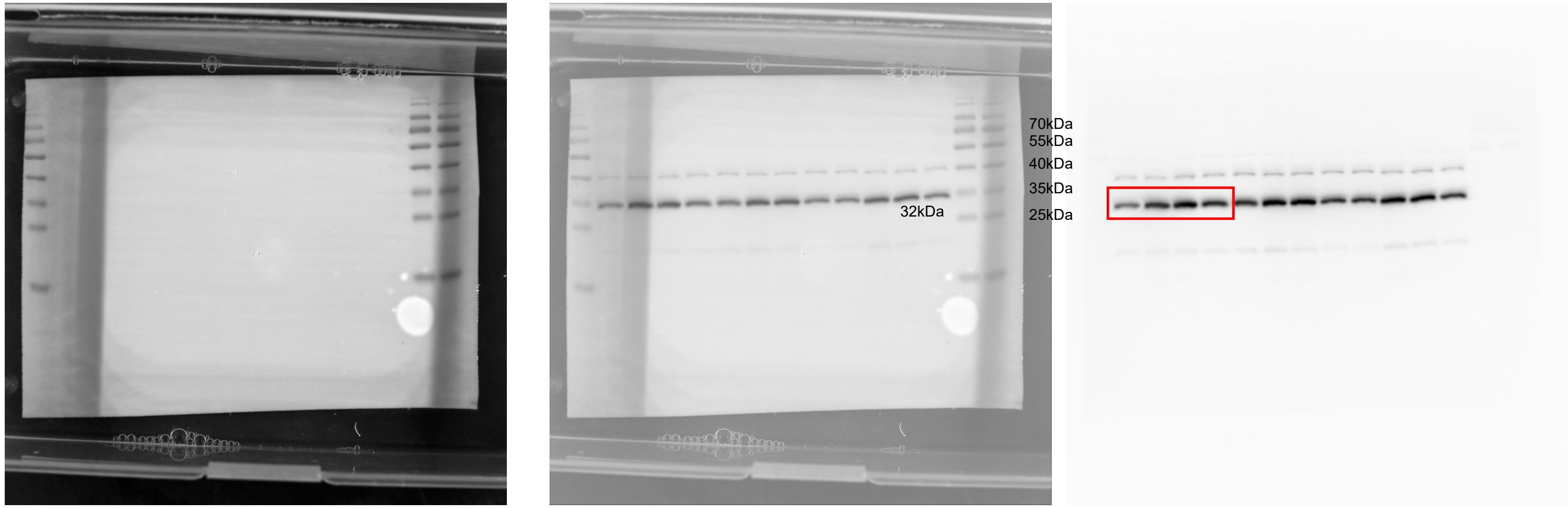

β-Actin

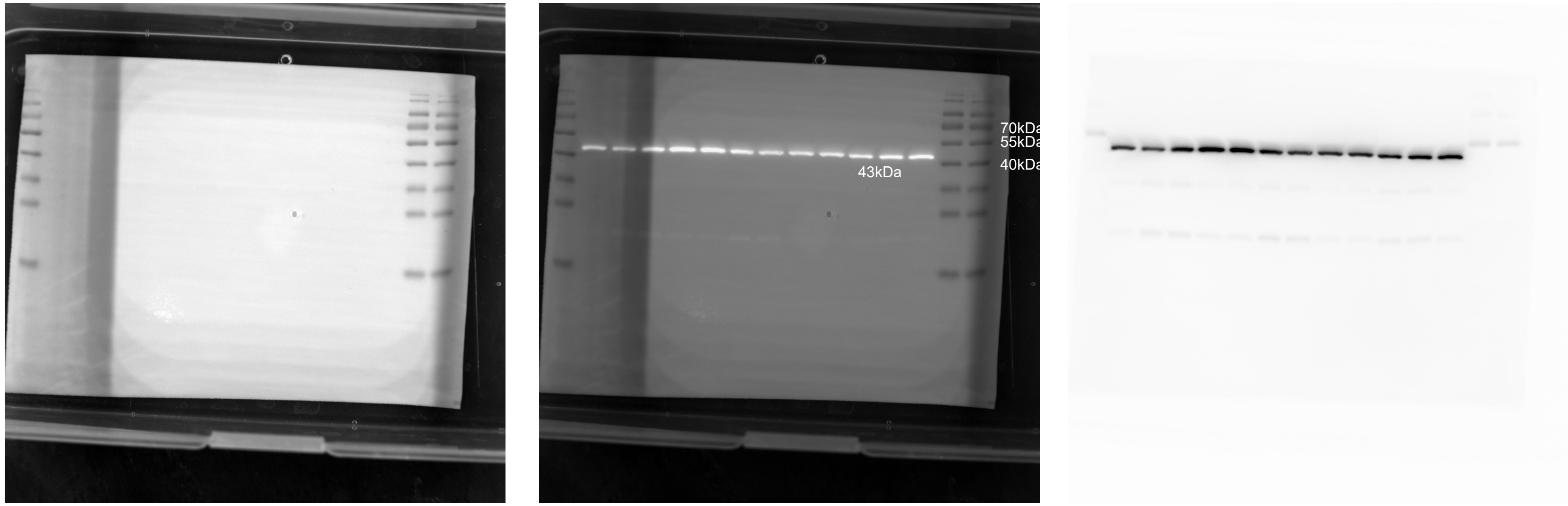

Marker: ThermoFisher 26616

Caspase-3

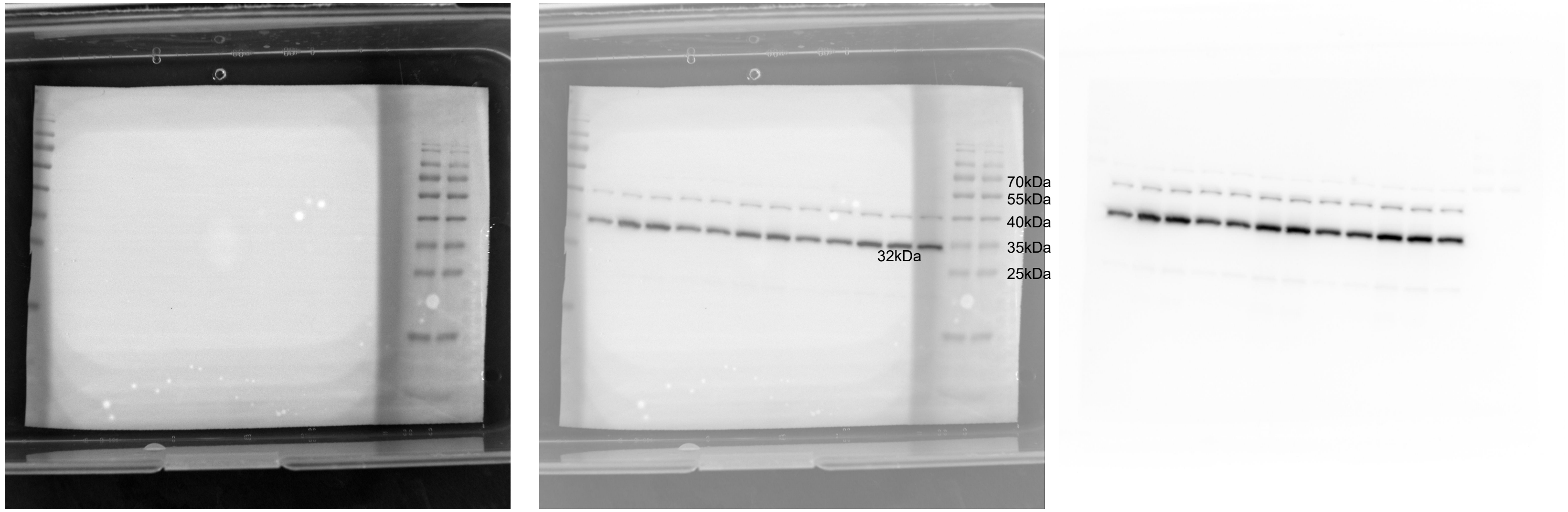

β-Actin

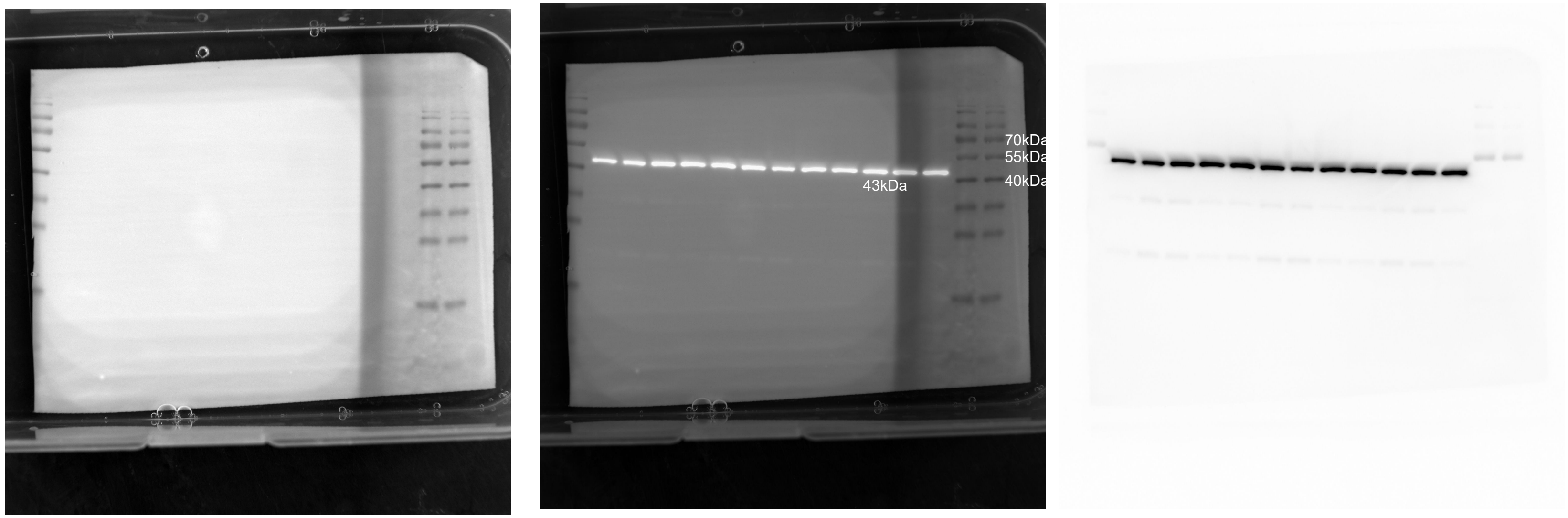

Mouse; Bax； Repeat*6
Group:Sham, ICH, M0-Evs, M2-Evs
Figure. 4C

Marker: ThermoFisher 26616

Bax

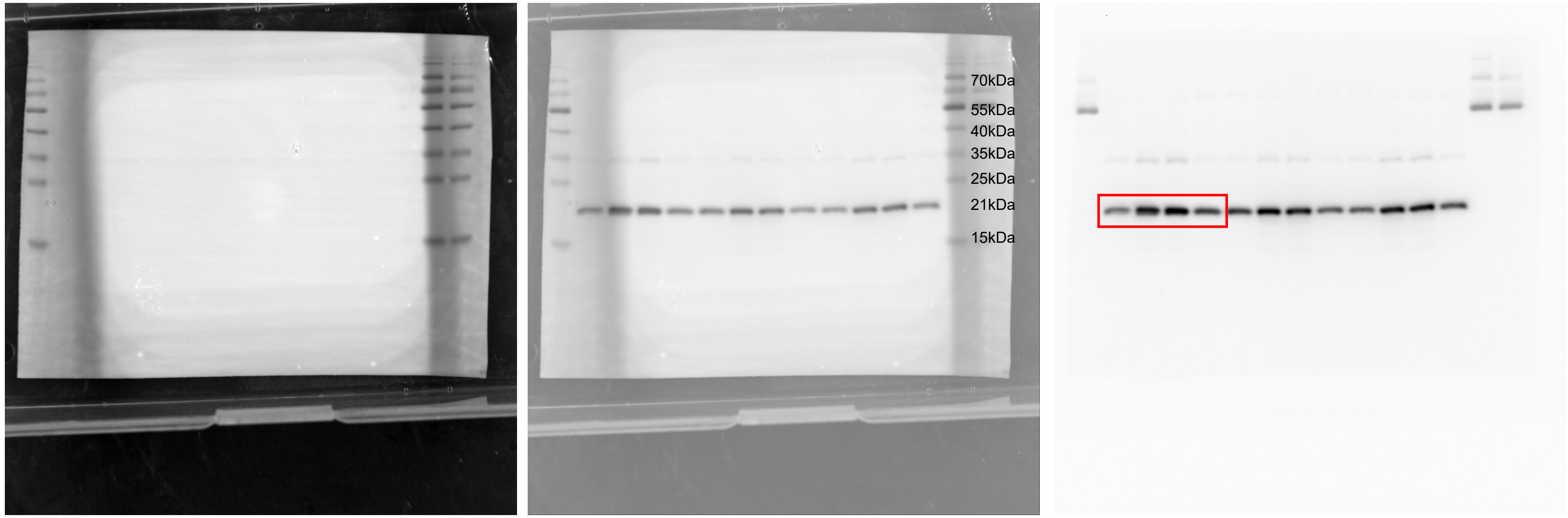

β-Actin

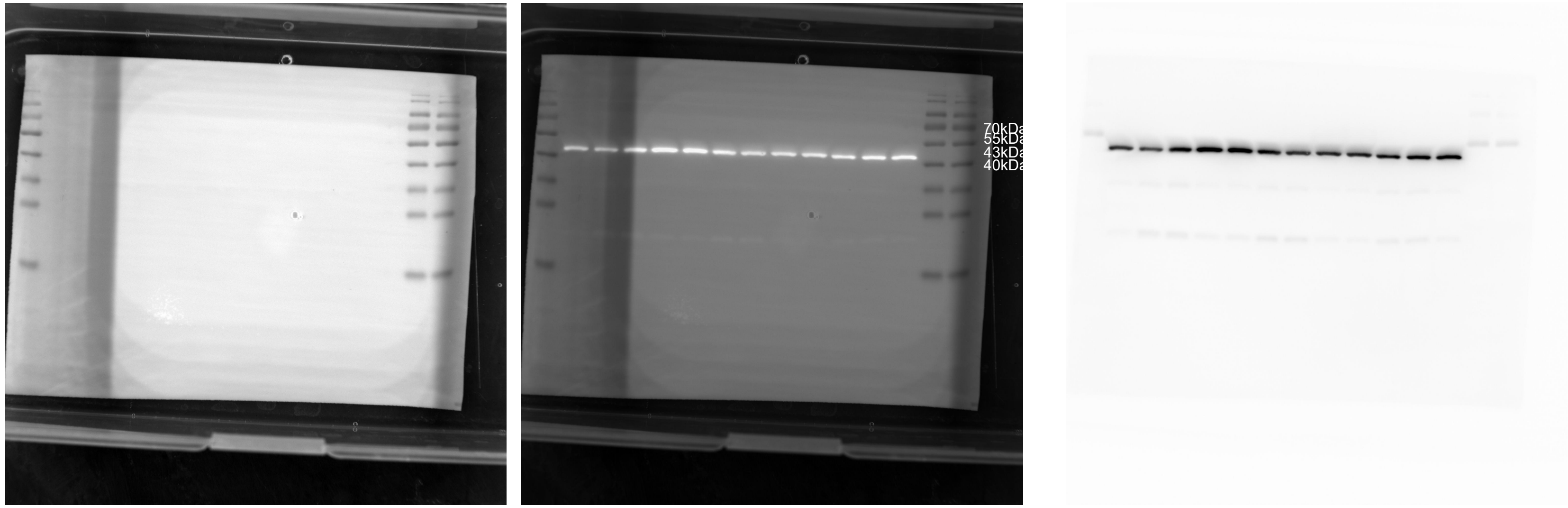

Marker: ThermoFisher 26616

Bax

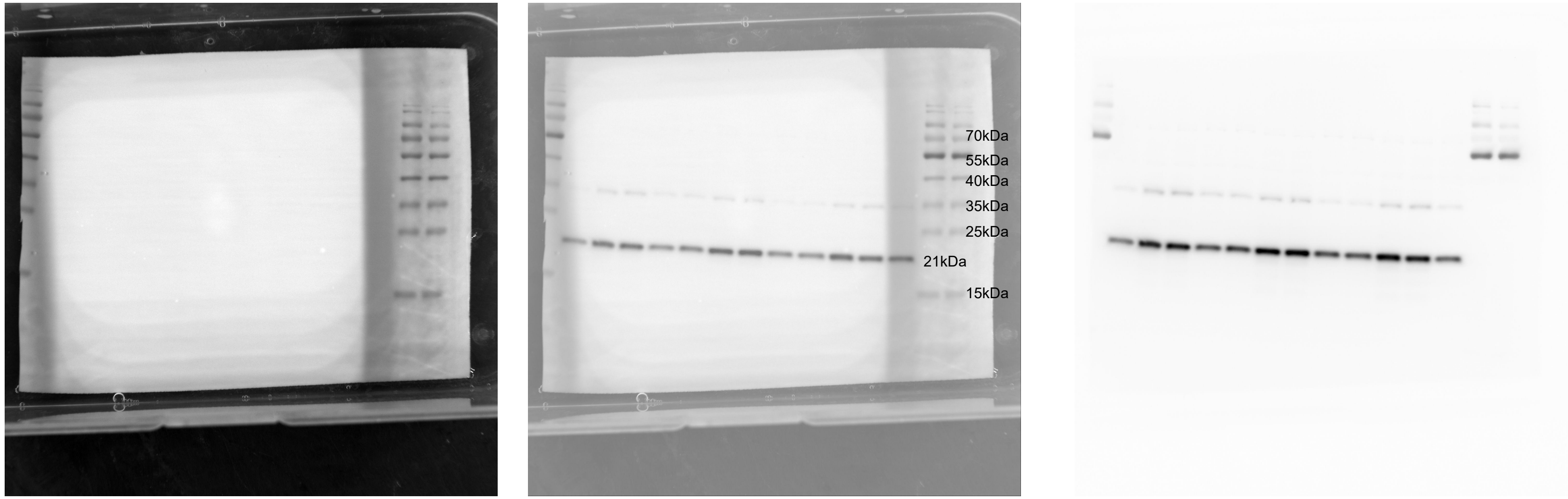

β-Actin

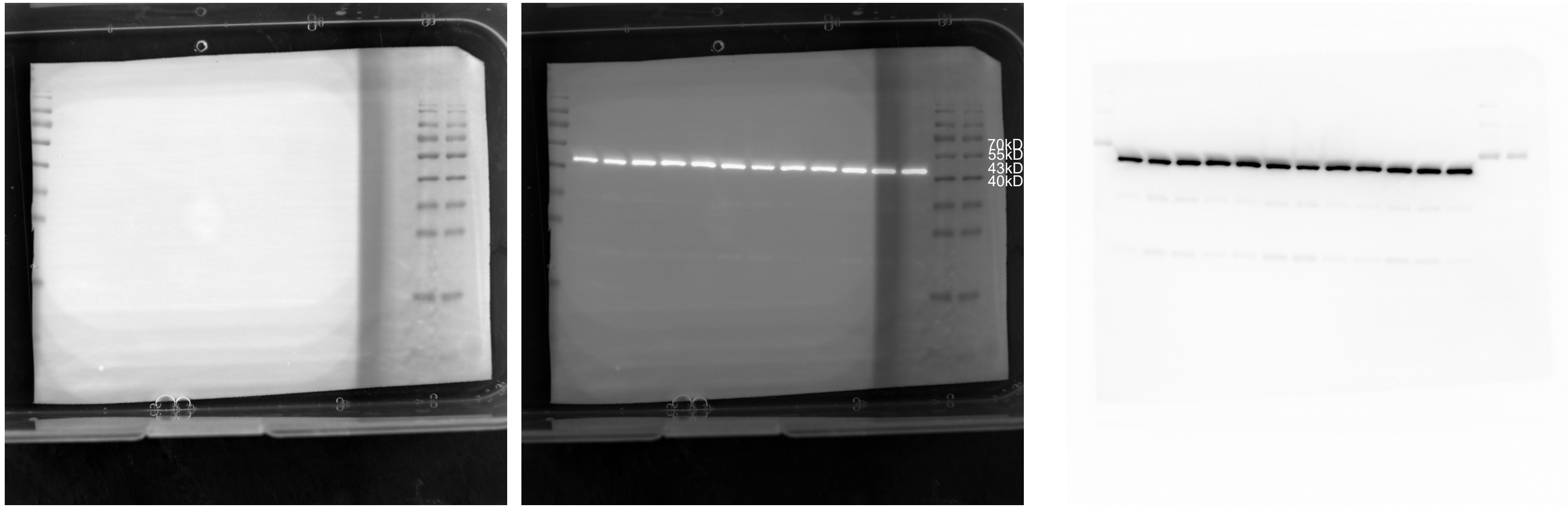

Mouse; Bcl-2； Repeat*6
Group:Sham, ICH, M0-Evs, M2-Evs
Figure. 4C

Marker: ThermoFisher 26616

Bcl-2

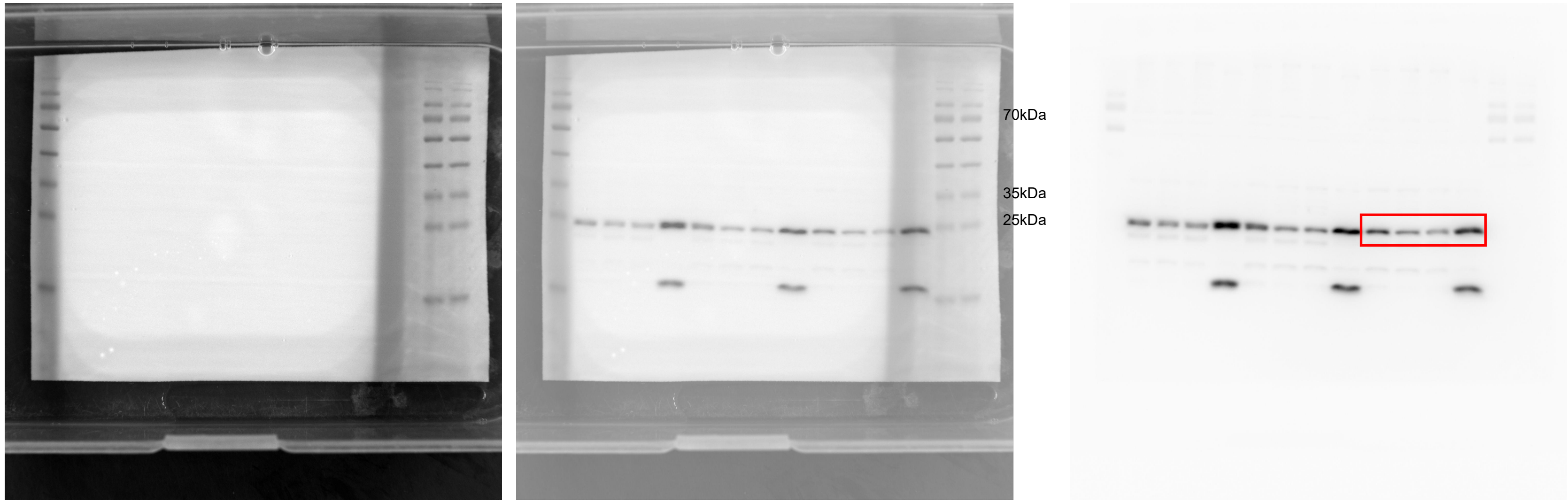

β-Actin

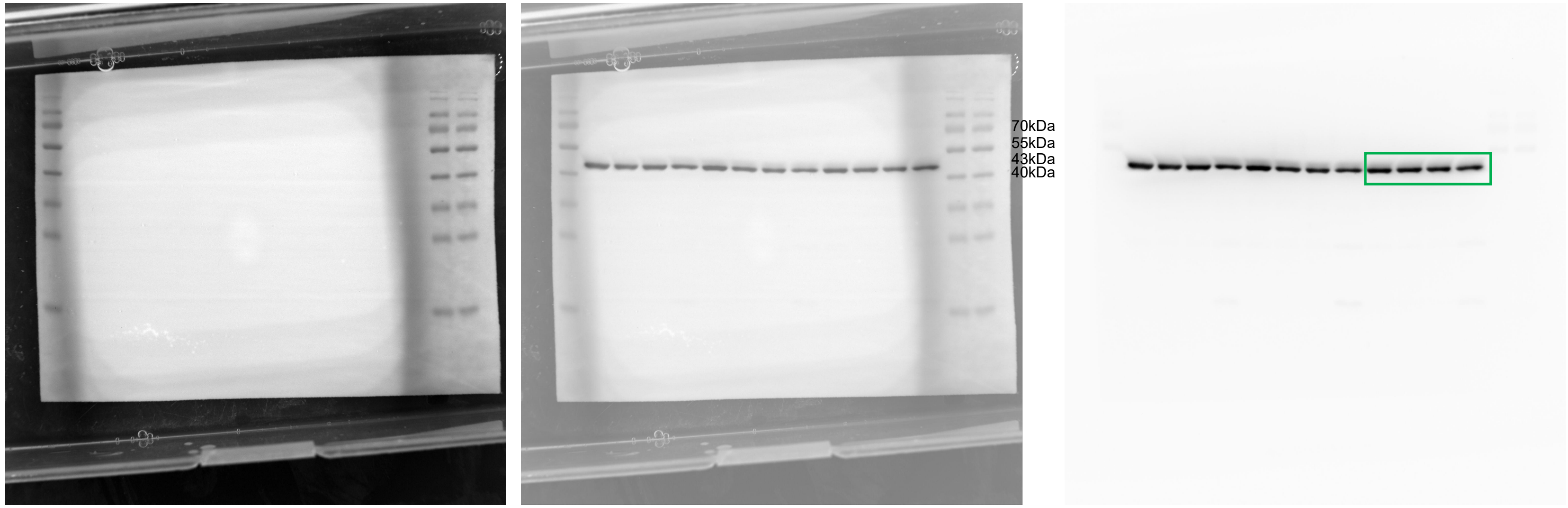

Marker: ThermoFisher 26616

Bcl-2

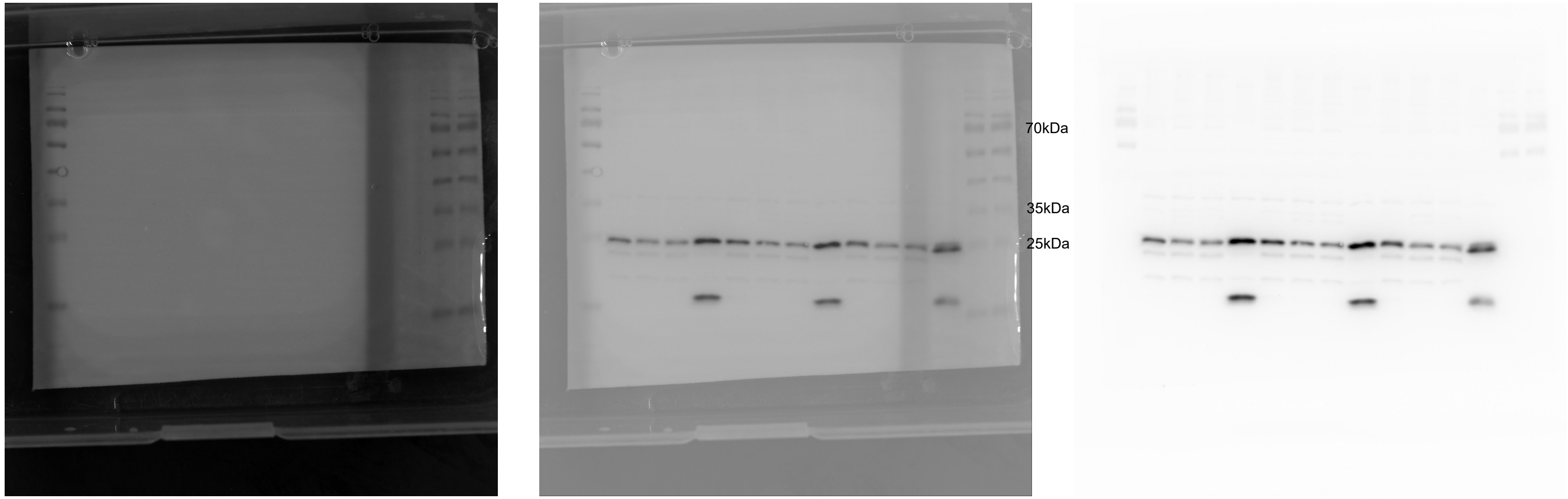

β-Actin

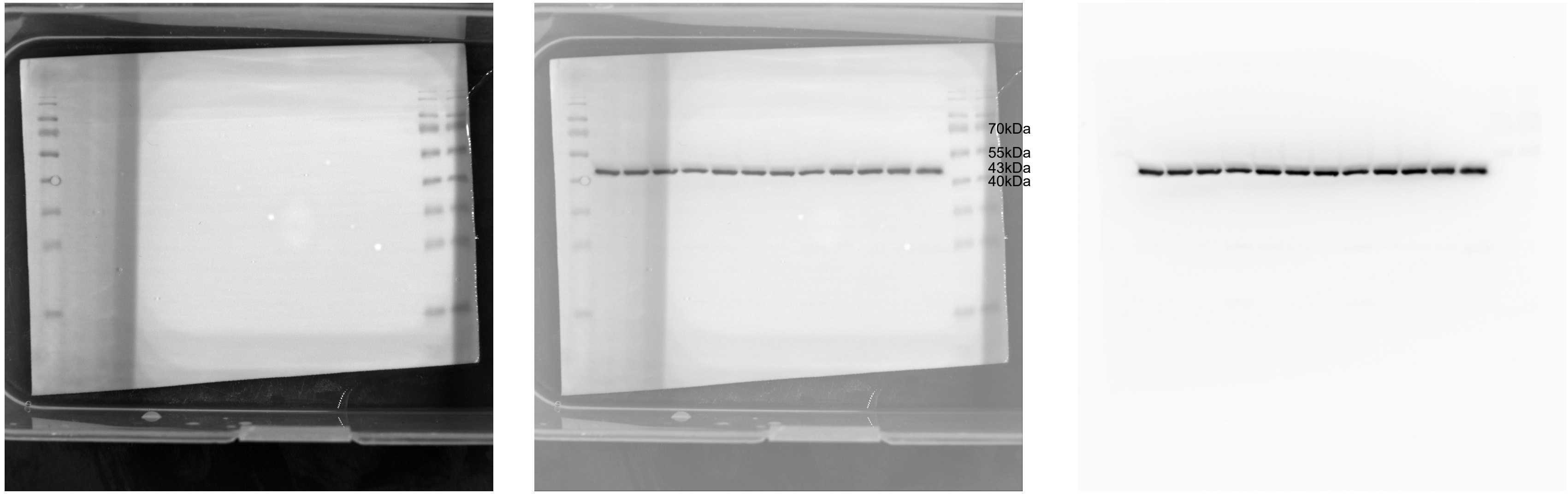

Mouse;Cyt-C；Repeat*6

Group:Sham, ICH, M0-Evs, M2-Evs

Figure. 4C

Marker: ThermoFisher 26616

Cyt-C

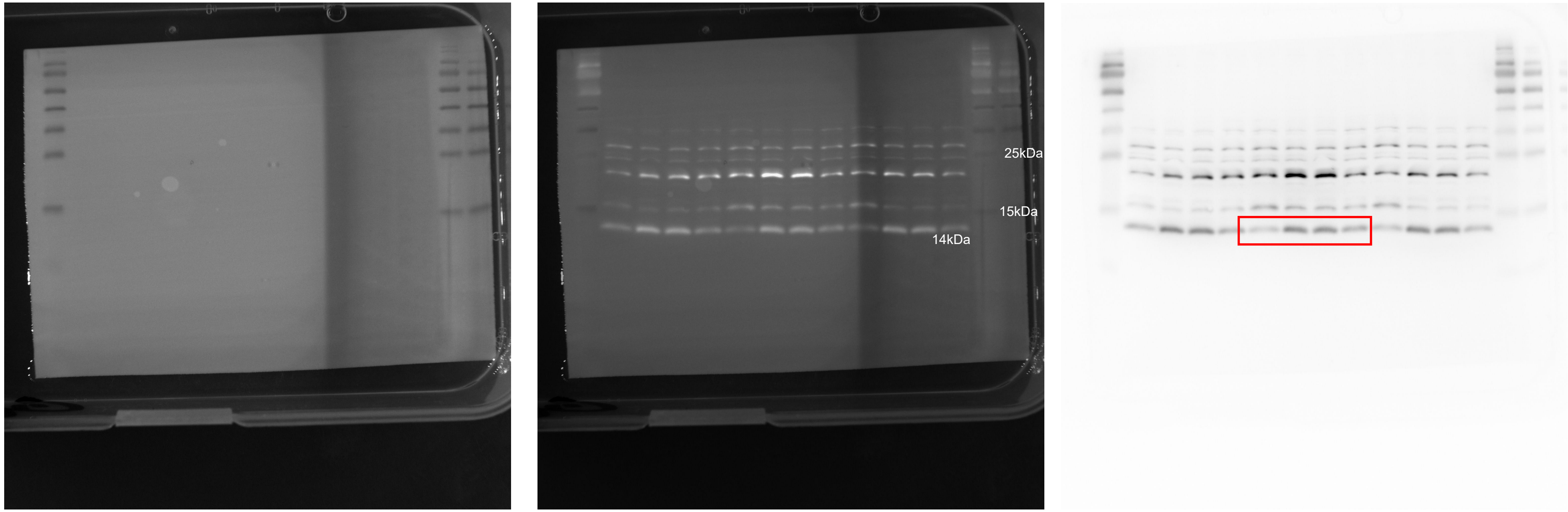

β-Actin

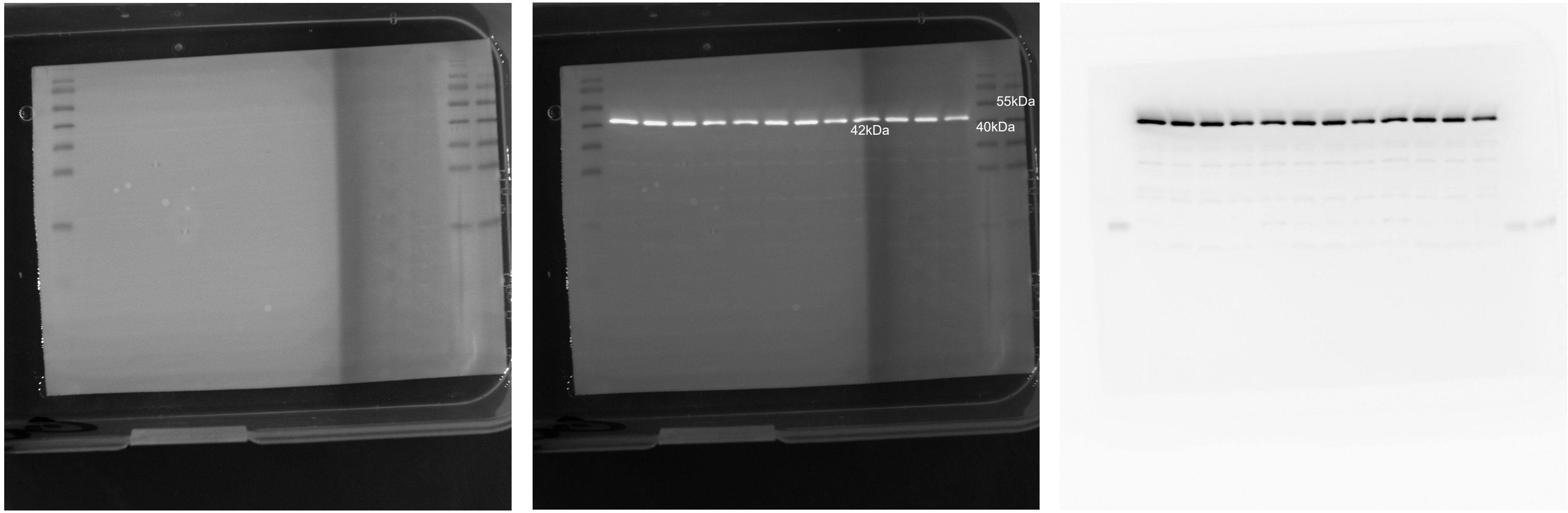

Marker: ThermoFisher 26616

Cyt-C

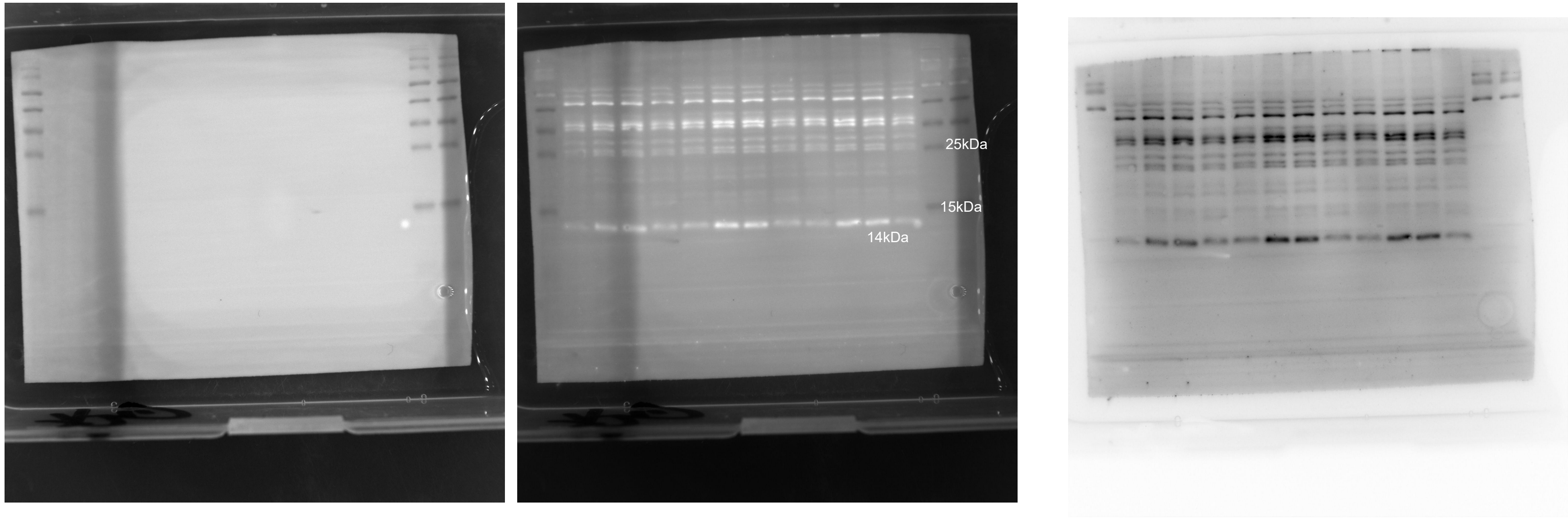

β-Actin

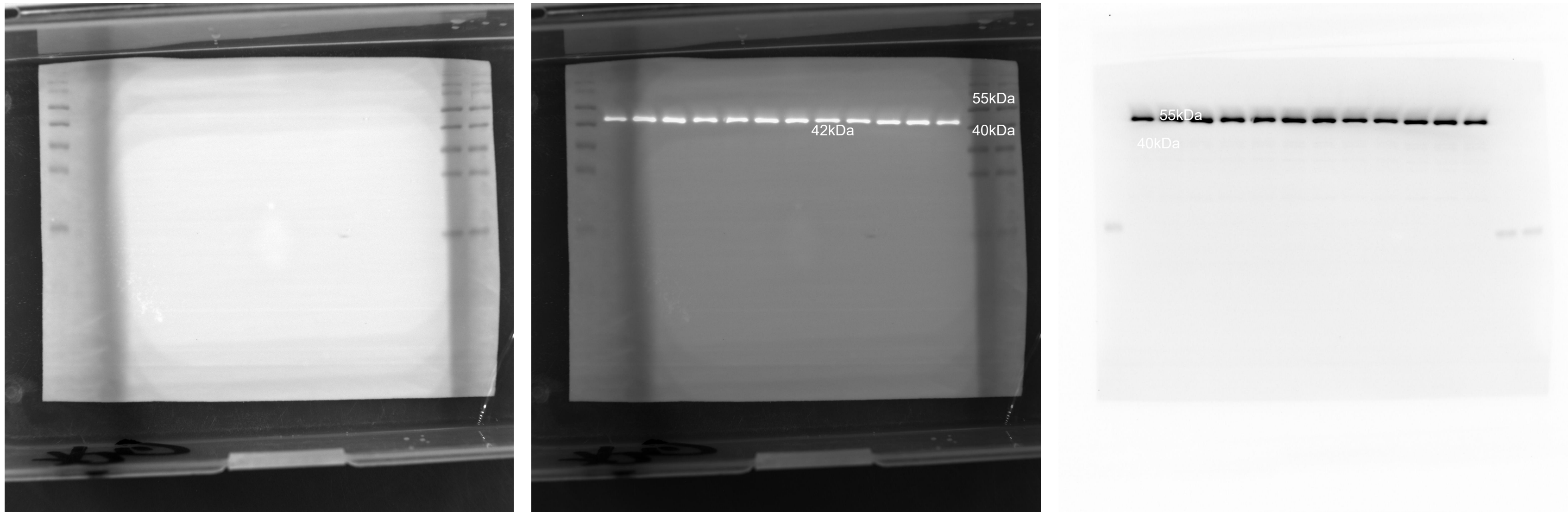

Mouse; Claudin 5; Repeat*6
Group:Sham, ICH, M0-Evs, M2-Evs
Figure. 4K

Marker: ThermoFisher 26616

Claudin 5

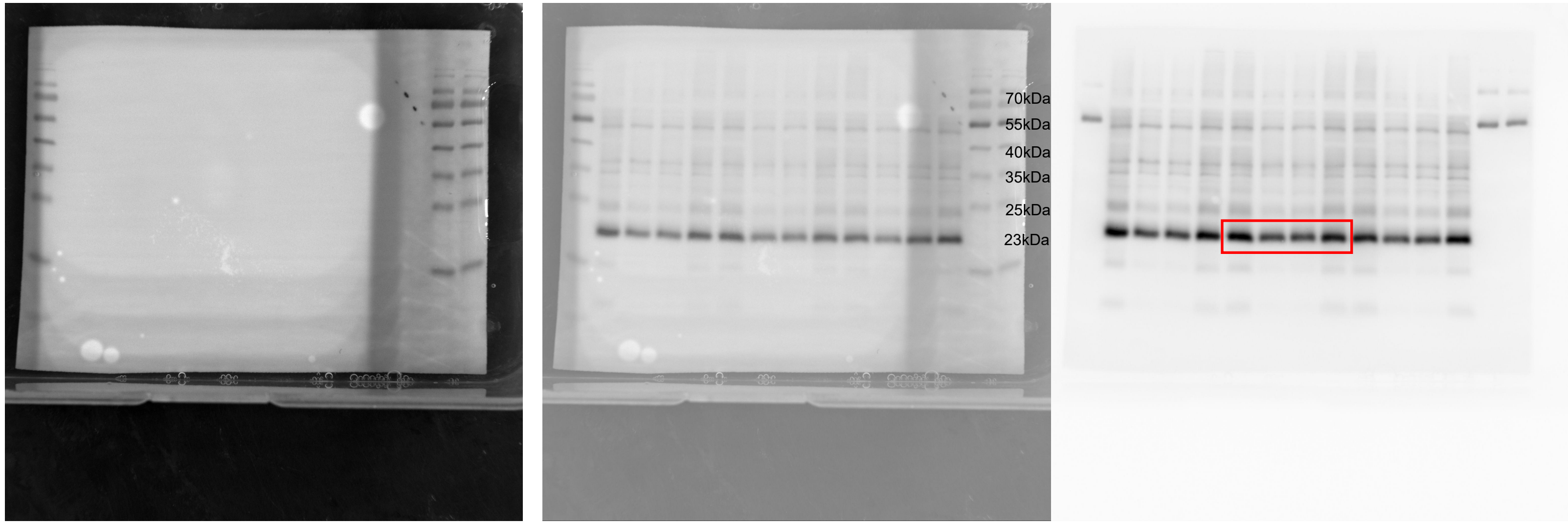

β-Actin

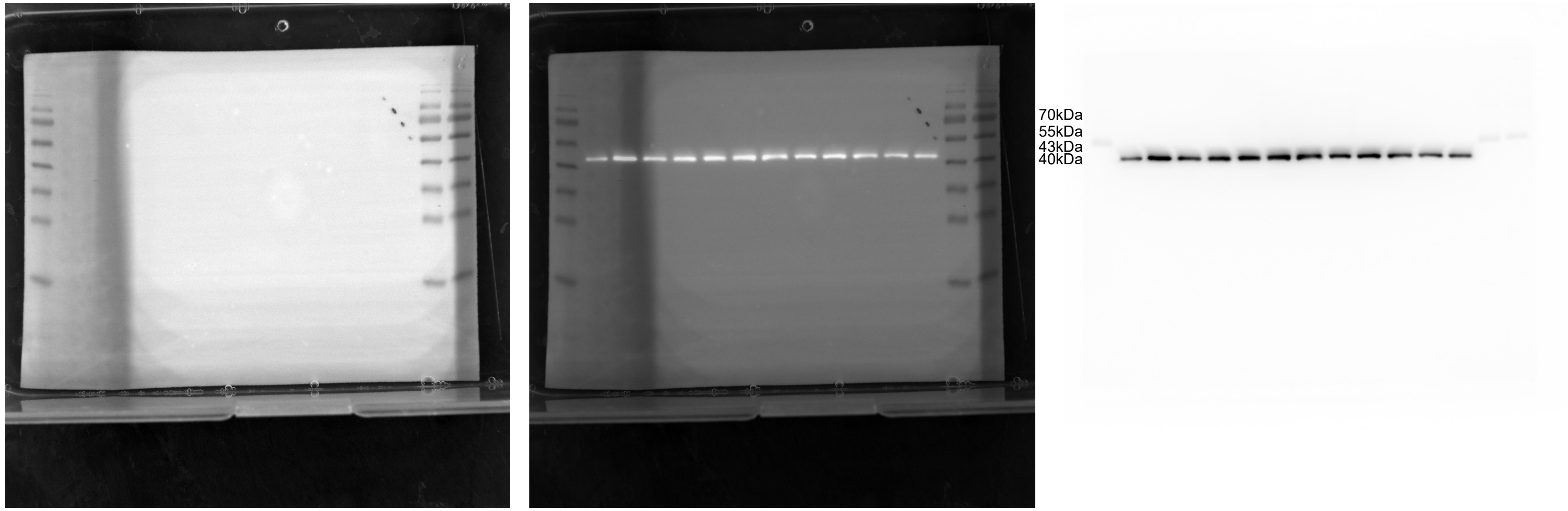

Marker: ThermoFisher 26616

Claudin 5

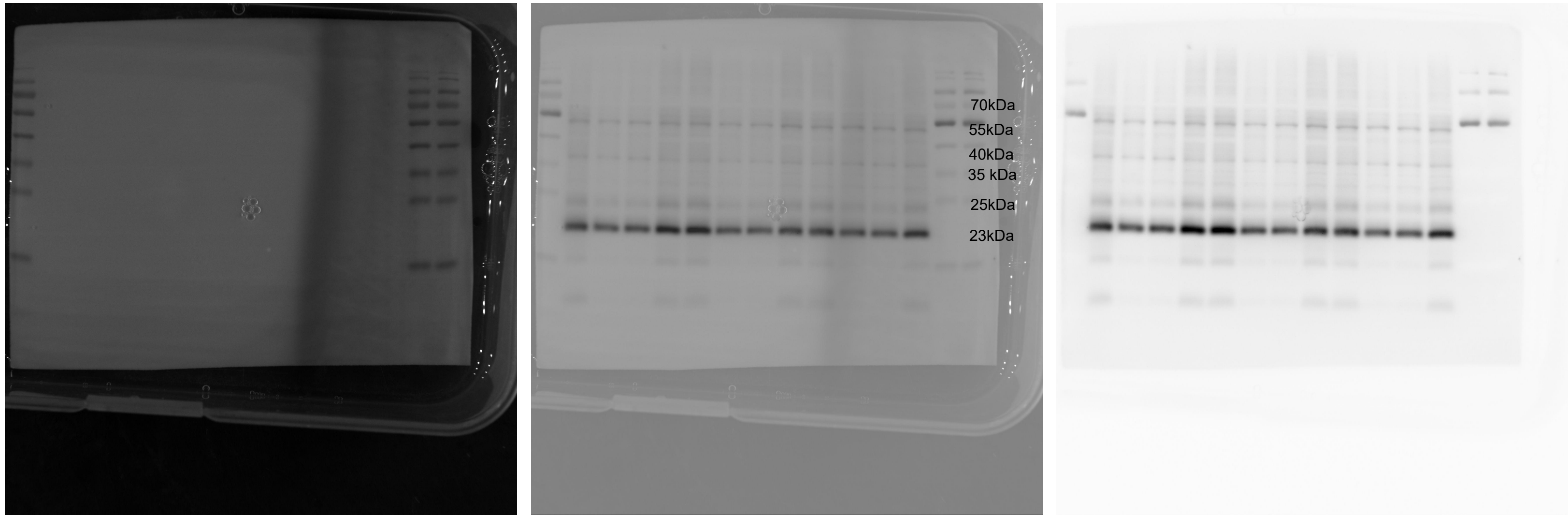

β-Actin

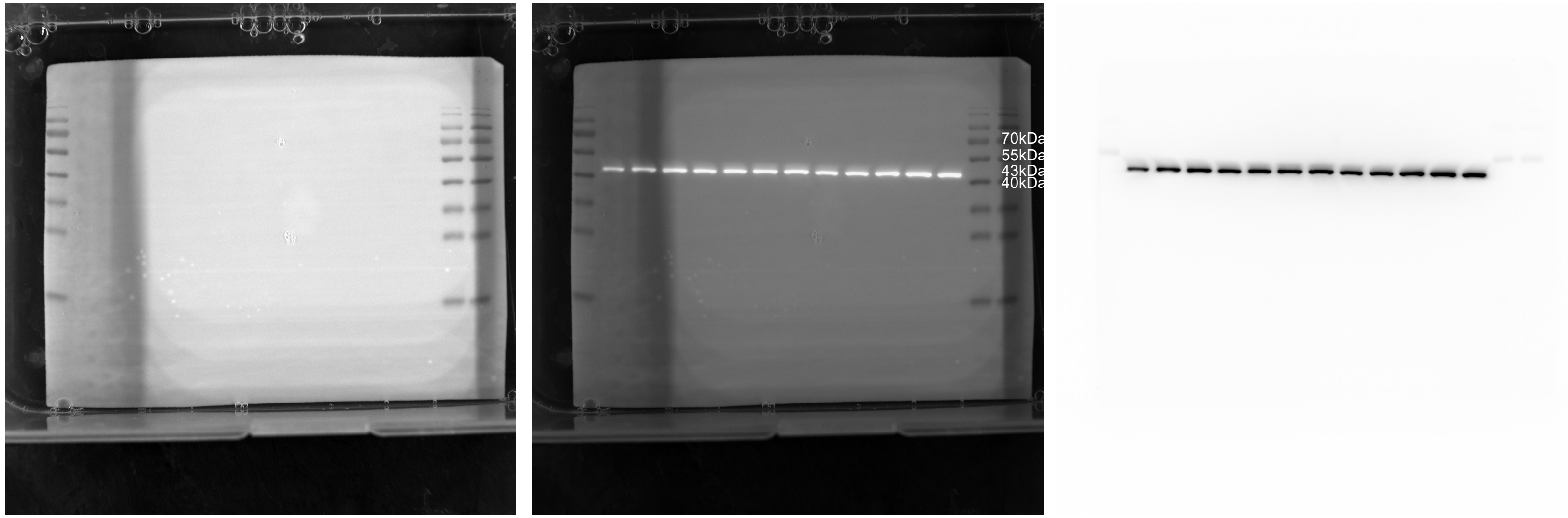

Mouse; Occludin; Repeat*6
Group:Sham, ICH, M0-Evs, M2-Evs
Figure. 4K

Marker: ThermoFisher 26616

Occludin

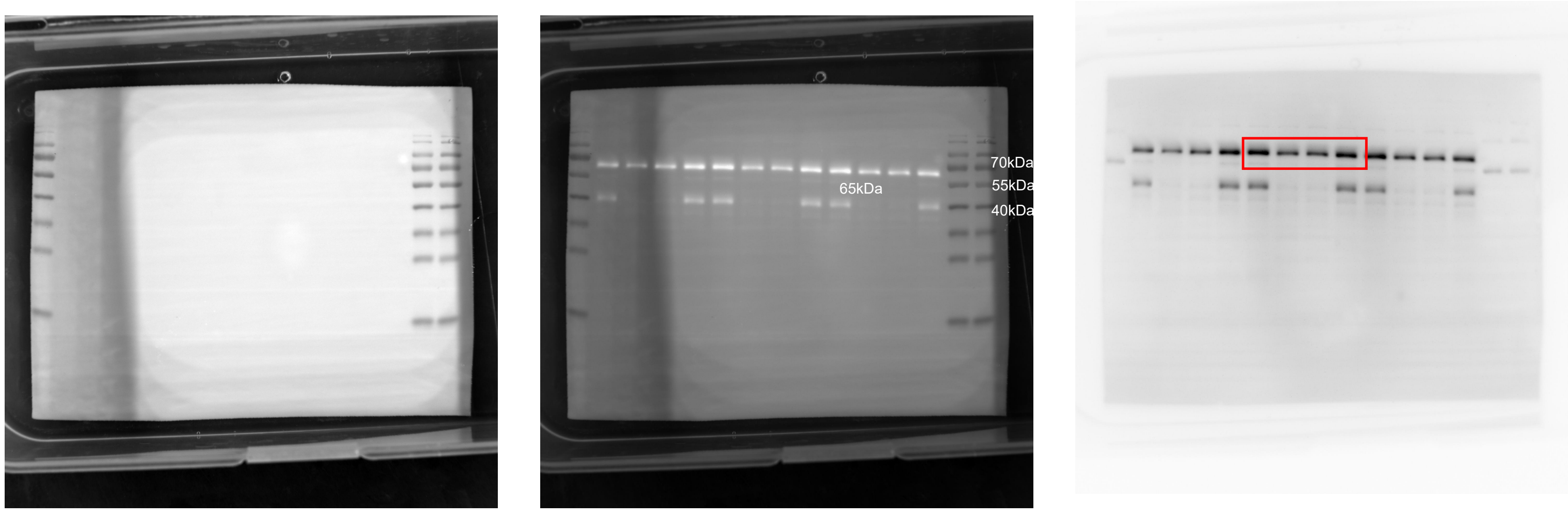

β-Actin

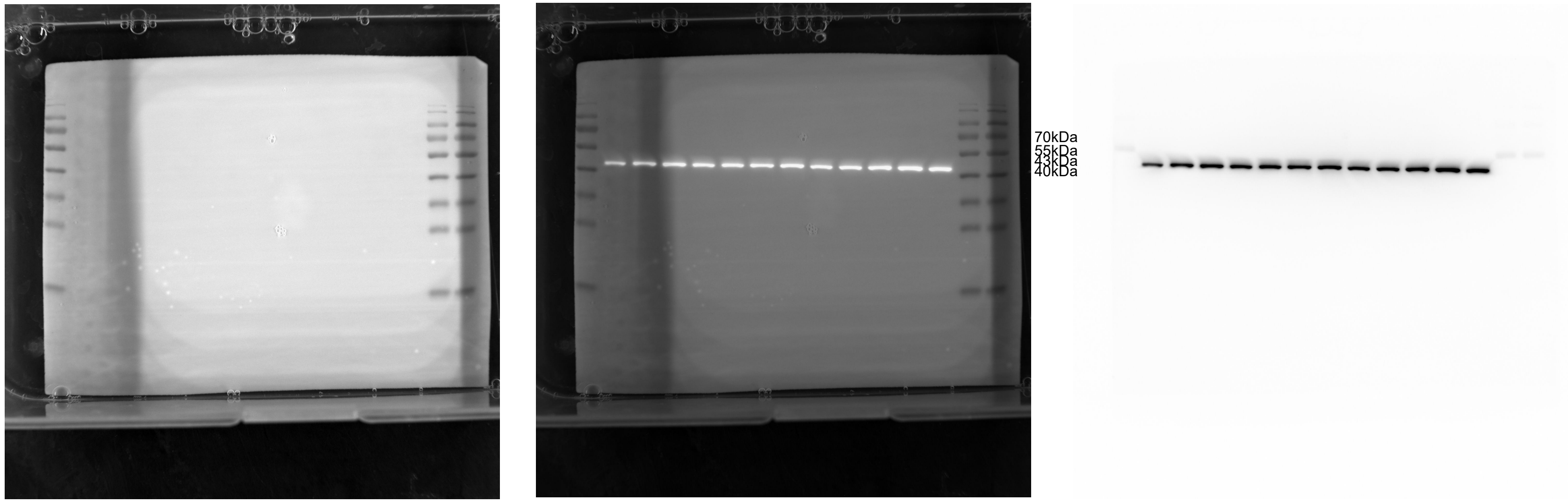

Marker: ThermoFisher 26616

Occludin

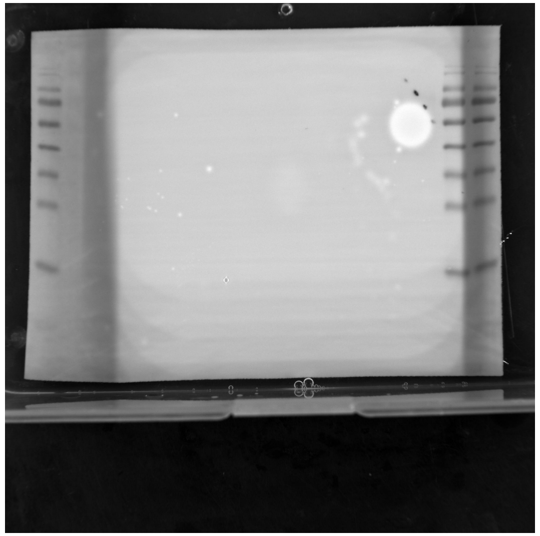

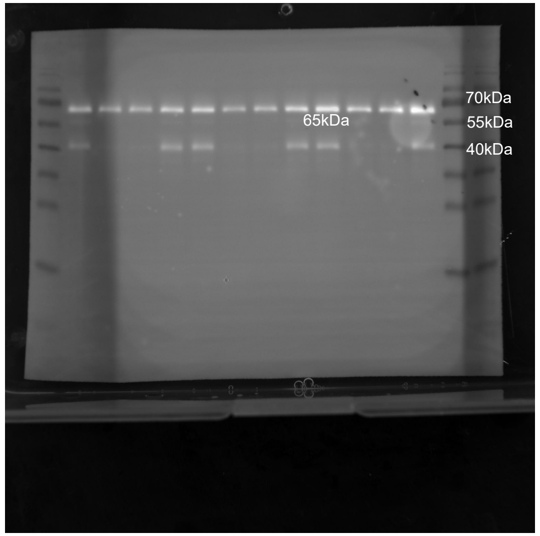

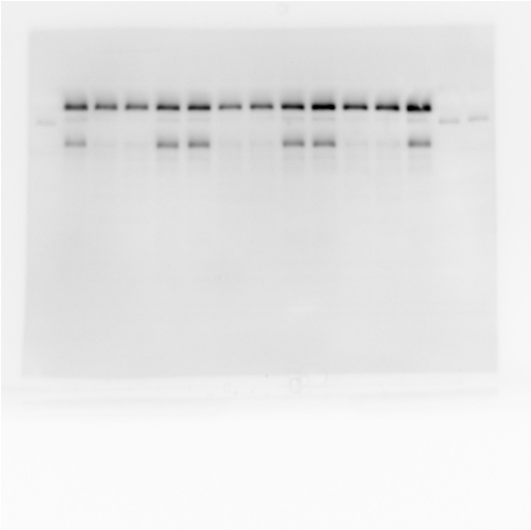

β-Actin

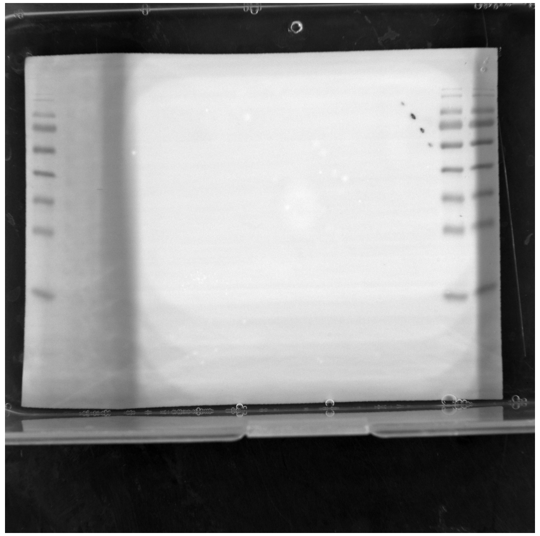

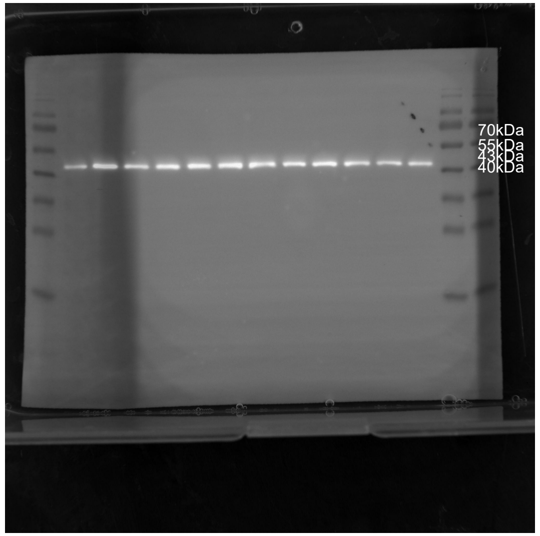

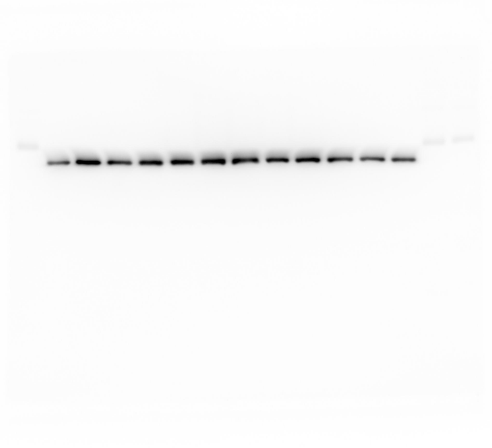

Mouse; VE-Cadherin；Repeat*6

Group:Sham, ICH, M0-Evs, M2-Evs

Figure. 4K

Marker: ThermoFisher 26616

VE-Cadhrein

β-Actin

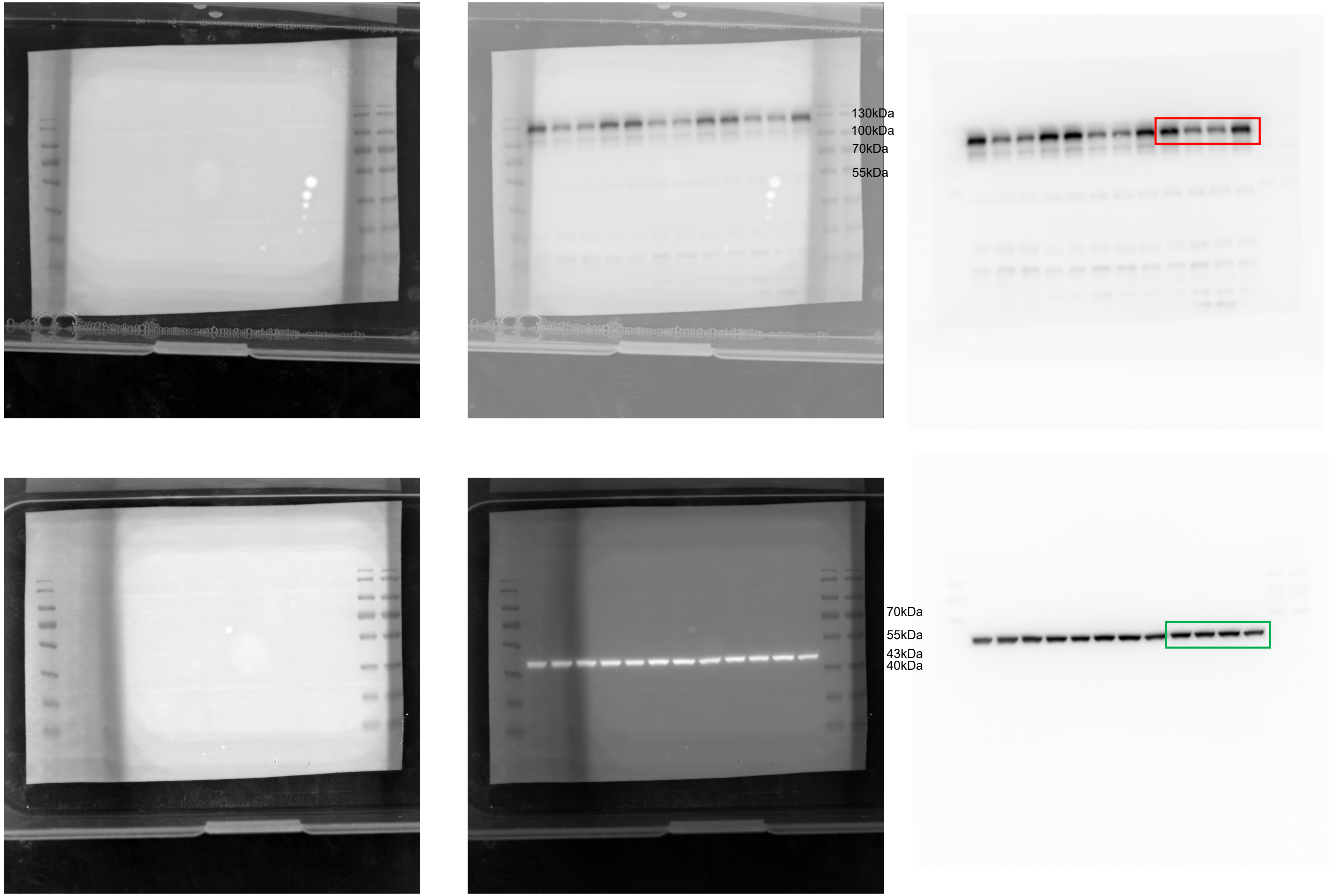

Marker: ThermoFisher 26616

VE-Cadhrein

β-Actin

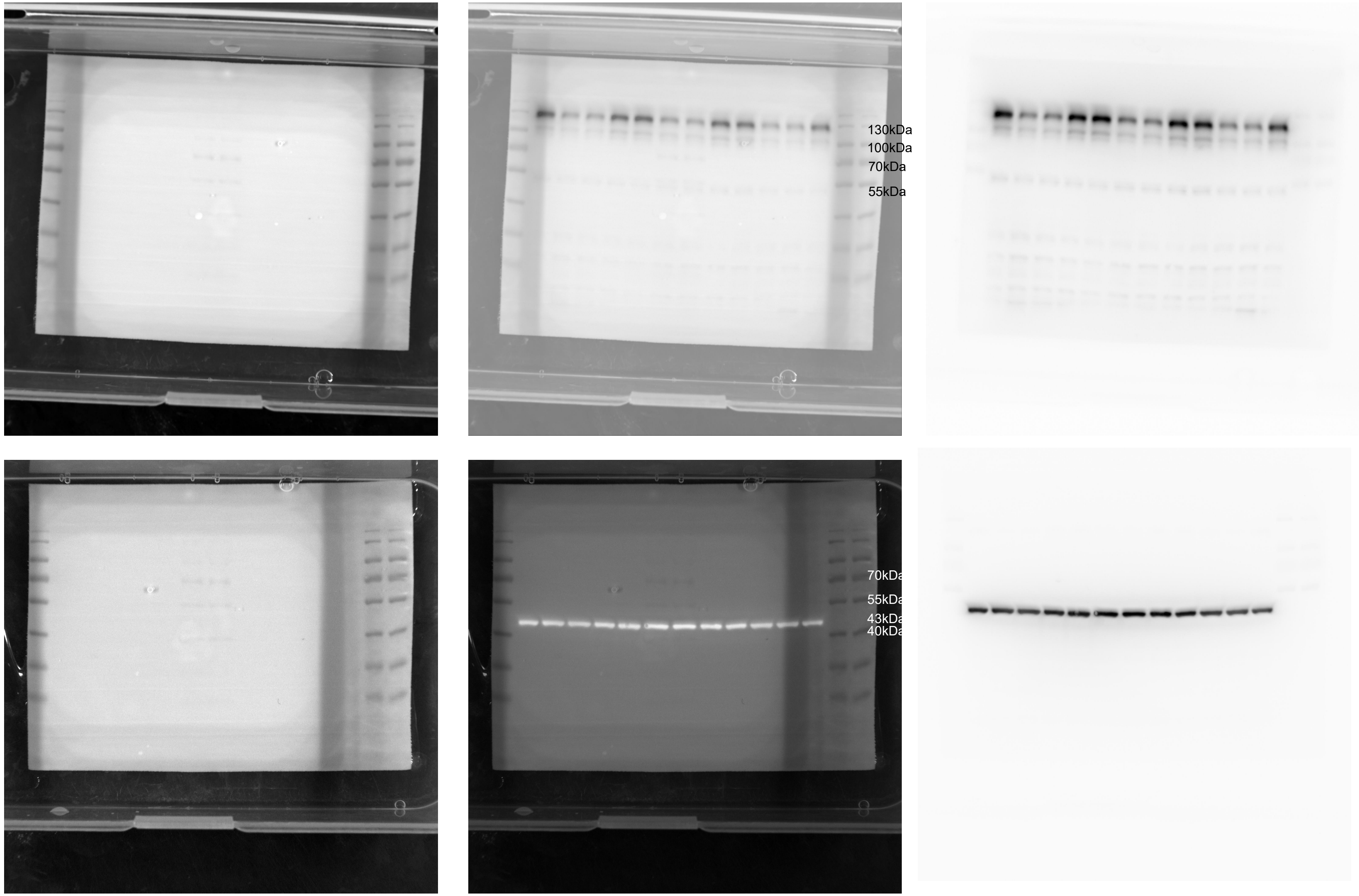

Cell; Caspase-3；Repeat*3
Group:Sham, Hemin, M2-Evs, miR-27n-3p cn, miR-27b-3p k/d
Figure. 6E

Marker: ThermoFisher 26616

Caspase-3

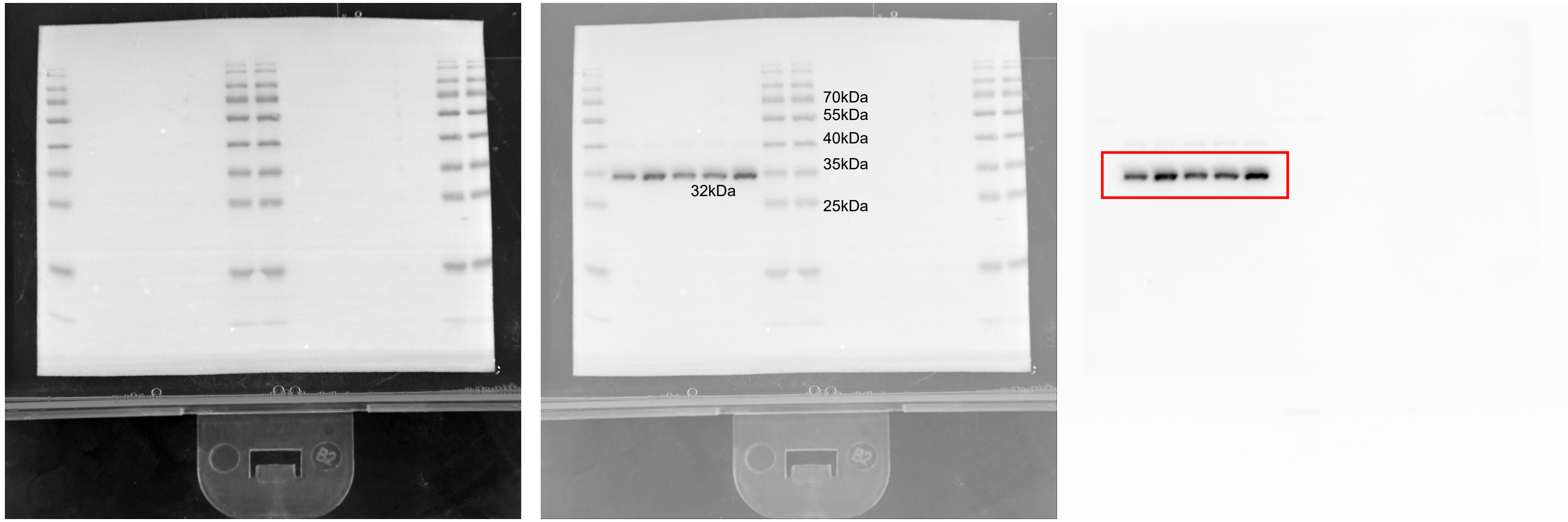

β-Actin

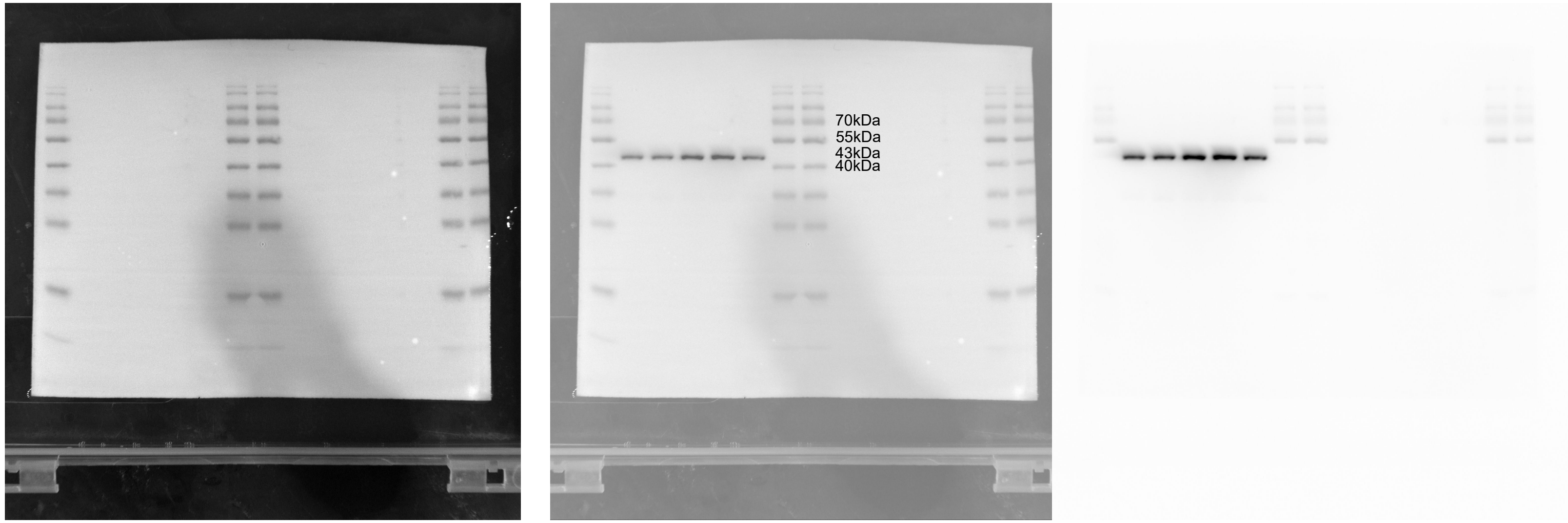

Marker: ThermoFisher 26616

Caspase-3

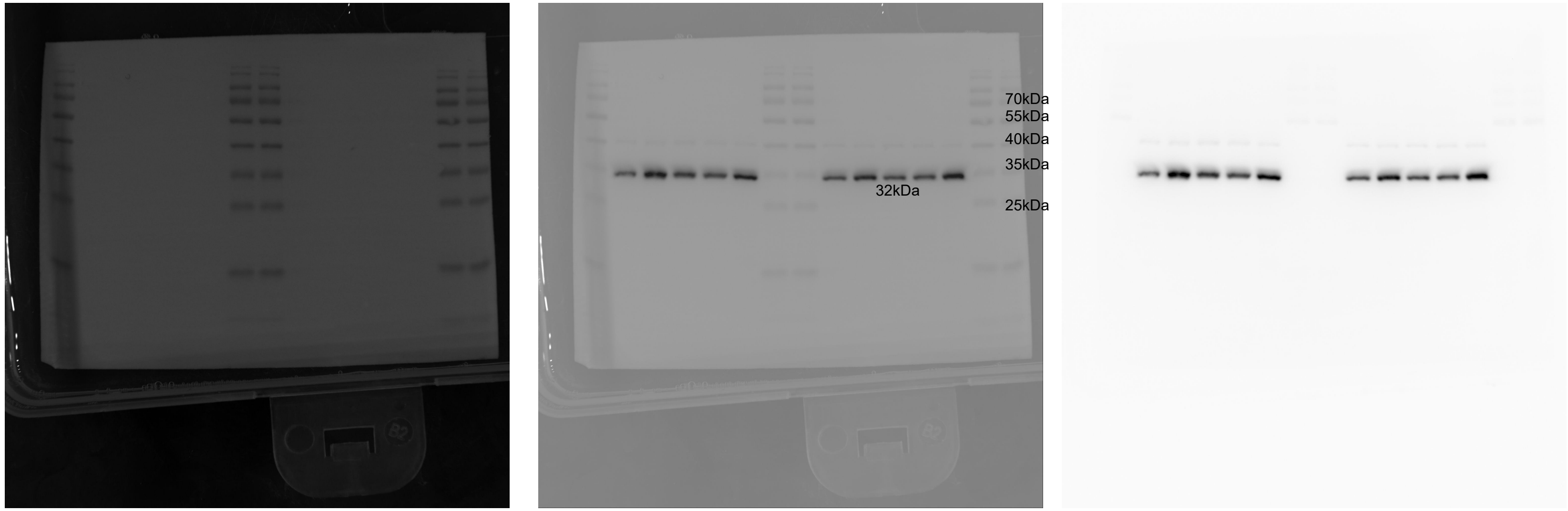

β-Actin

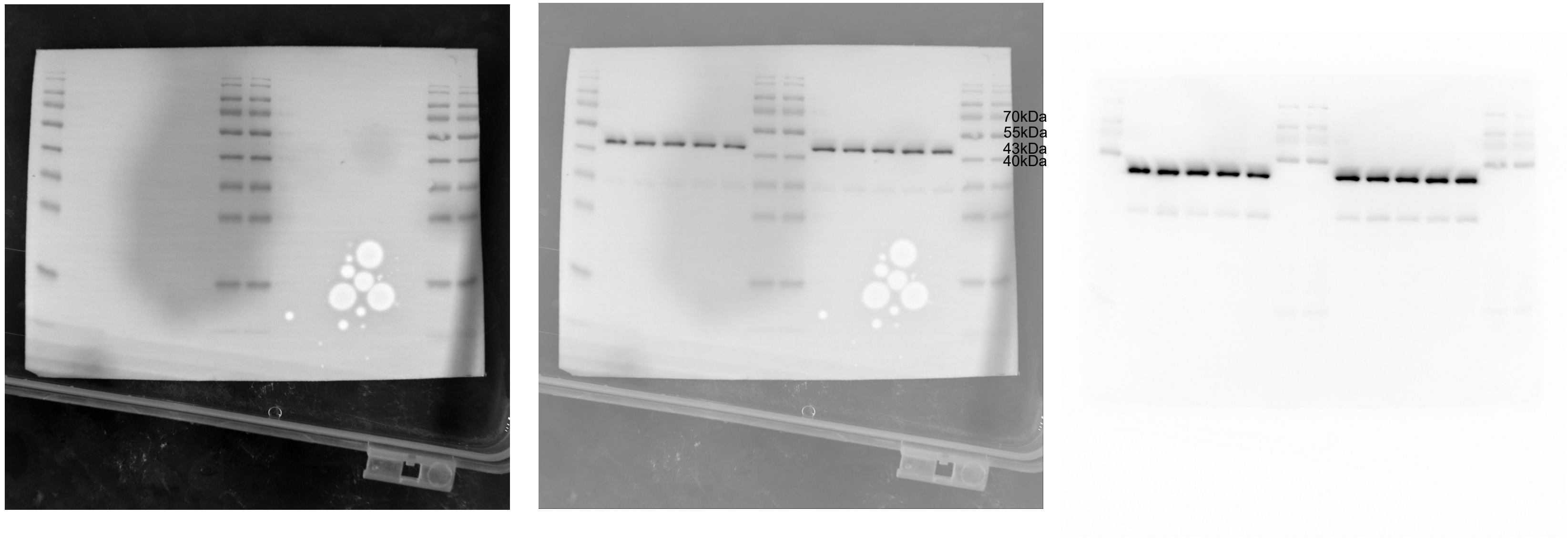

Cell; Bax；Repeat*3

Marker: ThermoFisher 26616

Group:Sham, Hemin, M2-Evs, miR-27n-3p cn, miR-27b-3p k/d

Figure. 6E

Bax

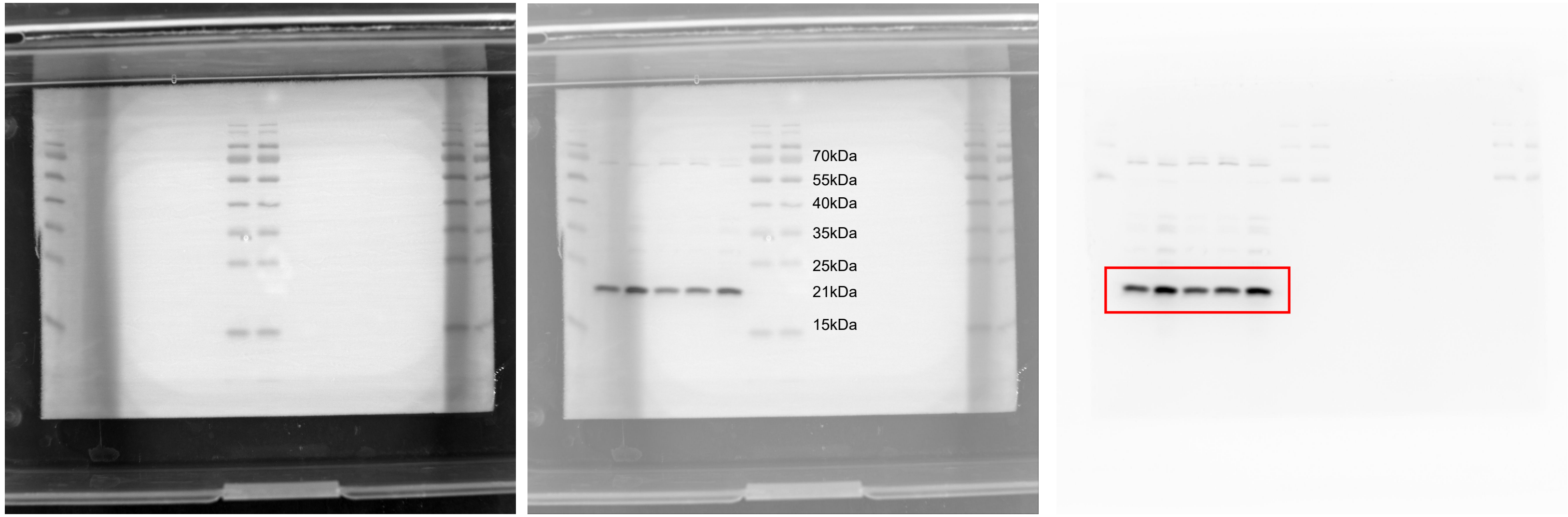

β-Actin

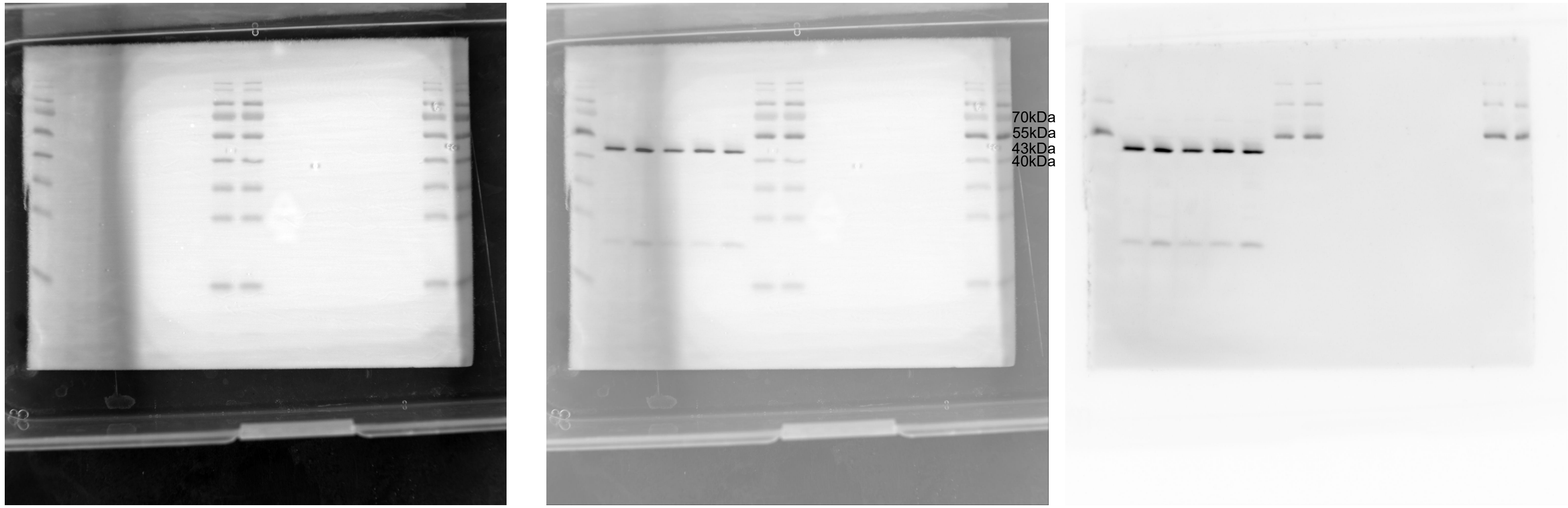

Marker: ThermoFisher 26616

Bax

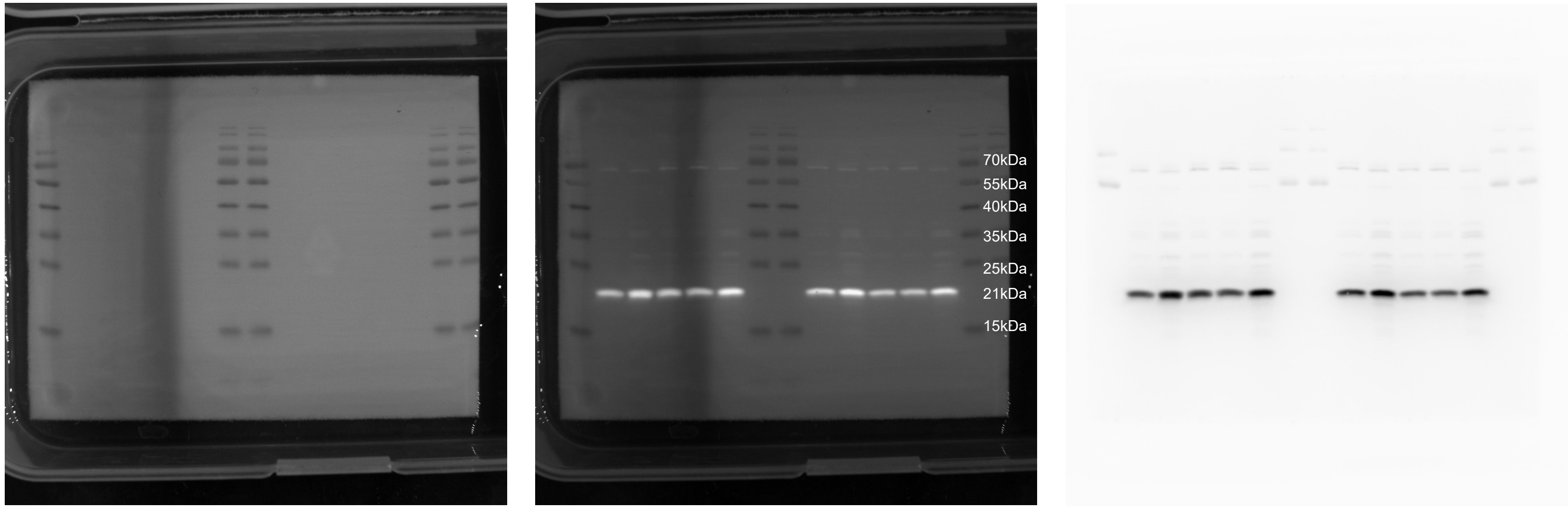

β-Actin

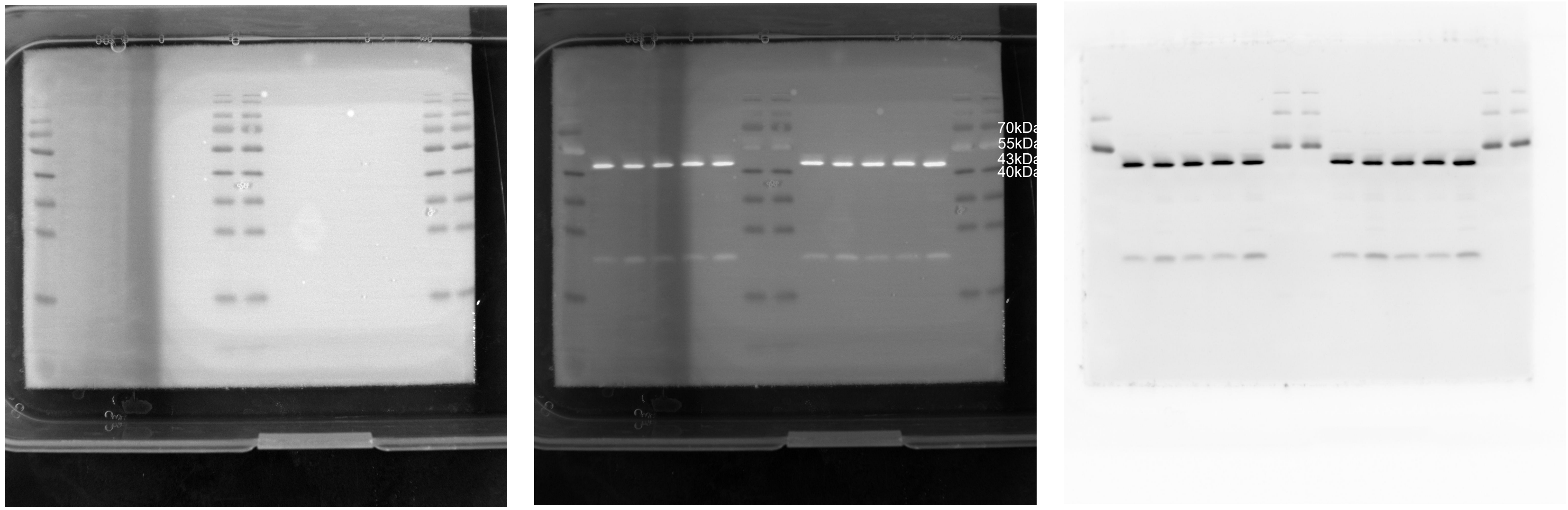

Cell; Bcl-2；Repeat*3

Group:Sham, Hemin, M2-Evs, miR-27n-3p cn, miR-27b-3p k/d

Figure. 6E

Marker: ThermoFisher 26616

Bcl-2

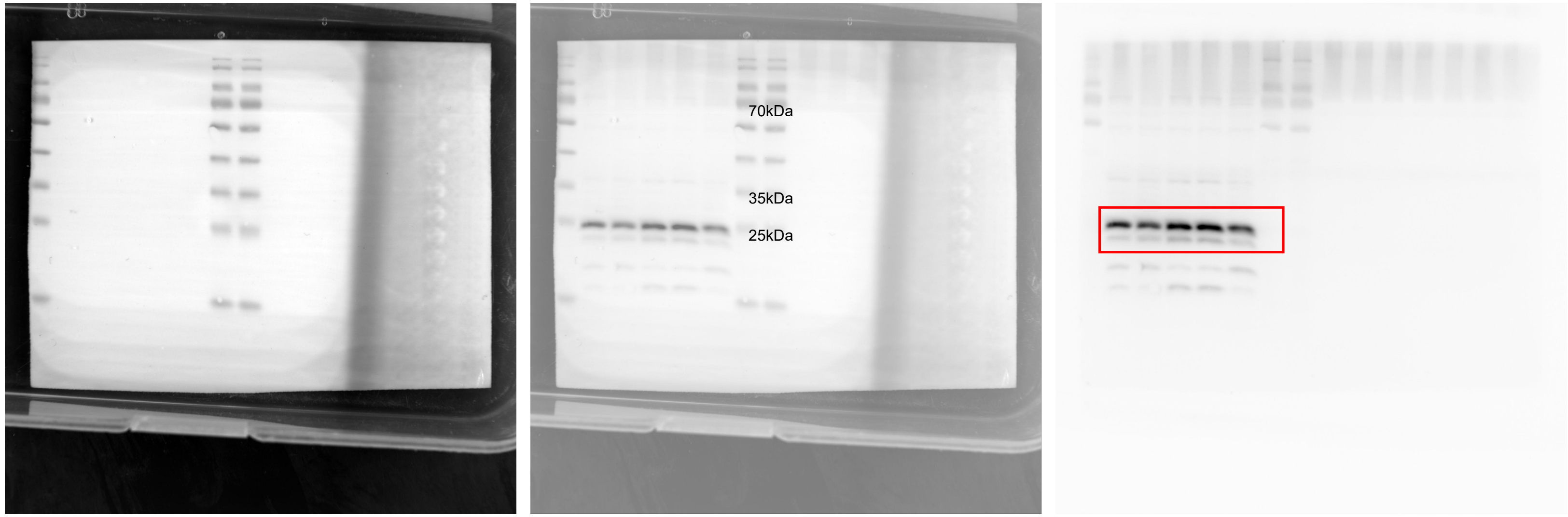

β-Actin

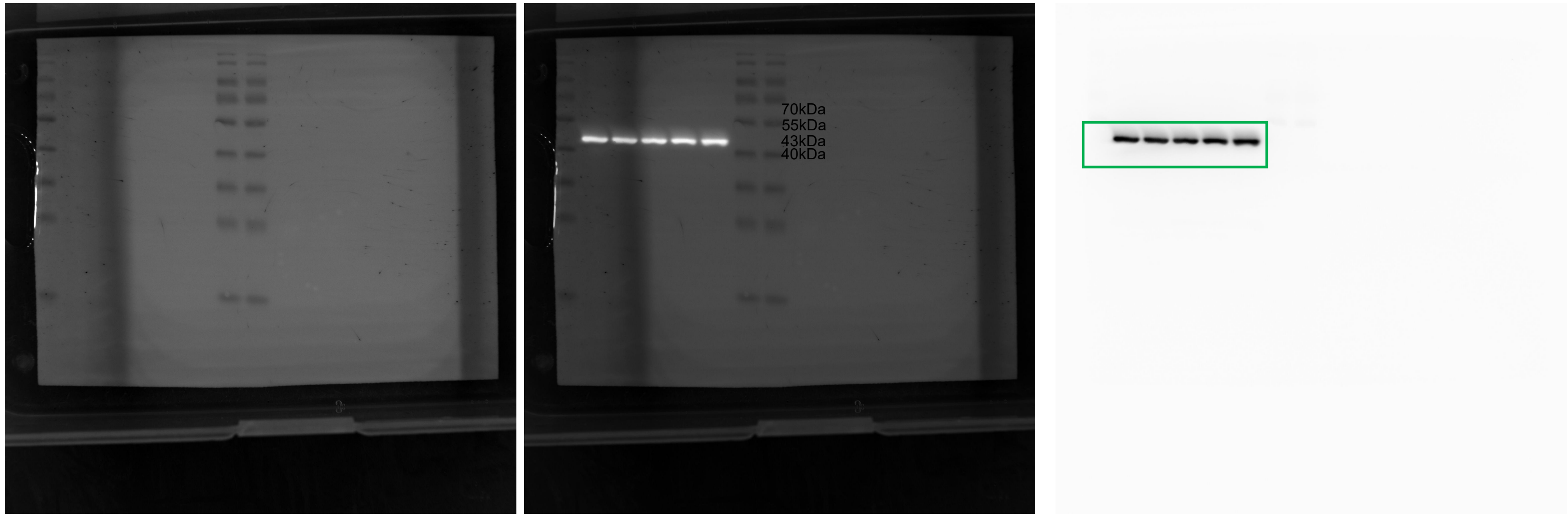

Marker: ThermoFisher 26616

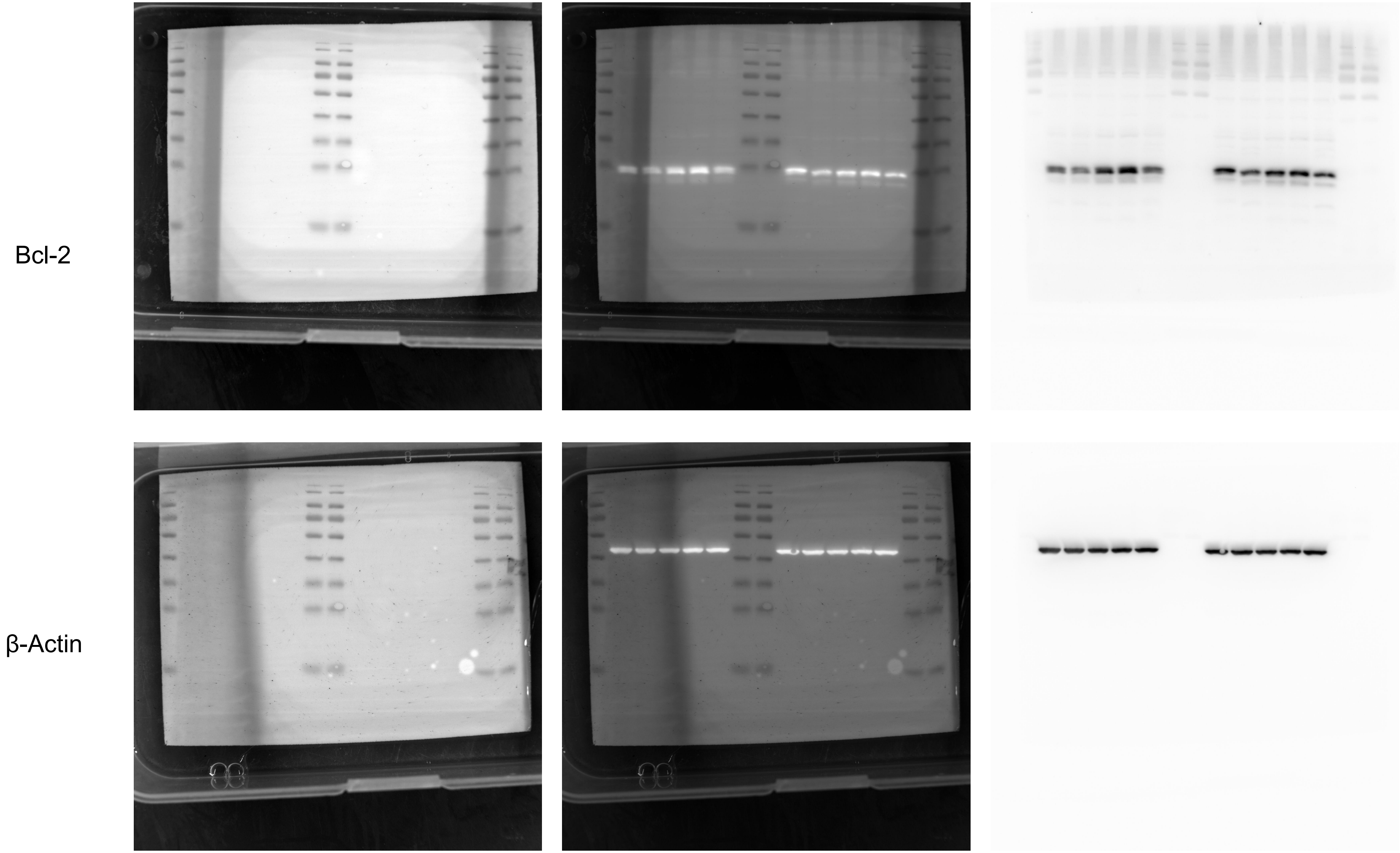

Cell; Cyt-C； Repeat*3

Group:Sham, Hemin, M2-Evs, miR-27n-3p cn, miR-27b-3p k/d

Figure. 6E

Marker: ThermoFisher 26616

Cyt-C

β-Actin

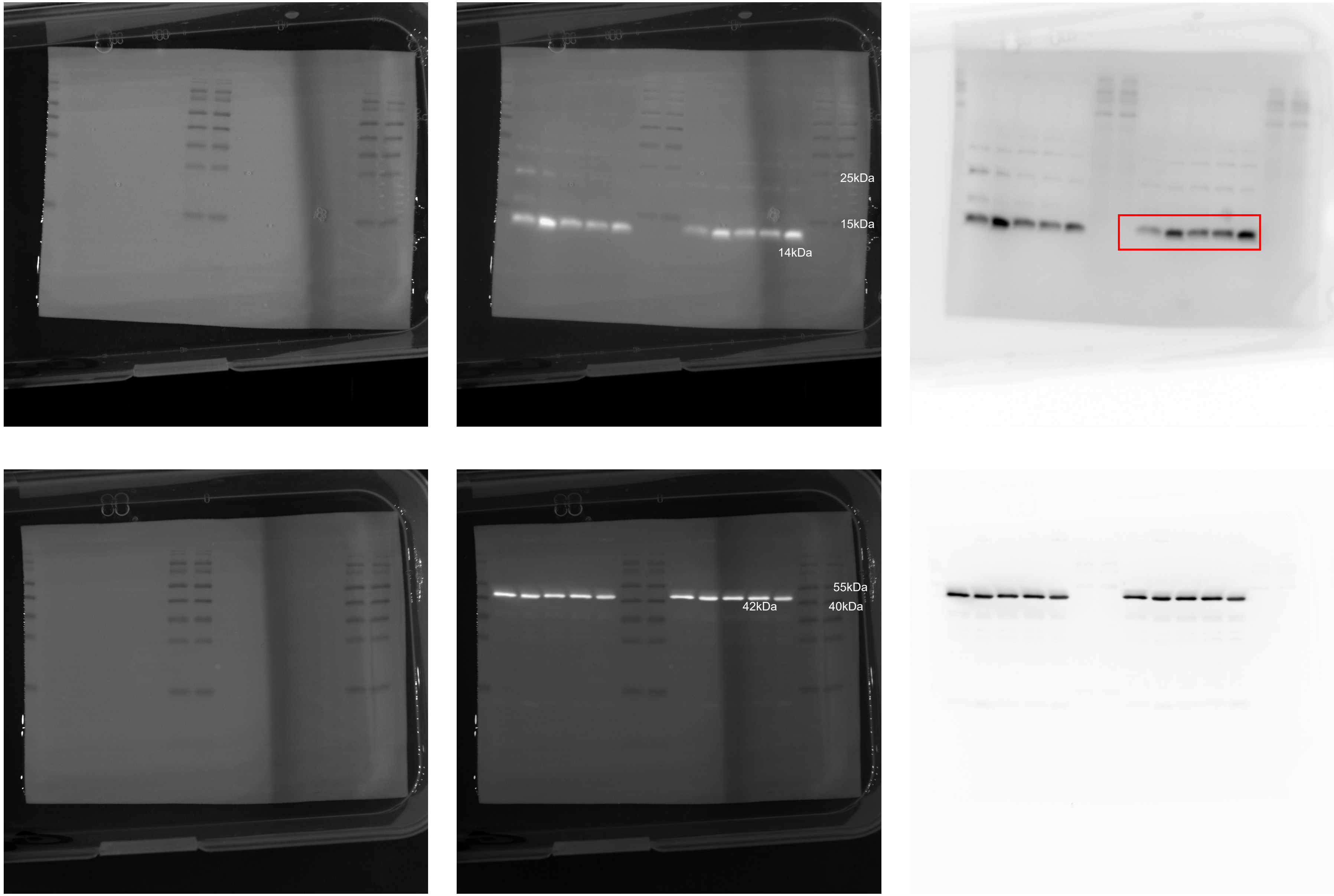

Marker: ThermoFisher 26616

Cyt-C

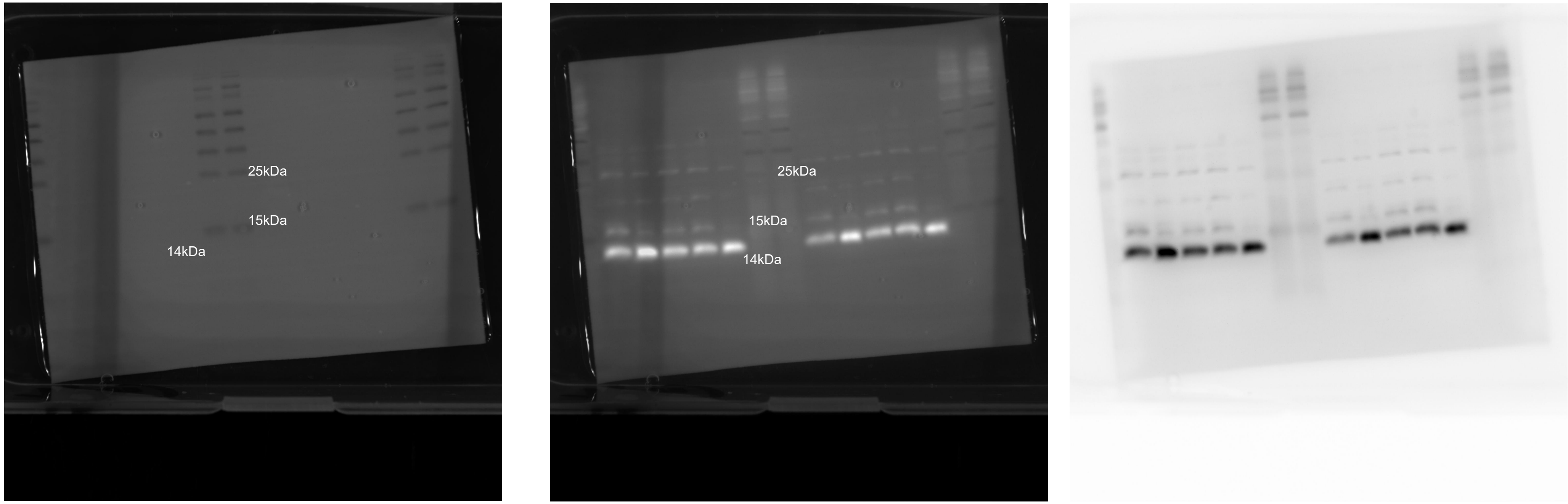

β-Actin

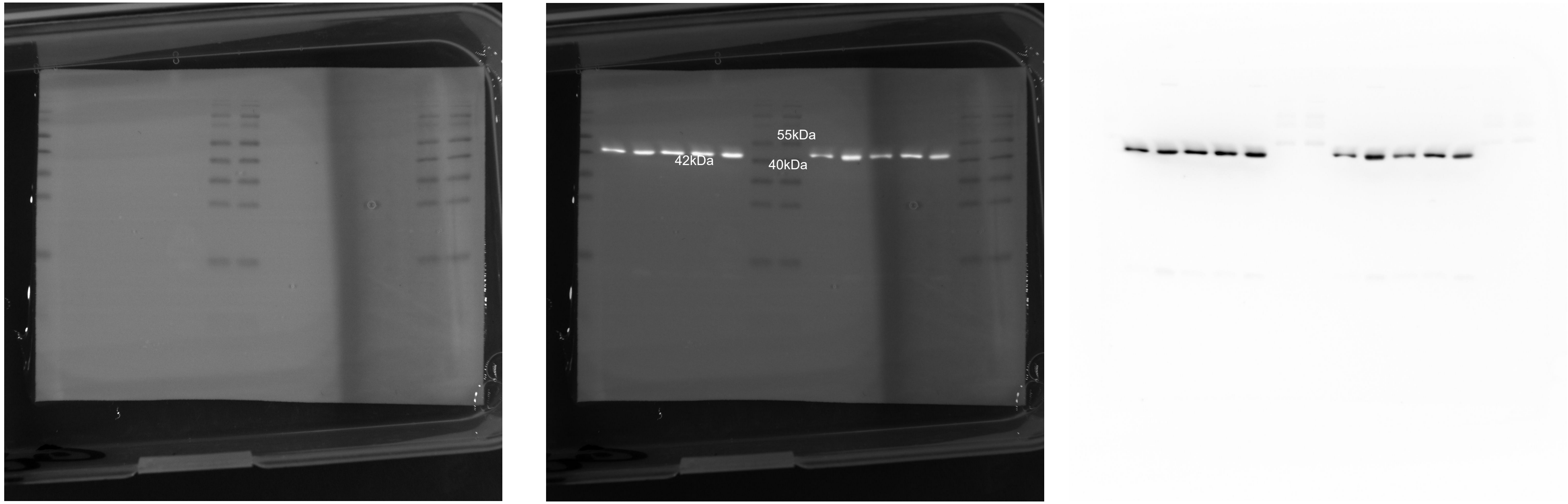

Cell; Claudin 5; Repeat*3

Group:Sham, Hemin, M2-Evs, miR-27n-3p cn, miR-27b-3p k/d

Figure. 6L

Marker: ThermoFisher 26616

Claudin 5

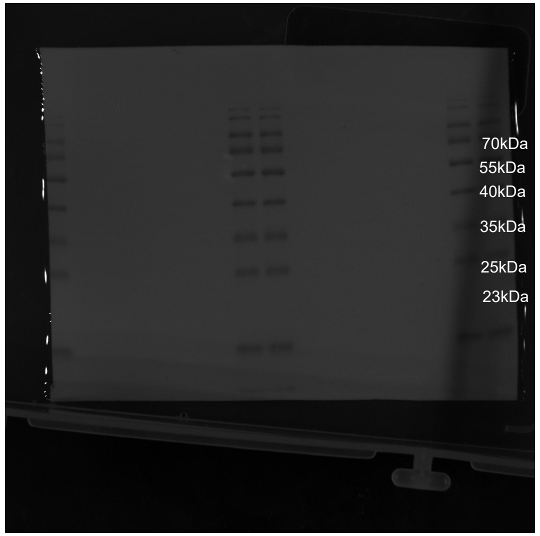

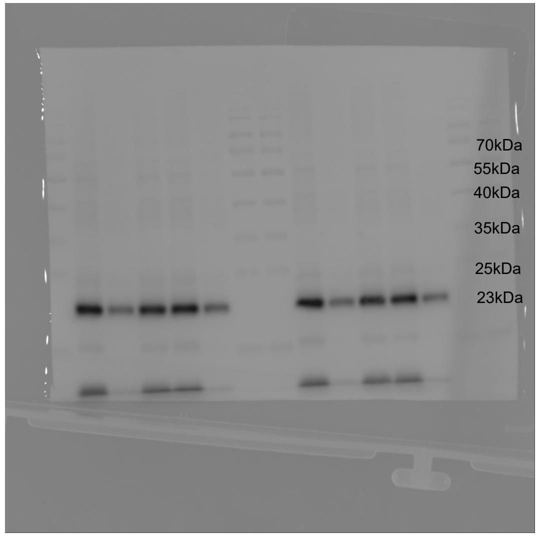

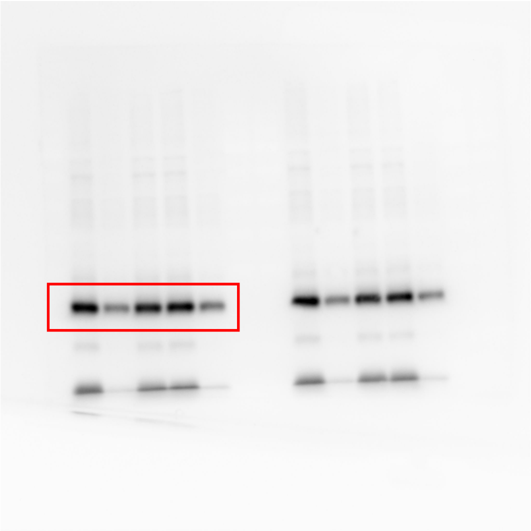

β-Actin

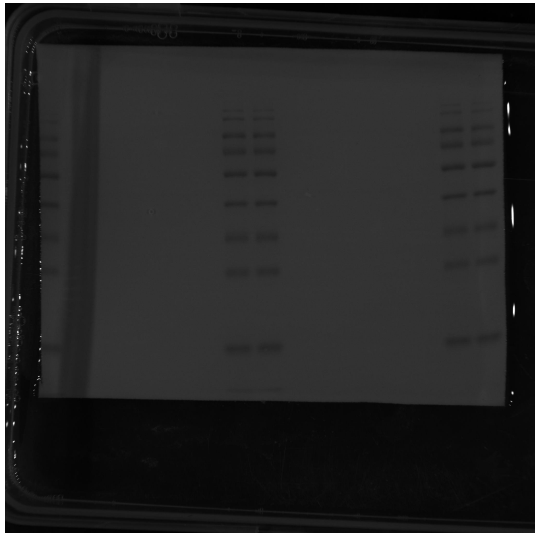

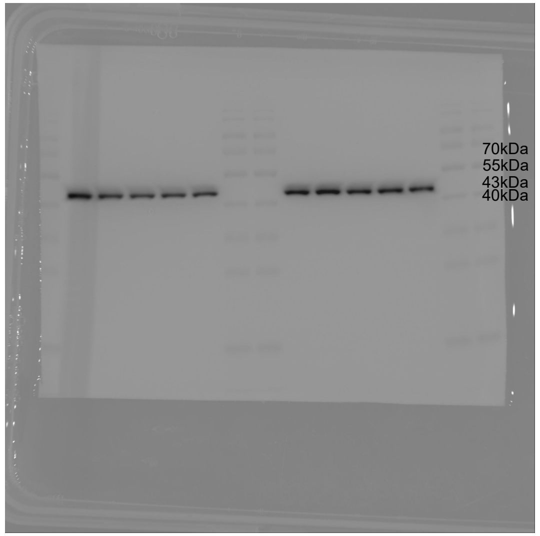

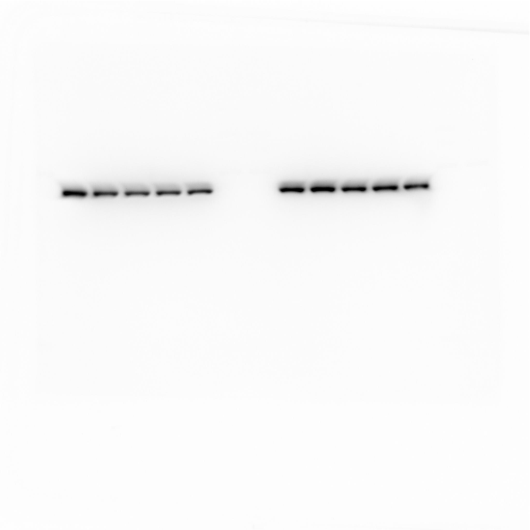

Marker: ThermoFisher 26616

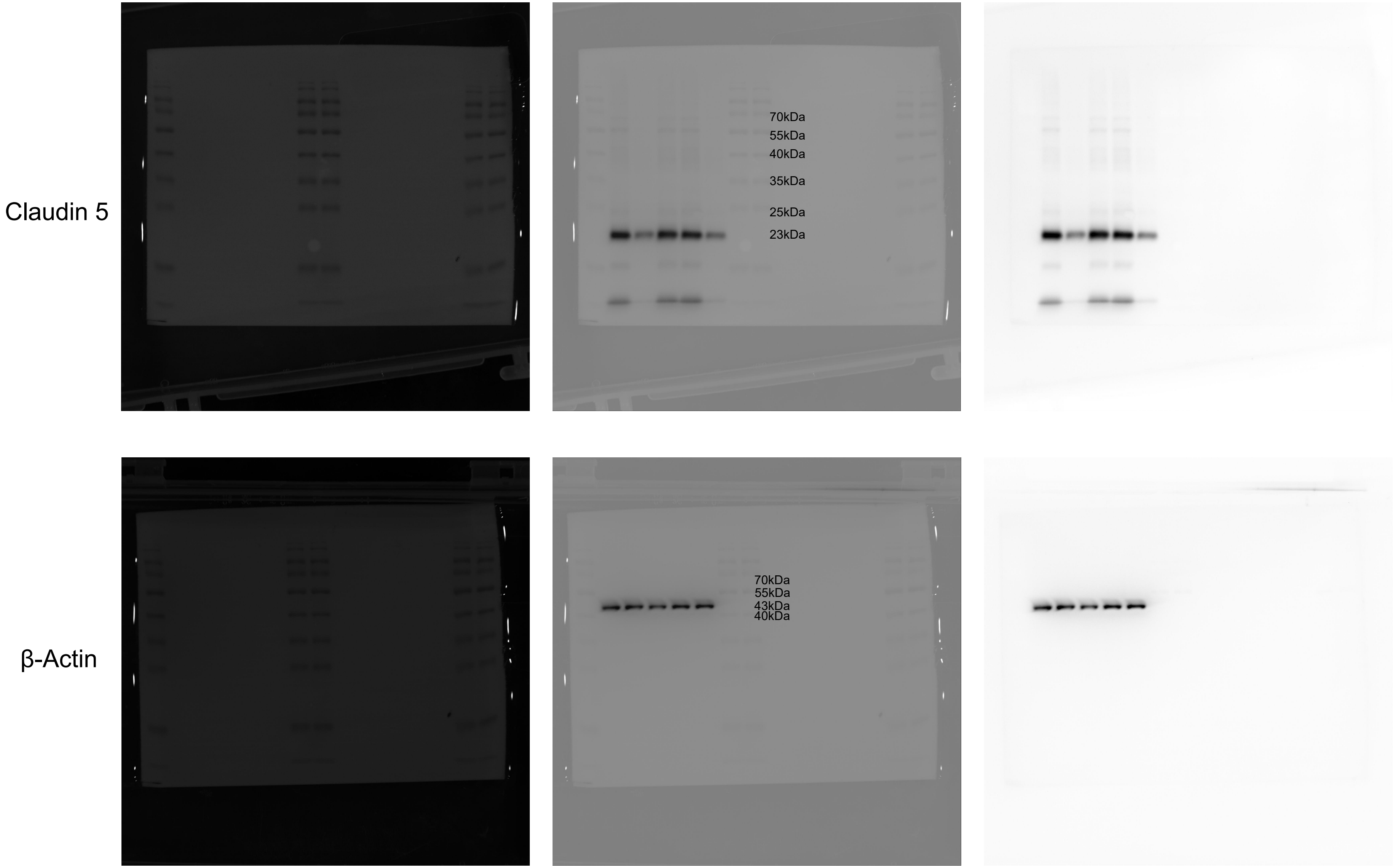

Cell; Occludin； Repeat*3

Marker: ThermoFisher 26616

Group:Sham, Hemin, M2-Evs, miR-27n-3p cn, miR-27b-3p k/d

Figure. 6L

Occludin

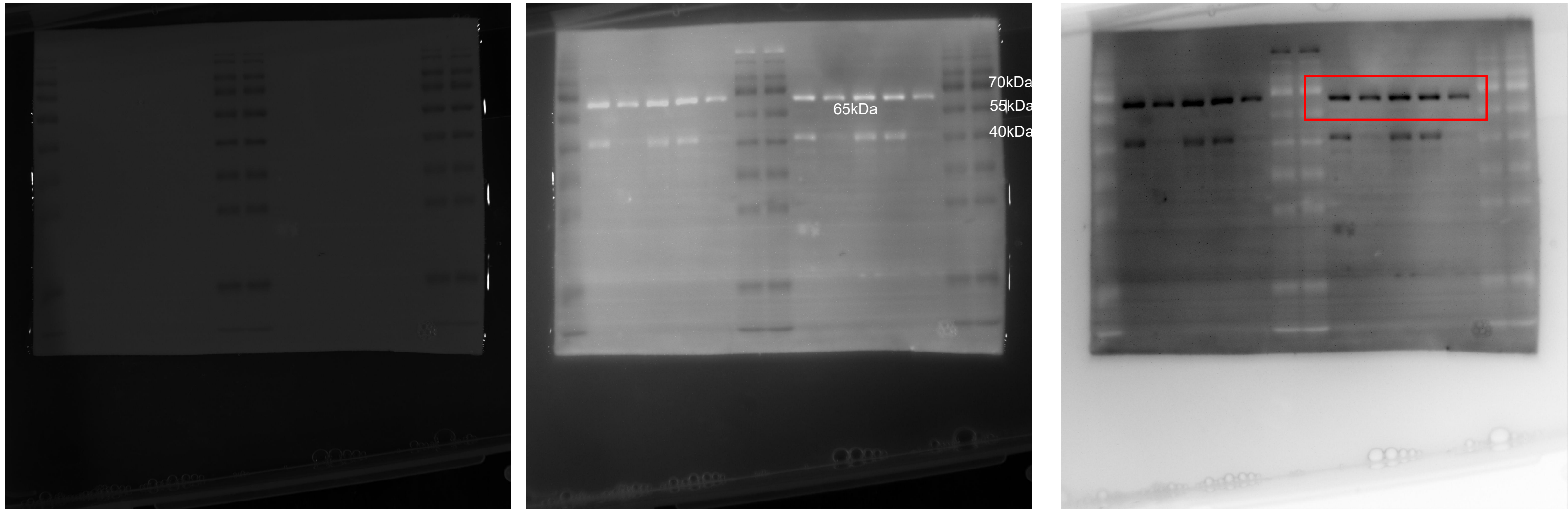

β-Actin

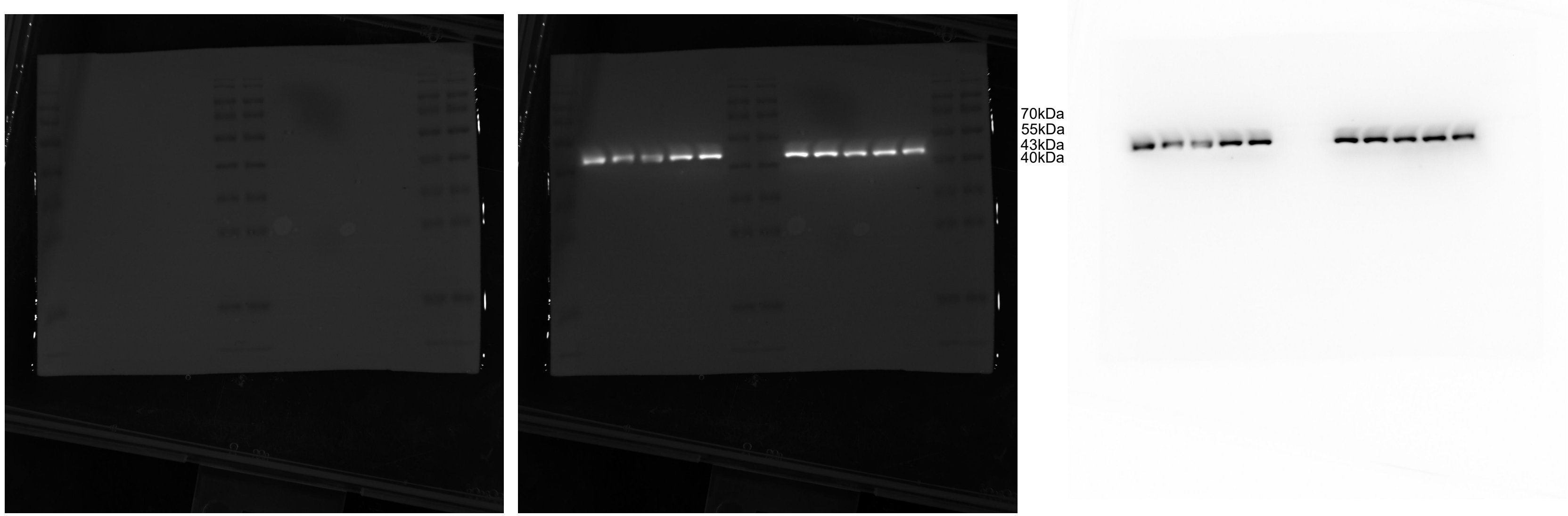

Marker: ThermoFisher 26616

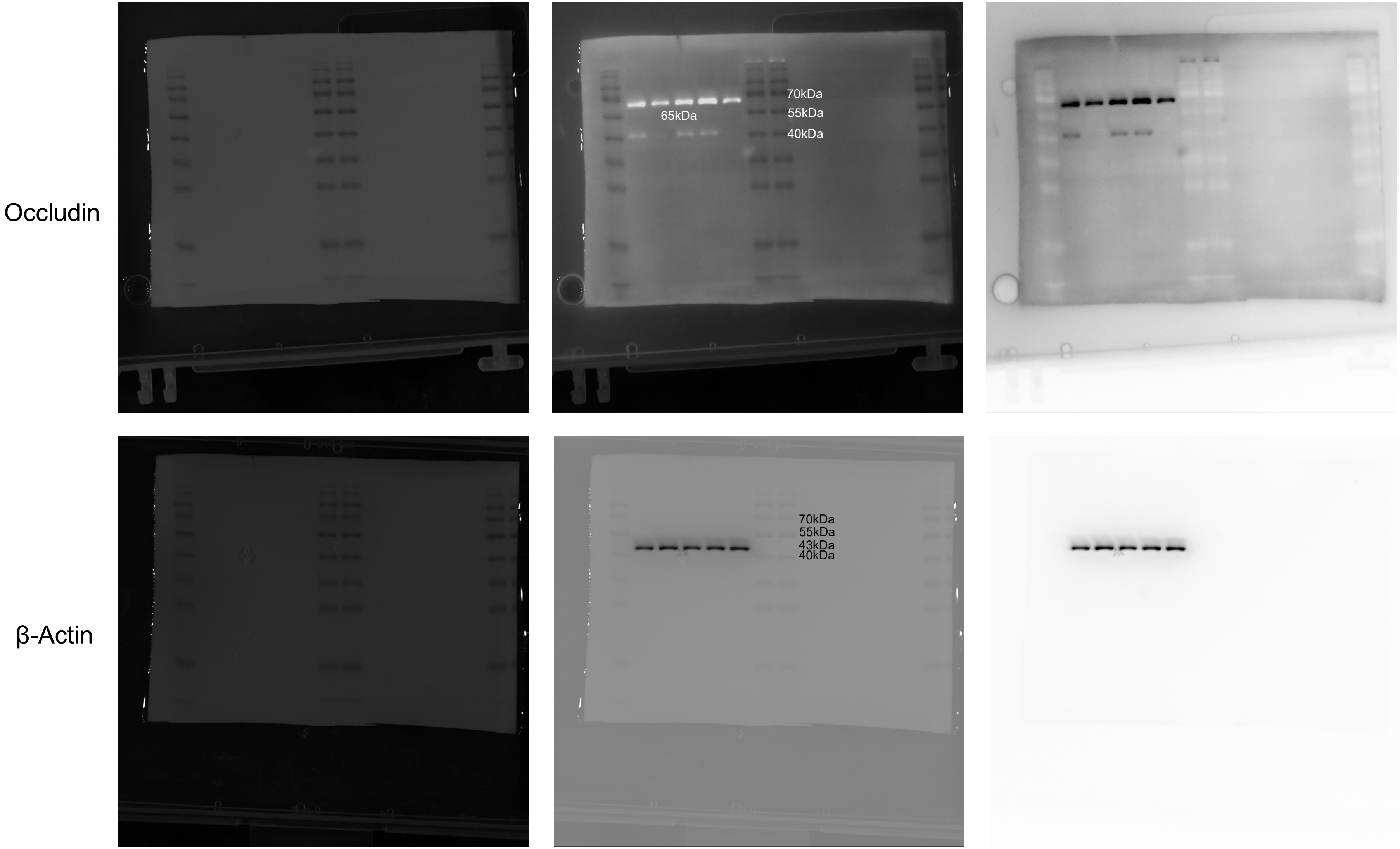

Cell; VE-Cadherin； Repeat*3

Marker: ThermoFisher 26616

Group:Sham, Hemin, M2-Evs, miR-27n-3p cn, miR-27b-3p k/d

Figure. 6L

VE-Cadhrein

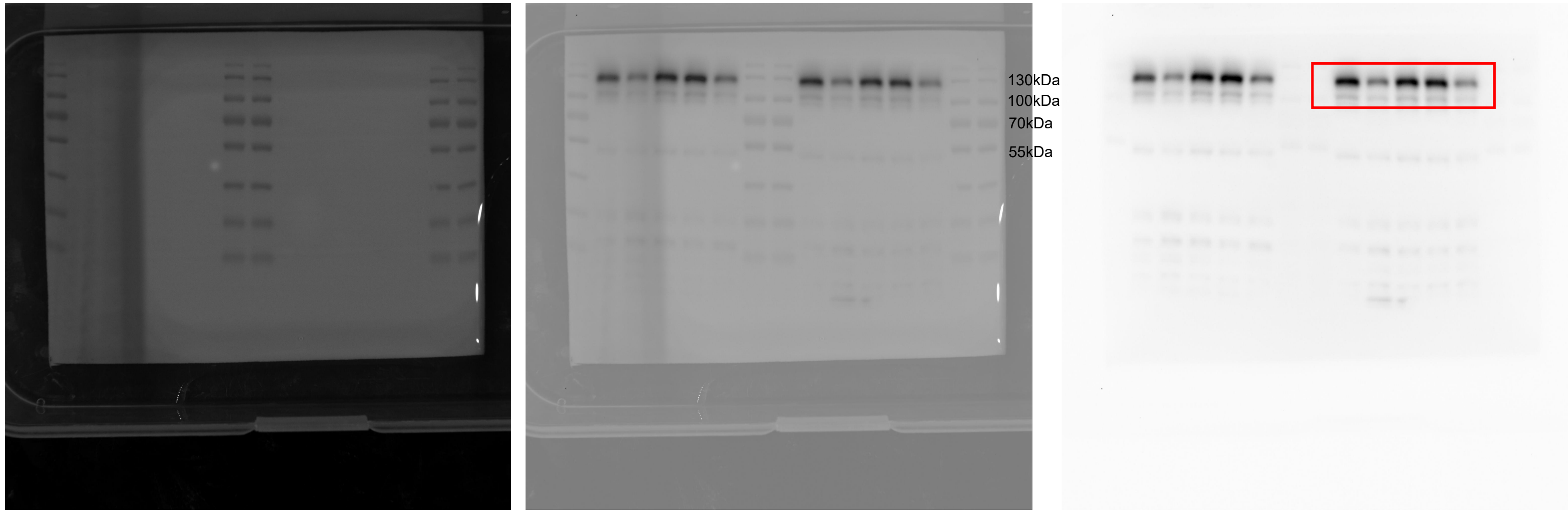

β-Actin

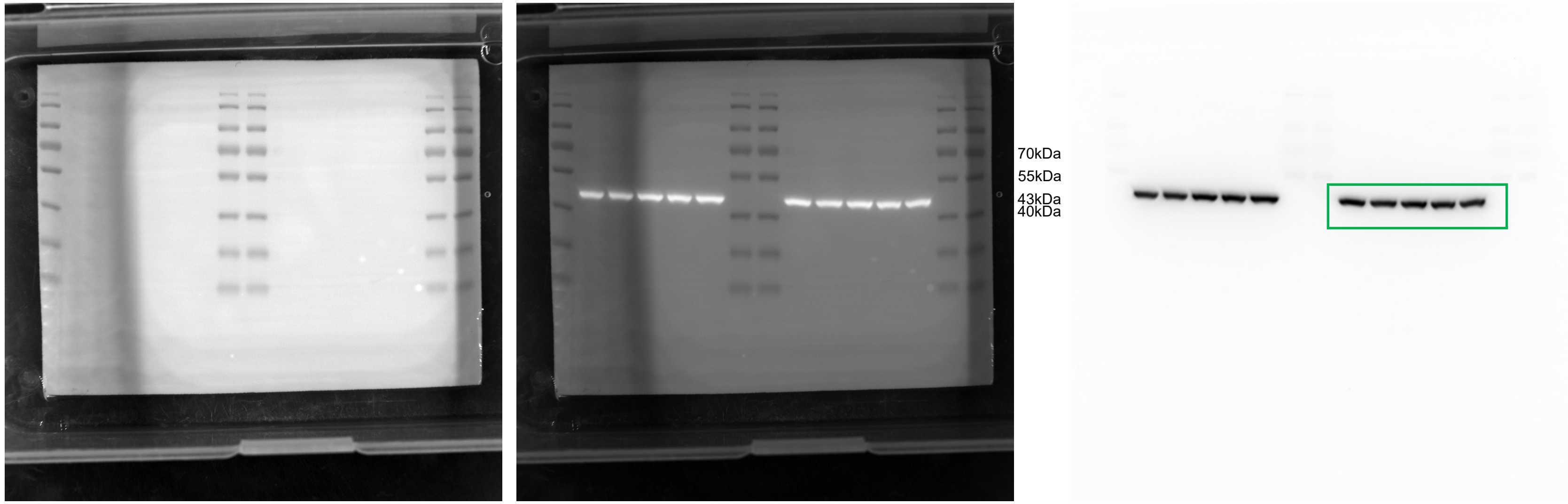

Marker: ThermoFisher 26616

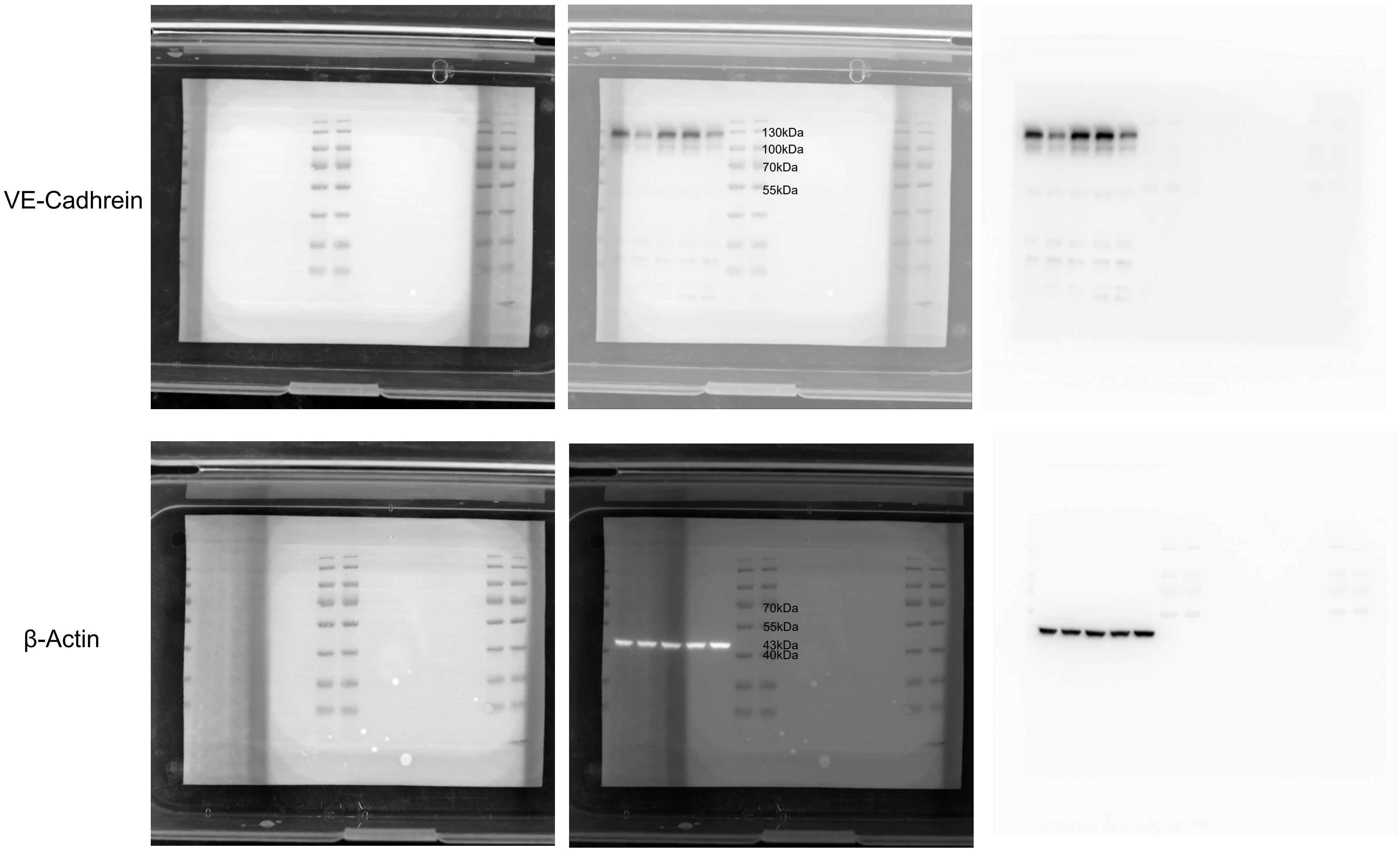

Mouse; Caspase-3; Repeat*6

Marker: ThermoFisher 26616

Group:Sham, ICH, M2-Evs, miR-27n-3p cn, miR-27b-3p k/d

Figure. 8C

Caspase-3

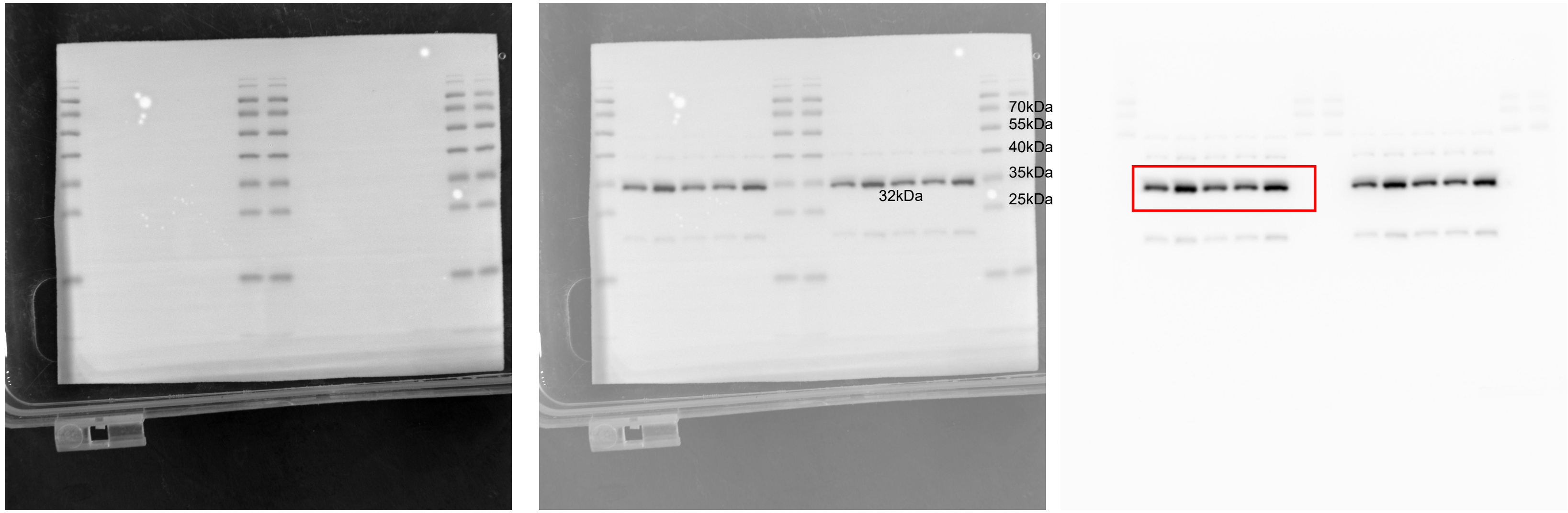

β-Actin

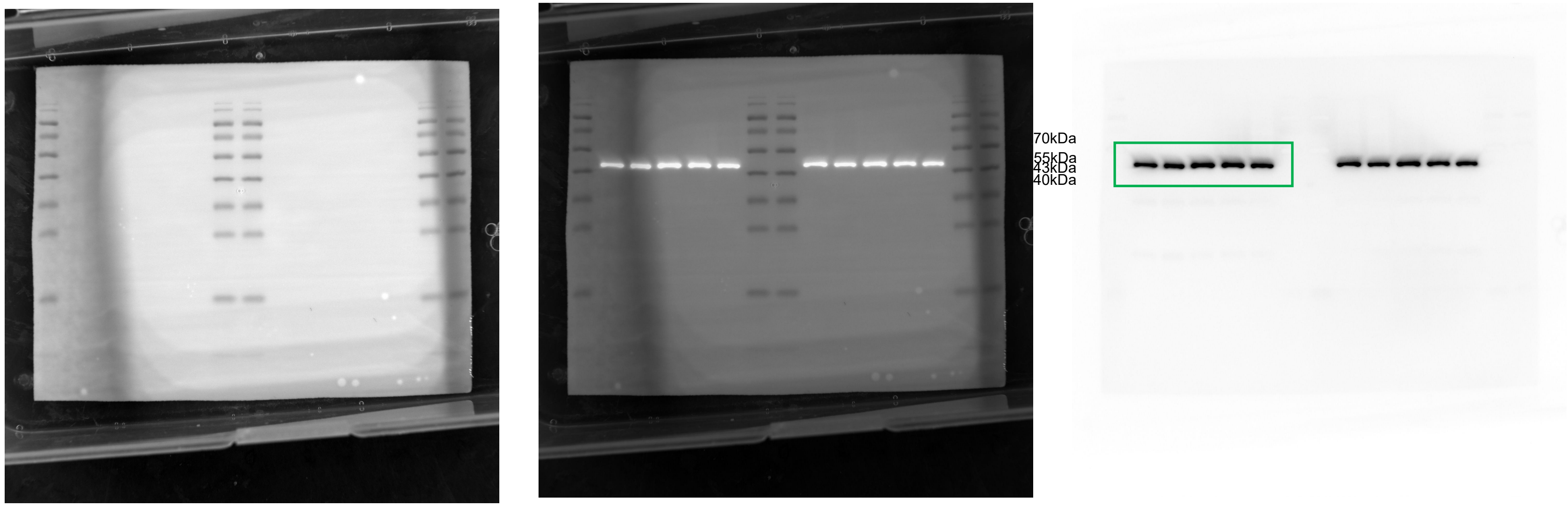

Marker: ThermoFisher 26616

Caspase-3

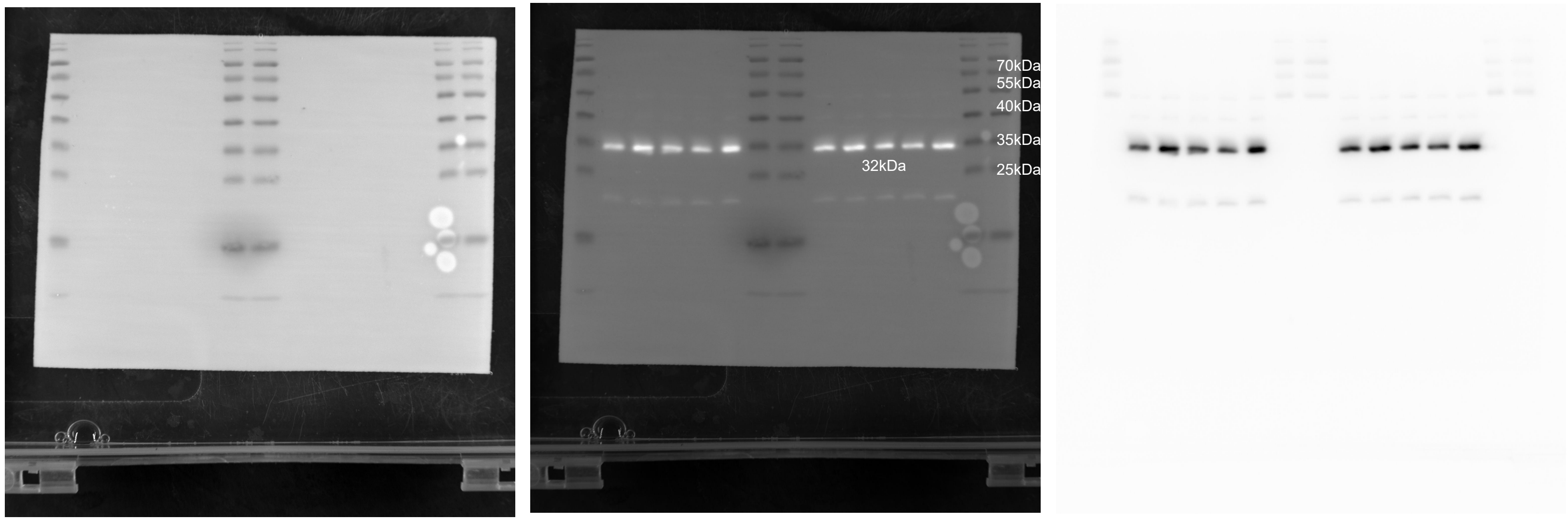

β-Actin

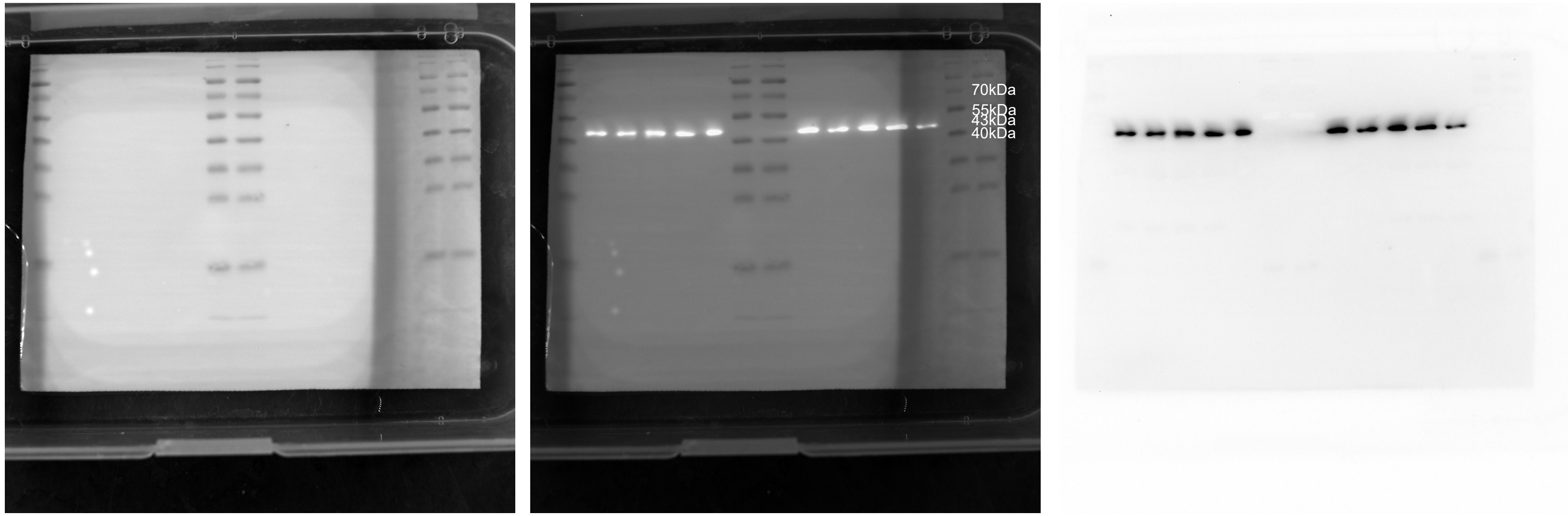

Marker: ThermoFisher 26616

Caspase-3

β-Actin

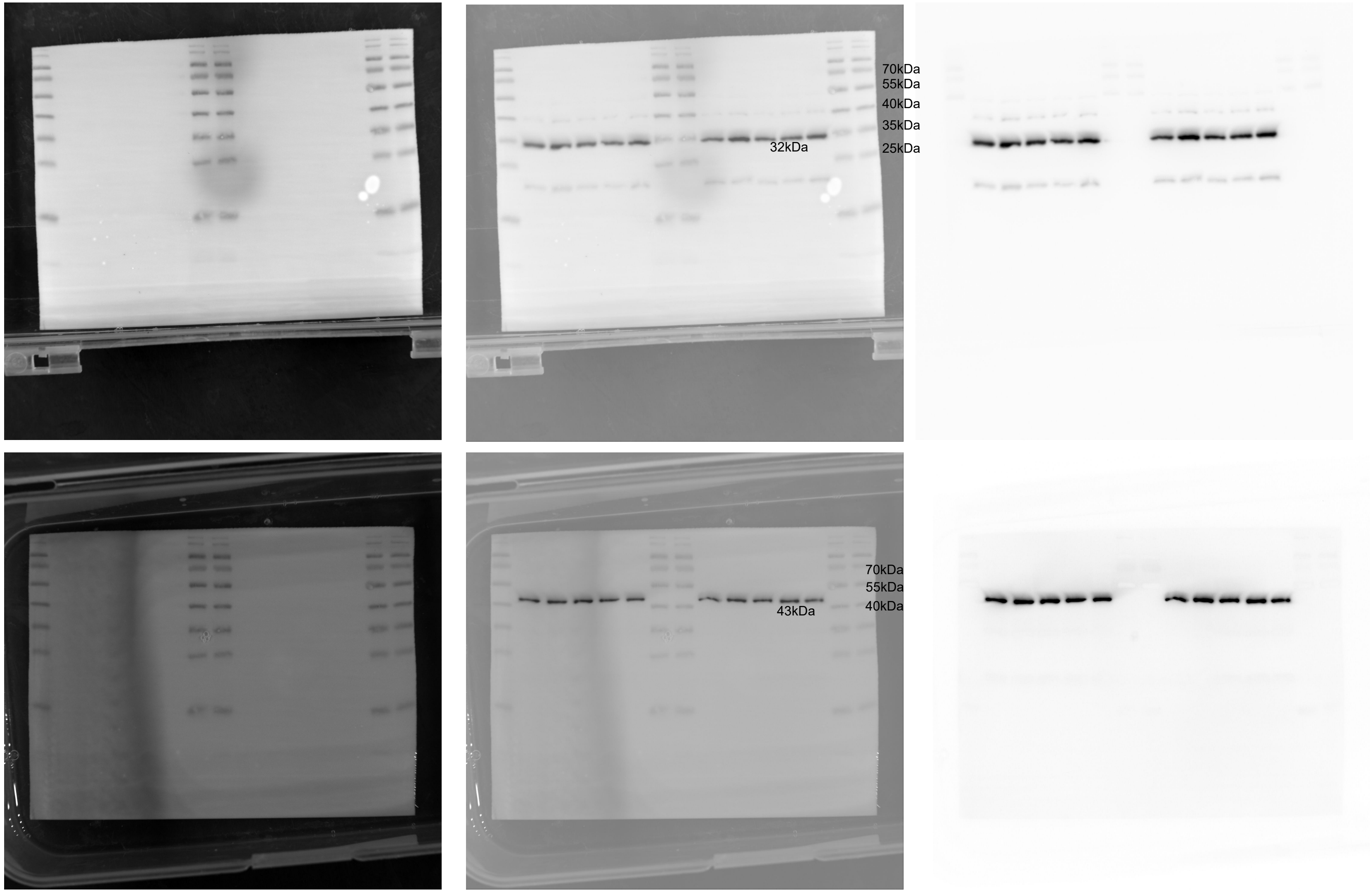

Mouse; Bax； Repeat*6

Marker: ThermoFisher 26616

Group:Sham, ICH, M2-Evs, miR-27n-3p cn, miR-27b-3p k/d

Figure. 8C

Bax

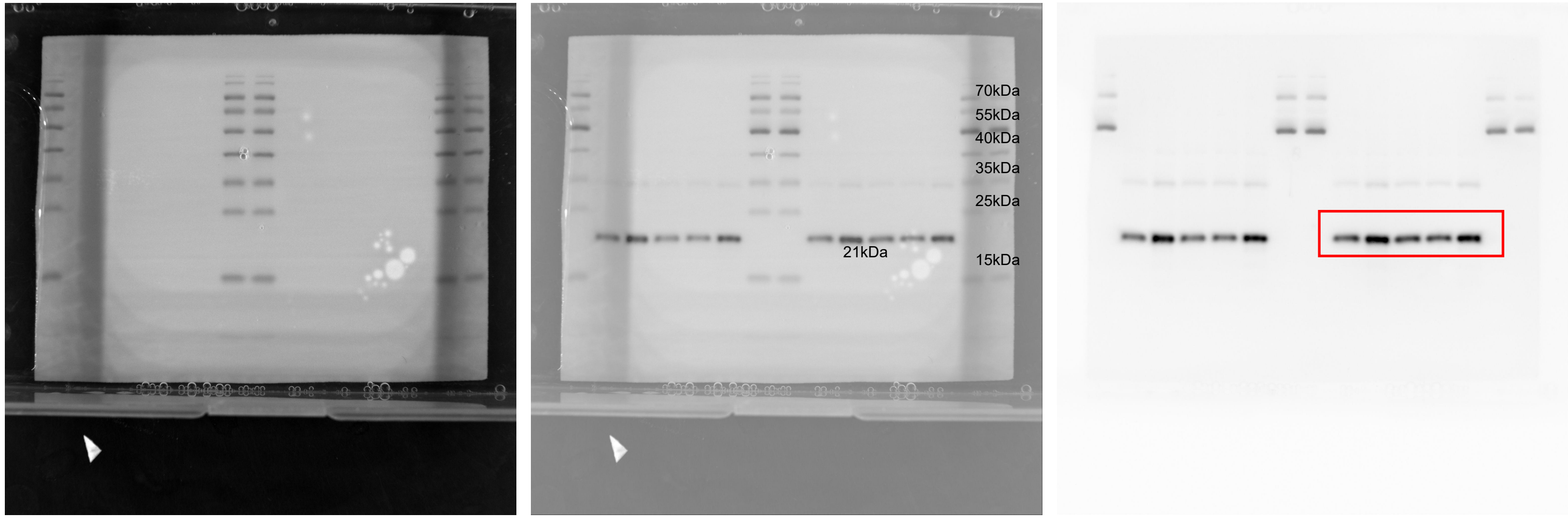

β-Actin

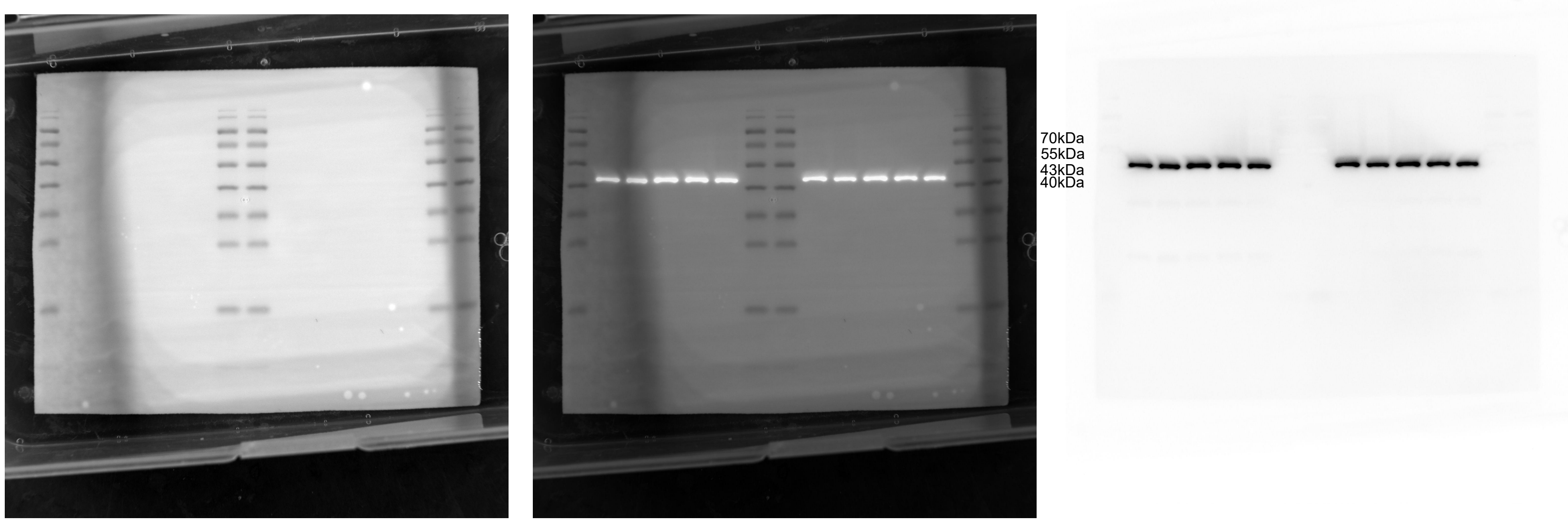

Marker: ThermoFisher 26616

Bax

β-Actin

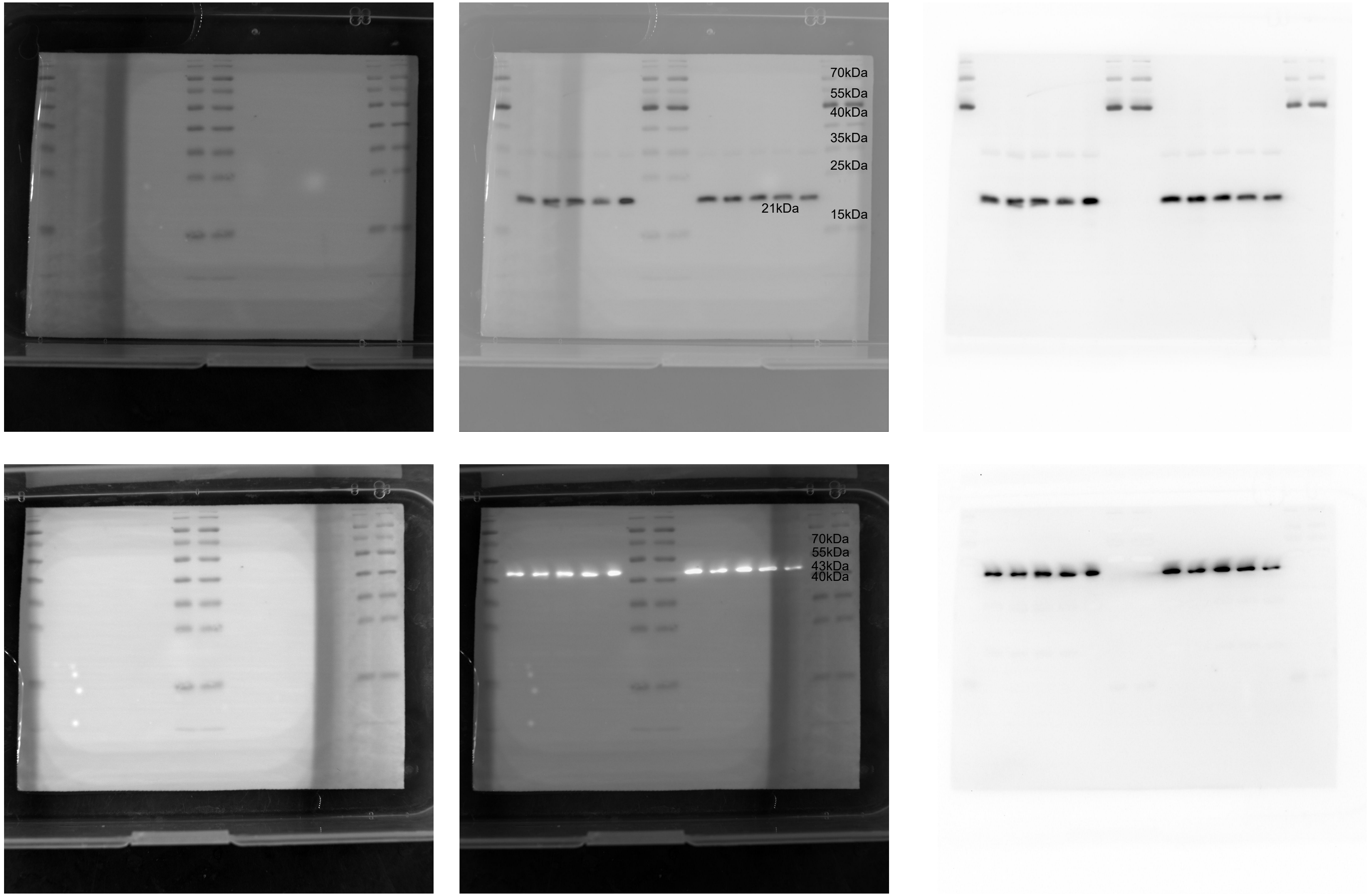

Marker: ThermoFisher 26616

Bax

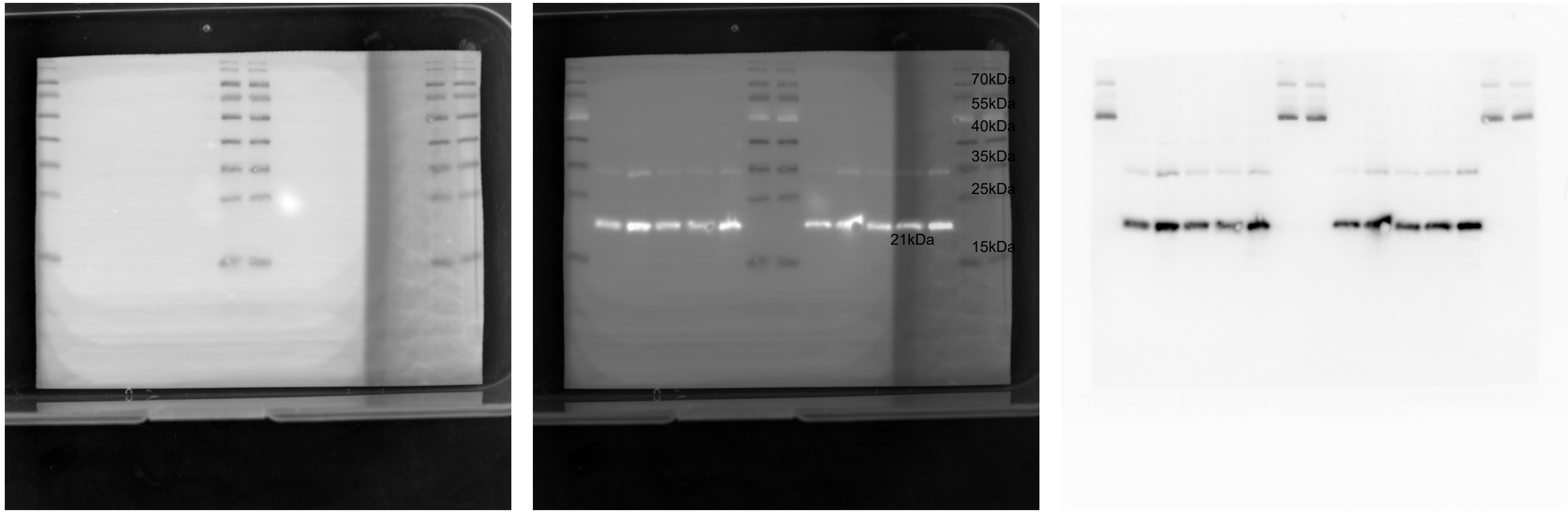

β-Actin

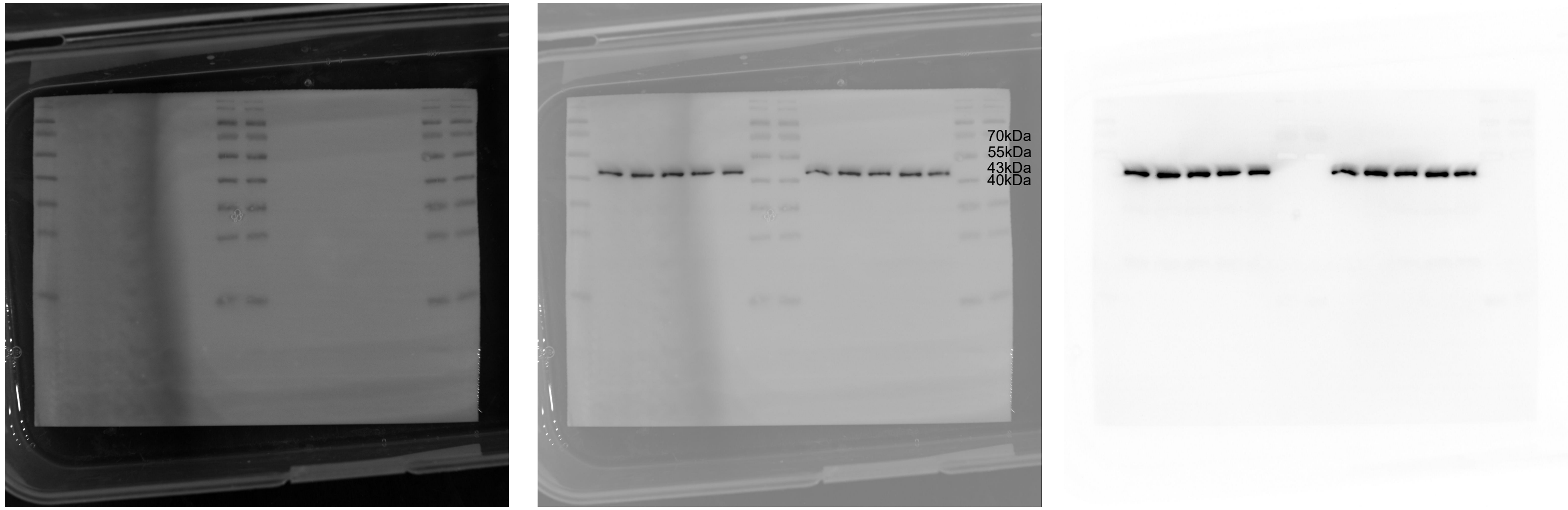

Mouse; Bcl-2； Repeat*6

Marker: ThermoFisher 26616

Group:Sham, ICH, M2-Evs, miR-27n-3p cn, miR-27b-3p k/d

Figure. 8C

Bcl-2

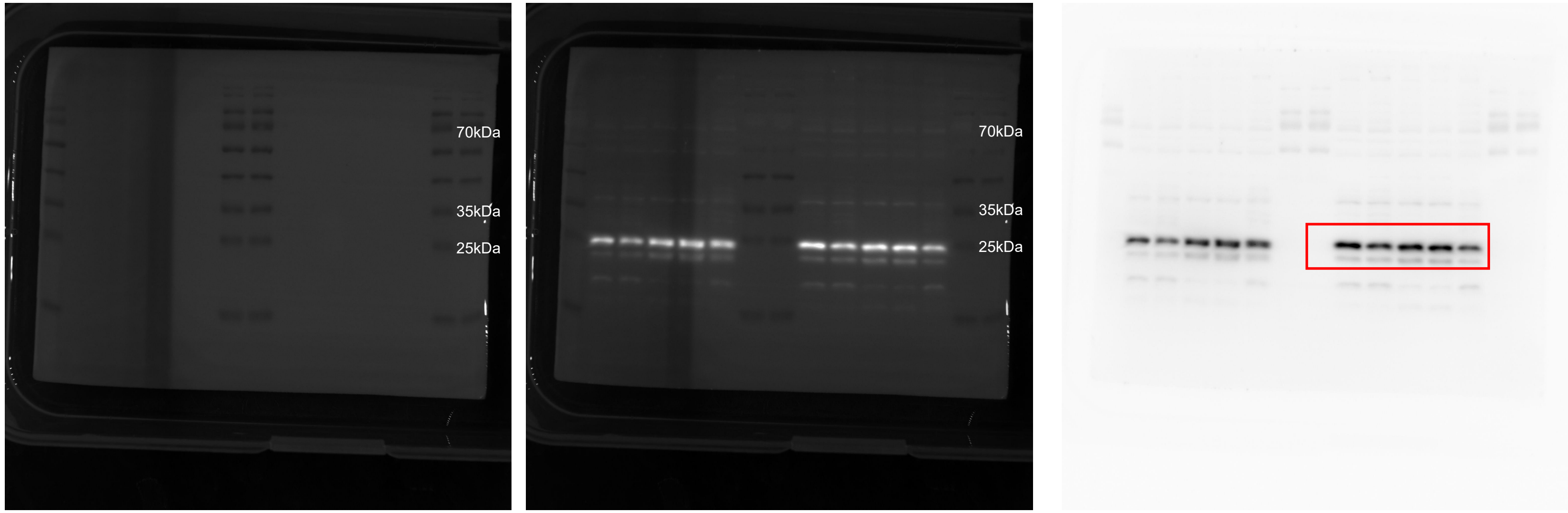

β-Actin

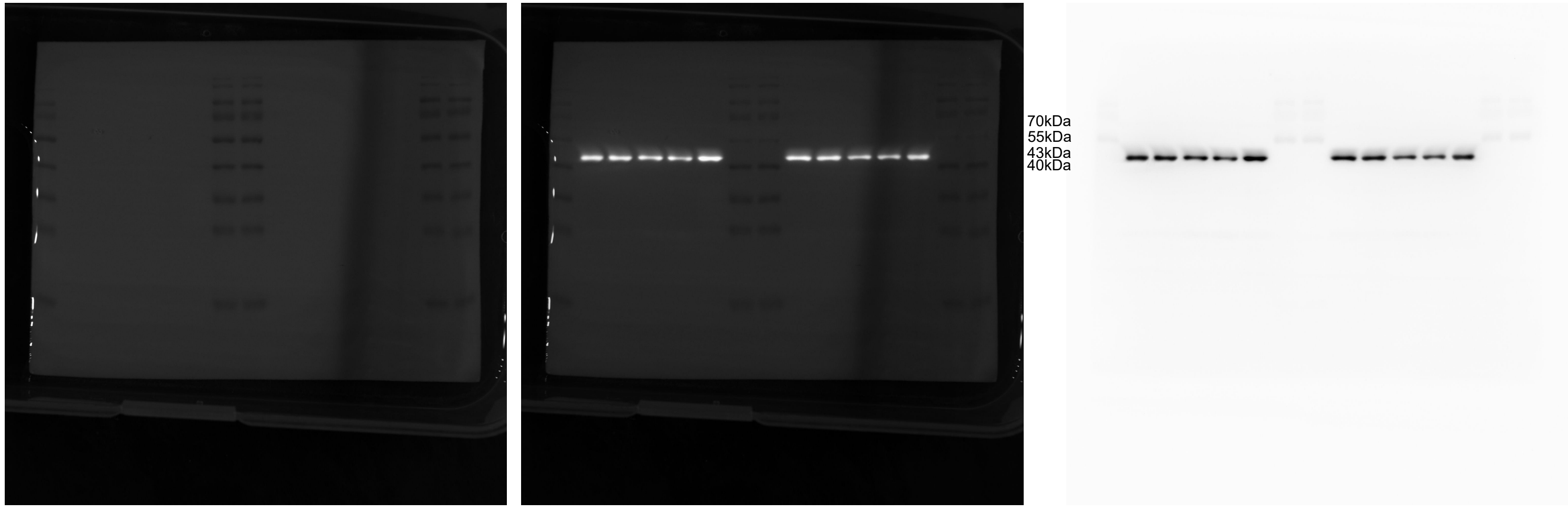

Marker: ThermoFisher 26616

Bcl-2

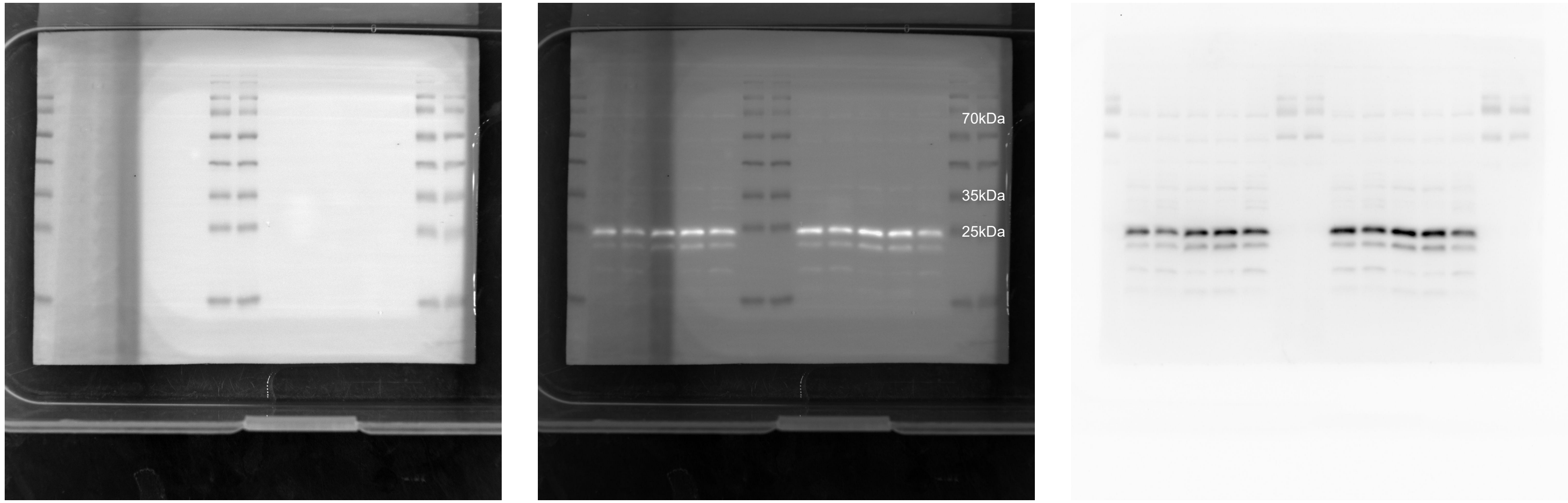

β-Actin

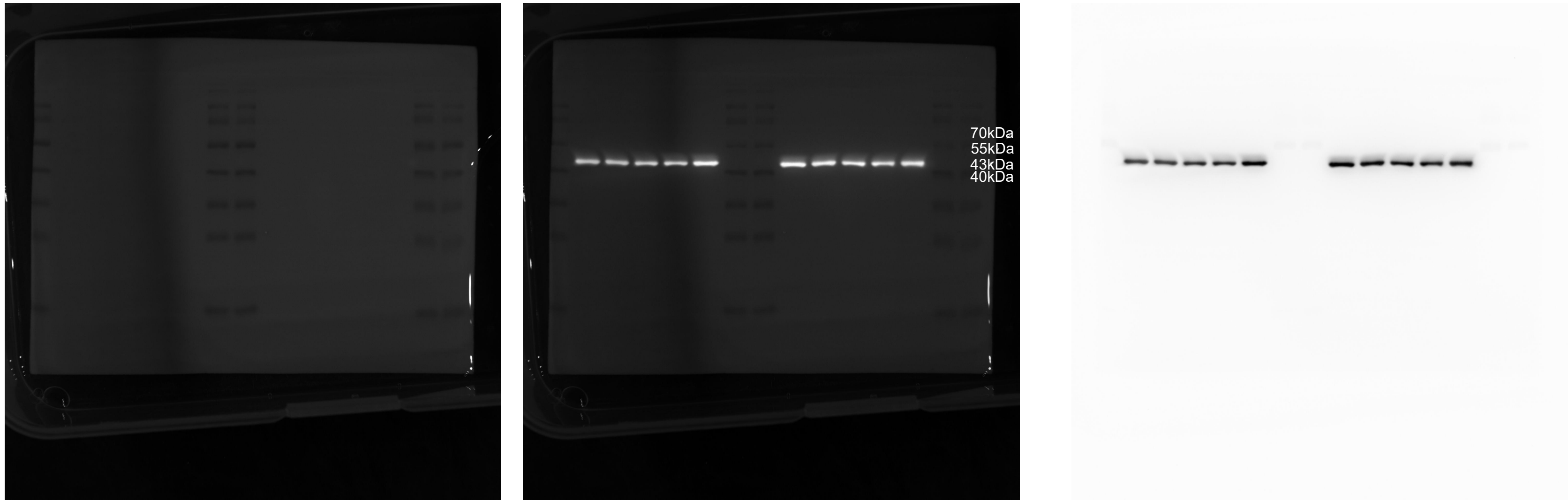

Marker: ThermoFisher 26616

Bcl-2

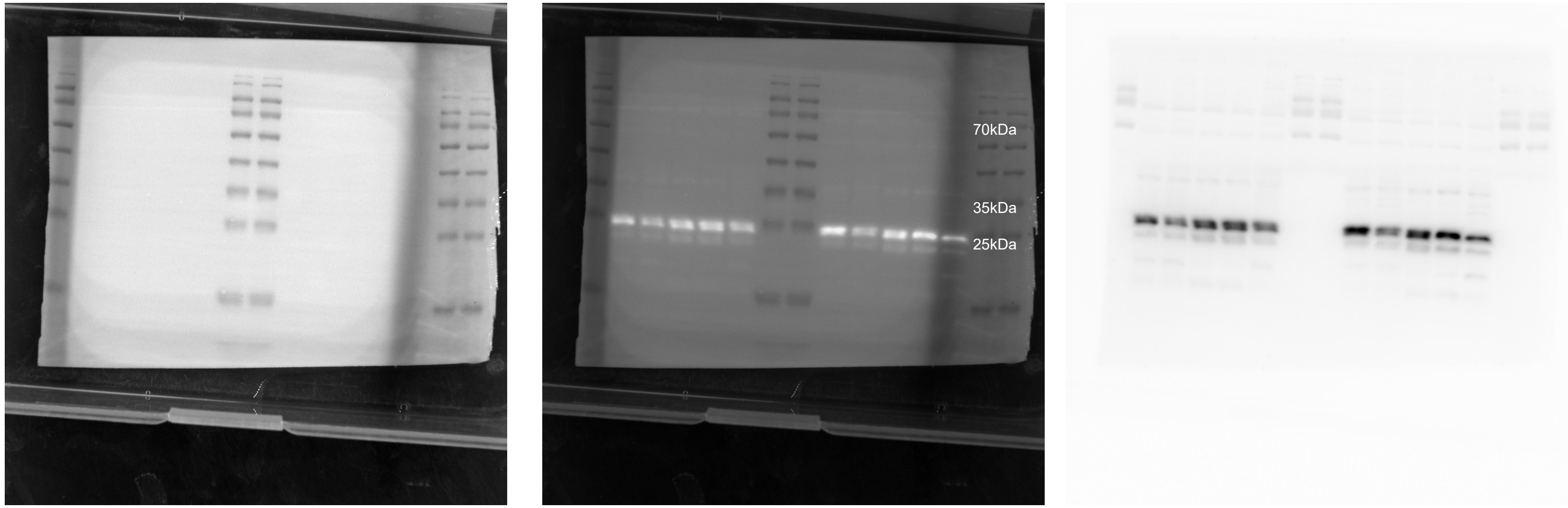

β-Actin

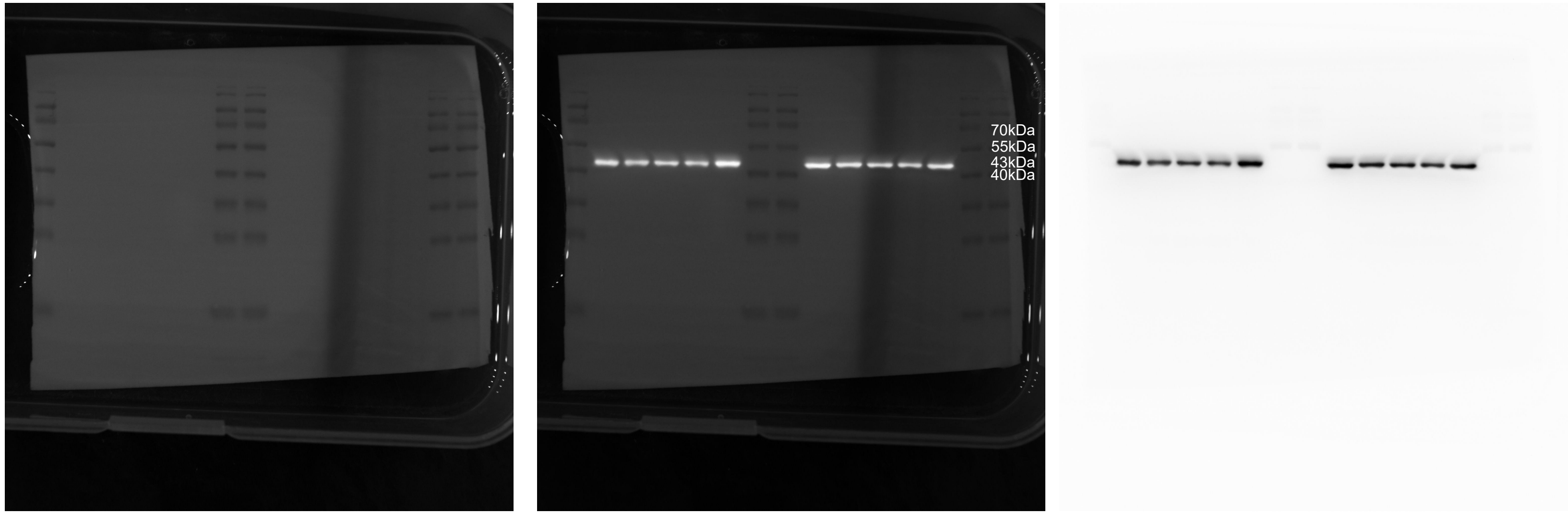

Mouse;Cyt-C；Repeat*6
Group:Sham, ICH, M2-Evs, miR-27n-3p cn, miR-27b-3p k/d
Figure. 8C

Marker: ThermoFisher 26616

Cyt-C

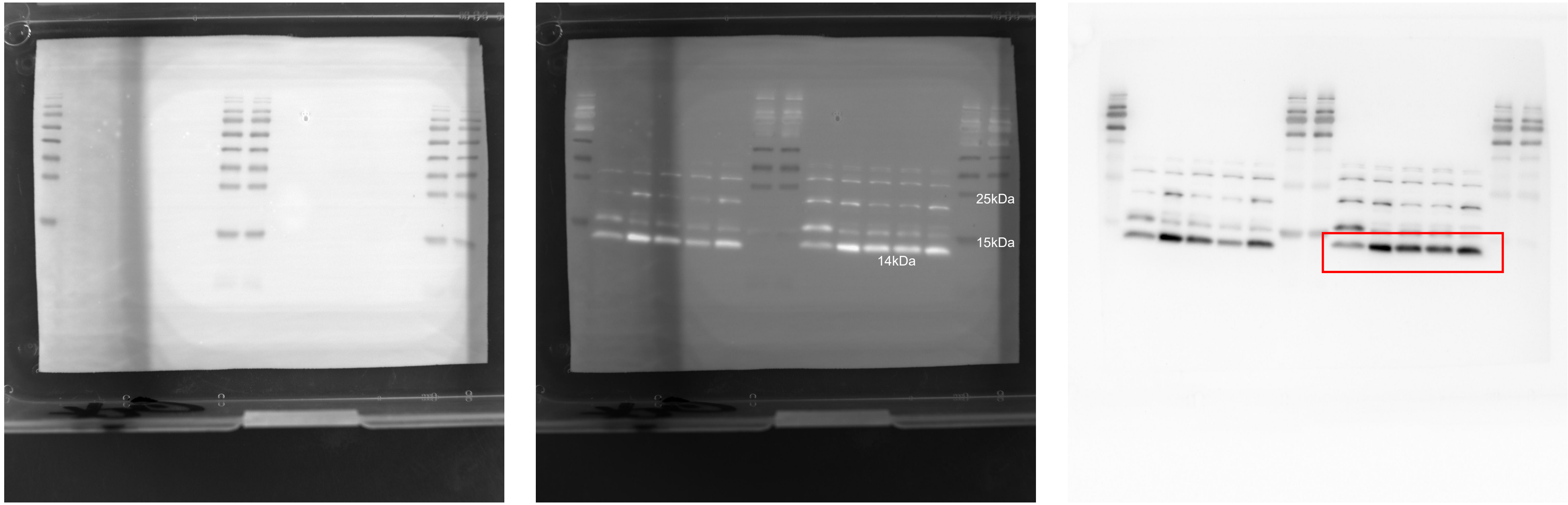

β-Actin

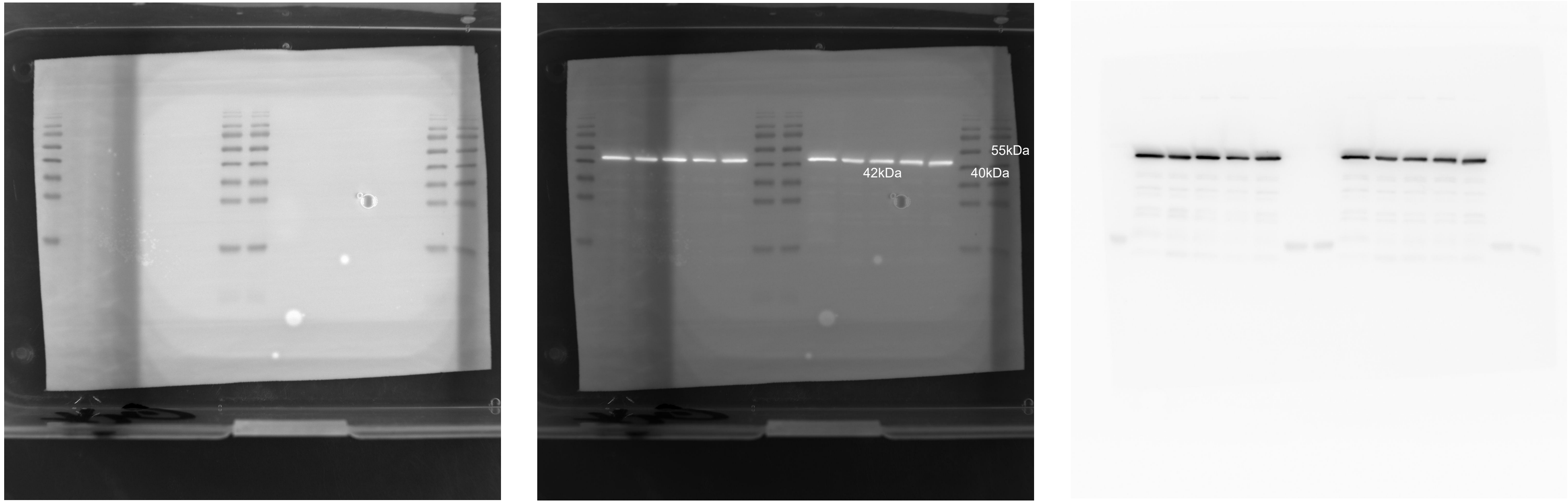

Marker: ThermoFisher 26616

Cyt-C

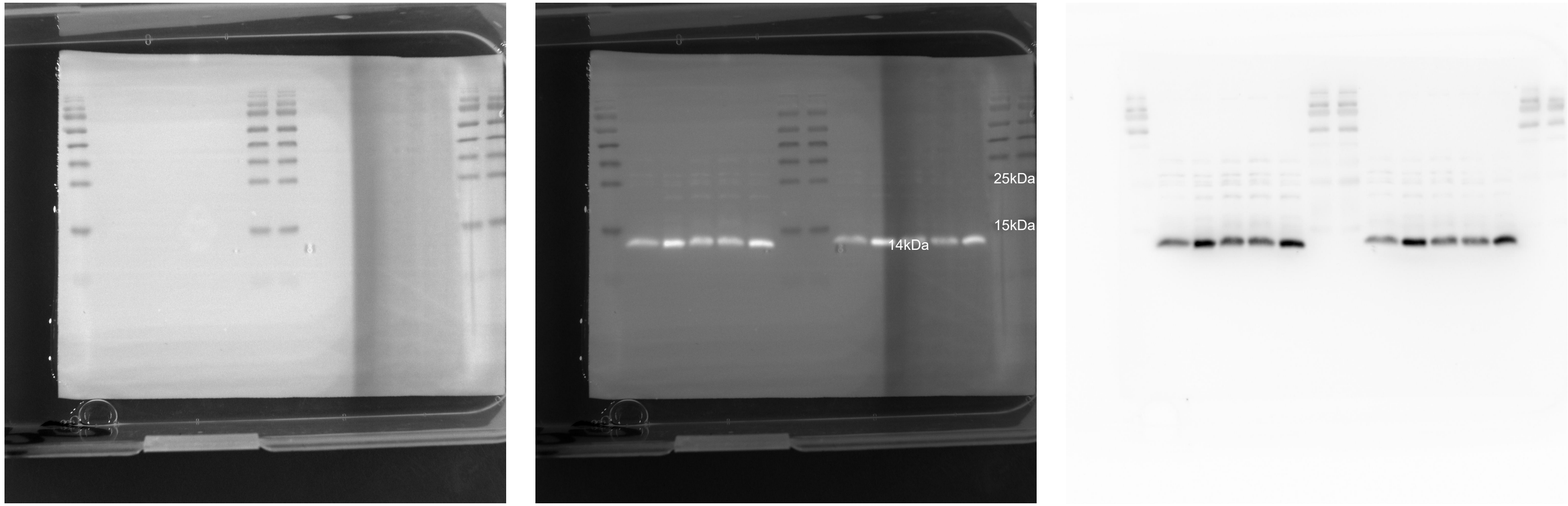

β-Actin

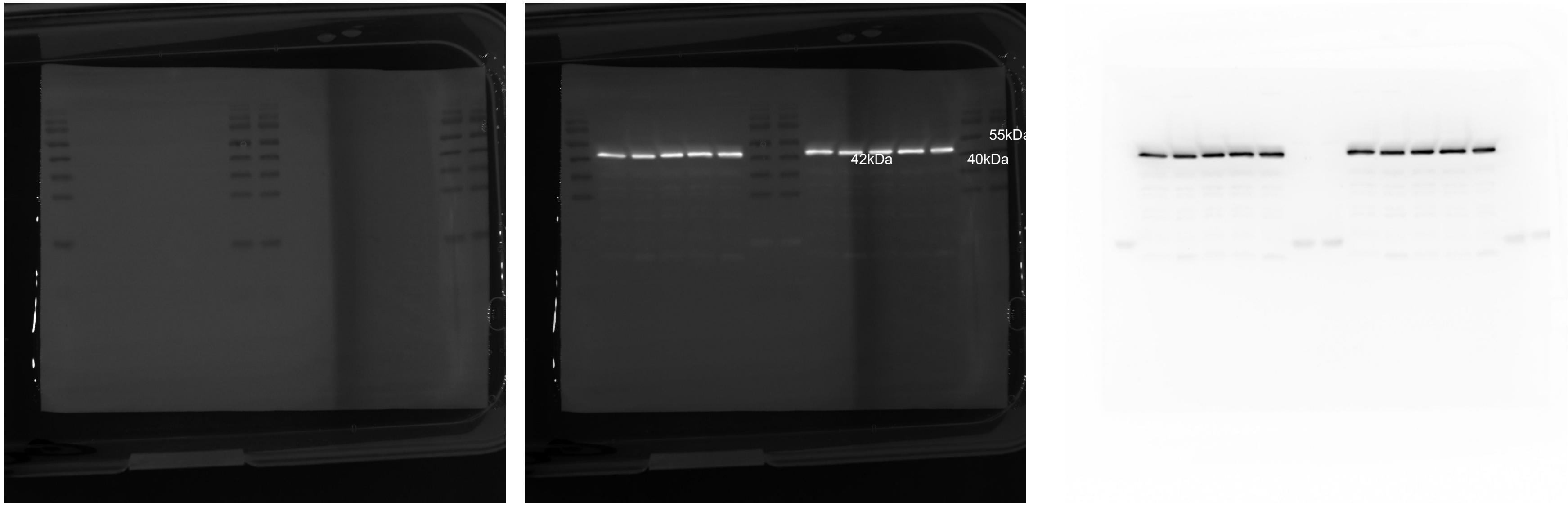

Marker: ThermoFisher 26616

Cyt-C

β-Actin

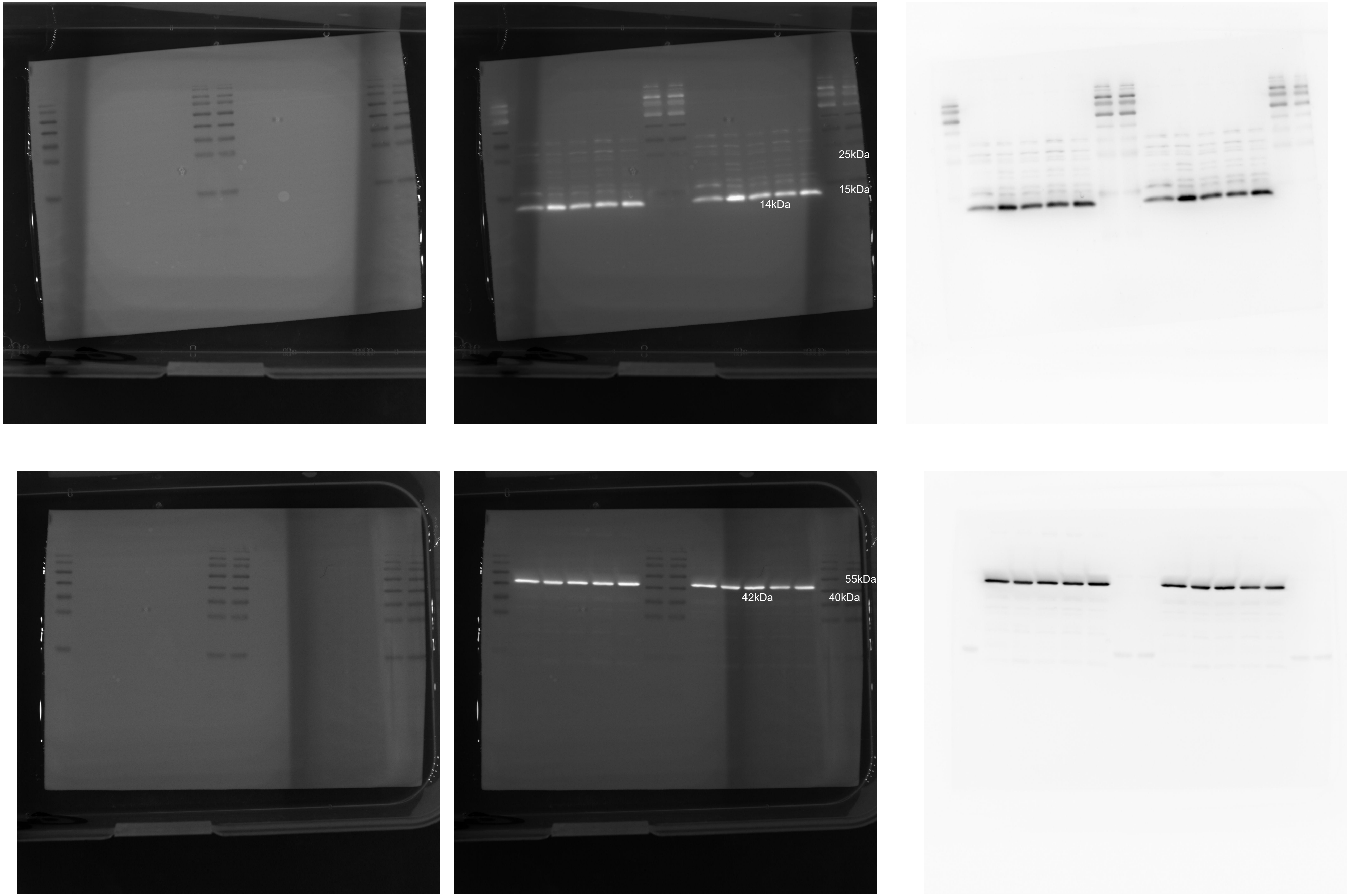

Mouse; Claudin 5; Repeat*6

Marker: ThermoFisher 26619

Group:Sham, ICH, M2-Evs, miR-27n-3p cn, miR-27b-3p k/d

Figure. 8K

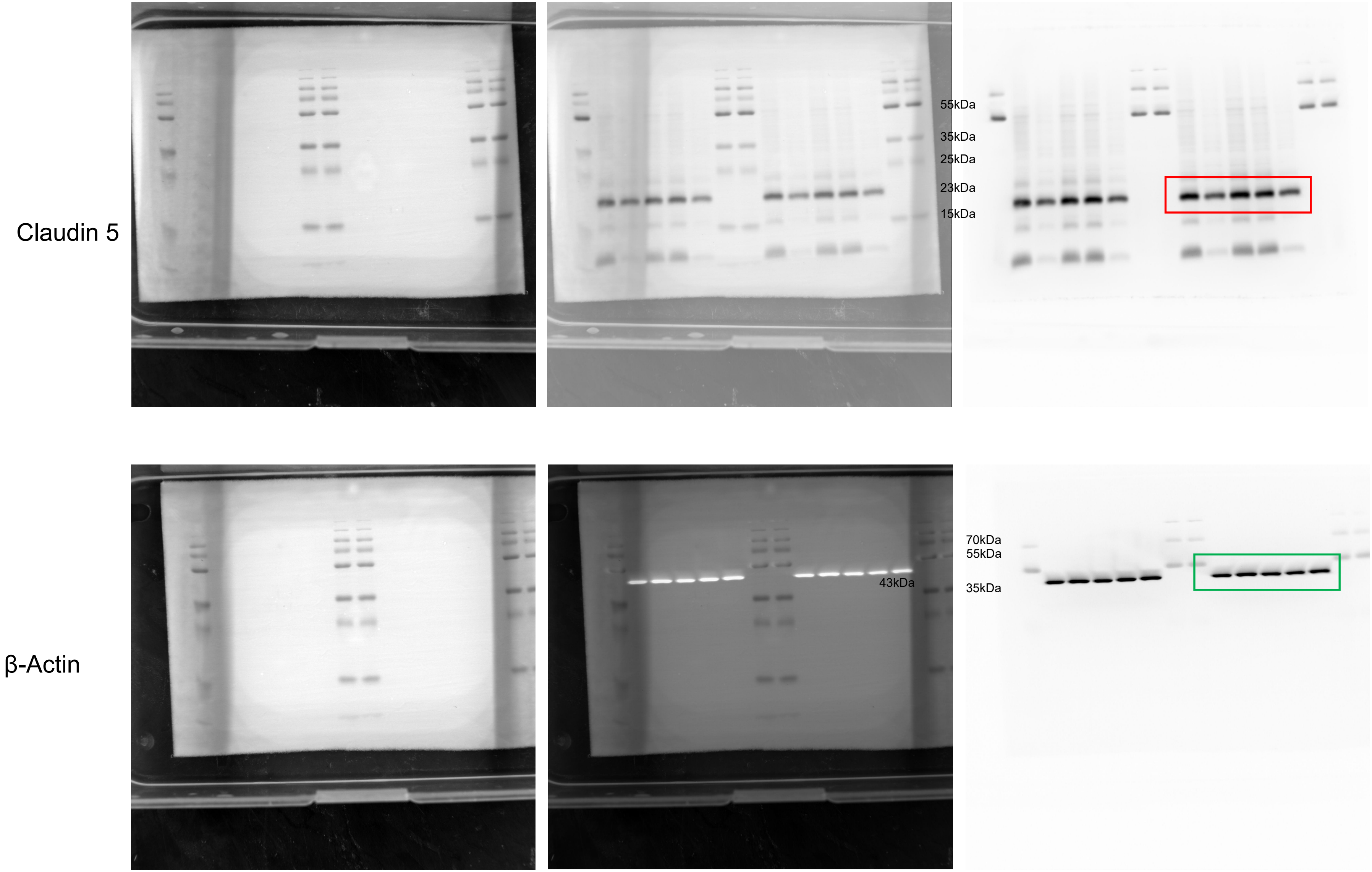

Marker: ThermoFisher 26619

Claudin 5

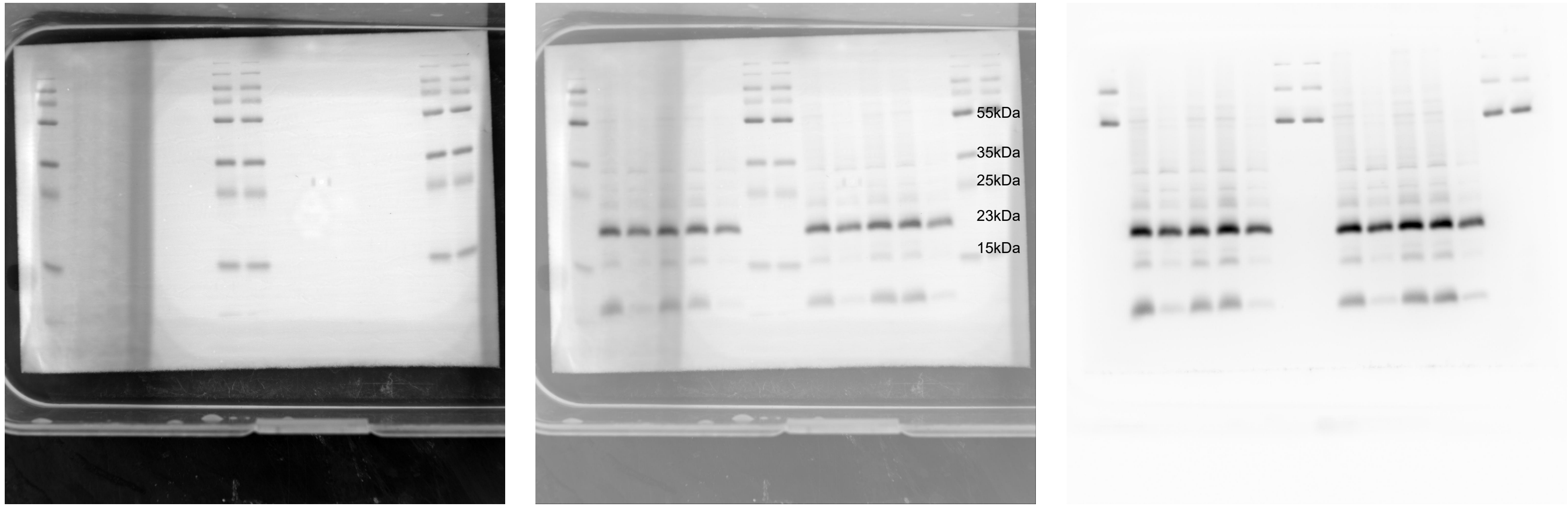

β-Actin

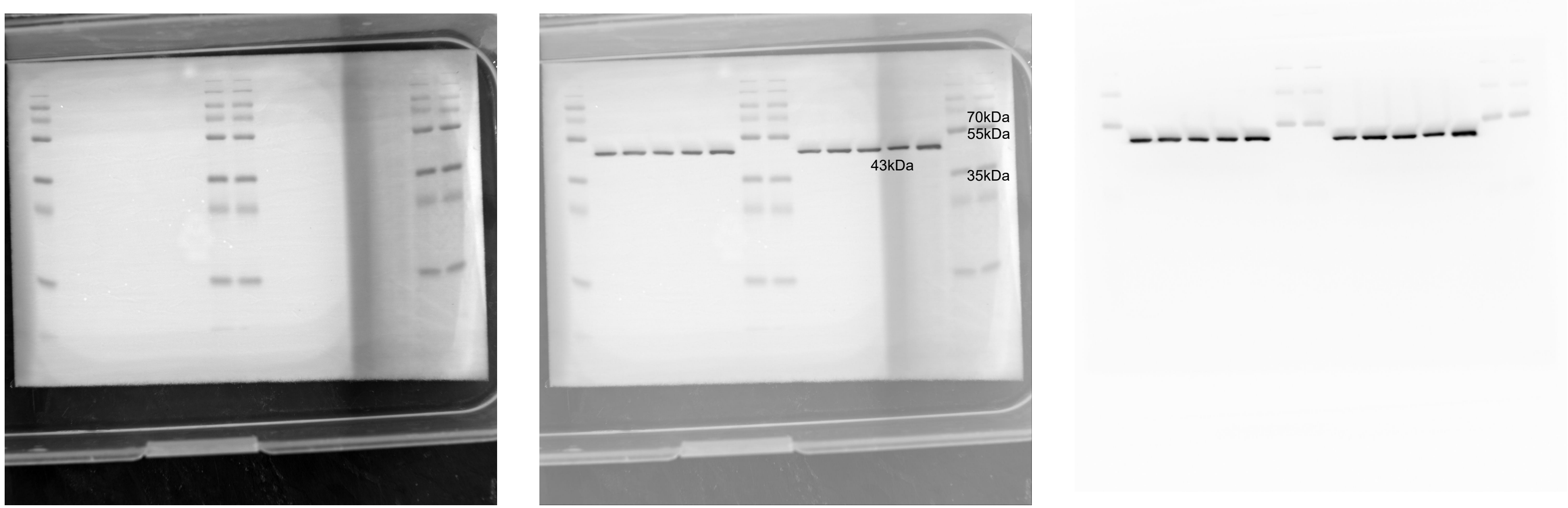

Marker: ThermoFisher 26619

Claudin 5

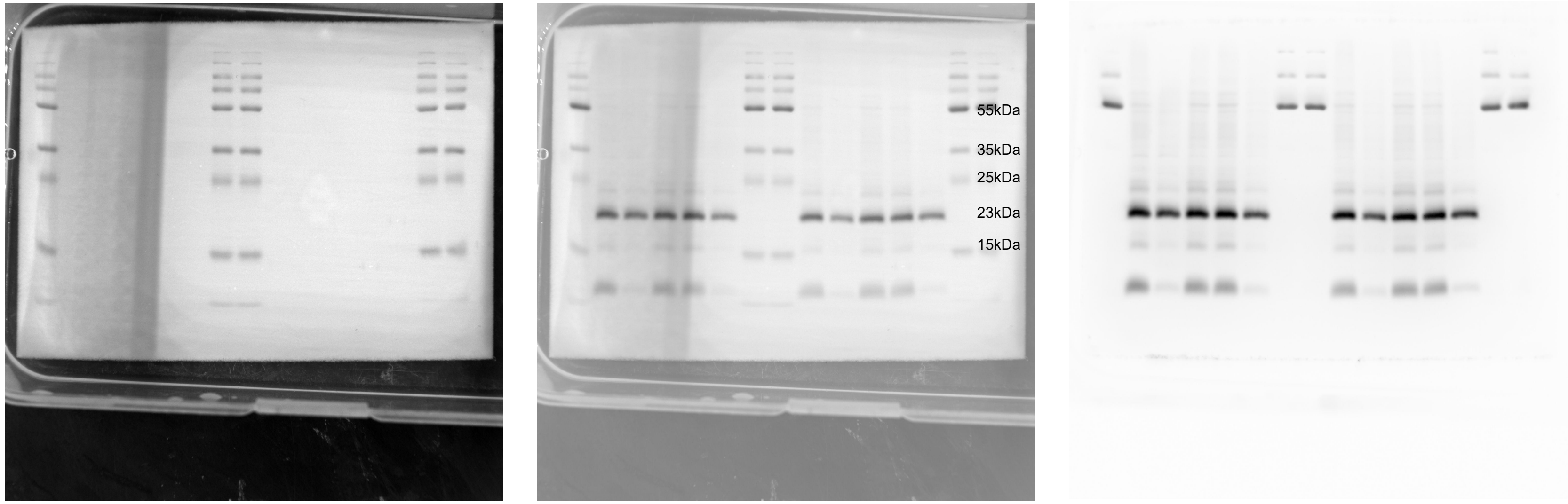

β-Actin

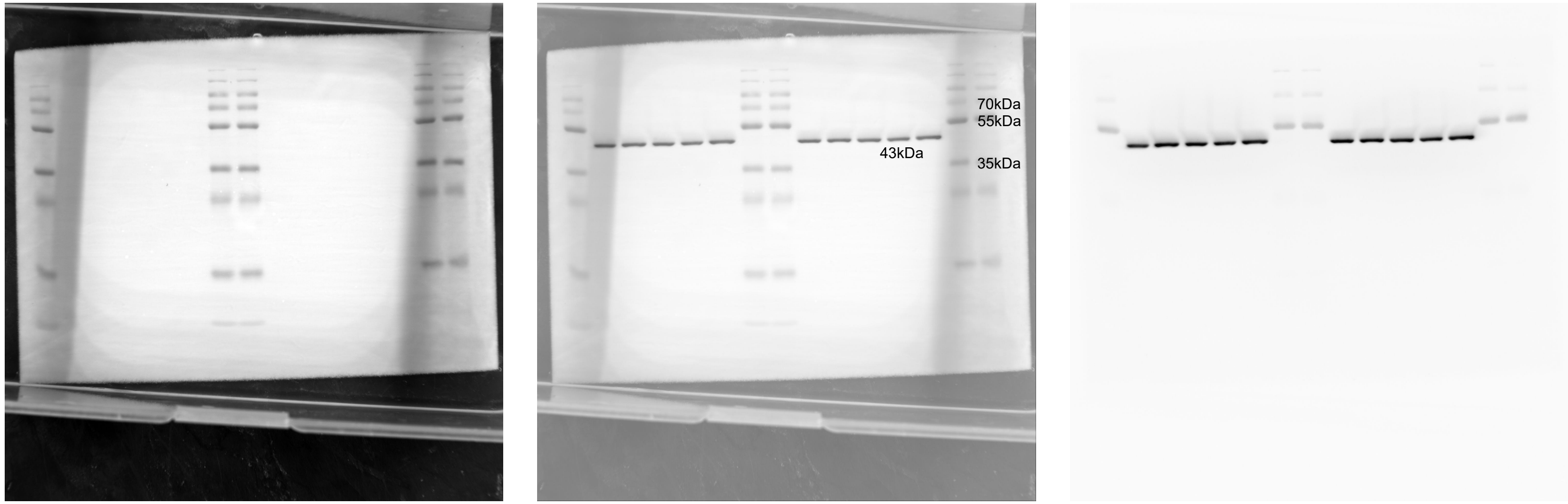

Mouse; Occludin; Repeat*6

Group:Sham, ICH, M2-Evs, miR-27n-3p cn, miR-27b-3p k/d

Figure. 8K

Marker: ThermoFisher 26616

Occludin

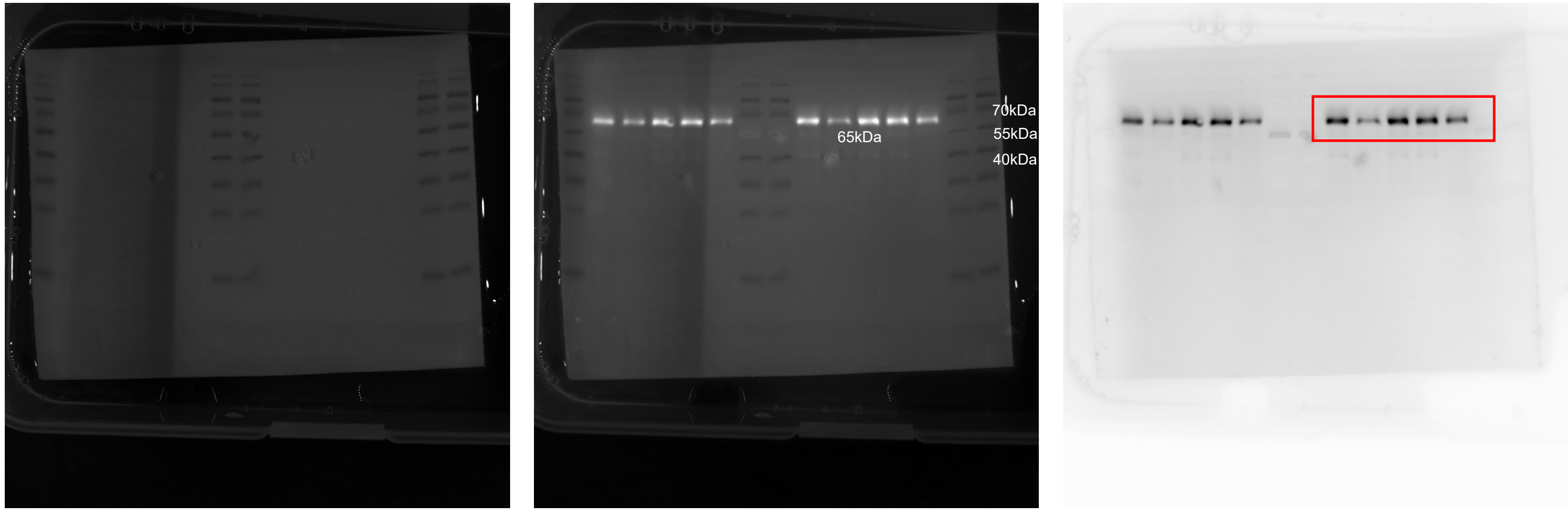

β-Actin

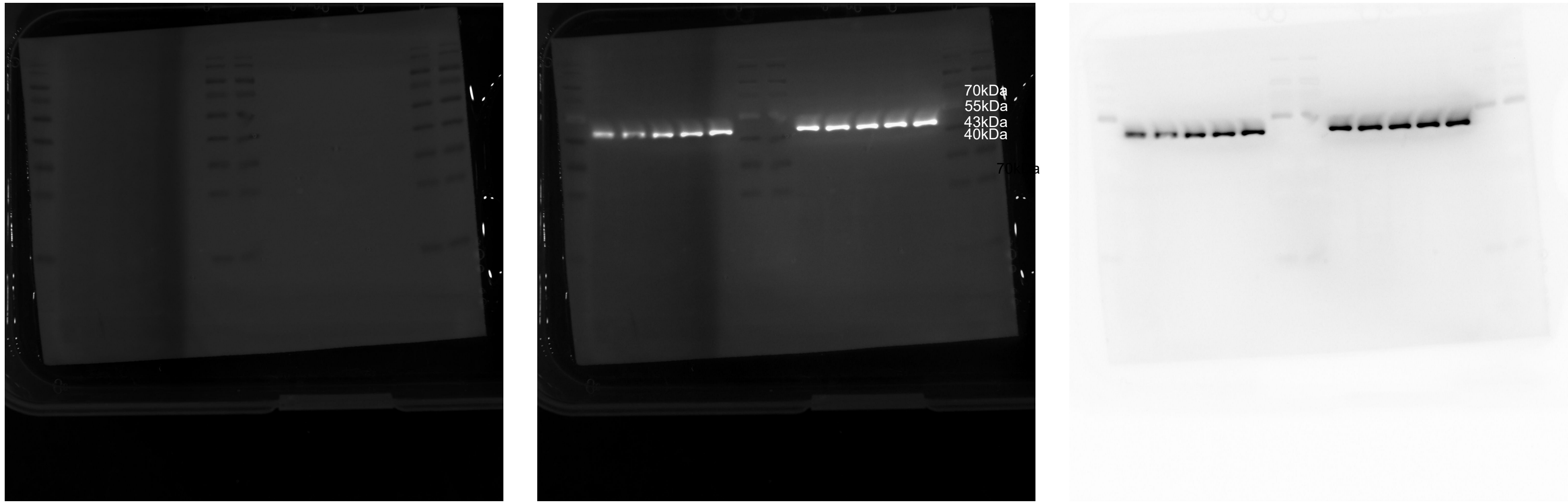

Marker: ThermoFisher 26616

Occludin

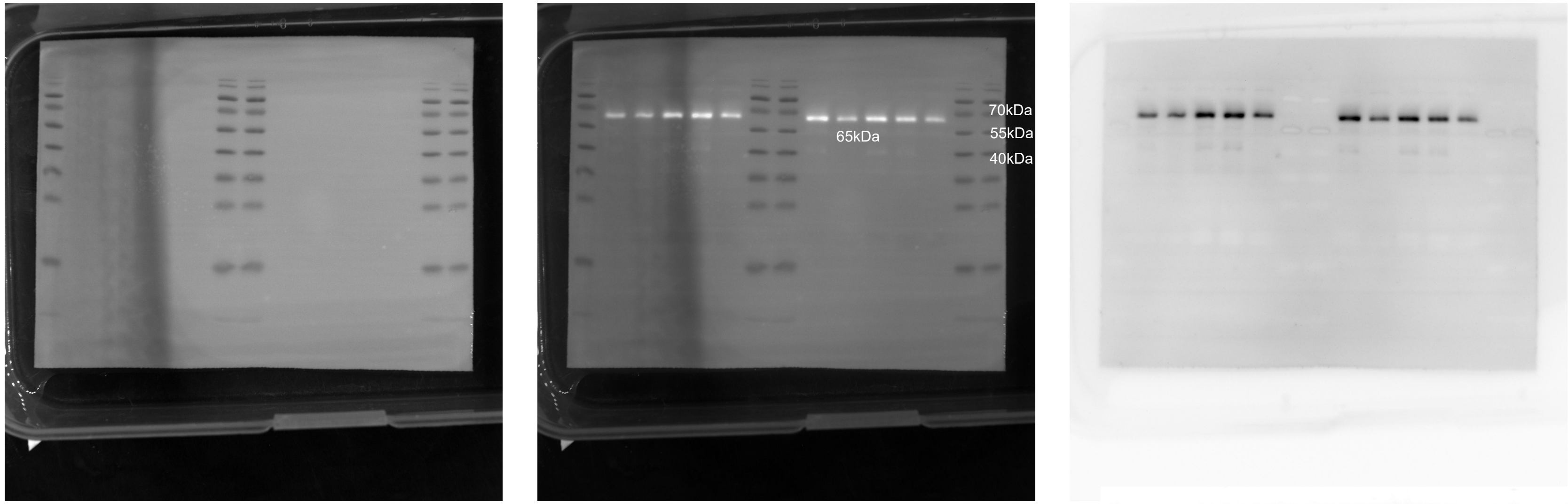

β-Actin

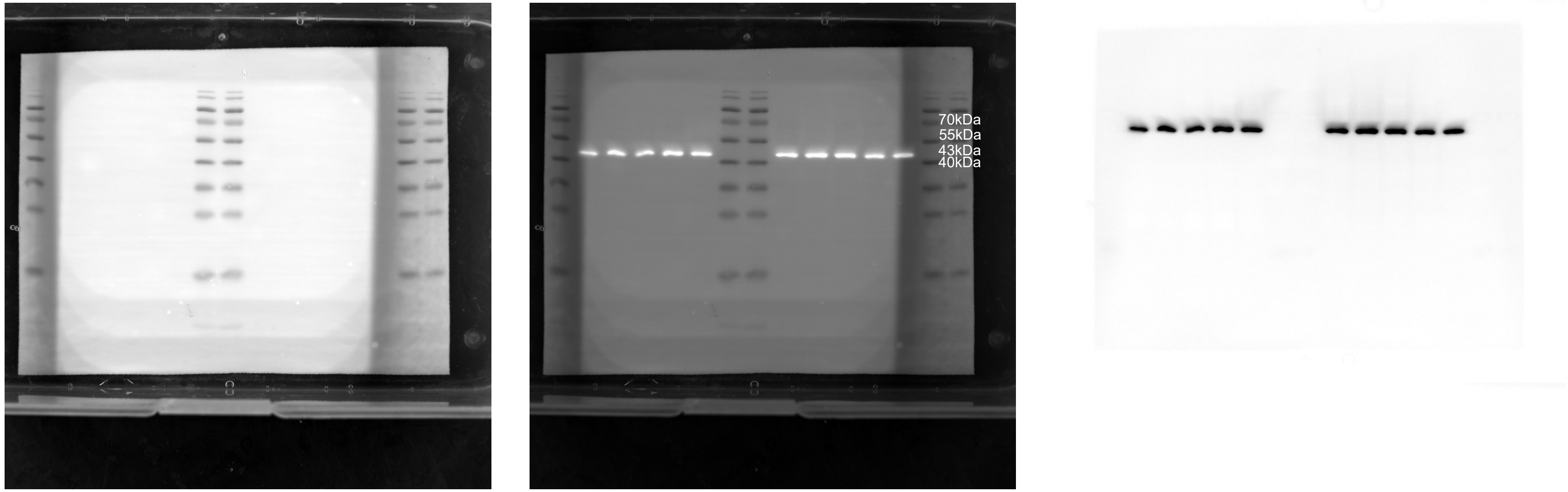

Marker: ThermoFisher 26616

Occludin

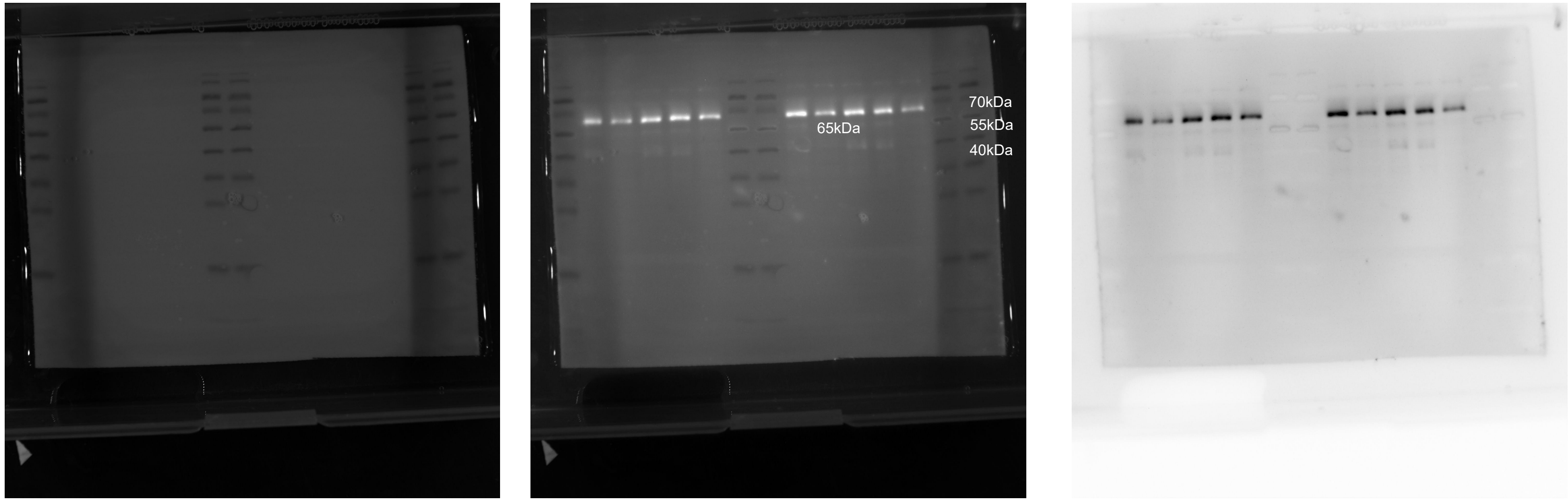

β-Actin

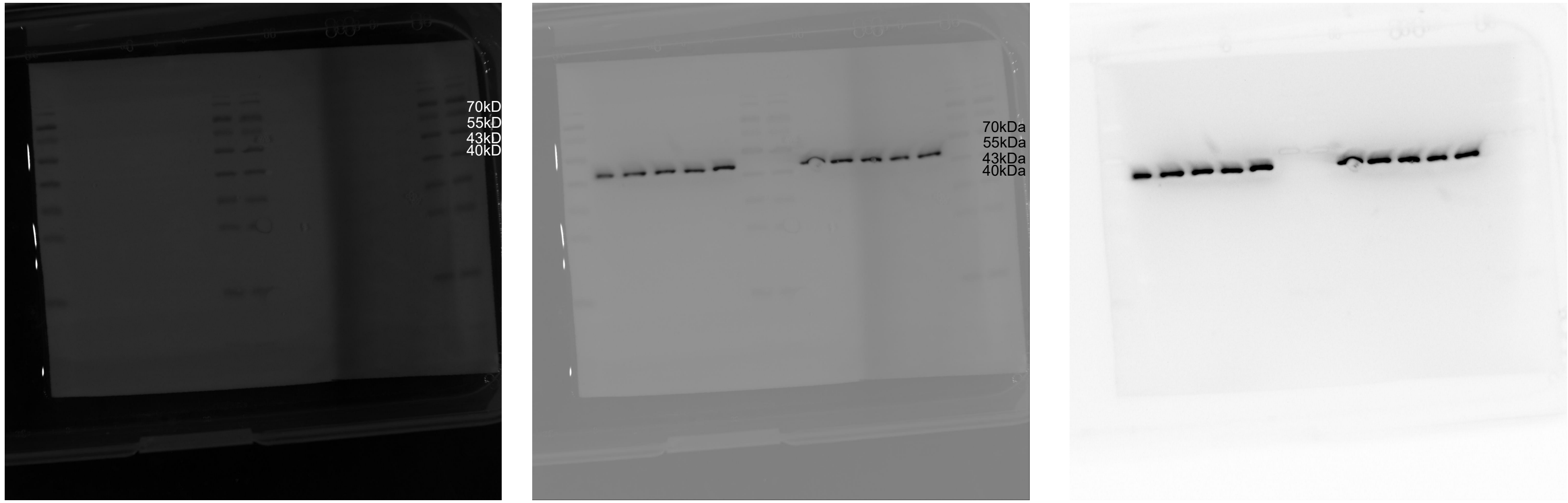

Mouse; VE-Cadherin；Repeat*6

Group:Sham, ICH, M2-Evs, miR-27n-3p cn, miR-27b-3p k/d

Figure. 8K

Marker: ThermoFisher 26616

VE-Cadherin

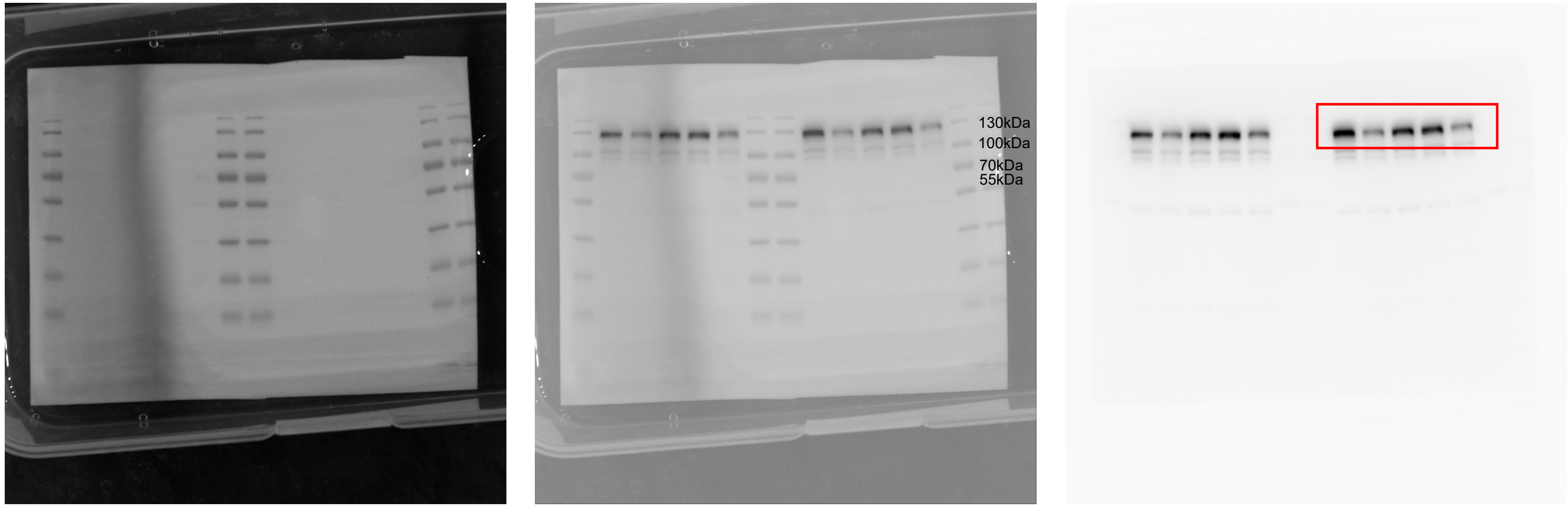

β-Actin

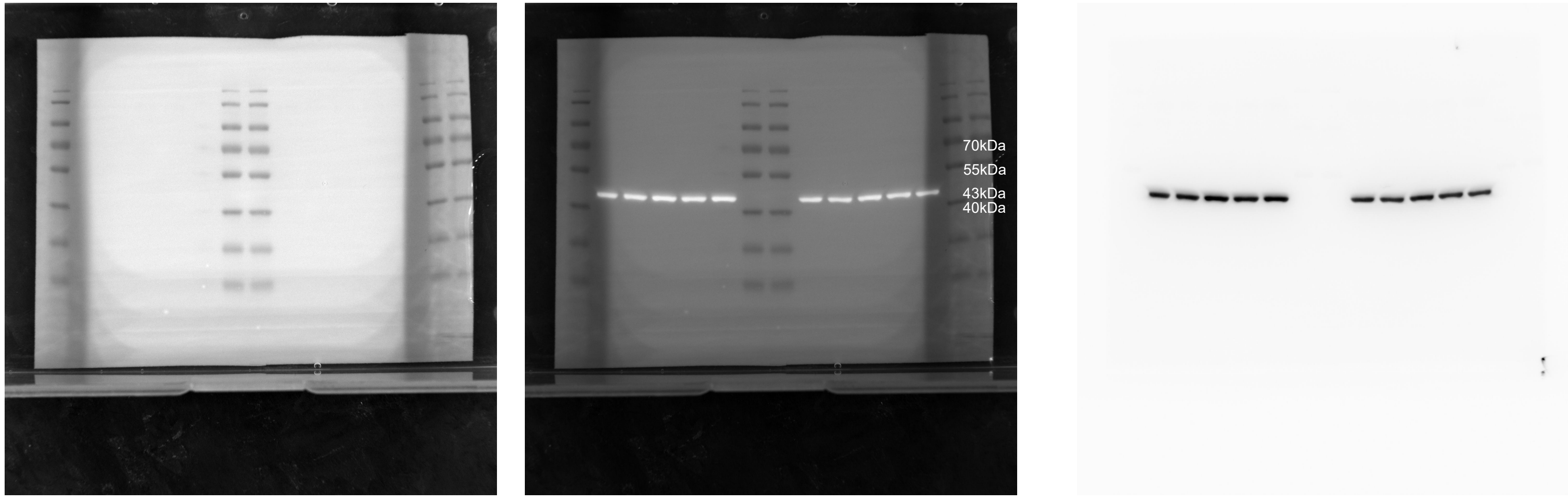

Marker: ThermoFisher 26616

VE-Cadherin

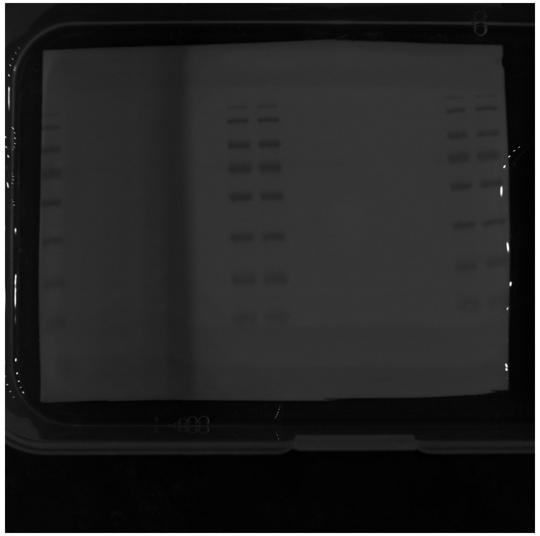

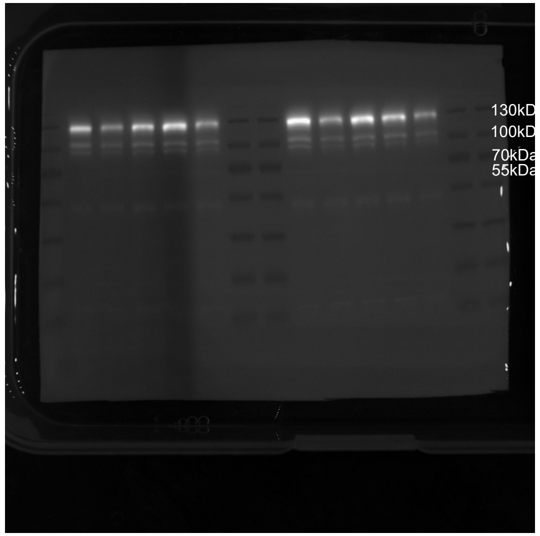

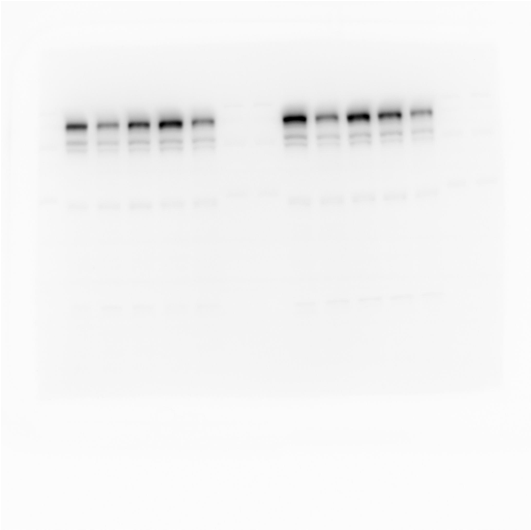

β-Actin

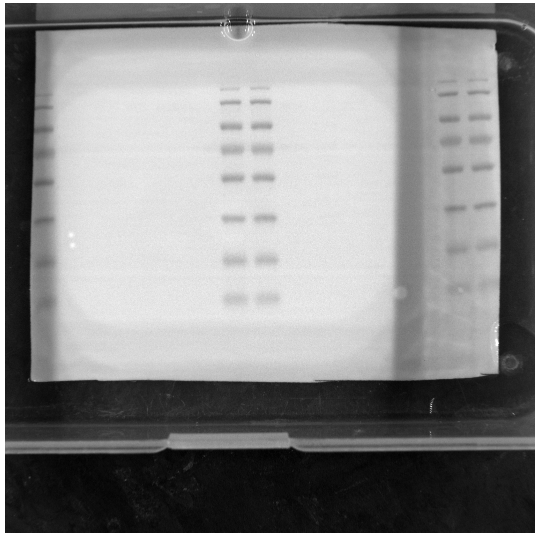

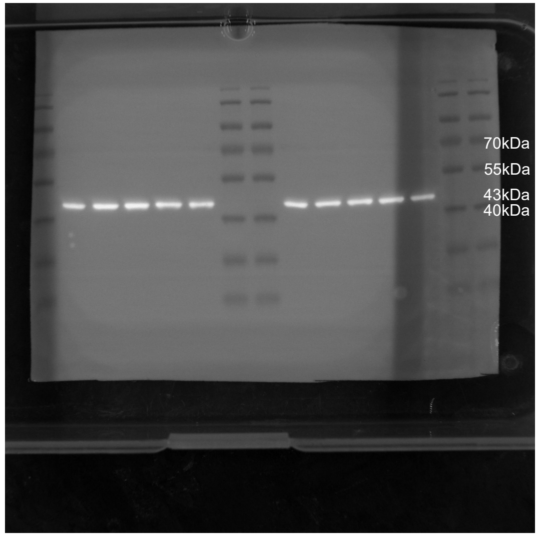

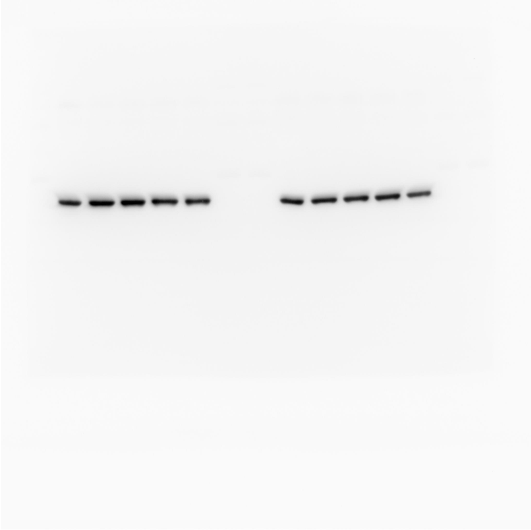

Marker: ThermoFisher 26616

VE-Cadherin

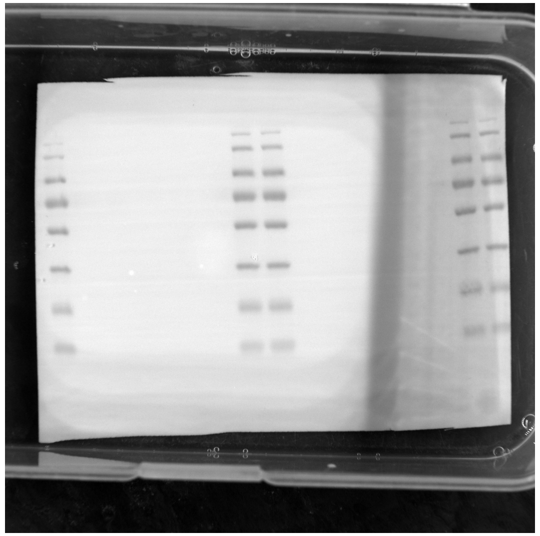

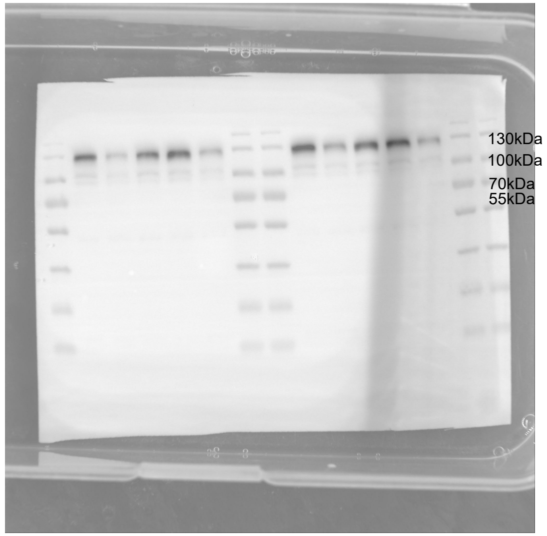

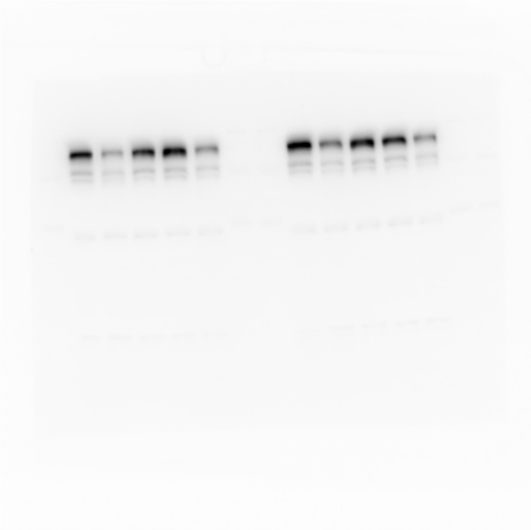

β-Actin

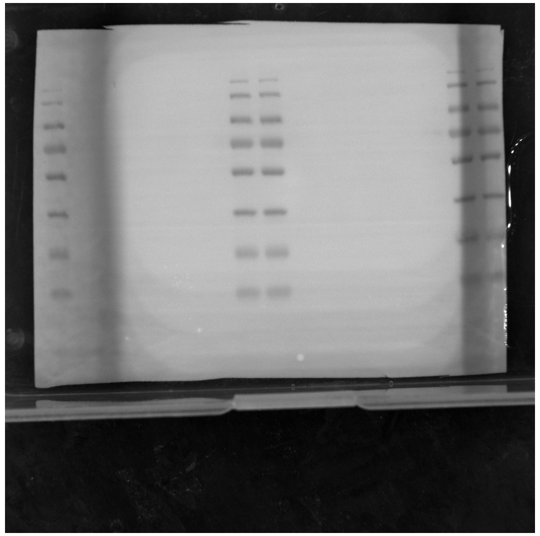

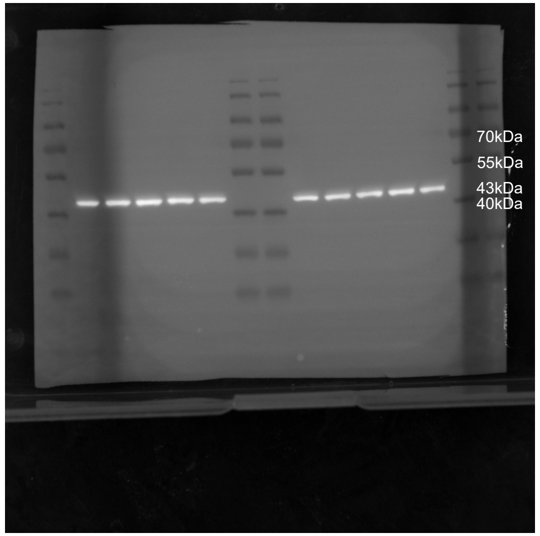

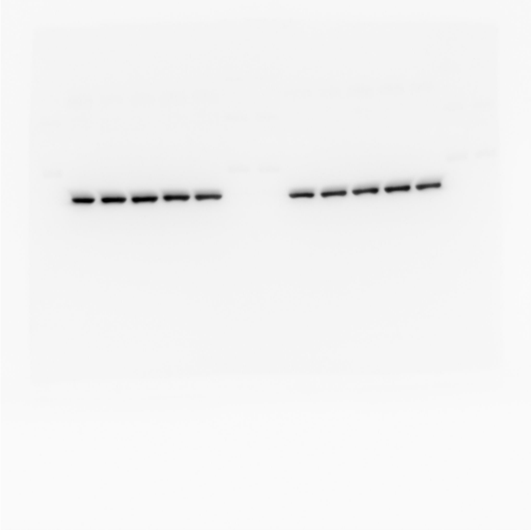

Cell; p-MKK4; Repeat*3
Group:Sham, Hemin, M2-Evs, miR-27n-3p cn, miR-27b-3p k/d
Figure. 9G

Marker: ThermoFisher 26616

p-MKK4

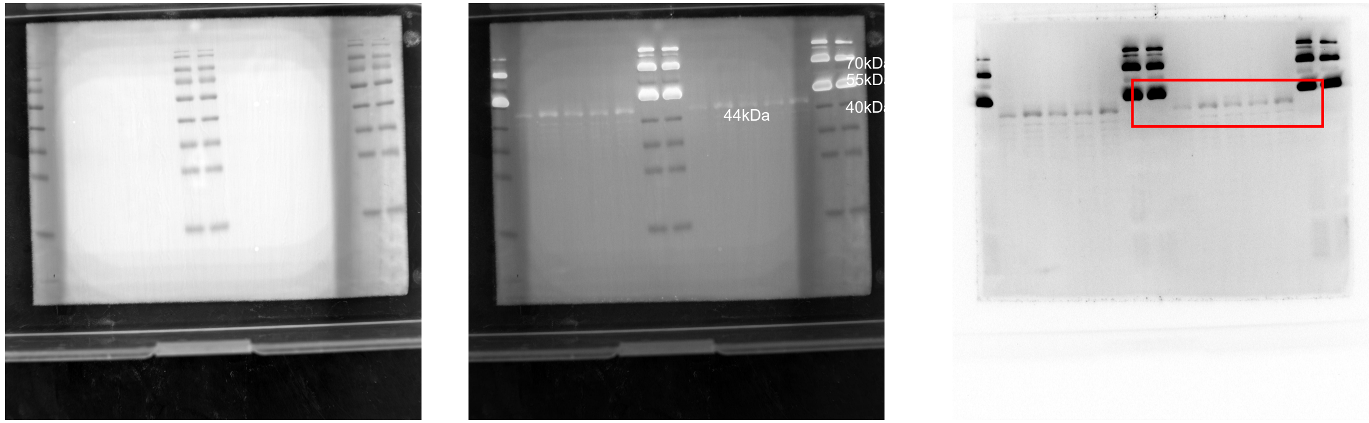

MKK4

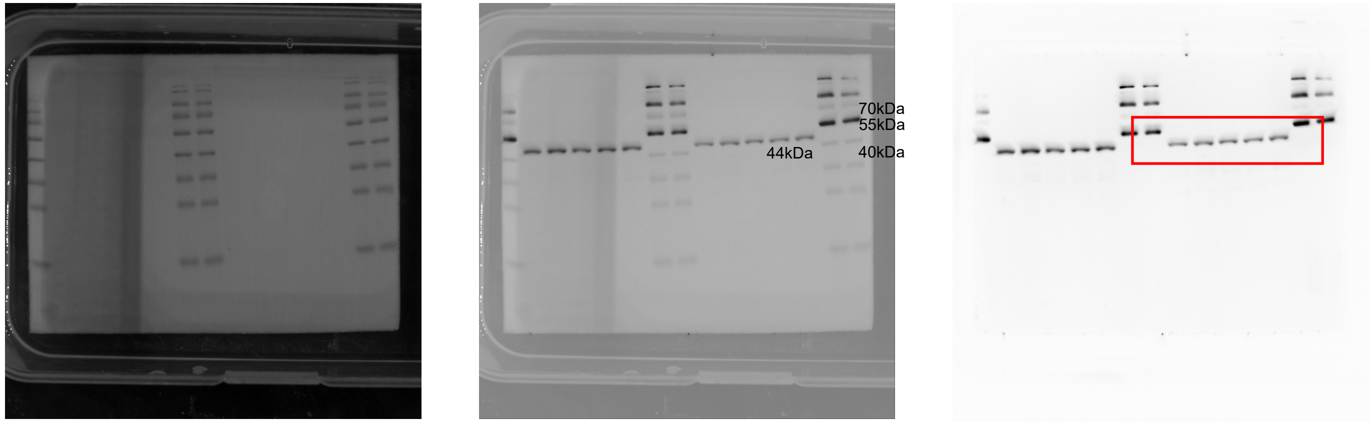

GAPDH

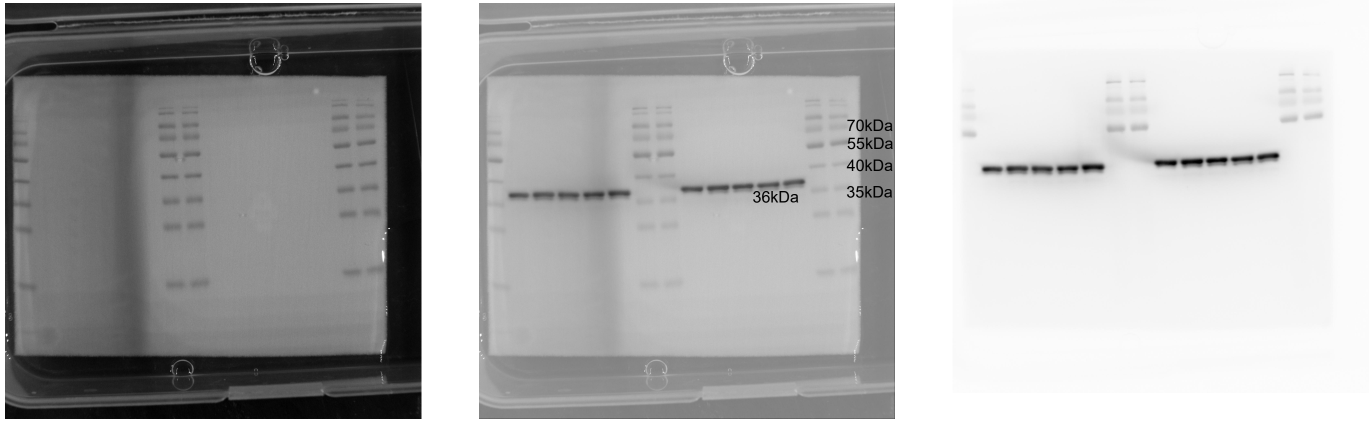

Marker: ThermoFisher 26616

p-MKK4

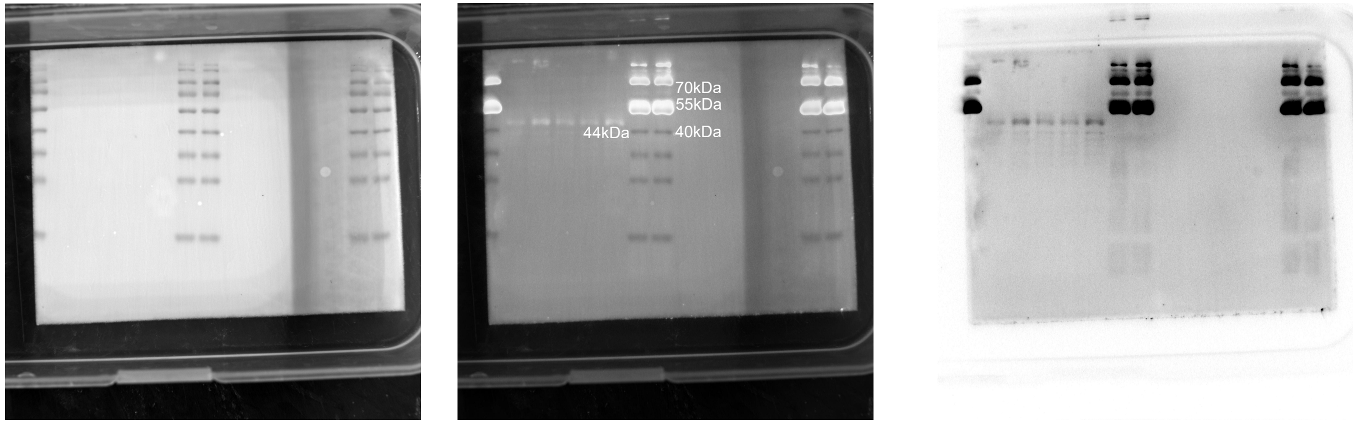

MKK4

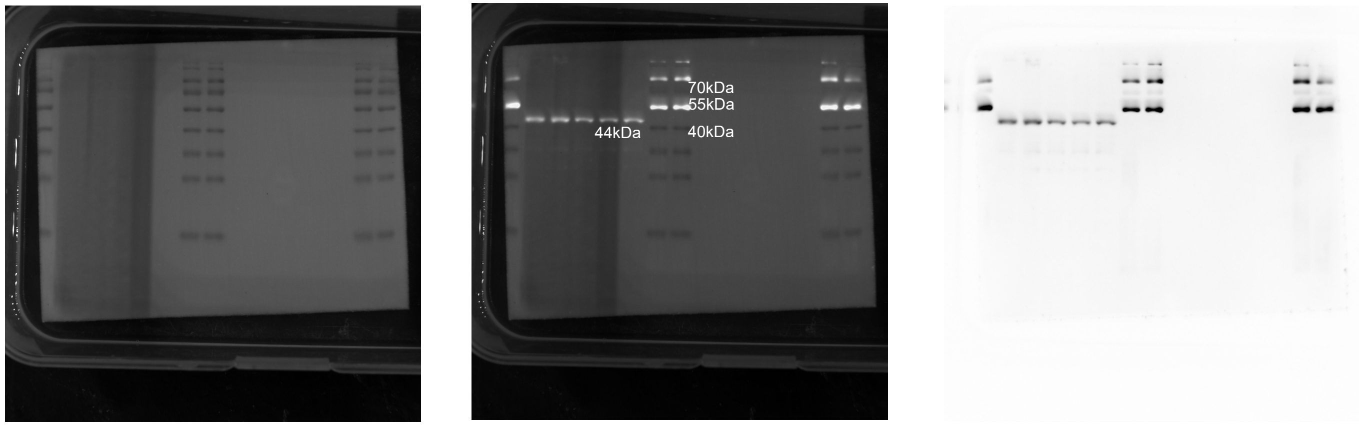

GAPDH

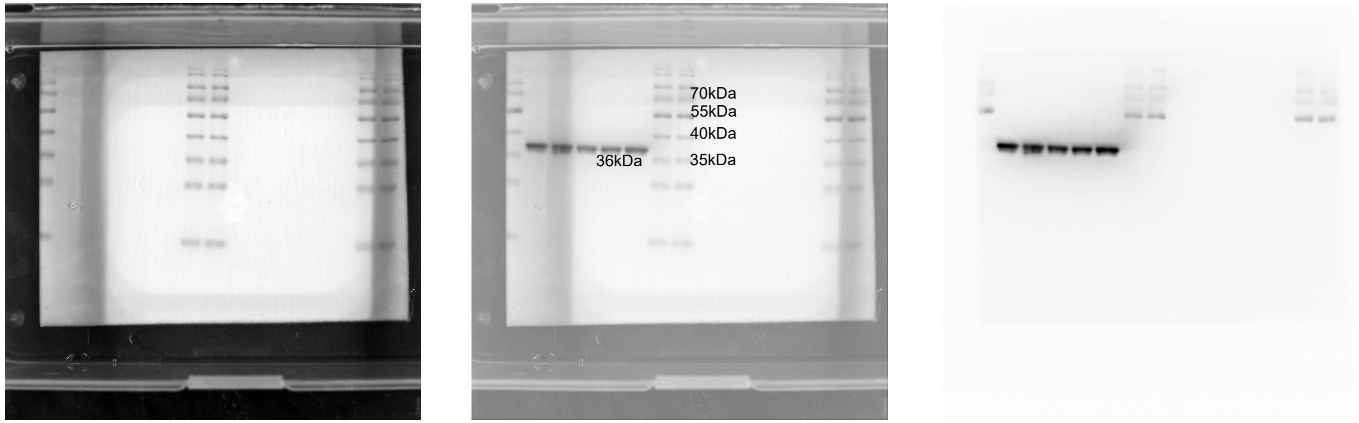

Cell; p-JNK; Repeat*3

Marker: ThermoFisher 26616

Group:Sham, Hemin, M2-Evs, miR-27n-3p cn, miR-27b-3p k/d

Figure. 9G

p-JNK

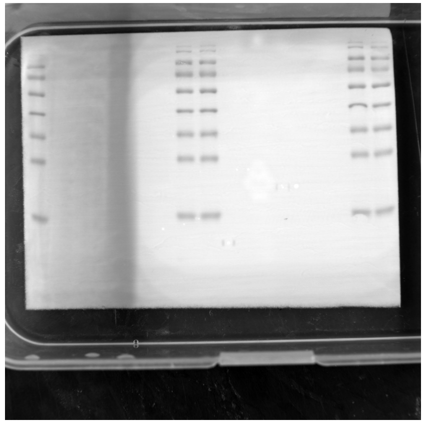

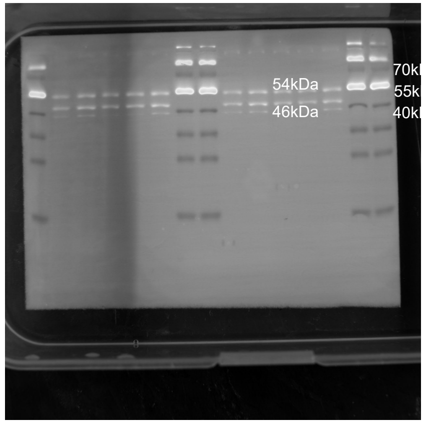

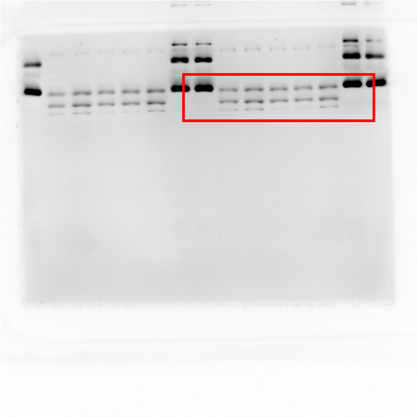

JNK

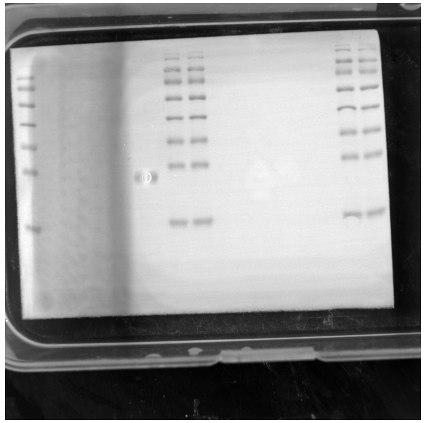

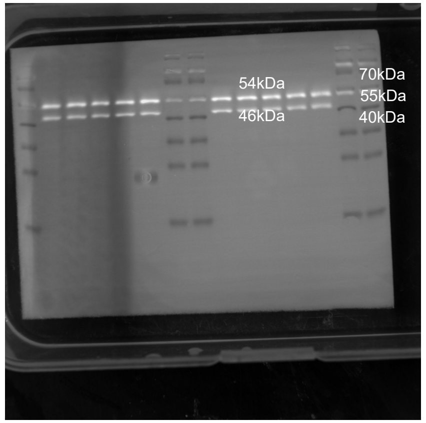

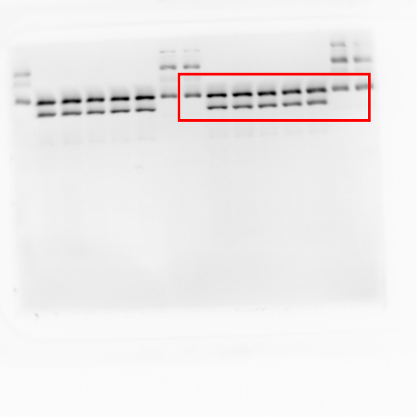

GAPDH

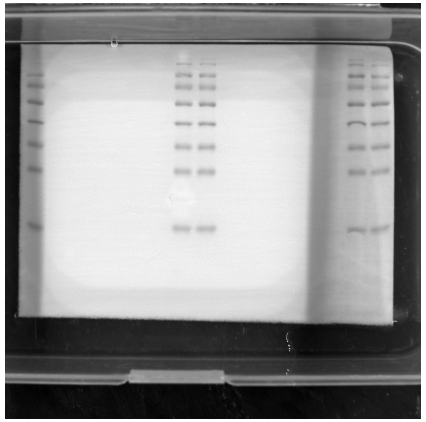

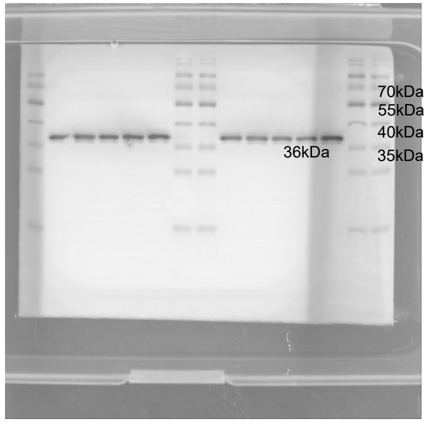

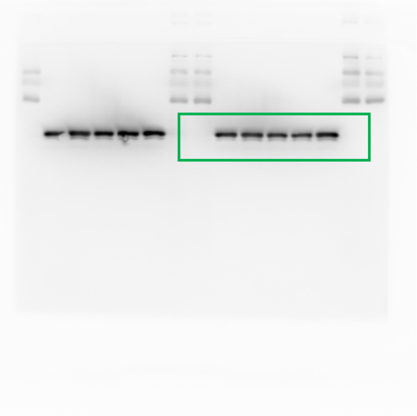

Marker: ThermoFisher 26616

p-JNK

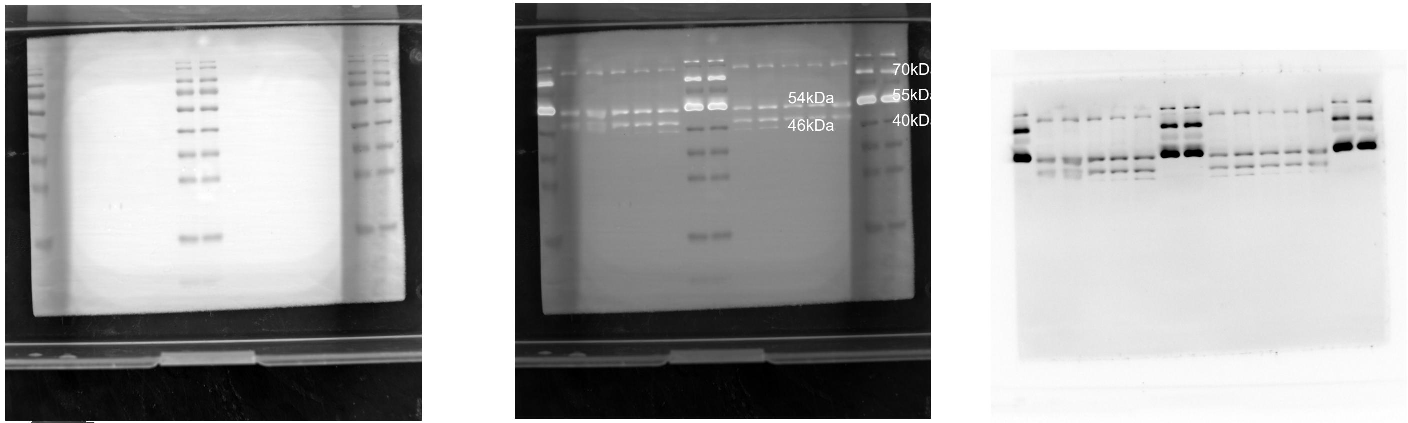

JNK

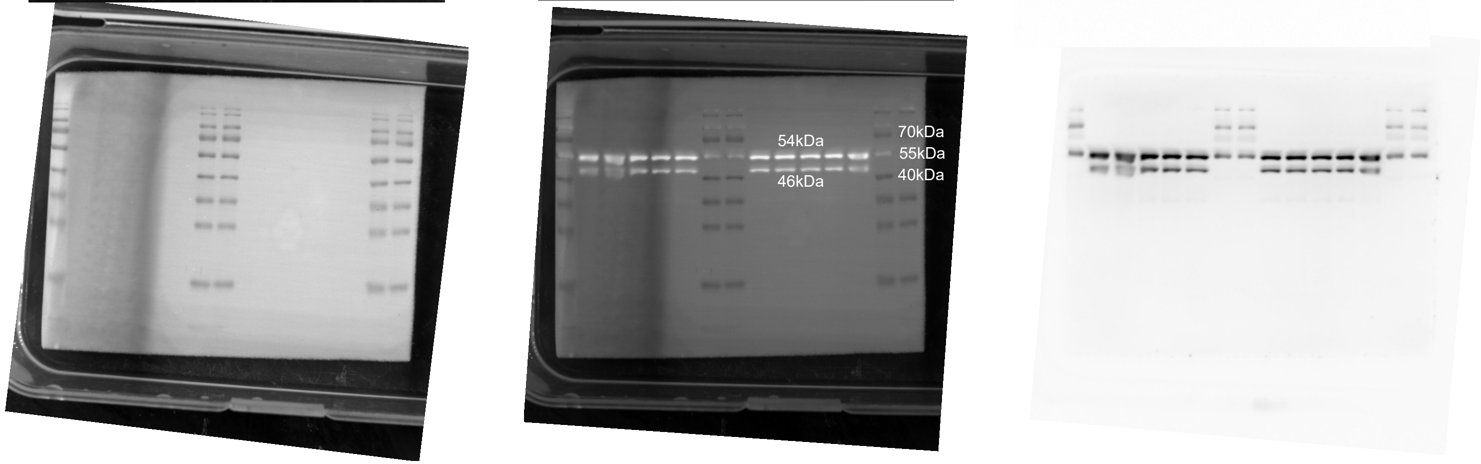

GAPDH

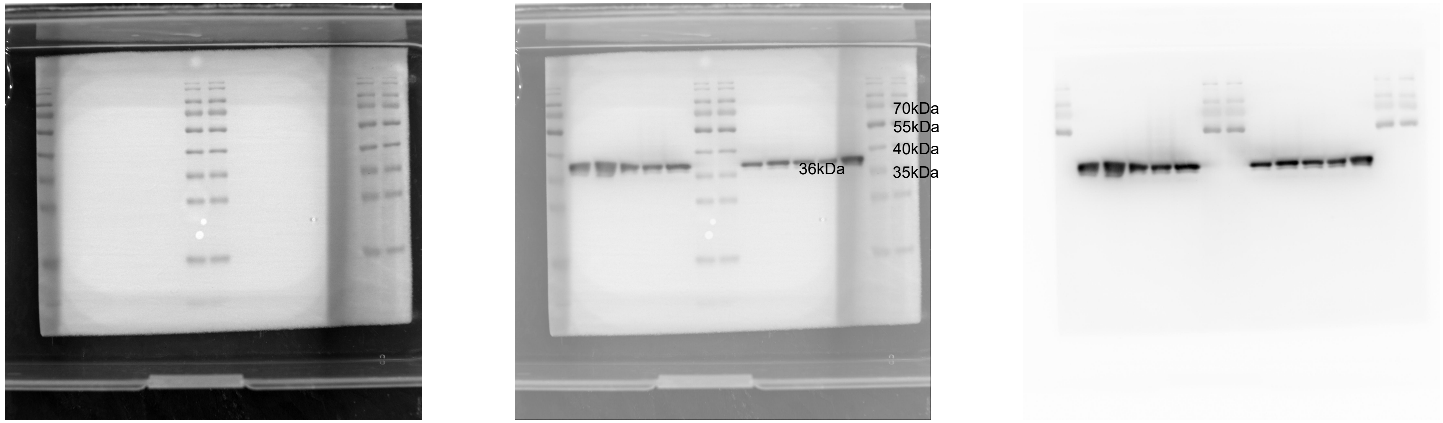

Mouse; p-MKK4; Repeat*6

Marker: ThermoFisher 26616

Group:Sham, Hemin, M2-Evs, miR-27n-3p cn, miR-27b-3p k/d

Figure. 9L

p-MKK4

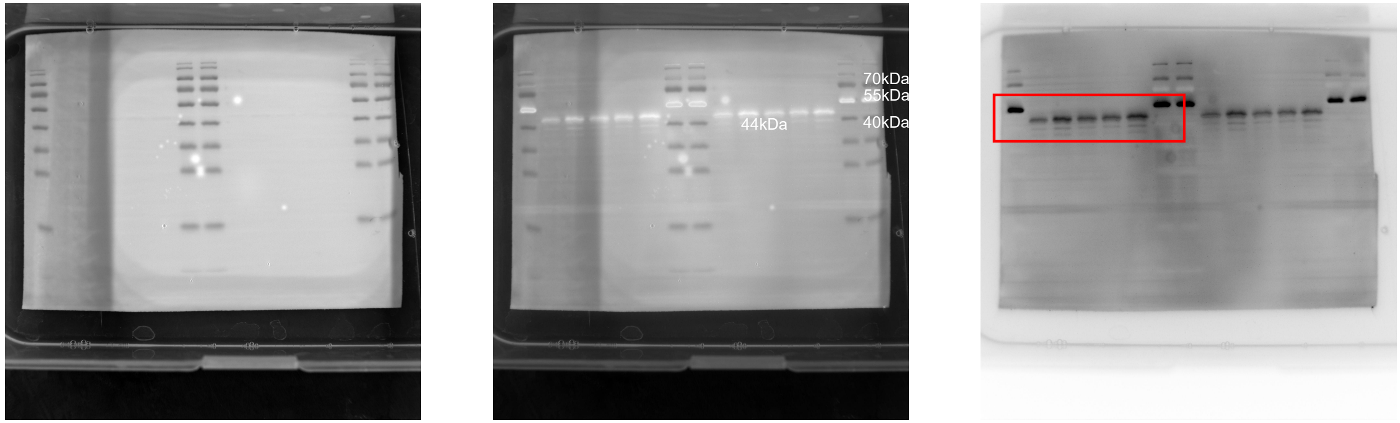

MKK4

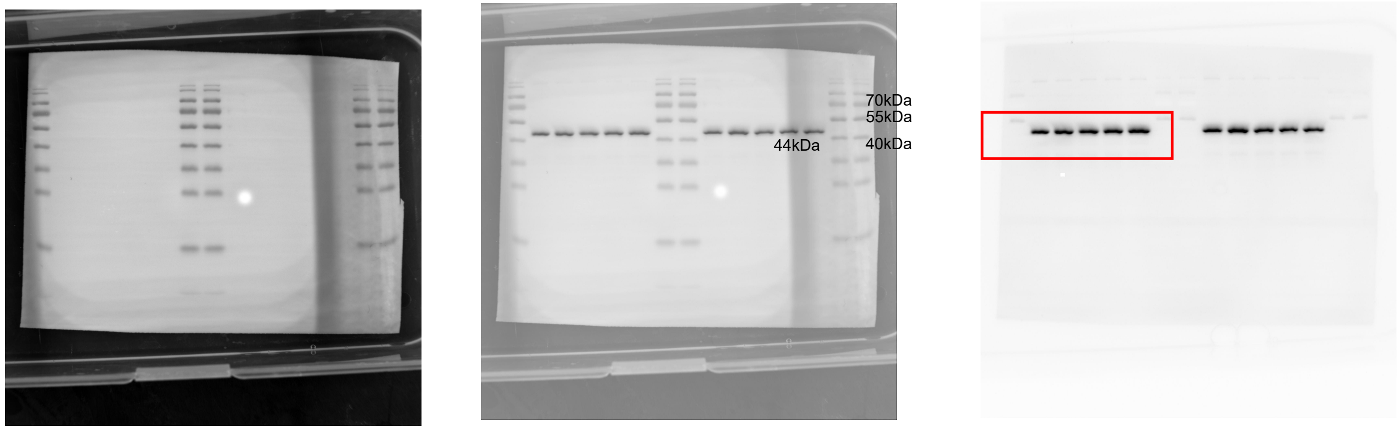

GAPDH

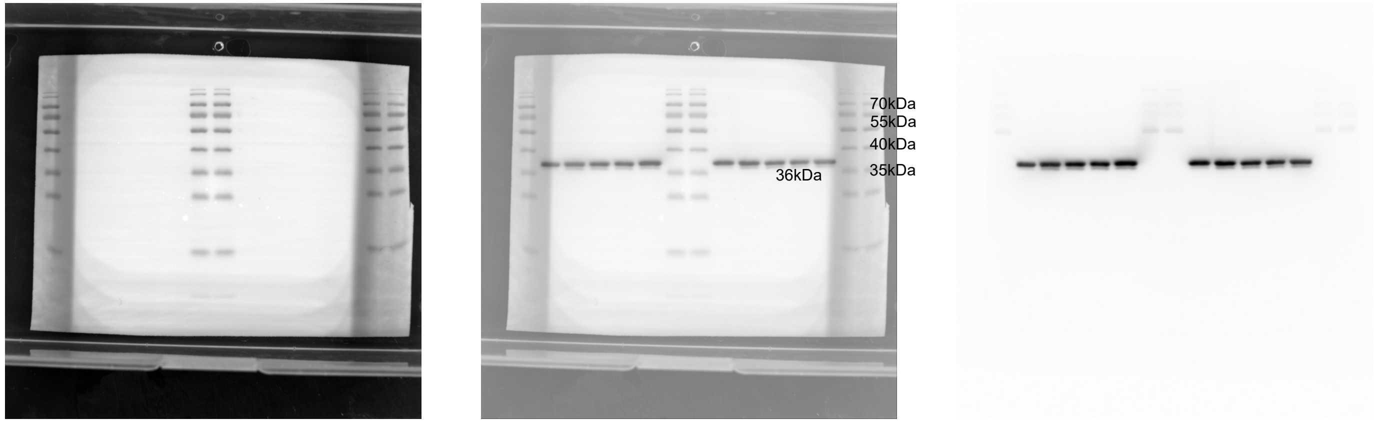

Marker: ThermoFisher 26616

p-MKK4

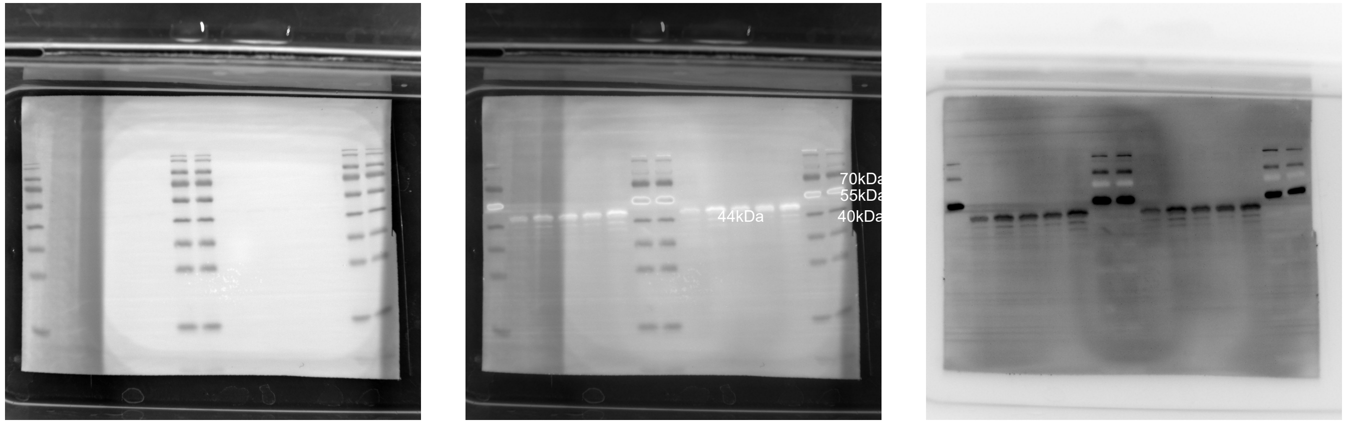

MKK4

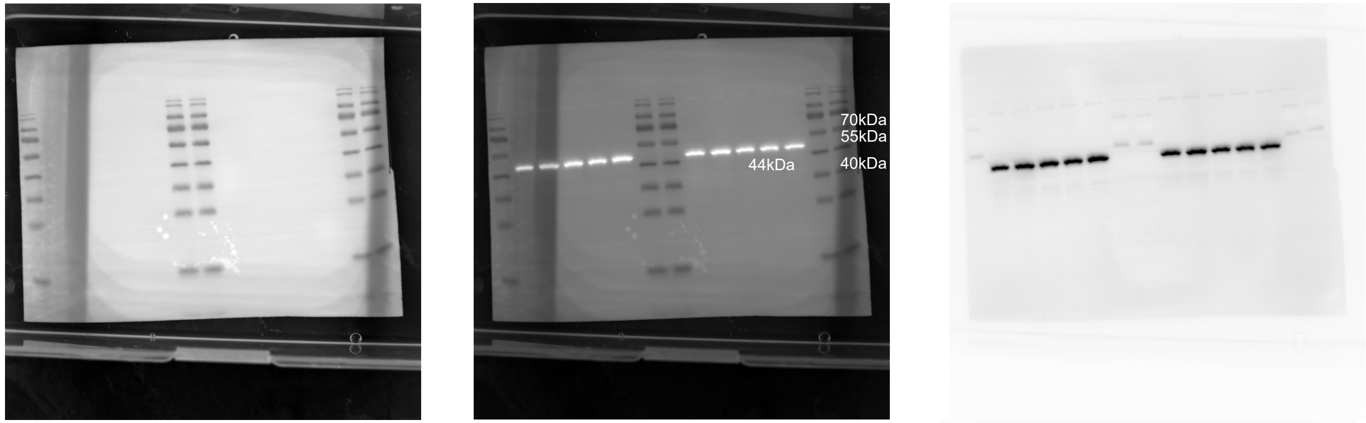

GAPDH

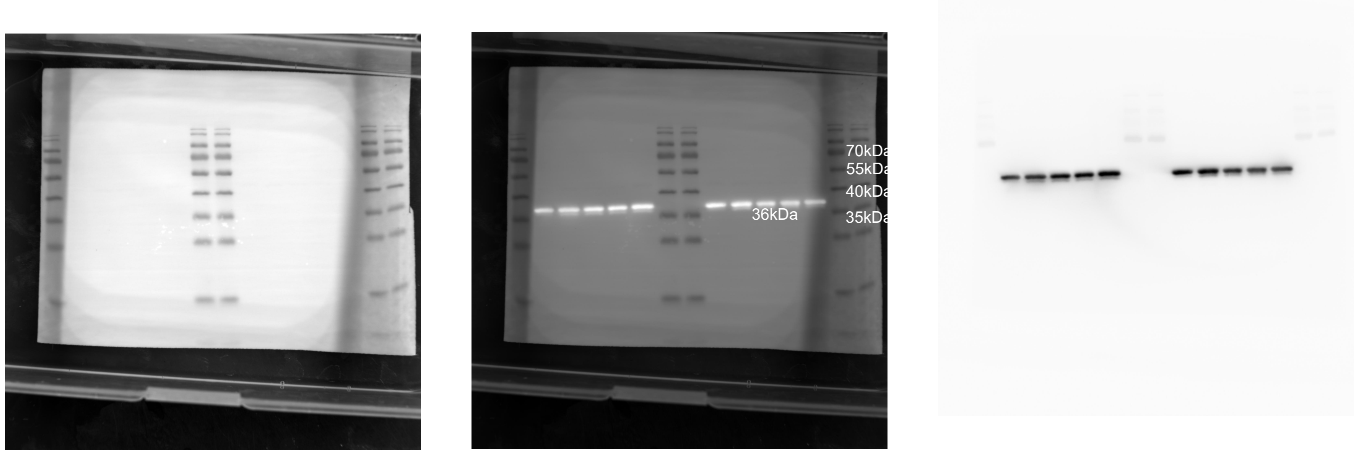

Marker: ThermoFisher 26616

p-MKK4

MKK4

GAPDH

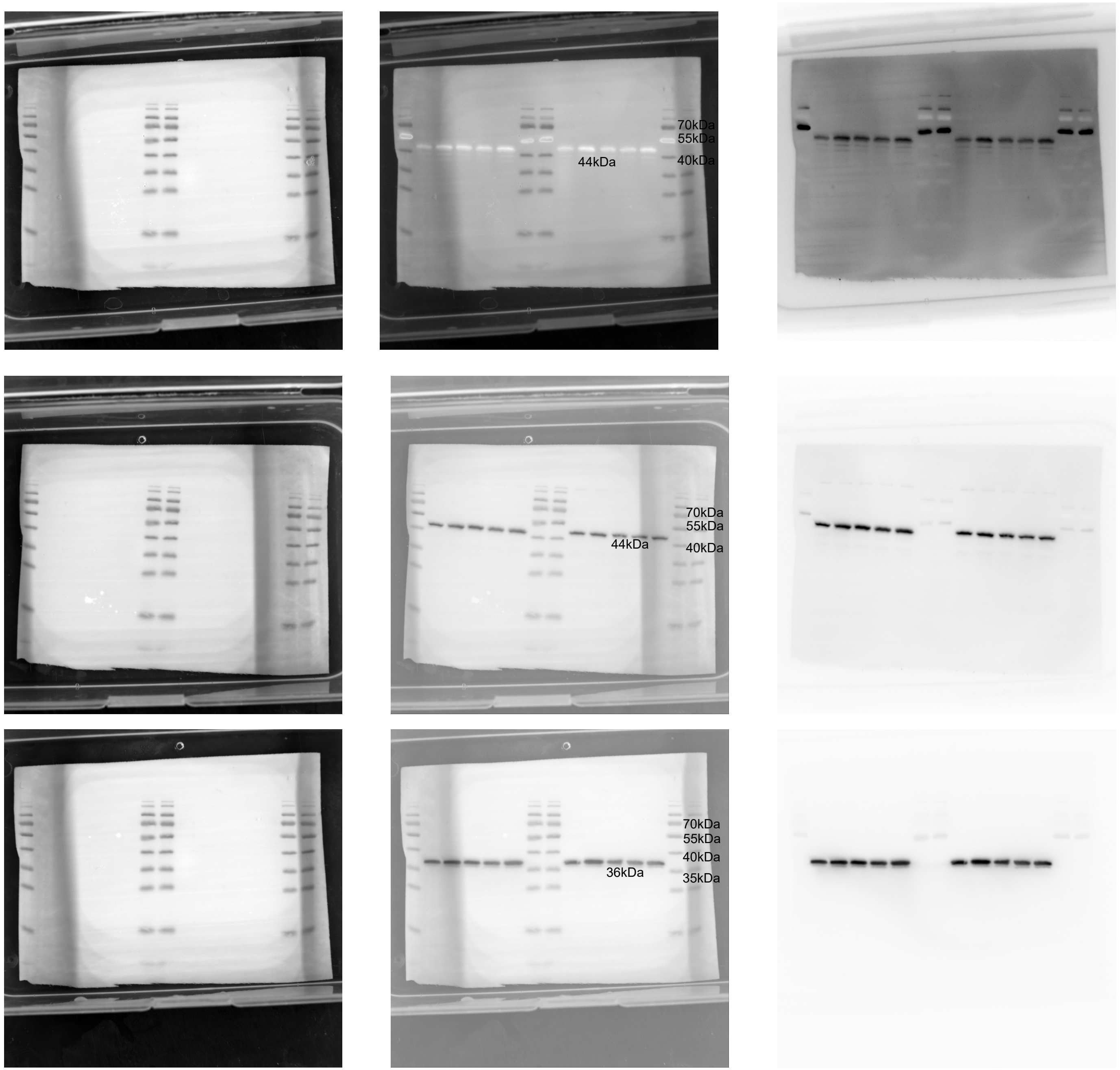

Mouse; p-JNK; Repeat*6

Group:Sham, Hemin, M2-Evs, miR-27n-3p cn, miR-27b-3p k/d

Figure. 9L

Marker: ThermoFisher 26616

p-JNK

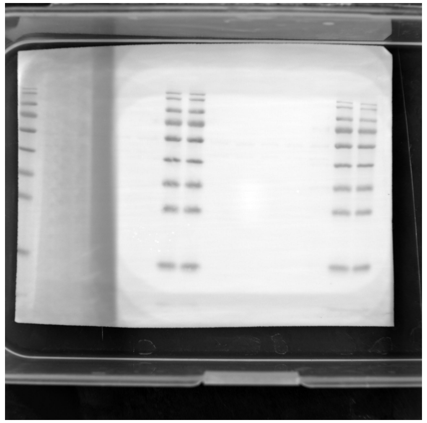

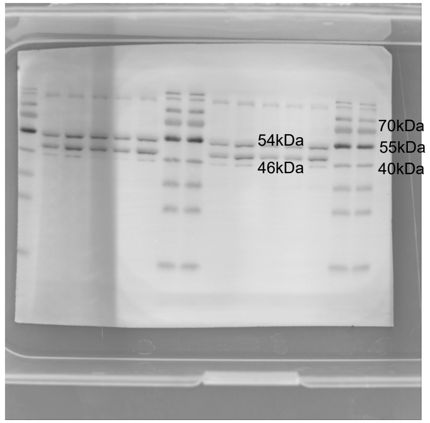

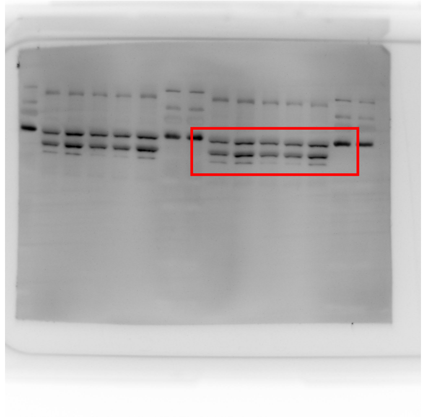

JNK

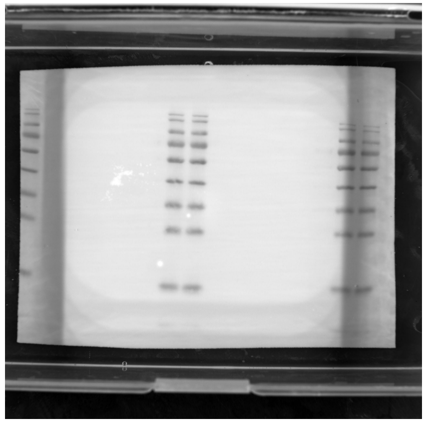

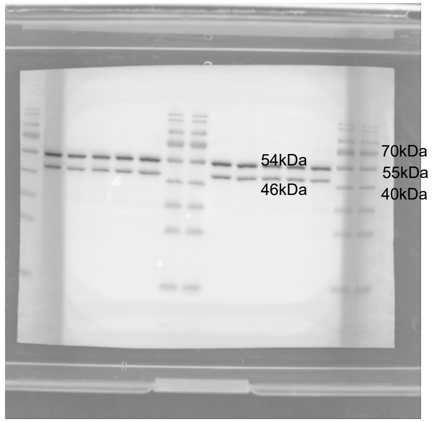

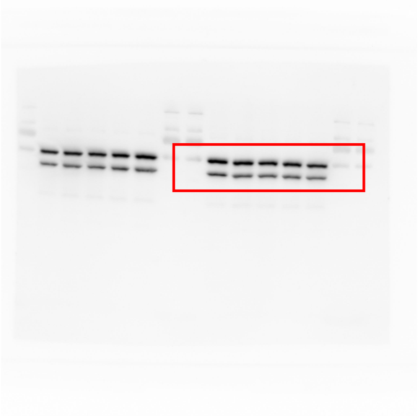

GAPDH

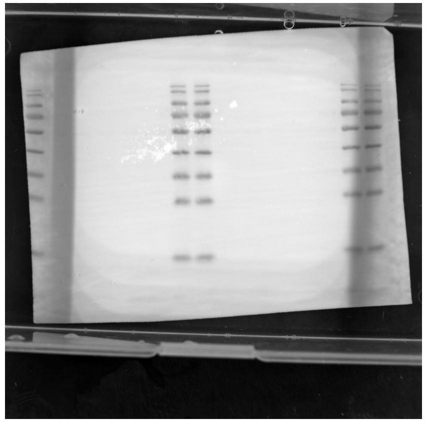

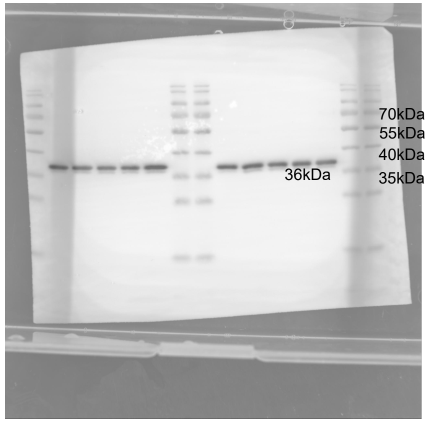

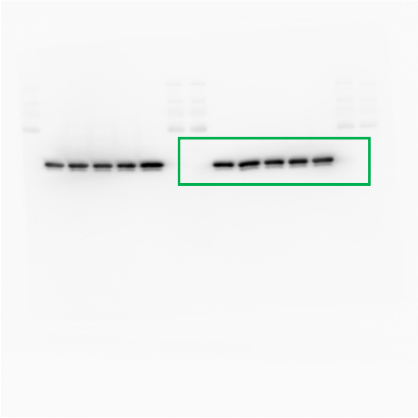

Marker: ThermoFisher 26616

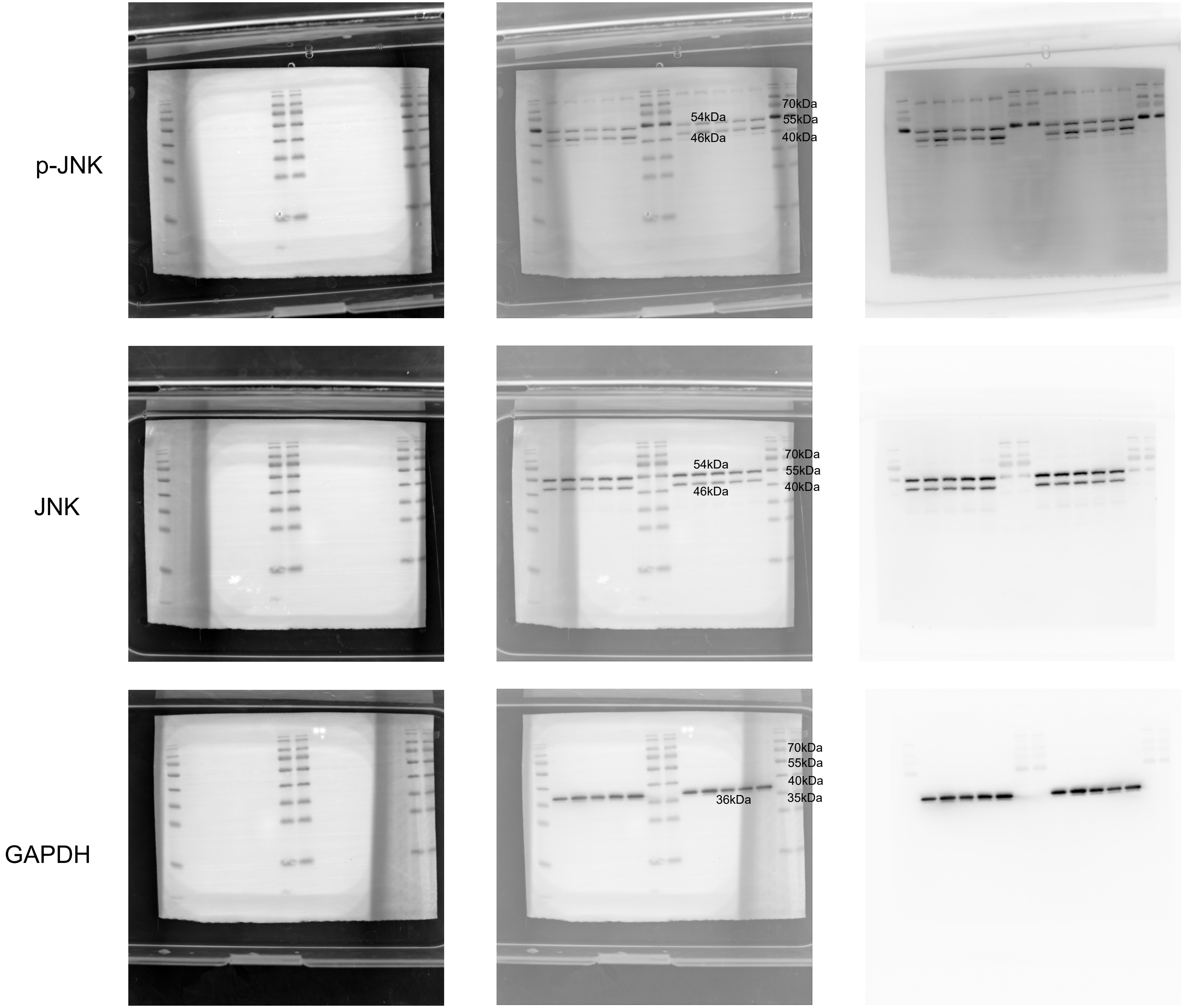

Marker: ThermoFisher 26616

p-JNK

JNK

GAPDH

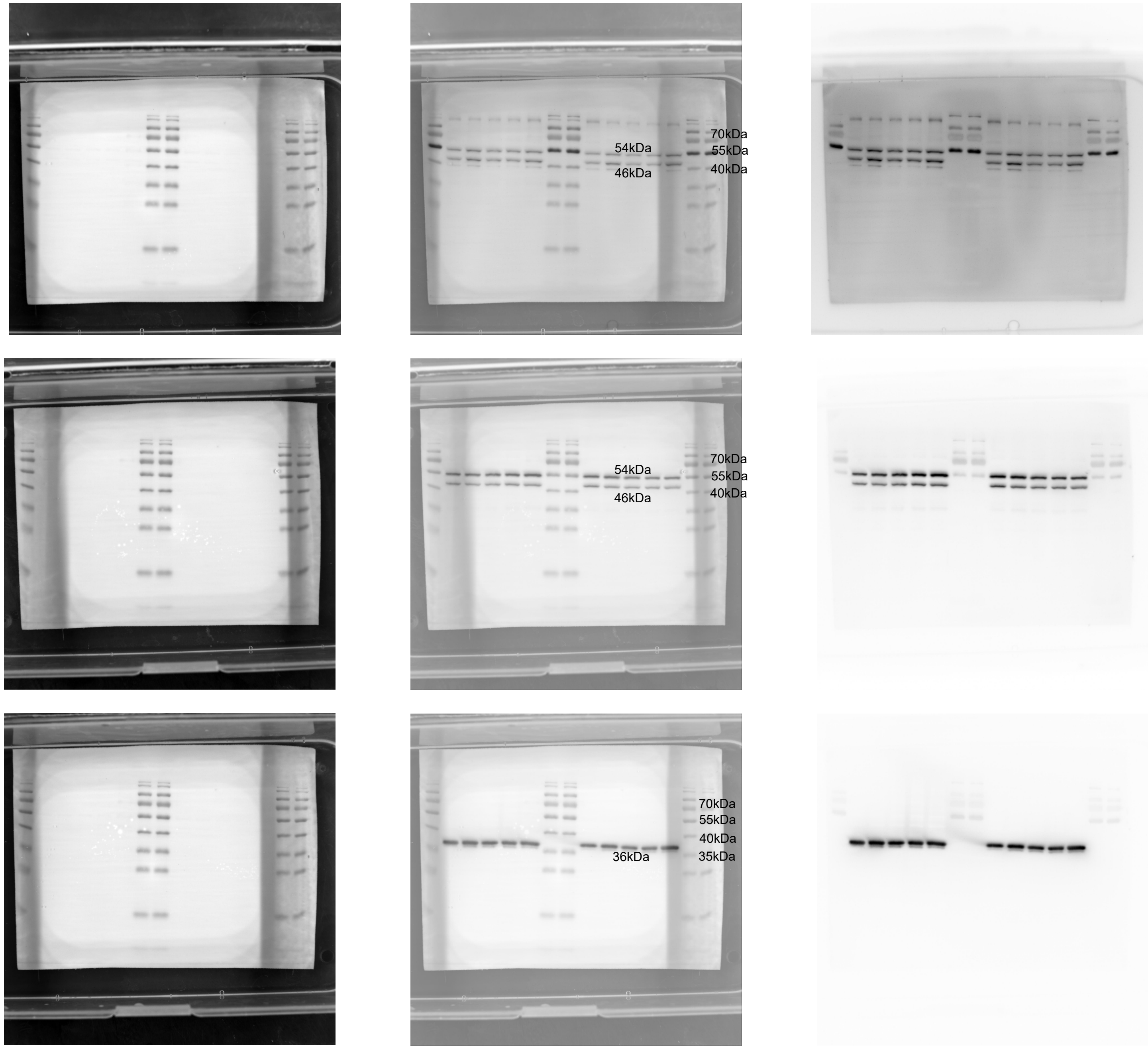

Supplement: Supplementary file 1 — (PDF 69.0 MB) [file 12035_2026_5718_MOESM1_ESM.pdf]
